# Widespread hydroxylation of unstructured lysine-rich protein domains by JMJD6

**DOI:** 10.1073/pnas.2201483119

**Published:** 2022-08-05

**Authors:** Matthew E. Cockman, Yoichiro Sugimoto, Hamish B. Pegg, Norma Masson, Eidarus Salah, Anthony Tumber, Helen R. Flynn, Joanna M. Kirkpatrick, Christopher J. Schofield, Peter J. Ratcliffe

**Affiliations:** ^a^Hypoxia Biology Laboratory, Francis Crick Institute, London, NW1 1AT, United Kingdom;; ^b^Ludwig Institute for Cancer Research, Nuffield Department of Clinical Medicine, University of Oxford, Oxford, OX3 7DQ, United Kingdom;; ^c^Chemistry Research Laboratory, Department of Chemistry, University of Oxford, Oxford, OX1 3TA, United Kingdom

**Keywords:** JMJD6, lysine hydroxylation, lysine-rich domain, 2-oxoglutarate-dependent dioxygenase, hypoxia

## Abstract

The Jumonji domain–containing protein JMJD6 is a 2-oxoglutarate–dependent dioxygenase associated with a broad range of biological functions. Cellular studies have implicated the enzyme in chromatin biology, transcription, DNA repair, mRNA splicing, and cotranscriptional processing. Although not all studies agree, JMJD6 has been reported to catalyze both hydroxylation of lysine residues and demethylation of arginine residues. However, despite extensive study and indirect evidence for JMJD6 catalysis in many cellular processes, direct assignment of JMJD6 catalytic substrates has been limited. Examination of a reported site of proline hydroxylation within a lysine-rich region of the tandem bromodomain protein BRD4 led us to conclude that hydroxylation was in fact on lysine and catalyzed by JMJD6. This prompted a wider search for JMJD6-catalyzed protein modifications deploying mass spectrometric methods designed to improve the analysis of such lysine-rich regions. Using lysine derivatization with propionic anhydride to improve the analysis of tryptic peptides and nontryptic proteolysis, we report 150 sites of JMJD6-catalyzed lysine hydroxylation on 48 protein substrates, including 19 sites of hydroxylation on BRD4. Most hydroxylations were within lysine-rich regions that are predicted to be unstructured; in some, multiple modifications were observed on adjacent lysine residues. Almost all of the JMJD6 substrates defined in these studies have been associated with membraneless organelle formation. Given the reported roles of lysine-rich regions in subcellular partitioning by liquid–liquid phase separation, our findings raise the possibility that JMJD6 may play a role in regulating such processes in response to stresses, including hypoxia.

## Significance

Enzyme-catalyzed posttranslational hydroxylation is increasingly recognized as affecting intracellular proteins and their functions, including in the signaling of hypoxic stress. The current work applies mass spectrometry methods to reveal more than 100 sites of lysine hydroxylation catalyzed by the 2-oxoglutarate–dependent dioxygenase JMJD6 in regions of the proteome that are refractory to conventional trypsin-based analyses. These sites were distributed across proteins involved at multiple levels in the control of gene expression. Targeted regions were strongly enriched for unstructured lysine-rich regions that have been associated with biomolecular condensates. The findings open routes to the understanding of the biological roles of JMJD6, including in subcellular reorganization in response to stress.

Enzymatic protein hydroxylation is increasingly recognized as a prevalent posttranslational modification with diverse roles in biology (for review see ref. 1). Classical studies on the biochemistry of collagen first identified the proline and lysine hydroxylation of residues in procollagen chains that are necessary for the assembly of collagen fibrils (for review see ref. 2). More recently, protein hydroxylation has been recognized to occur in other settings, including on intracellular proteins. For instance, proline hydroxylation was identified as the principal oxygen-sensitive regulatory modification on hypoxia-inducible factor (HIF), which transduces transcriptional responses to hypoxia (for review see ref. 3). These modifications are catalyzed by 2-oxoglutarate–dependent dioxygenases that split molecular oxygen and couple hydroxylation of the target amino acid residue to the oxidative decarboxylation of 2-oxoglutarate (1). Their catalytic Fe(II) center is susceptible to oxidative inactivation and under many circumstances, ascorbate is required for full catalytic activity. These properties potentially render the 2-oxoglutarate–dependent dioxygenases suitable for the sensing of hypoxic, metabolic, or oxidant stresses. Enzymatic hydroxylation has also been identified at specific sites on the side chains of asparagine, aspartate, arginine, histidine, and lysine residues in a range of other protein substrates (1, 4). These modifications are also catalyzed by enzymes within the 2-oxoglutarate–dependent oxygenase superfamily, many of which belong to an evolutionarily distinct group of small Jumonji C domain–containing (JMJD) proteins (1, 5). Although some of these small JMJD proteins have been reported to catalyze the demethylation of lysine or arginine residues, they differ from the majority of histone demethylases, both in terms of the architecture of the catalytic site and by the absence of an extensive set of protein domains beyond the catalytic domain and are believed to function principally as protein hydroxylases (5, 6).

One of the most interesting but enigmatic proteins is Jumonji domain–containing protein 6 (JMJD6). JMJD6 has been reported to be involved in a wide range of biological processes, including epigenetic regulation of histones, DNA repair, transcription elongation, stress responses, RNA metabolism, and mRNA splicing (for review see ref. 7). Although the mechanisms are not yet clear, it is believed to be important as an oxygen sensor regulating angiogenesis and placental function, as well as playing a role in the promotion of cancer (8, 9). JMJD6 has been reported to be both a protein lysine hydroxylase and an arginine demethylase (for review see ref. 10). However, many of its proposed biological functions have not been linked to an unequivocally identified catalytic target. In part, these uncertainties have arisen from difficulties in directly defining JMJD6-catalyzed protein modifications in cells (7). Although mass spectrometry potentially identifies protein hydroxylation as a +16-Da mass shift on peptide ions derived from the relevant substrate, interpretation can be confounded by artifactual oxidations occurring ex vivo. Mass spectrometry often lacks the required sensitivity for analyses of modifications on low-abundance proteins in cells. Furthermore, its application to the study of posttranslational modification can be restricted by the distribution of proteolytic cleavage sites and consequent difficulties in generating ions of an appropriate mass/charge ratio from some regions of the proteome, particularly those rich in lysine and arginine residues. Hence many studies of JMJD6-catalyzed protein modification have relied on in vitro analyses or indirect methods in cells.

Among the reported protein associations of JMJD6 is an interaction with the epigenetic “reader” bromodomain-containing protein 4 (BRD4), where it has been proposed to function in the regulation of transcription by pause release (11, 12). BRD4 binds acetylated lysine residues in histone tails via tandem bromodomains in the N-terminal region of the protein (13). These bromodomains are connected to the C-terminal regions of the molecule by intrinsically disordered regions, including a basic residue enriched interaction domain (BID). The BID in BRD4 has recently been reported to contain a site of proline hydroxylation catalyzed by the principal “oxygen sensing” HIF prolyl hydroxylase, PHD2 (proline hydroxylase domain isoform 2), otherwise known as EGLN1 (14, 15).

Further analyses of this region led us to conclude that, in the material we examined and within the limit of detection, the modification is not on proline and is not catalyzed by PHD2; rather that the region is subject to high stoichiometry hydroxylation on adjacent lysine residues catalyzed by JMJD6. Prompted by this finding and the general difficulty in assignment of protein oxidation in such regions, we performed an extensive analysis of the proteome for other regions that are modified by JMJD6 using a combination of affinity capture, derivatization, and mass spectrometric methods.

Here we report direct evidence that JMJD6 catalyzes high stoichiometry hydroxylation of lysine residues on numerous proteins. Using different methods of protein capture and mass spectrometric analyses in wild-type (WT) and genetically engineered JMJD6-defective cells, the work identifies more than 100 previously unknown sites of JMJD6-catalyzed lysine hydroxylation, including the hydroxylation of multiple consecutive residues in lysine-rich regions at a wide range of sites across the proteome.

## Results

**Fig. 1.**
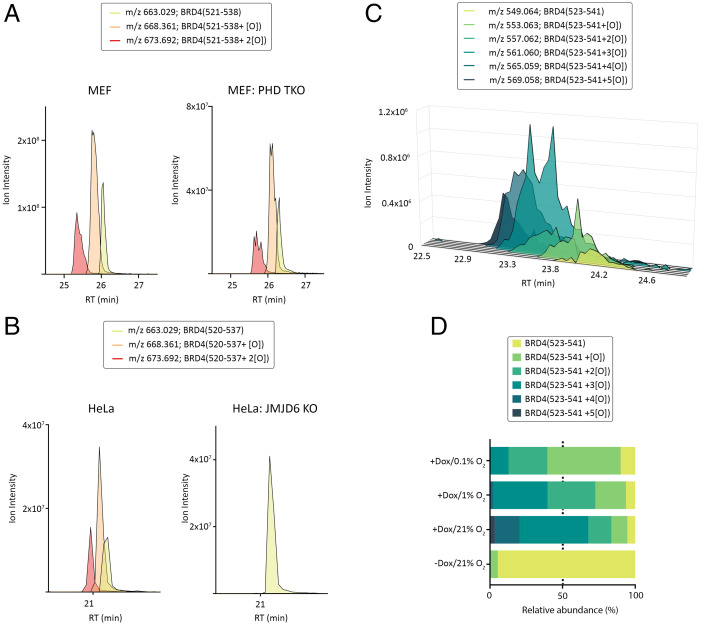
Oxygen-dependent hydroxylation of BRD4 by JMJD6. (*A*) XICs show peptide ion intensities corresponding to unoxidized (green-yellow), singly oxidized (orange), and doubly oxidized (red) forms of the tryptic BRD4(521 to 538) ([M+3H]^3+^) peptide isolated from MEFs. No difference in hydroxylation of BRD4 was observed between wild-type and PHD1 to 3 TKO cells. (*B*) XIC data for the identical peptide (residues 520 to 537 in the human protein) from HeLa cells. Hydroxylated BRD4(520 to 537) peptide ions are absent in material from JMJD6-defective HeLa cells. (*C*) XIC showing peptide ion intensity of unoxidized (yellow-green) and multiply hydroxylated (one to five oxidations; green to dark blue) forms of the Asp-N proteolysed BRD4(523 to 541) ([M+4H]^4+^) peptide isolated from SH-SY5Y cells. (*D*) Relative quantitation of BRD4(523 to 541) hydroxylation in response to hypoxic stress. Dox-inducible JMJD6 reexpressing cells (HeLa) were treated with dox and concurrently exposed to the indicated concentration of oxygen for 24 h. Area integrations of XICs were performed; relative peptide abundance data are presented as a stacked bar chart. A graded decrease in hydroxylation was observed with hypoxic stress; the 50th percentile value is highlighted to denote the median site occupancy (i.e., number of oxidations) at the specified oxygen concentration. Oxidation detected in the minus dox condition represents low-level (leaky) JMJD6 expression.

In an attempt to identify novel substrates of the 2-oxoglutarate–dependent oxygenase family, we performed an extensive survey of highly fractionated tryptic “deep proteome” datasets (12.6 million assigned tandem mass spectrometry [MSMS] spectra) (16–18), searching for signatures of enzyme-catalyzed hydroxylation in the human proteome. Among the candidates identified, singly and doubly oxidized peptides corresponding to residues 520 to 537 of human BRD4 were frequently assigned by mass spectrometry (MS) database search algorithms (*n* = 85 oxidized peptides). Probability scores for the site of modification favored assignment of hydroxylysine at the peptide C terminus, but hydroxylation of the adjacent proline residue could not be excluded (see *SI Appendix*, Table S1 for summary of analyses). Of note, proline hydroxylation at this site (P536) had previously been reported and was subsequently assigned as being catalyzed by the HIF proline hydroxylase, PHD2 (14, 15). Difficulties confirming the reported substrate repertoire of the HIF proline hydroxylases (19), together with the observation that lysine hydroxylation was favored by our algorithm for localization of posttranslational modification, led us to reevaluate the assignment of PHD2-catalyzed proline hydroxylation. We immunoprecipitated endogenous BRD4 protein from mouse embryonic fibroblasts (MEFs) in which each of the three HIF proline hydroxylases (PHD1 to PHD3) have been inactivated, termed triple knockout (TKO) (20), and compared the hydroxylation status of the target peptide by liquid chromatography tandem mass spectrometry (LC-MSMS) with control wild-type MEFs (*SI Appendix*, Fig. S1 for validation of MEFs). Peptides corresponding to unmodified, singly and doubly oxidized BRD4 were assigned by MSMS in both wild-type and TKO cell preparations (*SI Appendix*, Fig. S2, for representative MSMS from wild-type MEFs). Peptide abundance measurements by extracted ion chromatogram (XIC) revealed no difference in the hydroxylation status of the target peptide in wild-type versus TKO (PHD-defective) MEFs, suggesting that prolyl hydroxylation might have been misassigned (Fig. 1*A*). The alternate assignment of hydroxylysine raised a hypothesis that JMJD6, a lysine hydroxylase that is known to physically associate with BRD4, was the targeting enzyme in cells (11, 12). To test this possibility, we performed an analogous set of experiments, comparing the hydroxylation status of BRD4 in wild-type HeLa cells to a subline in which the catalytic activity of JMJD6 had been inactivated by CRISPR/Cas9-mediated gene ablation. Significantly, hydroxylated peptide ions were neither assigned by MSMS nor detected in XICs derived from JMJD6-defective HeLa cells, indicating a nonredundant function for JMJD6 in catalyzing hydroxylation on this peptide (Fig. 1*B*; see *SI Appendix*, Fig. S3 for representative MSMS from HeLa). Quantitation of diagnostic fragment ions by parallel reaction monitoring assigned K535 and K537 as the sites of hydroxylation (*SI Appendix*, Fig. S4).

Based on these results we sought to determine whether JMJD6-catalyzed lysyl hydroxylation occurred on related BRD proteins. Analysis of tryptic peptides from anti-BRD2 and anti-BRD3 immunoprecipitates performed in wild-type and JMJD6-defective HeLa cells revealed that JMJD6-catalyzed hydroxylation extended to a homologous peptide sequence in the related bromodomain proteins, BRD2-3 (*SI Appendix*, Fig. S5 for BRD2-3 XICs).

When the sequence context of the hydroxylation sites in BRD4 were considered, it was noted that the target lysine residues (K535/7) form the start of a lysine-rich sequence that is ∼50 residues long and incompletely resolved by mass spectrometry using standard trypsin digestion, which cleaves after lysine and arginine. To improve the peptidic coverage, BRD4 was immunoprecipitated and digested with proteases with orthogonal specificity to trypsin, including Asp-N, which cleaves before aspartate and to a lesser extent before glutamate residues. An Asp-N proteolyzed peptide, corresponding to residues 523 to 541, was detected that contained five lysine residues at the C terminus. Multiply oxidized forms of this peptide were detected by LC-MSMS with an unprecedented five hydroxylations assigned in both SH-SY5Y cells (Fig. 1*C* and *SI Appendix*, Fig. S6 for MSMS) and HeLa cells. The level of hydroxylation was found to be an order of magnitude higher than any previously assigned JMJD6 substrate; over 95% of BRD4(523 to 541) peptides were hydroxylated, with triply oxidized peptides the predominant species.

These experiments strongly suggested that JMJD6 catalyzes the hydroxylation of multiple lysine residues in BRD4. To demonstrate this directly, we reacted a peptidyl substrate corresponding to residues 511 to 550 of BRD4 with recombinant JMJD6 in vitro and analyzed the products by LC-MS. In this way, we directly demonstrated the catalytic activity of JMJD6 on BRD4 as revealed by multiple hydroxylations that were suppressed by the oxoglutarate analog and catalytic inhibitor 2,4-pyridinedicarboxylic acid (*SI Appendix*, Fig. S7).

To test the oxygen dependence of JMJD6-catalyzed hydroxylation in cells, we then monitored hydroxylation of the Asp-N proteolyzed BRD4(523 to 541) peptide in a HeLa cell line in which JMJD6 had been inactivated by CRISPR/Cas9 genetic modification, but then reexpressed under the control of a doxycycline (dox)-inducible promoter. This enabled JMJD6 reexpression in parallel with hypoxic exposure, permitting the specific assay of newly catalyzed hydroxylation at different oxygen concentrations. XIC-based quantitation revealed a graded response to reduced oxygen; the median number of hydroxylation sites assigned to each condition decreased from three in normoxia to two at 1% oxygen and one at 0.1% oxygen (Fig. 1*D*).

**Fig. 2.**
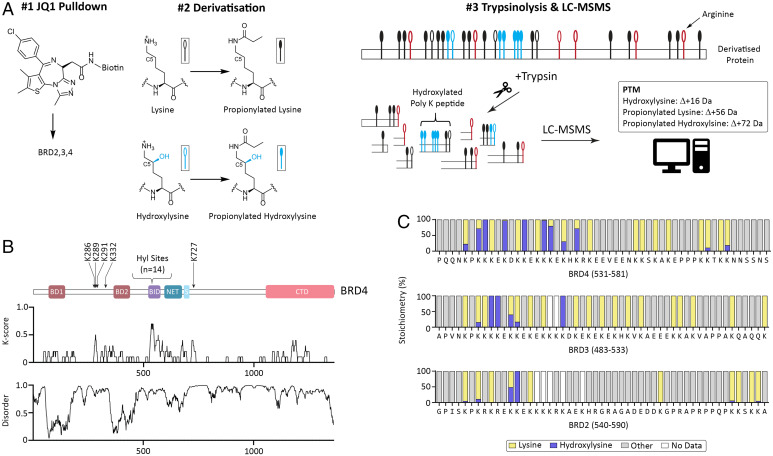
The BIDs of bromodomain proteins are polyhydroxylated by JMJD6. (*A*) Schematic of affinity enrichment and chemical derivatization: #1 Bromodomain proteins are enriched by JQ1 pulldown; #2 lysine/hydroxylysine residues are derivatized by addition of propionic anhydride; #3 derivatization blocks trypsinolysis (trypsin only cleaves at arginine/nonderivatized lysine residues) to yield lysine-rich peptides for MS analysis. (*B*) Summary of BRD4 hydroxylations (*Upper*; domain structure of BRD4: BD, bromodomain; CTD, C-terminal domain); extensive hydroxylation (14 sites) was observed in the BID, which is both lysine rich (*Middle*; K-score: the proportion of lysine residues in a 10-residue sliding window) and predicted to be structurally disordered (*Lower*; disorder determined by IUPred2A prediction tool) (46). (*C*) Stoichiometry of hydroxylation on the indicated residues of BRD4 (*Upper*), BRD3 (*Middle*), and BRD2 (*Lower*) in HEK293 cells. Note, low stoichiometry hydroxylations (<1%) cannot be visualized; see *SI Appendix*, Table S2 for site assignments.

Aside from the identification of the highly hydroxylated peptide in BRD4 that is observed with Asp-N digestion, peptide coverage of the lysine-rich domain in BRD4 remained low with all of the proteases that we tested. We therefore developed an alternative strategy to increase coverage. First, BRD4 protein yield was increased by switching from antibody to probe-based affinity enrichment; JQ1, a small molecule inhibitor with nanomolar affinity toward bromodomain proteins (BRD2 to BRD4) (21), was linked to biotin and immobilized on streptavidin beads for purification of endogenous bromodomain proteins. Second, and most importantly, lysine residues were derivatized by addition of propionic anhydride to yield propionyl lysine, prior to digestion with trypsin. Propionylation of the ε-amino group of lysyl residues serves a dual purpose: neutralizing the charge of the primary amine to increase hydrophobicity and peptide retention on liquid chromatography and inhibiting trypsin cleavage of modified lysine residues, giving rise to longer peptides that are better suited for LC-MSMS workflows (22). Proteolysis persists at arginine and nonderivatized lysine residues (i.e., derivatization is incomplete) to yield lysine-rich peptides for MS analysis (see Fig. 2*A* for schematic overview). Database search strategies were modified to take into consideration the increase in search complexity, with propionylation and hydroxylation as nonexclusive modifications. It was not possible, however, to customize the cleavage specificity of trypsin toward propionylated lysine in the search engine (PeaksDB; Peaks Studio). Thus, in order to assign lysine-rich peptides, database searches were performed, allowing an unusually high (*n* = 5) number of missed tryptic cleavages, which impacted on the false discovery rate and suppressed peptide significance scoring despite high-quality MSMS data. To reduce the search complexity, we therefore restricted the database inquiry to those proteins identified in JQ1 pulldowns of nonderivatized cell lysate by conventional MS analysis. Search results were filtered postprocessing to remove peptide assignments that were inconsistent with the trypsin cleavage specificity rules that would be anticipated after derivatization. Thus, peptides with more than one “true” missed cleavage (i.e., at a nonderivatized lysine residue) and peptides bearing a propionylated lysine residue at the C terminus were discarded from subsequent analyses (see *SI Appendix*, *Supplementary Materials and Methods* for full description of processing workflow).

Despite these challenges, this workflow provided clear insight into the extent of JMJD6-catalyzed hydroxylation of BRD2 to BRD4. MS data collected across HeLa, HEK293, and MCF7 cells assigned 19 hydroxylation sites in BRD4, with the majority (*n* = 14) localized to the lysine-rich and structurally disordered BID, encompassing residues 524 to 579 of BRD4 (Fig. 2*B* for schematic summary of HEK293 data). The stoichiometry of hydroxylation, resolved to specific lysine residues, was determined using peptide ion area counts. At sites that could be detected with both procedures, the residue-specific values for the level of hydroxylation were comparable between propionylated peptides and those obtained by Asp-N without derivatization (*SI Appendix*, Fig. S8), verifying the method. Remarkably, despite the low sequence complexity, when the stoichiometry of hydroxylation on N-terminal residues of the BID in BRD4 was compared across the three different cell lines a very similar pattern was observed at individual sites in each cell type (*SI Appendix*, Fig. S9). Near complete hydroxylation was also observed at multiple sites in BRD2 and BRD3, although these proteins contain fewer lysine residues and fewer sites of hydroxylation than BRD4 (Fig. 2*C*).

**Fig. 3.**
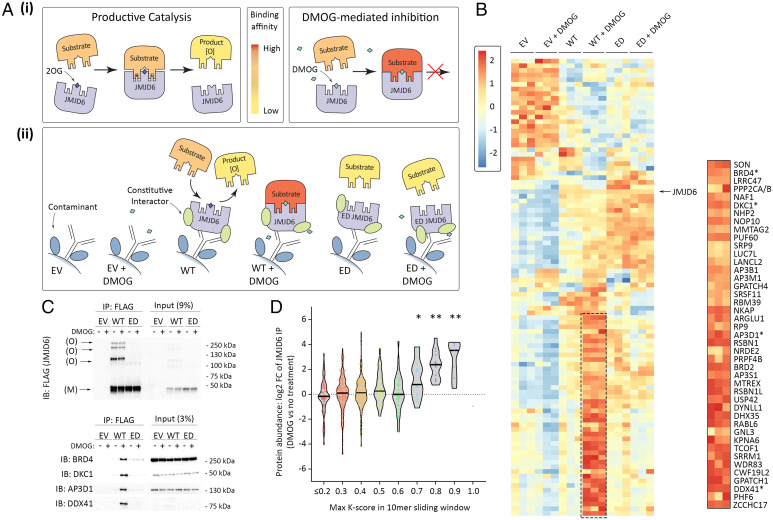
Substrate-trapping identifies multiple lysine-rich proteins as putative JMJD6 substrates. (*A*) Schematic of JMJD6 catalysis and substrate-trapping with DMOG. (*i*) DMOG, a prodrug that is hydrolysed in cells to N-oxalylglycine (a 2OG analog), inhibits catalysis and stabilizes JMJD6 substrate interactions; fill color of substrate represents relative binding affinity. (*ii*) Summary of experimental design. (*B*) Summary of experimental results; heatmap shows z-score of log2 transformed protein abundance values corresponding to each copurified protein across replicates (*n* = 3) and experimental conditions. Color indicates z-score; DMOG treatment was for 9 h. Identity of species manifesting DMOG-inducible interaction with JMJD6 is given in the expanded view on the *Right*. (*C*) Validation of selected JMJD6 interactions as defined in *B* (*) by immunoprecipitation and immunoblotting. *Upper*, equivalent capture of JMJD6 monomer (M); oligomers (O) visible as more slowly migrating species; *Lower*, enhanced capture of the indicated JMJD6-interacting species with DMOG (+). (*D*) DMOG selectively enriches association of lysine-rich proteins with JMJD6. JMJD6-interacting proteins were grouped according to the maximum number of lysine residues in a 10-residue sliding window (“max K-score”). Violin plots show DMOG-dependent interaction as a function of the max K-score. Horizontal bars indicate median values. Statistical significance was determined by reference to species with a maximum K-score of ≤0.2 using the Mann–Whitney *U* test. *P < 0.05, **P < 0.005. *P* values were adjusted for multiple comparisons using Holm’s method.

These findings led us to consider the wider existence of JMJD6-catalyzed hydroxylation of lysine-rich sequences. To identify putative substrates, we first performed a protein interaction screen with JMJD6 that employed substrate-trapping with dimethyloxalylglycine (DMOG), a broad-spectrum inhibitor that is known to enhance some 2-oxoglutarate–dependent oxygenase/substrate associations (Fig. 3 *A*, *i*) (23). Optimal conditions for substrate trapping were defined in advance of the protein interaction screen by assaying the interaction between JMJD6 and BRD4 by immunoprecipitation and immunoblotting. Increased immunoprecipitation of BRD4 was observed in DMOG-treated cells and found to be maximal in cells pretreated with 1 mM DMOG for 9 h. Accordingly, these conditions were used in experiments performed in HeLa cells in which JMJD6 had been inactivated by CRISPR/Cas9, then rescued by lentiviral delivery of FLAG-tagged transgenes encoding WT JMJD6 or a catalytically inactive (enzyme dead [ED]) form bearing mutations in residues coordinating the catalytic iron, i.e., H187A and D189A or empty vector (EV) control (Fig. 3 *A*, *ii*). Affinity-purified FLAG-JMJD6 complexes were digested with trypsin and biological replicates (*n* = 3) were analyzed by MSMS in data-independent acquisition mode. The relative abundance of interacting proteins was determined by label-free quantification. Immunoprecipitated proteins displaying significant changes between conditions are presented in a heatmap with protein targets (annotated in rows) ordered by hierarchical clustering. The heatmap depicts the relative enrichment of JMJD6-associating proteins in the presence or absence of DMOG (Fig. 3*B*). Visualizing the data in this way revealed little change in the level of JMJD6 bait detected across WT and ED pulldowns. This contrasted with a subset of coprecipitating proteins (dashed box and *Inset*) that displayed enhanced DMOG-dependent binding to JMJD6 that was dependent on the integrity of the catalytic site. Significantly, BRD2 and BRD4, and previously assigned peptidyl substrates, LUC7L (Luc7-like pre-mRNA splicing factor) and SRSF11 (serine- and arginine-rich splicing factor 11) (24), were among the ∼40 proteins displaying these characteristics. The accuracy of the MS-based quantitation was confirmed by immunoprecipitation and immunoblot analysis of a number of JMJD6-associated proteins: DKC1 (dyskeratosis congenita 1), AP3D1 (adaptor-related protein complex 3 subunit delta 1) and DDX41 (DEAD-box helicase 41), displayed marked DMOG-dependent enrichment in immunoprecipitations from WT FLAG-JMJD6 cells (Fig. 3*C*). Interestingly, many of the proteins identified in the JMJD6 interactome, particularly those showing high-level enrichment with DMOG, contained lysine-rich regions similar to those targeted for JMJD6-catalyzed hydroxylation in the bromodomain protein substrates. To assess this statistically, we examined the relationship between lysine content of the polypeptide sequence, expressed as the maximum proportion of lysine residues in a 10-residue sliding window across the protein (referred to as the K-score) and the level of enrichment in JMJD6 immunoprecipitates from DMOG-treated versus untreated cells. This confirmed a highly significant association with proteins containing regions with high K-scores (between 0.7 and 0.9; Fig. 3*D*).

**Fig. 4.**
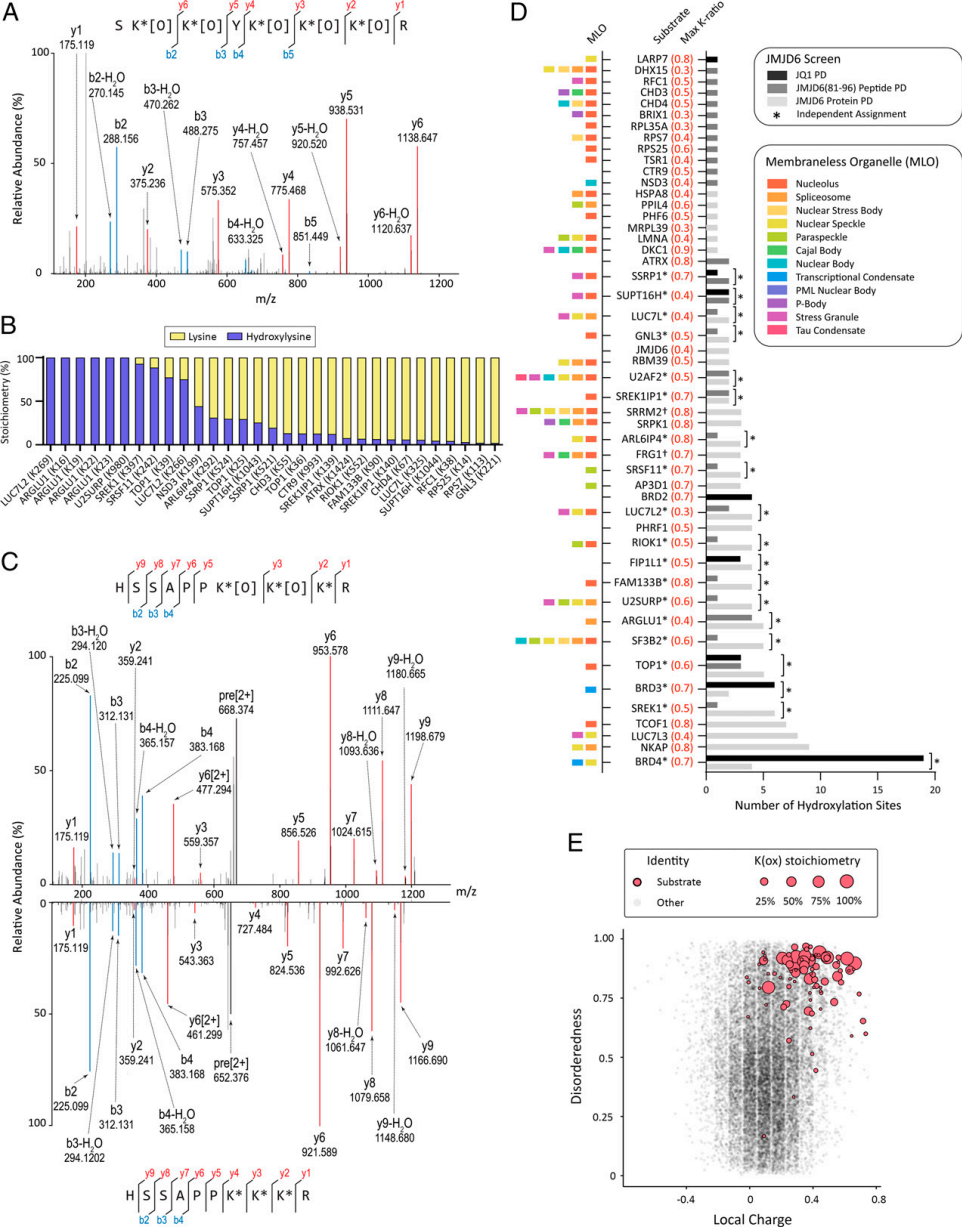
JMJD6 is a polylysyl hydroxylase targeting a broad range of substrates. (*A*) MSMS analysis illustrating polyhydroxylation of a lysine-rich region in NKAP. Fragment ions are from a peptide corresponding to residues 246 to 253, obtained following derivatization with propionic anhydride and digestion with trypsin. Assigned ions are annotated; *derivatized lysine, [O] site of hydroxylation. (*B*) Stoichiometry of JMJD6-dependent hydroxylation identified by JMJD6(81 to 96) peptide affinity purification from wild-type and JMJD6-defective HeLa cells. (*C*) MSMS mirror plot of FACT complex subunit SPT16 (SUPT16H, residues 1037 to 1046) peptide in doubly hydroxylated (*Upper*) and unmodified (*Lower*) form derived from wild-type and JMJD6-defective HeLa cells, respectively. (*D*) Summary of hydroxylysine assignments from assembled screens. Columns depict the number of hydroxylation sites assigned to JMJD6 targets. Numbers in parentheses (red) give the maximum K-score for each protein; ^†^nonunique hydroxylated peptide. Colored bars summarize reported associations (PhaSepDB) (47) of each protein with different membraneless organelles (MLO). (*E*) Bubble plot showing the relationship between JMJD6-catalyzed hydroxylation (red bubble; scaled by stoichiometry hydroxylation at the target site), the probability of disorderedness (determined by the IUPred2A prediction tool) (46), and calculated charge in the vicinity (−5 to +5 residues) of the hydroxylation site. Data combines material from JQ1 and JMJD6(81 to 96) peptide affinity experiments in HeLa cells. Control values for lysine are presented as background scatterplots (gray dots) derived from all MSMS assignments for these experiments in material from wild-type HeLa cells. Compared to control, JMJD6-catalyzed hydroxylysine shows a significant bias toward unstructured regions bearing strong positive charge (*P* < 10^−10^ as determined by two-sided Mann–Whitney *U* test).

To pursue direct evidence for JMJD6-catalyzed hydroxylation at these sites, we modified the FLAG-JMJD6 affinity approach, which had been designed to maximize the capture and identification of candidate JMJD6 substrates, to one which might be predicted to reveal their hydroxylation more completely. Thus, cells were treated with a lower dose of DMOG (0.5 mM) and the preincubation time was reduced from 9 h to 1 h in order to limit the inhibitory effect of the compound. These experiments also employed the lysine derivatization method, which had not been applied in the first interaction screen in order to maximize discovery by reducing the complexity of searches aimed at simple protein identification. Validating this approach, we detected a polyhydroxylated peptide derived from the BID of BRD4 that had previously been assigned in JQ1 pulldowns (*SI Appendix*, Fig. S10, for BRD4 peptide MSMS assignment). Further analyses revealed extensive lysine hydroxylation on peptides derived from many other JMJD6 coprecipitating proteins, including 12 targets that showed DMOG-inducible binding to JMJD6 in the earlier interaction experiment (Fig. 3*B*, *Inset*). In total, 108 hydroxylation sites were assigned to 32 different JMJD6-interacting proteins; many of the sites of hydroxylation were in lysine-rich sequences (Fig. 4*D*, JMJD6 protein pulldown). A representative MSMS corresponding to a lysine-rich peptide in NKAP (NF-kappa-B-activating protein) that contains five hydroxylysine residues is illustrated in Fig. 4*A* (for all other MSMS assignments see *SI Appendix*, Fig. S14).

Trapping of JMJD6-associated proteins that are polyhydroxylated on lysine argued that these proteins are all JMJD6 substrates; however, this was not proven directly. A proteomic screen that directly compared the hydroxylation status in wild-type and JMJD6-inactivated HeLa cells was therefore designed using a biotinylated peptide corresponding to residues 81 to 96 of JMJD6 as an affinity reagent. This alpha-helical peptide has been shown to bind directly to an isolated domain of BRD4 in vitro (25), and we reasoned that it might be used to capture other JMJD6 substrates. In support of this, we detected known substrates LUC7L2 (Luc7-like 2 pre-mRNA splicing factor) (26) and SRSF11 (24) at high abundance in pilot LC-MSMS data following JMJD6(81 to 96) peptide pulldown. Accordingly, JMJD6(81 to 96) peptide-coated streptavidin beads were incubated with lysate derived from wild-type or JMJD6-defective HeLa cells. Captured proteins were eluted, then propionylated prior to trypsinolysis and extensive LC-MSMS analysis. This defined 39 hydroxylysine sites in 26 different proteins; sites displaying >1% stoichiometry are summarized in Fig. 4*B*. Notably, these hydroxylations were only detected on peptides derived from cells expressing JMJD6, indicating that many, if not all, are bona fide sites of JMJD6-catalyzed hydroxylation. As an example, representative MSMS assigning hydroxylation to residues 1043 and 1044 of the FACT (facilitates chromatin transcription) complex subunit protein SPT16 are provided in Fig. 4 *C*, which compares spectra from wild-type cells (*Upper*; doubly hydroxylated) and JMJD6-inactivated (*Lower*; unmodified) as a mirror plot. In support of the assignment as sites of JMJD6-catayzed hydroxylation, half of the sites defined in this experiment (21 of 39) were also identified in FLAG-JMJD6 immunoprecipitates (Fig. 4*D*).

Combining the different experimental approaches, a total of 150 sites of lysyl hydroxylation were defined in 48 proteins. The results are summarized in Fig. 4*D* (see *SI Appendix*, Table S2 for reference to supporting MSMS) and reveal a strong enrichment of proteins bearing lysine-rich domains that were predicted to be unstructured and often the target site of hydroxylation (Fig. 4*E*). Because of the increased complexity of database searching following derivatization and the intrinsic difficulty of mapping low-complexity lysine-rich peptides, this likely represents an underestimate of the extent of JMJD6-catalyzed hydroxylation. We therefore also utilized a de novo peptide sequencing algorithm to directly compare high-quality fragmentation spectra derived from either wild-type or JMJD6-inactivated cells, independent of database matching. This confirmed the existence of multiple JMJD6-dependent hydroxylation sites in many but not all such regions across the JQ1 and JMJD6(81 to 96) peptide pulldown datasets (*SI Appendix*, Fig. S11).

## Discussion

JMJD6 has been implicated in a diverse range of cellular processes, including transcription elongation, mRNA splicing, DNA repair, angiogenesis, viral infection, stem cell behavior, and tumor growth (9, 11, 26–30). However, the molecular understanding of its actions has been held back by difficulties in directly defining its catalytic activity on endogenous proteins.

Noting that many JMJD6-associated proteins contain lysine-rich regions that are largely refractory to mass spectrometry of tryptic peptides, we applied orthogonal methods of affinity capture, protease digestion, and derivatization to identify JMJD6-catalyzed protein modifications at scale. Our analyses confirmed the original report of JMJD6-catalyzed lysine hydroxylation on the splicing factor U2AF65 (26) and defined more than 100 sites of lysine hydroxylation on numerous additional JMJD6-associated proteins. At many sites, the level of lysine hydroxylation was much higher than that at any previously reported site of JMJD6-catalyzed lysine hydroxylation and several of the proteins were very extensively hydroxylated, with 19 sites being defined on the bromodomain protein, BRD4. Our findings therefore, reveal very much more extensive JMJD6-catalyzed lysine hydroxylation than has previously been considered.

Recent work has revealed that in addition to the established lysyl hydroxylation (5*R*) of collagen by PLOD family members (PLOD1 to 3), several other 2-oxogluarate–dependent oxygenases hydroxylate lysyl residues, though the modifications are biochemically distinct. Thus, JMJD4 and JMJD7 catalyze hydroxylation at the C4 and C3 positions of lysyl side chains, respectively, while JMJD6 catalyses lysyl hydroxylation with 5*S* stereochemistry (1).

One question raised by the finding of multiple JMJD6 substrates is that of the determinants of JMJD6-catalyzed lysine hydroxylation. The protein substrates that we identified were strikingly enriched for unstructured lysine-rich regions, which were often the site of hydroxylation. Nevertheless, the level of hydroxylation at specific residues within these predicted disordered regions was distinct and near identical in material derived from different cells cultured under similar conditions, presumably indicating that specific structural features are induced by binding to JMJD6 or other molecules. Interestingly, these regions were enriched for both positively and negatively charged residues (*SI Appendix*, Fig. S12 and Table S3 for list of all sequence contexts). We also noted a difference in the binding characteristics of JMJD6 to different types of substrate. Although some substrates, particularly those bearing multiple hydroxylations in lysine-rich domains, showed marked enhancement of binding to JMJD6 when catalysis was inhibited with DMOG, this was not observed for other proteins, despite clear evidence for JMJD6-catalyzed hydroxylation. Such differences would support the existence of more than one type of substrate binding interaction, with some interactions being distinct from the catalytic site, as has been reported for BRD4 (25).

Another question relates to other proposed catalytic activities of JMJD6, particularly the arginine demethylation activity that has been reported on histones H3 and H4 (31). Although our affinity enrichment methods were not specifically designed to test this activity, we did not observe significant differential methylation among sites of arginine mono- and dimethylation that could be identified in the JMJD6-peptide and JQ1 affinity enrichment experiments performed on JMJD6 wild-type and inactivated cells (*SI Appendix*, Fig. S13). Nevertheless, we did not directly assess reported sites of arginine demethylation. Our data therefore neither confirm nor refute these additional catalytic activities of JMJD6.

In contrast, many sites of lysine hydroxylation coincided with proteins that have been associated with JMJD6, though not previously recognized to be modified in this way. For instance, it has been reported that JMJD6 and BRD4 cooperate to positively regulate RNA polymerase pause release at a set of genes that cobind these molecules at antipause enhancers (11). In this work, it was proposed that two separate demethylase activities of JMJD6 contribute to the release of promoter-paused RNA polymerase 2, demethylation of the repressive mark H4R3me2(sym), and demethylation of the cap on 7SK snRNA. These activities were proposed to remove or sequestrate the inhibitory 7SK snRNA/HEXIM1 complex enabling BRD4-dependent activation of the pause release complex, positive-transcriptional elongation factor (P-TEFb). Interestingly, in addition to BRD4 being the most heavily hydroxylated protein identified in the current work, JMJD6-catalyzed lysine hydroxylation was identified on several other proteins that are involved in BRD4 or P-TEFb–mediated effects on transcription. The 7SK snRNA binding protein, La-related protein 7, which stabilizes the 7SK snRNP complex and DKC1 that catalyzes stabilizing pseudouridylation of 7SK snRNA, were both hydroxylated within lysine-rich sequences by JMJD6 (32, 33). Thus, our work defines multiple catalytic actions of JMJD6 among protein complexes implicated in transcriptional control.

We also identified many sites of JMJD6-catalyzed lysine hydroxylation on multiple SR (serine and arginine) proteins, including several LUC7L pre-mRNA splicing factors, SRSF11, RBM39 (RNA binding motif protein 39), and U2SURP (U2 SnRNP-associated SURP domain containing). These RNA binding proteins are increasingly recognized to function at different stages in the regulation of gene expression encompassing transcriptional elongation, as well as transcription-coupled RNA splicing and other steps in RNA processing and metabolism (34). Several SR proteins have been identified in protein complexes with JMJD6, but catalytic modifications were not identified or only identified using recombinant JMJD6 and substrates (24, 35). Our analyses revealed multiple hydroxylations on endogenous SR proteins, many of which are high-level or near complete modifications. JMJD6-catalyzed lysine hydroxylation was also observed on many other proteins associated with the nucleolus, consistent with JMJD6 also being observed in the nucleolus in certain circumstances (36). Interestingly, other recent work has defined a direct role for JMJD6 in the repair of damage to ribosomal DNA. In this process, JMJD6 is reported to interact with the ribosome biogenesis factor, Treacle (encoded by *TCOF1*) to regulate its interaction with Nibrin (or NBS1), a component of the MRN (MRE11-RAD50-NBN) DNA double strand break recognition and signaling complex (29). It was reported that an undefined catalytic action of JMJD6 is required for efficient relocalization of ribosomal DNA to nucleolar caps and repair. Our analysis defines seven sites of JMJD6-catalyzed lysine hydroxylation in the Treacle protein. Overall, the work reveals multiple catalytic actions of JMJD6 on proteins involved at many stages in pathways linking transcription, RNA processing, and translation, suggesting that the functional interface of JMJD6 with these processes may be highly complex.

Based on the very extensive lysine hydroxylation and DMOG-induced alterations in substrate association with JMJD6, it might be predicted that condition-dependent alteration in JMJD6 catalytic activity would create multiple reassortments of protein interactions in JMJD6-associated complexes. A related and interesting possibility is that JMJD6 catalysis of lysine hydroxylation regulates higher order protein associations through effects on liquid–liquid phase separation and the actions of this process on membraneless organelles or cellular condensates. Liquid–liquid phase separation is driven by multivalent interactions between nucleic acids and amino acid residues in intrinsically disordered polypeptide regions (for review see ref. 37). This property has been demonstrated for polylysine sequences both in vitro and in vivo (38). Moreover, many of the polyhydroxylated lysine-rich regions defined in the current study have been directly implicated in this process, including those of BRD4 in transcriptional condensates (39), NKAP, and USP42 in nuclear speckles (40, 41), and DKC1 in nucleolar localization (42).

Very recently, involvement of JMJD6 in nucleolar phase separation was demonstrated for Liat1 (Ligand of ATE1), a protein that binds the arginyl–tRNA protein transferase ATE1 (43). A polylysine region in Liat1 was shown to be important in targeting the protein to the nucleolus. JMJD6 activity was shown to inhibit this nucleolar localization by comparison of the effects of wild-type and catalytically inactive mutant JMJD6, but a catalytic modification of Liat1 was not reported. Though we did not identify Liat1 in our affinity capture experiments, its sequence is very similar to the multiply hydroxylated polylysine domains that we did identify. Overall, a remarkable 42 of the 48 JMJD6 substrates that we identified have been associated with one or more types of membraneless organelle or cellular condensate (Fig. 4*D*). Given its dependence on molecular oxygen and potential susceptibility to metabolic and oxidant stresses, an intriguing possibility is that JMJD6 catalytic activity might alter the formation or composition of these cellular condensates in response to cellular stresses. Our findings, together with development of new mass spectrometry approaches to the analyses of lysine hydroxylation, should enable these questions to be addressed in future work.

## Materials and Methods

### Cell Culture: Cell Lines, Treatments, Transductions, and Gene Editing.

Cell lines (HeLa, SH-SY5Y, mouse embryonic fibroblasts) were maintained in Dulbecco's modified Eagle's medium supplemented with 10% fetal bovine serum, 2 mM L-glutamine, and where appropriate, antibiotic selection (2 μg/mL puromycin). Cell line authentication (short tandem repeat profiling) and mycoplasma screening was provided by Cell Services at the Francis Crick Institute. Hypoxic exposure of cells was performed in an atmosphere of 0.1% or 1% oxygen, 5% carbon dioxide, and balance nitrogen for 24 h (InvivO2 400; Baker-Ruskinn); pharmacological inhibition of JMJD6 activity used 0.5 or 1 mM dimethyloxalylglycine (Merck) for the indicated duration.

The JMJD6 gene was edited using the Cas9^D10A^ nickase variant, which was developed to minimize off-target effects. Guide sequences complementary to regions in exon 2 were selected by in silico prediction (https://zlab.bio/guide-design-resources) and inserted into plasmids bearing the sgRNA backbone and either a GFP reporter (PX461; sequence: AACAAAACCACGGGCTTGTAAGG) or the puromycin resistance gene (PX462; sequence: GCAAGAGGGCTGGTCTGCGCAGG). These plasmids were cotransfected into HeLa cells. JMJD6-defective clones were identified by immunoblotting and genomic DNA sequencing, following GFP enrichment. CRISPR/Cas9-induced loss-of-function mutations were identified in a clone that was used to create both the doxycycline-inducible (pLIX402 JMJD6) and constitutive (pRRL FLAG-JMJD6) rescue cell lines by lentiviral transduction of sequences encoding, transcript variant 2 (National Center for Biotechnology Information reference sequence: NM_015167.3), the canonical form (isoform 1) of JMJD6 protein.

### Peptide Synthesis and Biotin Conjugation to JQ1.

The biotinylated JMJD6(81-96) peptide was synthesized at the Peptide Chemistry Facility, Francis Crick Institute. In brief, solid-phase peptide synthesis was performed on an automated peptide synthesizer (Intavis Multipep; CEM Corporation) using a Rink Amide AM resin LL (0.05 mmol; Merck) and N(α)-Fmoc amino acids, including Fmoc-6-aminohexanoic acid, which served as a linker for the biotin group. The peptide was biotinylated on the synthesizer using d-biotin dissolved in 1:1 dimethyl sulfoxide (DMSO):*N*-methyl-2-pyrrolidone. Peptides were cleaved from the resin, precipitated by addition of diethyl ether, and purified by high pressure liquid chromatography.

Conjugation of biotin-PEG11-amine (Thermo Scientific) to JQ1 (Focus Biomolecules) was performed using established methods (44).

### Affinity Purification and Immunoblotting.

Cells were lysed in 20 mM Tris (pH 7.4), 100 mM NaCl, 0.5% Igepal CA630, and 5 μM MgCl_2_ supplemented with protease inhibitor mixture (Thermo Fisher) and DNA/RNA endonuclease (Benzonase 125 units/mL; Merck) for affinity purification. An indirect immunoprecipitation method was employed for affinity pulldowns, i.e., saturating amounts of antibody/probe were bound to magnetic beads prior to incubation in cell lysate. Preparative scale pulldowns typically used 5 mg of cell lysate and 1) 700 μg of anti-rabbit IgG magnetic beads (prebound to anti-BRD2,3,4 polyclonal antibodies; Bethyl); 2) 40 μL of FLAG M2 affinity beads (Merck); 3) 50 μg of streptavidin beads coated in biotinylated JQ1; and 4) 600 μg of streptavidin beads immobilized with biotinylated JMJD6(81 to 96) peptide. Lysate/bead incubations were performed overnight with constant rotation at 4 °C. Beads were washed in lysis buffer and proteins were eluted by addition of 0.5 M ammonium hydroxide, pH 11, and 0.5 mM ethylenediaminetetraacetic acid. Samples were resolved by sodium dodecyl sulfate–polyacrylamide gel electrophoresis and standard immunoblotting procedures were followed for the detection of specific proteins (anti-DKC1, Bethyl; anti AP3D1, Santa Cruz Biotechnology; anti-DDX41, Cell Signaling Technology) by chemiluminescence.

### In Vitro Hydroxylation Assays.

Recombinant human JMJD6 (full length with a N-terminal His6 tag; in pET-28b plasmid) was produced in *Escherichia coli* BL21 (DE3) cells. Induced cultures (isopropyl β-d-1-thiogalactopyranoside 0.5 mM, 4 h at 16 °C) were lysed in: 50 mM Tris⋅HCl pH 8.0, 200 mM NaCl, 20 mM imidazole, 0.5 mM Tris (2-carboxyethyl)phosphine (TCEP), 5% (vol/vol) glycerol, supplemented with 1× protease inhibitor mixture (C0mplete; Roche) and nuclease (benzonase 25 units/mL; Merck), and subject to high pressure homogenization (EmulsiFlex-C5; Avestin). JMJD6 was purified by standard nickel-affinity and size exclusion (Superdex 200) chromatography methods. In vitro hydroxylation assays employed 2 μM JMJD6, 20 μM BRD4 peptide, 10 μM Fe(II), 200 μM 2-oxoglutarate, 100 μM ascorbate, and, where appropriate, 100 μM of 2,4-pyridinedicarboxylic acid. The assay mixtures were incubated at room temperature for up to 2 h prior to addition of 10% (vol/vol) formic acid (to stop hydroxylation) and analysis of intact peptide mass on an Agilent 6550 iFunnel Q-TOF LC/MS system.

### Sample Preparation for Mass Spectrometry.

Lysine derivatization with propionic anhydride (Merck) was performed by combining standard in-gel trypsinolysis workflows and histone derivatization methods (45). In brief, acrylamide gel pieces were destained by addition of 50% methanol/5% acetic acid solution and either reduced and alkylated by addition of 10 mM dithiothreitol and 50 mM iodocaetamide or derivatized without prior reduction/alkylation [FLAG-JMJD6 and JMJD6(81 to 96) peptide experiments]. Gel pieces were dehydrated by addition of 100% acetonitrile (MeCN) prior to two rounds of propionylation, which was performed in small batches to enable precise timing of the reaction. Gel pieces were rehydrated by addition of four volumes of 100 mM ammonium bicarbonate directly followed by one volume of propionylation reagent (propionic anhydride and anhydrous MeCN in a 1:3 vol/vol ratio). Samples were mixed and the pH was immediately adjusted to pH 8 by addition of ammonium hydroxide. The reaction was incubated at 51 °C for 20 min with constant mixing. A second round of derivatization was performed prior to two exchanges (dehydration and rehydration) in 100% MeCN and 100 mM ammonium bicarbonate to remove residual propionylation reagent prior to in-gel digestion with trypsin. For the JMJD6 interactome experiments, FLAG-affinity eluates were precipitated by methanol/chloroform and resuspended in urea buffer (6 M urea, 100 mM Tris pH 7.8) prior to reduction/alkylation and trypsinolysis, which was performed in solution under denaturing (1 M urea) conditions. Where appropriate, peptide preparations were desalted by solid-phase extraction (Oasis HLB sorbent; Waters Corporation) in accordance with the manufacturer’s instructions, and resuspended in aqueous 2% (vol/vol) MeCN, 0.1% (vol/vol) formic acid for mass spectrometric analysis.

### Mass Spectrometry.

Data-dependent acquisitions (DDAs) were typically acquired on a nano ultra performance liquid chromatography system (UltiMate 3000 RSLCnano; Thermo Scientific) coupled to an Orbitrap Fusion Lumos Tribrid mass spectrometer (Thermo Scientific). Reverse phase separation was performed at a flow rate of 250 nL/min on an EASY-spray PepMap rapid separation liquid chromatography C18 column (75 μm × 500 mm, 2 μm particle size; Thermo Scientific) over a 1-h gradient of 2 to 40% MeCN in 5% DMSO/0.1% formic acid. MS1 spectra were typically acquired with a resolution of 120 k and an automatic gain control (AGC) target of 4e5. MSMS spectra were acquired at a resolution of 30 k for up to 54 ms and an AGC target of 5e4 with a cycle time of 3 s and a normalized collision energy of 32% in the higher-energy C-trap dissociation (HCD) cell. Selected precursors were isolated in the quadrupole with an isolation window of 1.2 Da and excluded for 60 s for repeated selection.

Data-independent acquisitions (DIAs) were acquired on an Evosep One HPLC system coupled to an Orbitrap Fusion Lumos. Reverse phase separation was performed at a flow rate of 500 nL/min on an EV1106 analytical column (150 μm × 15 cm, 1.9 μm particle size; Evosep) using the vendor’s predefined 44-min gradient method. Lumos instrument settings were as follows: MS1 data acquired in the Orbitrap with a resolution of 120 k, max injection time of 20 ms, AGC target of 1e6, in positive ion mode, in profile mode, over the mass range of 393 to 907 *m/z*. DIA segments over this mass range (20 *m/z* wide/1 Da overlap/27 in total) were acquired in the Orbitrap following fragmentation in the HCD cell (32%), with 30 k resolution over the mass range 200 to 2,000 *m/z* and with a max injection time of 54 ms and AGC target of 1e6.

### Mass Spectrometry Data Processing and Analysis Pipeline.

Associated methods describing the data processing and analysis pipelines can be found in *SI Appendix*, *Supplementary Materials and Methods*.

## Supplementary Material

Supplementary File

## Data Availability

Detailed instrument settings for DIA and DDA runs are documented in the MS data (RAW) files, which have been deposited to the ProteomeXchange Consortium via the PRIDE partner repository with the dataset identifiers PXD031155 (48) and PXD031221 (49) respectively.
