# Supplementary material for: Widespread hydroxylation of unstructured lysine-rich protein domains by JMJD6
Source: Proc Natl Acad Sci U S A. 2022 Aug 5;119(32):e2201483119. doi: 10.1073/pnas.2201483119 (PMC9371714; doi:10.1073/pnas.2201483119)
Supplement: Supplementary File [file pnas.2201483119.sapp.pdf]

PNAS
www.pnas.org

## Supplementary Information for

# Widespread hydroxylation of unstructured lysine-rich protein domains by JMJD6

Matthew E. Cockman[1]*, Yoichiro Sugimoto[1], Hamish B. Pegg[1], Norma Masson[2], Eidarus Salah[3], Anthony Tumber[3], Helen R. Flynn[1], Joanna M. Kirkpatrick[1], Christopher J. Schofield[3] and Peter J. Ratcliffe[1,2]*

[1]The Francis Crick Institute, London, U.K.

[2]Ludwig Institute for Cancer Research, Nuffield Department of Clinical Medicine, University of Oxford, Oxford, U.K.

[3]Chemistry Research Laboratory, Department of Chemistry, University of Oxford, Oxford, UK.

*Matthew E. Cockman and Peter J. Ratcliffe

**Email:** matthew.cockman@crick.ac.uk; peter.ratcliffe@crick.ac.uk

**This PDF file includes:**

Supplementary text
SI References
Tables S1 to S3
Figures S1 to S14

## Supplementary Materials and Methods

### Overview of data analysis

DDA or DIA MS data were first analysed by PEAKS Studio (Bioinformatics Solutions) or Spectronaut (Biognosys) software, respectively (details are described below). Subsequent analyses were performed using R (4.0.0)[1] with the following packages (data.table (1.12.8)[2], dplyr (1.0.0)[3], stringr (1.4.0)[4], magrittr (1.5)[5], and Biostrings (2.56.0)[6]); The computational analysis pipeline used for this study is available from (https://github.com/YoichiroSugimoto/20211110_JMJD6_K_hydroxylation). The data were visualised using either Microsoft Excel, Graphpad Prism (9.3), pheatmap (1.0.12)[7], ComplexHeatmap (2.4.3)[8] and ggplot2 (3.3.1)[9].

Initial analyses of BRD4 hydroxylation in publicly available data were performed on deep proteome datasets (PXD002395, PXD003977, PXD004452) downloaded from the PRIDE repository and processed with PEAKS Studio (v10). Processing parameters were as follows: Precursor ion tolerance was 10 ppm; Fragment ion tolerance was 0.5 Da for Ion Trap acquisitions and 0.02 Da for Orbitrap; trypsin cleavage rules were specific with a maximum of 2 missed cleavages; Carbamidomethylation (C) was selected as a fixed modification and variable modifications (up to 3 permitted) were deamidation (N,Q), acetylation of protein *N*-termini, oxidation (C, D, F, H, K, M, N, P, R, W, Y) and dioxidation (F, M, W, Y). Data were matched against the canonical human Uniprot database proteome (retrieved 24th September 2019).

Quantitation of fragment ions from PRM acquisitions were performed by manual extraction and area integration in Qualbrowser (Xcalibur v4.1; Thermo Scientific).

### Database analyses of DDA data

Processing of "in-house" DDA data used PEAKS Studio (v10.5 and 10.6). The following parameters were applied to non-derivatised tryptic data acquired by Higher-energy C-trap dissociation (HCD): 15 ppm mass error tolerance for precursor ions and 0.02 Da tolerance for fragment ion masses; trypsin cleavage rules were specific with a maximum of 2 missed cleavages; carbamidomethylation (C) was selected as a fixed modification and variable modifications (up to 3 permitted) were: deamidation (N,Q), acetylation of protein *N*-termini, oxidation (M,W,K) and dioxidation (M,W). Data were matched against the appropriate species-specific (human/mouse) canonical Uniprot database (retrieved 8th February 2021) with a list of common contaminants appended (cRAP database; http://crapome.org). Peptide false discovery rate (FDR) was set to 1%

using a target-decoy method and PTM site determination was informed by a probability-based ambiguity score (Ascore; -10lgP). The number of variable modifications was increased to 5 for Asp-N digestion (cleavage before D, E) in order to monitor hydroxylation of lysine-rich sequences. To analyse data from propionylated samples, the DDA workflow was adapted for processing incompletely derivatised tryptic peptides. It was not possible to modify the *in-silico* specificity of trypsin toward propionylated lysine within the PEAKS DB search engine. Thus, a large number (n=5) number of missed cleavage occurrences was specified in order to detect derivatised lysine-rich polypeptides. A maximum number of 5 variable modifications were considered, specifically: deamidation (N,Q), acetylation of protein *N*-termini, oxidation (M,W,K), propionylation (K) and oxidised propionylation (K); note, carbamidomethylation (C) was considered a fixed modification only in preparations that were treated with iodoacetamide. To reduce prohibitively long search times, precursor mass error tolerance was reduced from 15 ppm to 7.5 ppm and, where appropriate, custom databases curated from DDA searches of non-derivatised experiments were specifically interrogated (e.g., JQ1 PD and FLAG-JMJD6 interactome PD). Peptides meeting the significance filter (-10lgP >15) and MS2 ion intensity filter (>1%) were exported as "protein-peptides.csv" files.

For the calculation of site-specific PTM stoichiometry, analyses were restricted to JQ1 and JMJD6 peptide pulldown datasets to enable direct comparison between wild-type and JMJD6-defective HeLa cells. PEAKS export files were filtered to remove peptides that were not uniquely assigned to a protein and peptides with an AUC (area under the curve, LC-MS/MS data) of 0. Peptides that were unlikely to be products of trypsinolysis were also filtered out. For this purpose, we assumed that trypsin cleaves *C*-terminal to lysine, hydroxylysine, arginine, and mono-methyl arginine, but not propionyl lysine and di-methyl arginine. Thus, peptides with a modified *C*-terminal amino acid incompatible with cleavage by trypsin were excluded. In addition, peptides that had more than one "genuine" tryptic cleavage site were filtered out; we did not consider lysine or arginine followed by a proline a tryptic cleavage site. We only considered PTMs that passed the PEAKS MS2 ion intensity filter (>1%). Based on the filtered data, the stoichiometry of a PTM at a specific residue of a protein was calculated using the following formula:

$$\frac{\sum_{\substack{\text{all the peptides with} \\ \text{the PTM at the residue}}} AUC}{\sum_{\substack{\text{all the peptides without} \\ \text{the PTM at the residue}}} AUC + \sum_{\substack{\text{all the peptides with} \\ \text{the PTM at the residue}}} AUC}$$

Note, these measurements are relative values, which are not corrected for any effect of propionylation and/or hydroxylation on ionisation efficiency. Filtered data comparing peptide identifications between wild-type and JMJD6-defective preparations were used to shortlist JMJD6-dependent hydroxylations that were verified by manual inspection of MSMS spectra.

### ***De novo* sequencing**

*De novo* sequencing results, which assist the PEAKS DB search algorithm, were also analysed to identify hydroxylysines in peptides irrespective of protein assignment. The sequencing results were exported from PEAKS studio as a "de novo peptides".csv file and pre-processed as follows: (1) tryptic peptides that did not have a *C*-terminus amino acid compatible with cleavage by trypsin, and peptides which had more than one "genuine" missed trypsin cleavage site were filtered out, as described above; (2) peptides with low sequence quality (average local confidence score < 50%) were filtered out. The local confidence score is reported by the PEAKS Studio software, and indicates the likelihood of the correct sequence assignment at a specific residue within a peptide. For heatmap visualization of hydroxylysine assignments (Fig. S11) peptides were aligned so that the hydroxylysine residues were clustered around the centre of the display (i.e. position 0). To this end, the sum of the local confidence scores for hydroxylysine residues within a 6-residue sliding window along the peptide was calculated. The position of the sliding window (4th residue from the start of the window) giving the highest aggregated score was defined as the position 0 of the peptide.

### **DIA data**

DIA data corresponding to the FLAG-JMJD6 interactome were processed using Spectronaut (v14; Biognosys). DIA data were searched against a spectral library that was created by combining DIA plus DDA search results (DpD search) of FLAG-JMJD6 pulldowns obtained with the Spectronaut Pulsar search engine. DpD data were searched against the human proteome (Canonical Uniprot; downloaded August 2019) with the following modifications considered: fixed, carbamidomethyl (C); variable, oxidation (M), acetyl (N-terminal protein) and a maximum of 5 variable modifications and 2 missed cleavages permitted. A 1% FDR filter was applied at the peptide and protein level to yield a spectral library containing 14,996 precursors in 1517 protein groups determined by Spectronaut protein inference. DIA data were subsequently searched against the DpD library using default Spectronaut analysis settings, with the following modifications; (1) exclusion of

protein identities based on less than 2 peptides; (2) quantitation: median peptide and precursor quantity, MS2 level area counts; (3) data filtering: Q-value percentile (fraction 0.16); (4) run-wise imputing strategy; (5) cross-run normalisation: no filter, local normalisation strategy and automatic selection. Protein level quantitation data were exported from Spectronaut as a pivot report for statistical analysis in R.

**Data processing for heatmap visualisation**

The display of proteins interacting with FLAG-tagged transgenes encoding: wild-type JMJD6, a catalytically-inactive form, or empty vector control, which are represented in Figure 3B, comprises all proteins manifesting: (i) differential interaction with wild-type JMJD6 versus empty vector or enzyme-dead JMJD6 or (ii) differential interaction with wild-type JMJD6 in the presence or absence of DMOG, as identified using an empirical Bayes moderated *t*-statistic on log2 transformed protein abundance data with the limma package (3.44.1)[10]. The heatmap illustrates the Z-score of log2 transformed abundance data for each protein relative to all replicates and experimental conditions. Proteins were ordered according to the results of hierarchical clustering using Spearman's rank correlation for the calculation of distance and applied Ward's algorithm for clustering.

**Analysis of sequence and biophysical features of proteins**

Protein disorder was predicted using IUPred2A software[11] with default parameters. The local charge in a protein was calculated using the idpr package (1.2.0)[12] by considering the sequence from -5 to +5 residues. K-score was calculated as the maximum number of lysine residues in a 10-residue sliding window along a protein sequence. Stoichiometry was only calculated for sites with more than two assigned peptide features (Figure 4E; bubble plot).

**Supplemental References**


1. R Core Team. R: A Language and Environment for Statistical Computing. (2020).
2. Dowle, M. & Srinivasan, A. data.table: Extension of `data.frame`. (2019). R Package Version 1.12.8.
3. Wickham, H., François, R., Henry, L. & Müller, K. dplyr: A Grammar of Data Manipulation. (2020). R Package Version 1.0.0.
4. Wickham, H. stringr: Simple, Consistent Wrappers for Common String Operations. (2019). R Package Version 1.4.0.
5. Bache, S. M. & Wickham, H. magrittr: A Forward-Pipe Operator for R. (2014). R Package Version 1.5.
6. Pagès, H., Aboyoun, P., Gentleman, R. & DebRoy, S. Biostrings: Efficient manipulation of biological strings. (2020). R Package Version 2.56.0.
7. Kolde, R. pheatmap: Pretty Heatmaps. (2019). R Package Version 1.0.12.
8. Gu, Z., Eils, R. & Schlesner, M. Complex heatmaps reveal patterns and correlations in multidimensional genomic data. Bioinformatics 32, 2847–2849 (2016). R Package Version 2.4.3.
9. Wickham, H. ggplot2: elegant graphics for data analysis. (2009). R Package Version 3.3.1.
10. Ritchie, M. E. et al. Limma powers differential expression analyses for RNA-sequencing and microarray studies. Nucleic Acids Research 43, e47 (2015).
11. Mészáros, B., Erdös, G. & Dosztányi, Z. IUPred2A: Context-dependent prediction of protein disorder as a function of redox state and protein binding. Nucleic Acids Research 46, W329–W337 (2018).
12. McFadden, W. M. & Yanowitz, J. L. idpr: Profiling and Analyzing Intrinsically Disordered Proteins in R. (2021). R Package Version 1.2.0.

**Supplementary Data Legends**

**Table S1: Summary of hydroxylation assignments on BRD4(520-537) from analysis of large-scale proteomic datasets.** Counts relating to the oxidation site assignment of singly- (Δ mass: +15.99) and doubly-oxidised (Δ mass: +31.99) forms of the tryptic BRD4(520-537) peptide in publicly deposited proteome datasets using PEAKS software. The numbers of assigned peptide spectra (FDR 1%) were counted using stringency filters for PTM assignment confidence (AScore: >0) and fragment ion intensity (>5%), applied separately and in combination. Most of the singly-oxidised peptides failed to pass the combined stringency filter. In contrast, secure assignments were seen on doubly-oxidised peptides with all such assignments (i.e., passing both Ascore and Ion Intensity filters) supporting hydroxylation of the lysyl (K535 and K537) residues.

**Table S2: Summary of all hydroxylation assignments from this study.** List of all hydroxylation sites defined in this study; sites are presented in alphabetical order referenced to gene name, Uniprot accession, site of modification and representative MSMS assignment (Figure S14). † Denotes non-unique peptide shared between proteins that were expressed at similar levels.

**Table S3: Summary of hydroxylation site sequence context.** Reference table detailing the sequence context of all assigned hydroxylation sites. Sites are presented in alphabetical order referenced to gene name, Uniprot accession and site of modification. Site context is defined as residues -5 to +5 of the targeted lysine residue, which is indicated in bold font. † Denotes hydroxylation assignment from non-unique tryptic peptide shared between indicated proteins that were expressed at similar levels.

**Figure S1: Triple Knockout Mouse Embryonic Fibroblasts are defective for HIF hydroxylase activity.** Validation of PHD1,2,3 defective (TKO) MEFs used in Fig 1A. Wild type and TKO MEFs were treated with proteasomal inhibitor (MG132, 12.5 µM for 4 hours) in the presence or absence of the hydroxylase inhibitor DMOG (1 mM for 24 h). Hydroxylated HIF1α was detected by immunoblot (anti-Hyp564) in lysates derived from proteasomally-blocked wild type but not TKO MEFs.

**Figure S2: MSMS fragmentation spectra and ion tables for BRD4 peptides depicted in Figure 1A.** HCD fragmentation spectra were acquired in the orbitrap of: **(a)** unoxidised; **(b)** singly-oxidised; **(c)** doubly-oxidised forms of the tryptic BRD4(521-538) peptide. Figure header indicates: peptide sequence ([O] site of hydroxylation) and MS-related information including peptide significance score (-10 lgP value) and MSMS scan number with raw data file reference, provided in parentheses. Ion tables give the theoretical mass of each fragment ion; assigned fragment ions (within the defined mass tolerance of 0.02 Da) are displayed in colour; b-ions in blue, y-ions in red.

**Figure S3: MSMS fragmentation spectra and ion tables for BRD4 peptides depicted in Figure 1B.** HCD fragmentation spectra acquired in linear ion trap of: **(a)** unoxidised; **(b)** singly-oxidised; **(c)** doubly-oxidised forms of the tryptic BRD4(520-537) peptide; [O] site of hydroxylation. Additional peptide information is provided for each MSMS, including peptide significance score (-10 lgP value) and MSMS scan number with raw data file reference. Ion tables give the theoretical mass of each fragment ion; assigned fragment ions (within the defined mass tolerance of 0.5 Da) are displayed in colour; b-ions in blue, y-ions in red.

**Figure S4: Assignment of K535 and K537 hydroxylation by parallel reaction monitoring (PRM).** **(A)** DDA-derived XIC depicting precursor ion (MS1) intensities of m/z 663.0288, m/z 668.3605, and m/z 673.6921 corresponding to unoxidised (yellow-green), singly-oxidised (orange), and doubly-oxidised (red) forms of BRD4(520-537) derived from SH-SY5Y cells by LC-MSMS. **(B)** High resolution MS2 spectra were continually acquired from isolated BRD4(520-537) precursor ions (upper panels, i-iii for different hydroxylated forms) using the PRM methodology. Chromatograms

of fragment ions, including ions of discriminatory mass for lysine and proline oxidation (see inset for schematic representation of fragment ions: dotted line, no mass adduct; dashed line, single oxidation; solid line, double oxidation) were extracted at 5 ppm mass tolerance and presented below the indicated precursor ion. The presence of m/z 163.1077 (dashed red line; K[O]) and absence of m/z 147.1128 (dotted red line; K) in the MS2 XIC for the singly-oxidised peptide (middle panel, ii), reliably assign K537ox and exclude P536ox, at least to the limit of detection. For the doubly oxidised peptide, the only diagnostic fragment ions detected were: m/z 163.1077 (dashed red line; K[O]); m/z 260.1605 (dashed yellow-green line; imputed from y1 as: PK[O]) and; m/z 404.2503 (solid green line; K[O]PK[O]).

**Figure S5: Hydroxylation of BRD2 and BRD3 by JMJD6 (A)** Extracted ion chromatograms (XIC) show peptide ion intensities corresponding to unoxidised (green-yellow) and singly-oxidised (turquoise) forms of the tryptic BRD2(529-546) ($[M+3H]^{3+}$) peptide isolated from HeLa cells. Hydroxylated BRD2(529-546) peptide ions are absent from JMJD6-defective HeLa cell preparations (right panel). **(B)** Extracted ion chromatograms (XIC) show peptide ion intensities corresponding to unoxidised (green-yellow) and singly-oxidised (pink) forms of the tryptic BRD3(472-489) ($[M+3H]^{3+}$) peptide isolated from HeLa cells. Hydroxylated BRD3(472-489) peptide ions are absent from JMJD6-defective HeLa cell preparations.

**Figure S6: Fragmentation spectra and ion tables for assignment of BRD4(523-541) peptides depicted in Figure 1C.** Exemplar orbitrap HCD fragmentation spectra corresponding to the different oxidation [O] states of the Asp-N proteolyzed BRD4(523-541) in SH-SY5Y cells: **(a)** unmodified; **(b)** 1[O]; **(c)** 2[O]; **(d)** 3[O]; **(e)** 4[O]; **(f)** 5[O]. Additional peptide information is provided for each MSMS, including peptide significance score (-10 lgP value) and scan number (in raw data file). Ion tables are provided for each MSMS spectra depicting the theoretical mass of each fragment ion. Assigned fragment ions (matching within the defined mass tolerance of 0.02 Da) are displayed in colour; *N*-terminal b-ions are blue, *C*-terminal y-ions are red.

**Figure S7: JMJD6-catalysed hydroxylation of BRD4 peptide substrate. (A)** A 40-residue synthetic peptide (20 µM) corresponding to residues 511-550 of BRD4 was incubated with recombinant

JMJD6 (2 μM) in the presence (i) or absence (ii) of an inhibitor, 2,4-pyridine-dicarboxylic acid (2,4-PDCA, 100 μM). LC-MS spectra showing efficient hydroxylation of BRD4 peptide; 3 successive +16 m/z shifts were observed following 60 min incubation with JMJD6 and co-factors (100 μM L-ascorbic acid, 10 μM ferrous ammonium sulphate, 200 μM 2-oxoglutarate). **(B)** MSMS assignment of K547ox in (Aii). A mass identical to a hydroxylysine-containing fragment ion is observed in the JMJD6-reacted peptide ($[M+9H]^{9+}$ = m/z 524.30) at the y4 position that assigns hydroxylation to Lysine-547.

**Figure S8: Comparison of quantitative assessments of hydroxylysine modification by orthogonal proteomic methods.** Site specific stoichiometry of hydroxylysine as assessed by (i) non-derivatised (Asp-N mediated proteolysis) and (ii) derivatised (propionic anhydride and trypsin) workflows was similar. BRD4 protein was immunopurified from HeLa cells.

**Figure S9: Comparison of JMJD6-dependent hydroxylation of BRD4 across multiple cell lines.** BRD4 was purified from HEK293, MCF-7 and HeLa cell lines, prior to derivatisation, trypsinolysis and LC-MSMS analysis. Site specific stoichiometry of hydroxylation was similar across these cells, but not detected in JMJD6-defective HeLa cell preparations (lower panel).

**Figure S10: Assignment of BRD4 poly-lysyl hydroxylation in FLAG-JMJD6 pulldown.** Fragmentation spectra of BRD4(547-553) obtained following FLAG-JMJD6 pulldown, derivatization with propionic anhydride and digestion with trypsin; * derivatised lysine, [O] site of hydroxylation. Additional peptide information is provided, including: peptide significance score (-10 lgP value) and scan number (in raw data file). An Ion table is provided showing the theoretical mass of each fragment ion; note, +72.02 Da corresponds to the mass of oxidised propionylated lysine. Assigned fragment ions (matching within the defined mass tolerance of 0.02 Da) are displayed in colour; *N*-terminal b-ions are blue, *C*-terminal y-ions are red.

**Figure S11: *De-novo* MSMS sequencing indicate poly-lysine hydroxylation is a JMJD6-dependent property.** Heatmap visualisation of lysine hydroxylation assigned by *de novo* sequencing of

derivatised tryptic peptides from wild-type (left panel) and JMJD6-defective (right panel) HeLa cells. Each row represents a single peptide; purple tone indicates the confidence of hydroxylysine assignment at the indicated position in the peptide. All other amino acid assignments are represented as white tiles, with transition to grey indicating the limits of the sequenced peptide. Peptides are aligned (x-axis) by the position of a 6-mer window giving the highest hydroxylysine score across the sequenced peptide so that hydroxylysines are clustered around the centre of the plot (i.e. position 0), and sorted (y-axis) by that score. Separate heatmaps give the results for peptides binned according to the maximum K-score of a 6-residue sliding window across the peptide. A much higher level of hydroxylysine is observed in wild-type cells in the bin representing the highest K-score (top left). In this bin, high hydroxylysine scores are observed at multiple positions, indicating multiple hydroxylations of adjacent lysine residues. Within this bin of lysine rich sequences, many (top, purple) but not all peptides (bottom, white) are multiply hydroxylated. In contrast, hydroxylysine is only modestly increased in JMJD6 wild-type cells in bins representing a K-score of 4/6 and not evident below this score.

**Figure S12: Analysis of sequence features surrounding sites of JMJD6-catalysed hydroxylation.** Heatmap depicts enrichment or depletion of amino acids residues (y-axis) at positions (x-axis) adjacent to JMJD6-catalysed hydroxylated lysine residues. All hydroxylysine sites defined in this study were analysed. Data are expressed as an odds ratio relative to all lysine residues in the total proteome identified in these experiments.

**Figure S13: Site-specific comparison of hydroxylysine and methylarginine PTM stoichiometry on derivatised tryptic peptides derived from wild-type and JMJD6-defective HeLa cells.** Points represent the difference in (delta) stoichiometry (WT minus JMJD6-defective (KO) cells) x100 for each specific site of post-translational modification assigned as hydroxylysine (Hyl), mono-methylarginine (mme-R) or di-methylarginine (dme-R) in JQ1 and JMJD6(81-96) peptide pulldown experiments. To improve the accuracy of the PTM assignments, database search results were filtered by (i) minimal significance score (-10lgP) of peptide >15; (ii) modification-specific fragment ion intensity > 1%; (iii) minimum of 2 independent MSMS assignments per cell line in each experiment; (iv) exclusion of hydroxylysine assignments that were likely confounded by

artefactual oxidation (i.e., within 2 residues of a methionine or tryptophan residue). Note, JMJD6-dependent modifications would manifest as positive delta values for hydroxylation and negative delta values for demethylation at methylarginine sites. A clear positive bias is observed for Hyl, whereas no such negative bias is observed for mme-R or dme-R; apparently randomly scattered positive and negative values likely represent noise, in part due to stochastic sampling of low abundance peptides in DDA mode.

**Figure S14: MSMS assignment of JMJD6-dependent hydroxylations.** Fragmentation spectra corresponding to sites of JMJD6-dependent lysyl hydroxylation defined in this study. Spectra are from protein pulldowns enriched by affinity to either: (i) JQ1; (ii) FLAG-JMJD6; or (iii) JMJD6(81-96) peptide, and subject to derivatization with propionic anhydride and digestion with trypsin. Sites are presented in alphabetical order referenced to gene name and target site (see Table S2 for summary). Figure header indicates: site assignment, peptide sequence (*derivatised lysine, [O] site of hydroxylation) and MS-related information including peptide significance score (-10 lgP value) and MSMS scan number with raw data file reference, provided in parentheses. An Ion table is provided showing the theoretical mass of each fragment ion; note, +56.03 Da and +72.02 Da corresponds to the mass of propionylated lysine and oxidised propionylated lysine, respectively. Assigned fragment ions (matching within the defined mass tolerance of 0.02 Da) are displayed in colour; *N*-terminal b-ions are blue, *C*-terminal y-ions are red.

**Table S1**

| | | # Spectra filtered by confidence of modification site | | | |
|---|---|---|---|---|---|
| Peptide delta mass | Oxidation sites | No filter | A score | Ion Intensity | A score & Ion Intensity |
| 0 | Unmodified | 66 | n/a | n/a | n/a |
| +15.99 | K535 | 9 | 8 | 1 | 1 |
| | P536 | 26 | 2 | 4 | 0 |
| | K537 | 17 | 11 | 0 | 0 |
| +31.99 | K535 / P536 | 13 | 0 | 5 | 0 |
| | K535 / K537 | 20 | 19 | 13 | 13 |

**Table S2**

| Gene | Uniprot | # Hyl Sites | Target residue(s) | MSMS assignment |
|---|---|---|---|---|
| AP3D1 | O14617 | 3 | 739; 749; 752 | Figure S14: 1-2 |
| ARGLU1 | Q9NWB6 | 5 | 13; 16; 19; 22; 23 | Figure S14: 3 |
| ARL6IP4 | Q66PJ3 | 3 | 290; 292; 294 | Figure S14: 4-5 |
| ATRX | P46100 | 2 | 1422; 1424 | Figure S14: 6-7 |
| BRD2 | P25440 | 4 | 546; 586; 589; 755 | Figure S14: 8-11 |
| BRD3 | Q15059 | 6 | 364; 487; 489; 651; 683; 684 | Figure S14: 12-17 |
| BRD4 | O60885 | 19 | 286; 289; 291; 332; 535; 537; 538; 539; 541; 544; 547; 548; 550; 552; 561; 562; 572; 575; 727 | Figure S14: 18-29 |
| BRIX1 | Q8TDN6 | 1 | 17 | Figure S14: 30 |
| CHD3 | Q12873 | 1 | 55 | Figure S14: 31 |
| CHD4 | Q14839 | 1 | 67 | Figure S14: 32 |
| CTR9 | Q6PD62 | 1 | 993 | Figure S14: 33 |
| DHX15 | O43143 | 1 | 18 | Figure S14: 34 |
| DKC1 | O60832 | 1 | 475 | Figure S14: 35 |
| FAM133B | Q5BKY9 | 4 | 90; 208; 209; 210 | Figure S14: 36-37 |
| FIP1L1 | Q6UN15 | 4 | 564; 566; 567; 569 | Figure S14: 38 |
| FRG1† | Q14331 | 3 | 27; 29; 30 | Figure S14: 39 |
| GNL3 | Q9BVP2 | 3 | 20; 23; 221 | Figure S14: 40-41 |
| HSPA8 | P11142 | 1 | 248 | Figure S14: 42 |
| JMJD6 | Q6NYC1 | 2 | 219; 307 | Figure S14: 43-44 |
| LARP7 | Q4G0J3 | 1 | 360 | Figure S14: 45 |
| LMNA | P02545 | 1 | 341 | Figure S14: 46 |
| LUC7L | Q9NQ29 | 2 | 323; 325 | Figure S14: 47 |
| LUC7L2 | Q9Y383 | 4 | 266; 269; 365; 368 | Figure S14: 48-49 |
| LUC7L3 | O95232 | 8 | 248; 249; 251; 359; 362; 375; 388; 392 | Figure S14: 50-55 |
| MRPL39 | Q9NYK5 | 1 | 322 | Figure S14: 56 |
| NKAP | Q8N5F7 | 9 | 198; 199; 200; 203; 247; 248; 250; 251; 252 | Figure S14: 57; 4A |
| NSD3 | Q9BZ95 | 1 | 199 | Figure S14: 58 |
| PHF6 | Q8IWS0 | 1 | 173 | Figure S14: 59 |
| PHRF1 | Q9P1Y6 | 4 | 1068; 1069; 1071; 1073 | Figure S14: 60 |
| PPIL4 | Q8WUA2 | 1 | 458 | Figure S14: 61 |
| RBM39 | Q14498 | 2 | 103; 119 | Figure S14: 62-63 |
| RFC1 | P35251 | 1 | 38 | Figure S14: 64 |
| RIOK1 | Q9BRS2 | 4 | 535; 539; 552; 555 | Figure S14: 65-66 |
| RPL335A | P18077 | 1 | 45 | Figure S14: 67 |
| RPS7 | P62081 | 1 | 113 | Figure S14: 68 |
| RPS25 | P62851 | 1 | 14 | Figure S14: 69 |
| SF3B2 | Q13435 | 5 | 320; 331; 332; 334; 335 | Figure S14: 70-71 |
| SREK1 | Q8WXA9 | 6 | 269; 397; 400; 414; 415; 418 | Figure S14: 72-74 |
| SREK1IP1 | Q8N9Q2 | 2 | 139; 140 | Figure S14: 75 |
| SRPK1 | Q96SB4 | 3 | 16; 18; 19 | Figure S14: 76 |
| SRRM2† | Q9UQ35 | 3 | 241; 243; 244 | Figure S14: 39 |
| SRSF11 | Q05519 | 3 | 242; 314; 411 | Figure S14: 77-79 |
| SSRP1 | Q08945 | 2 | 521; 524 | Figure S14: 80-81 |
| SUPT16H | Q9Y5B9 | 2 | 1043; 1044 | Figure 4C |
| TCOF1 | Q13428 | 7 | 1348; 1389; 1440; 1442; 1443; 1444; 1447 | Figure S14: 82-84 |
| TOP1 | P11387 | 5 | 25; 36; 39; 40; 159 | Figure S14: 85-88 |
| TSR1 | Q2NL82 | 1 | 42 | Figure S14: 89 |
| U2AF2 | P26368 | 2 | 15; 70 | Figure S14: 90-91 |
| U2SURP | O15042 | 4 | 962; 972; 980; 981 | Figure S14: 92-94 |

**Table S3**

| Gene | Uniprot | Site | Site Context |
|---|---|---|---|
| AP3D1 | O14617 | 739 | RQKLEKDKRRK |
| | | 749 | KKRKEKEKKGK |
| | | 752 | KEKEKKGKRRH |
| ARGLU1 | Q9NWB6 | 13 | SSSRSKHTKSS |
| | | 16 | RSKHTKSSKHN |
| | | 19 | HTKSSKHNKKR |
| | | 22 | SSKHNKKRSRS |
| | | 23 | SKHNKKRSRSR |
| ARL6IP4 | Q66PJ3 | 290 | RKKRGKYKDKR |
| | | 292 | KRGKYKDKRRK |
| | | 294 | GKYKDKRRKKK |
| ATRX | P46100 | 1422 | RSYKQKKKRRR |
| | | 1424 | YKQKKKRRRIK |
| BRD2 | P25440 | 546 | PISKPKRKREK |
| | | 586 | PPQPKKSKKAS |
| | | 589 | PKKSKKASGSG |
| | | 755 | TKKPPKKANEK |
| BRD3 | Q15059 | 364 | STVKRKMDGRE |
| | | 487 | QAPVNKPKKKK |
| | | 489 | PVNKPKKKKEK |
| | | 651 | SASGKKQAAKS |
| | | 683 | QLSSSKKPARK |
| | | 684 | LSSSKKPARKE |
| BRD4 | O60885 | 286 | PVKTKKGVKRK |
| | | 289 | TKKGVKRKADT |
| | | 291 | KGVKRKADTTT |
| | | 332 | PVKPPKKDVPD |
| | | 535 | QPQQNKPKKKE |
| | | 537 | QQNKPKKKEKD |
| | | 538 | QNKPKKKEKDK |
| | | 539 | NKPKKKEKDKK |
| | | 541 | PKKKEKDKKEK |
| | | 544 | KEKDKEKKKE |
| | | 547 | DKKEKKKEKHK |
| | | 548 | KKEKKKEKHKR |
| | | 550 | EKKKEKHKRKE |
| | | 552 | KKEKHKRKEEV |
| | | 561 | EVEENKKSKAK |
| | | 562 | VEENKKSKAKE |
| | | 572 | EPPPKKTKKNN |
| | | 575 | PKKTKKNNSSN |
| | | 727 | APKSKKKGHPG |
| BRIX1 | Q8TDN6 | 17 | FAVQAKKPKRN |
| CHD3 | Q12873 | 55 | RKRGPKKQKEN |
| CHD4 | Q14839 | 67 | DPKIPKSKRQK |
| CTR9 | Q6PD62 | 993 | KAEKKKAPKPE |
| DHX15 | O43143 | 18 | YPSGKKRAGTD |
| DKC1 | O60832 | 475 | KKKSKKDKKAK |
| FAM133B | Q5BKY9 | 90 | ESSSKKRQRKK |
| | | 208 | EVRAKKKKSSE |
| | | 209 | VRAKKKKSSEE |
| | | 210 | RAKKKKSSEER |
| FIP1L1 | Q6UN15 | 564 | SHRRHKHKKSK |
| | | 566 | RRHKHKKSKRS |
| | | 567 | RHKHKKSKRSK |
| | | 569 | KHKKSKRSKEG |
| FRG1† | Q14331 | 27 | KKKKSKDKKRK |
| | | 29 | KKSKDKKRKRE |
| | | 30 | KSKDKKRKREE |
| GNL3 | Q9BVP2 | 20 | CHKRYKIQKKV |
| | | 23 | RYKIQKKVREH |
| | | 221 | ITKRVKAKKNA |
| HSPA8 | P11142 | 248 | AEFKRKHKKDI |
| JMJD6 | Q6NYC1 | 219 | PRELIKVTRDE |
| | | 307 | VRGRPKLSRKW |
| LARP7 | Q4G0J3 | 360 | TKRKHKKKHKE |
| LMNA | P02545 | 341 | RLLAEKEREMA |
| LUC7L | Q9NQ29 | 323 | RDRSAKYKFSR |
| | | 325 | RSAKYKFSRER |
| LUC7L2 | Q9Y383 | 266 | SRSHSKNPKRS |
| | | 269 | HSKNPKRSRSR |
| | | 365 | RDRDRKDKKRS |
| | | 368 | DRKDKKRSYES |
| LUC7L3 | O95232 | 248 | RDERLKKEKQE |
| | | 249 | DERLKKEKQER |
| | | 251 | RLKKEKQEREE |
| | | 359 | KSRDRKSYKHR |
| | | 362 | DRKSYKHRSKS |
| | | 375 | REQDRKSKEKE |
| | | 388 | GSDDKKSSVKS |
| | | 392 | KKSSVKSGSRE |
| MRPL39 | Q9NYK5 | 322 | LERSRKMVTED |
| NKAP | Q8N5F7 | 198 | SKKRRKKKSSK |
| | | 199 | KKRRKKKSSKR |
| | | 200 | KRRKKKSSKRK |
| | | 203 | KKKSSKRKHKK |
| | | 247 | KKHRSKKYKKK |
| | | 248 | KHRSKKYKKKR |
| | | 250 | RSKKYKKKRSK |
| | | 251 | SKKYKKKRSKK |
| | | 252 | KKYKKKRSKKS |
| NSD3 | Q9BZ95 | 199 | KEKRKKSNKHD |
| PHF6 | Q8IWS0 | 173 | RKTNFKGLSED |
| PHRF1 | Q9P1Y6 | 1068 | ERAKRKKAKDK |
| | | 1069 | RAKRKKAKDKS |
| | | 1071 | KRKKAKDKSRE |
| | | 1073 | KKAKDKSREHR |
| PPIL4 | Q8WUA2 | 458 | YSNSHKSKYQT |
| RBM39 | Q14498 | 103 | PYSGPKFNSAI |
| | | 119 | LPHSIKLSRRR |
| RFC1 | P35251 | 38 | TLKAKKGIKEI |
| RIOK1 | Q9BRS2 | 535 | DKKERKKMVKE |
| | | 539 | RKKMVKEAQRE |
| | | 552 | KNKIPKHVKKR |
| | | 555 | IPKHVKKRKEK |
| RPL335A | P18077 | 45 | EFYLGKRCAYV |
| RPS7 | P62081 | 113 | SRTKNKQKRPR |
| RPS25 | P62851 | 14 | KKDAGKSAKKD |
| SF3B2 | Q13435 | 320 | TVSVSKKEKNR |
| | | 331 | KRRNRKKKKKP |
| | | 332 | RRNRKKKKKPQ |
| | | 334 | NRKKKKKPQRV |
| | | 335 | RKKKKKPQRVR |
| SREK1 | Q8WXA9 | 269 | TREKIKEKERV |
| | | 397 | SPRTSKTIKRK |
| | | 400 | TSKTIKRKSSR |
| | | 414 | PRSRNKKDKKR |
| | | 415 | RSRNKKDKKRE |
| | | 418 | NKKDKKREKER |
| SREK1IP1 | Q8N9Q2 | 139 | EKKKRKKEKHS |
| | | 140 | KKKRKKEKHSS |
| SRPK1 | Q96SB4 | 16 | RKKRTKAKKDK |
| | | 18 | KRTKAKKDKAQ |
| | | 19 | RTKAKKDKAQR |
| SRRM2† | Q9UQ35 | 241 | SKRKSKDKKRK |
| | | 243 | RKSKDKKRKRS |
| | | 244 | KSKDKKRKRSR |
| SRSF11 | Q05519 | 242 | IEPDKKEEKRR |
| | | 314 | KTRDKKKEDKE |
| | | 411 | KDRERKSESDK |
| SSRP1 | Q08945 | 521 | KRKQLKKAKMA |
| | | 524 | QLKKAKMAKDR |
| SUPT16H | Q9Y5B9 | 1043 | SSAPPKKKRK- |
| | | 1044 | SAPPKKKRK-- |
| TCOF1 | Q13428 | 1348 | ESRKRKLSGDQ |
| | | 1389 | TTSKGKAKRDK |
| | | 1440 | NPKSKKEKKKS |
| | | 1442 | KSKKEKKKSDK |
| | | 1443 | SKKEKKKSDKR |
| | | 1444 | KKEKKKSDKRK |
| | | 1447 | KKKSDKRKKDK |
| TOP1 | P11387 | 25 | DSHKHKDKHKD |
| | | 36 | REHRHKEHKKE |
| | | 39 | RHKEHKKEKDR |
| | | 40 | HKEHKKEKDRE |
| | | 159 | TEDTKKEKKRK |
| TSR1 | Q2NL82 | 42 | LKTLSKKVRKE |
| U2AF2 | P26368 | 15 | QLNENKQERDK |
| | | 70 | LTRGAKEEHGG |
| U2SURP | O15042 | 962 | SERSHKESSRS |
| | | 972 | SRSSHKDSPRD |
| | | 980 | PRDVSKKAKRS |
| | | 981 | RDVSKKAKRSP |

**Figure S1**

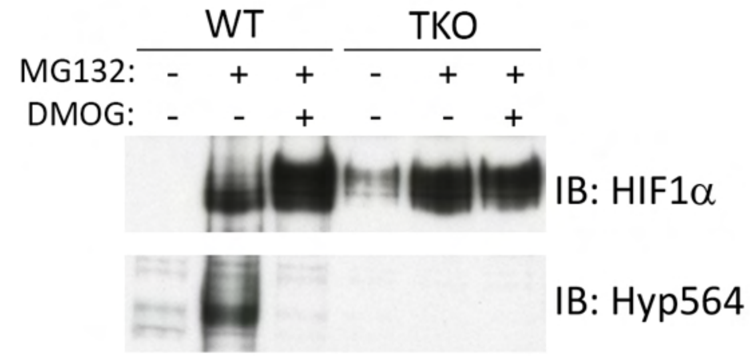

**Figure S2**

## (A) BRD4(521-538): AVHEQLAALSQPQQNKPK; -10lgP=60.84; Scan 7844 (MC91)

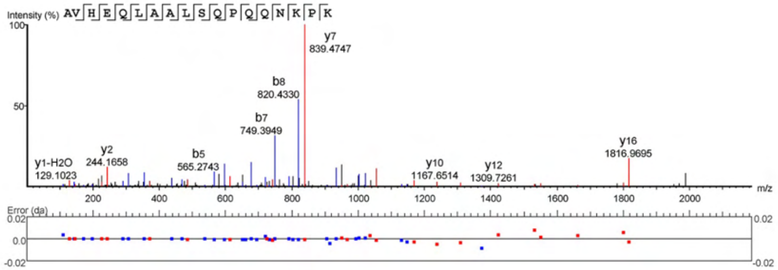


| # | Immonium | b | b-H2O | a | b (2+) | Seq | y | y-H2O | y (2+) | # |
|---|---|---|---|---|---|---|---|---|---|---|
| 1 | 44.0499 | 72.0449 | 54.0344 | 44.0499 | 36.5225 | A | | | | 18 |
| 2 | 72.0812 | 171.1129 | 153.1028 | 143.1177 | 86.0567 | V | 1916.0348 | 1898.0243 | 958.5174 | 17 |
| 3 | 110.0676 | 308.1721 | 290.1613 | 280.1772 | 154.5861 | H | 1816.9695 | 1798.9496 | 908.9832 | 16 |
| 4 | 102.0554 | 437.2152 | 419.2043 | 409.2198 | 219.1074 | E | 1679.9075 | 1661.8938 | 840.4537 | 15 |
| 5 | 101.0714 | 565.2743 | 547.2629 | 537.2789 | 283.1367 | Q | 1550.8632 | 1532.8458 | 775.9324 | 14 |
| 6 | 86.0969 | 678.3580 | 660.3478 | 650.3636 | 339.6787 | L | 1422.8022 | 1404.7958 | 711.9031 | 13 |
| 7 | 44.0499 | 749.3949 | 731.3848 | 721.3969 | 375.1973 | A | 1309.7261 | 1291.7117 | 655.3611 | 12 |
| 8 | 44.0499 | 820.4330 | 802.4223 | 792.4372 | 410.7159 | A | 1238.6902 | 1220.6746 | 619.8425 | 11 |
| 9 | 86.0969 | 933.5153 | 915.5098 | 905.5204 | 467.2579 | L | 1167.6514 | 1149.6375 | 584.3240 | 10 |
| 10 | 60.0449 | 1020.5464 | 1002.5360 | 992.5529 | 510.7739 | S | 1054.5660 | 1036.5502 | 527.7820 | 9 |
| 11 | 101.0714 | 1148.6095 | 1130.5979 | 1120.6113 | 574.8032 | Q | 967.5331 | 949.5206 | 484.2660 | 8 |
| 12 | 70.0656 | 1245.6592 | 1227.6487 | 1217.6642 | 623.3296 | P | 839.4747 | 821.4628 | 420.2367 | 7 |
| 13 | 101.0714 | 1373.7263 | 1355.7072 | 1345.7227 | 687.3588 | Q | 742.4227 | 724.4098 | 371.7103 | 6 |
| 14 | 101.0714 | 1501.7762 | 1483.7657 | 1473.7812 | 751.3881 | Q | 614.3633 | 596.3514 | 307.6810 | 5 |
| 15 | 87.0558 | 1615.8192 | 1597.8087 | 1587.8242 | 808.4096 | N | 486.3044 | 468.2928 | 243.6517 | 4 |
| 16 | 101.1078 | 1743.9142 | 1725.9037 | 1715.9192 | 872.4571 | K | 372.2612 | 354.2499 | 186.6302 | 3 |
| 17 | 70.0656 | 1840.9669 | 1822.9564 | 1812.9719 | 920.9835 | P | 244.1658 | 226.1552 | 122.5828 | 2 |
| 18 | 101.1078 | | | | | K | 147.1127 | 129.1023 | 74.0564 | 1 |

## (B) BRD4(521-538)AVHEQLAALSQPQQNK[O]PK; -10lgP=59.64; Scan 7620 (MC91)

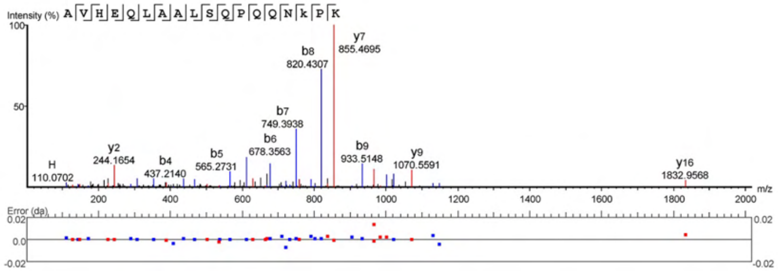


| # | Immonium | b | b-H2O | a | b (2+) | Seq | y | y-H2O | y (2+) | # |
|---|---|---|---|---|---|---|---|---|---|---|
| 1 | 44.0499 | 72.0449 | 54.0344 | 44.0499 | 36.5225 | A | | | | 18 |
| 2 | 72.0812 | 171.1125 | 153.1028 | 143.1177 | 86.0567 | V | 1932.0297 | 1914.0192 | 966.5009 | 17 |
| 3 | 110.0702 | 308.1717 | 290.1609 | 280.1772 | 154.5861 | H | 1832.9568 | 1814.9508 | 916.9807 | 16 |
| 4 | 102.0554 | 437.2140 | 419.2043 | 409.2241 | 219.1074 | E | 1695.9023 | 1677.8918 | 848.4512 | 15 |
| 5 | 101.0714 | 565.2731 | 547.2629 | 537.2784 | 283.1367 | Q | 1566.8597 | 1548.8492 | 783.9299 | 14 |
| 6 | 86.0969 | 678.3563 | 660.3469 | 650.3625 | 339.6787 | L | 1438.8011 | 1420.7906 | 719.9006 | 13 |
| 7 | 44.0499 | 749.3938 | 731.3837 | 721.4071 | 375.1973 | A | 1325.7172 | 1307.7067 | 663.3591 | 12 |
| 8 | 44.0499 | 820.4307 | 802.4204 | 792.4333 | 410.7159 | A | 1254.6801 | 1236.6696 | 627.8400 | 11 |
| 9 | 86.0969 | 933.5148 | 915.5052 | 905.5182 | 467.2579 | L | 1183.6429 | 1165.6324 | 592.3215 | 10 |
| 10 | 60.0449 | 1020.5482 | 1002.5347 | 992.5528 | 510.7739 | S | 1070.5591 | 1052.5483 | 535.7822 | 9 |
| 11 | 101.0714 | 1148.6107 | 1130.5918 | 1120.6113 | 574.8032 | Q | 983.5244 | 965.5179 | 492.2634 | 8 |
| 12 | 70.0656 | 1245.6592 | 1227.6487 | 1217.6642 | 623.3296 | P | 855.4695 | 837.4547 | 428.2341 | 7 |
| 13 | 101.0714 | 1373.7177 | 1355.7072 | 1345.7227 | 687.3588 | Q | 758.4158 | 740.4050 | 379.7078 | 6 |
| 14 | 101.0714 | 1501.7762 | 1483.7657 | 1473.7812 | 751.3881 | Q | 630.3575 | 612.3464 | 315.6785 | 5 |
| 15 | 87.0558 | 1615.8192 | 1597.8087 | 1587.8242 | 808.4096 | N | 502.2989 | 484.2878 | 251.6492 | 4 |
| 16 | 117.1027 | 1759.9091 | 1741.8986 | 1731.9141 | 880.4545 | K(+15.99) | 388.2566 | 370.2448 | 194.6277 | 3 |
| 17 | 70.0656 | 1856.9618 | 1838.9513 | 1828.9668 | 928.9809 | P | 244.1654 | 226.1551 | 122.5828 | 2 |
| 18 | 101.1078 | | | | | K | 147.1126 | 129.1018 | 74.0564 | 1 |

## (C) BRD4(521-538)AVHEQLAALSQPQQNK[O]PK[O]; -10lgP=58.47; Scan 7381 (MC91)

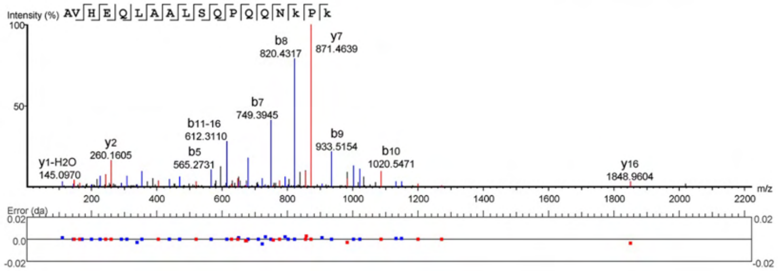


| # | Immonium | b | b-H2O | a | b (2+) | Seq | y | y-H2O | y (2+) | # |
|---|---|---|---|---|---|---|---|---|---|---|
| 1 | 44.0499 | 72.0449 | 54.0344 | 44.0499 | 36.5225 | A | | | | 18 |
| 2 | 72.0812 | 171.1129 | 153.1028 | 143.1177 | 86.0567 | V | 1948.0247 | 1930.0142 | 974.5123 | 17 |
| 3 | 110.0699 | 308.1717 | 290.1613 | 280.1772 | 154.5861 | H | 1848.9604 | 1830.9457 | 924.9781 | 16 |
| 4 | 102.0554 | 437.2146 | 419.2043 | 409.2198 | 219.1074 | E | 1711.8973 | 1693.8868 | 856.4456 | 15 |
| 5 | 101.0714 | 565.2731 | 547.2629 | 537.2784 | 283.1367 | Q | 1582.8547 | 1564.8442 | 791.9274 | 14 |
| 6 | 86.0969 | 678.3568 | 660.3469 | 650.3606 | 339.6821 | L | 1454.7961 | 1436.7856 | 727.8981 | 13 |
| 7 | 44.0499 | 749.3945 | 731.3812 | 721.4043 | 375.1973 | A | 1341.7120 | 1323.7015 | 671.3579 | 12 |
| 8 | 44.0499 | 820.4317 | 802.4205 | 792.4340 | 410.7159 | A | 1270.6748 | 1252.6644 | 635.8375 | 11 |
| 9 | 86.0969 | 933.5154 | 915.5052 | 905.5186 | 467.2579 | L | 1199.6379 | 1181.6273 | 600.3189 | 10 |
| 10 | 60.0449 | 1020.5471 | 1002.5377 | 992.5528 | 510.7739 | S | 1086.5542 | 1068.5433 | 543.7769 | 9 |
| 11 | 101.0714 | 1148.6052 | 1130.5945 | 1120.6113 | 574.8032 | Q | 999.5217 | 981.5145 | 500.2609 | 8 |
| 12 | 70.0656 | 1245.6592 | 1227.6487 | 1217.6642 | 623.3296 | P | 871.4639 | 853.4525 | 436.2316 | 7 |
| 13 | 101.0714 | 1373.7177 | 1355.7072 | 1345.7227 | 687.3588 | Q | 774.4100 | 756.4008 | 387.7052 | 6 |
| 14 | 101.0714 | 1501.7762 | 1483.7657 | 1473.7812 | 751.3881 | Q | 646.3522 | 628.3416 | 323.6759 | 5 |
| 15 | 87.0558 | 1615.8192 | 1597.8087 | 1587.8242 | 808.4096 | N | 518.2935 | 500.2827 | 259.6466 | 4 |
| 16 | 117.1027 | 1759.9091 | 1741.8986 | 1731.9141 | 880.4545 | K(+15.99) | 404.2503 | 386.2398 | 202.6252 | 3 |
| 17 | 70.0656 | 1856.9618 | 1838.9513 | 1828.9668 | 928.9809 | P | 260.1605 | 242.1499 | 130.5802 | 2 |
| 18 | 117.1027 | | | | | K(+15.99) | 163.1077 | 145.0970 | 82.0539 | 1 |

## Figure S3

**(A) BRD4(520-537)AVHEQLAALSQPQQNKPK; -10lgP=72.53; Scan 4653 (MC15)**

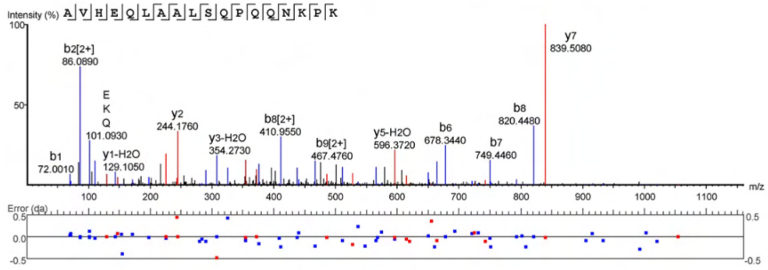


| # | Immonium | b | b-H2O | a | b (2+) | Seq | y | y-H2O | y (2+) | # |
|---|---|---|---|---|---|---|---|---|---|---|
| 1 | 44.0499 | 72.0010 | 54.0344 | 44.0499 | 36.5225 | A | | | | 18 |
| 2 | 72.0010 | 171.0460 | 153.0430 | 143.1320 | 86.0890 | V | 1916.0348 | 1898.0243 | 958.5174 | 17 |
| 3 | 110.1100 | 308.1680 | 290.2800 | 280.2970 | 154.9950 | H | 1816.9663 | 1798.9558 | 908.9832 | 16 |
| 4 | 101.9190 | 437.2480 | 419.2043 | 409.2580 | 219.1074 | E | 1679.9075 | 1661.8970 | 840.4537 | 15 |
| 5 | 101.0930 | 565.3670 | 547.4870 | 537.0430 | 283.2030 | Q | 1550.8649 | 1532.8544 | 775.9324 | 14 |
| 6 | 86.0890 | 678.3440 | 660.5960 | 650.3300 | 339.6787 | L | 1422.8063 | 1404.7958 | 711.9031 | 13 |
| 7 | 44.0499 | 749.4460 | 731.2830 | 721.3250 | 375.3620 | A | 1309.7222 | 1291.7117 | 655.0030 | 12 |
| 8 | 44.0499 | 820.4480 | 802.3880 | 792.4440 | 410.9550 | A | 1238.6851 | 1220.6746 | 619.9520 | 11 |
| 9 | 86.0969 | 933.6070 | 915.4190 | 905.6110 | 467.4760 | L | 1167.6479 | 1149.6375 | 584.3240 | 10 |
| 10 | 60.0449 | 1020.6660 | 1002.4430 | 992.8500 | 510.8680 | S | 1054.5601 | 1036.5535 | 527.9780 | 9 |
| 11 | 101.0714 | 1148.6063 | 1130.5958 | 1120.6113 | 574.6890 | Q | 967.5319 | 949.5214 | 484.2660 | 8 |
| 12 | 70.0160 | 1245.6592 | 1227.6487 | 1217.6642 | 623.3296 | P | 839.5080 | 821.4628 | 420.2367 | 7 |
| 13 | 101.0714 | 1373.7177 | 1355.7072 | 1345.7227 | 687.3588 | Q | 742.5350 | 724.3090 | 371.7103 | 6 |
| 14 | 101.0714 | 1501.7762 | 1483.7657 | 1473.7812 | 751.6310 | Q | 614.4230 | 596.3720 | 308.1680 | 5 |
| 15 | 87.0558 | 1615.8192 | 1597.8087 | 1587.8242 | 808.6460 | N | 486.3300 | 468.2928 | 243.2000 | 4 |
| 16 | 101.0930 | 1743.9142 | 1725.9037 | 1715.9192 | 872.4571 | K | 372.2480 | 354.2730 | 186.6302 | 3 |
| 17 | 70.0656 | 1840.9669 | 1822.9564 | 1812.9719 | 920.9835 | P | 244.1760 | 226.1670 | 122.5828 | 2 |
| 18 | 101.1078 | | | | | K | 147.0250 | 129.1050 | 74.0564 | 1 |

**(B) BRD4(520-537)AVHEQLAALSQPQQNKPK[O]; -10lgP=62.03; Scan 4581 (MC15)**

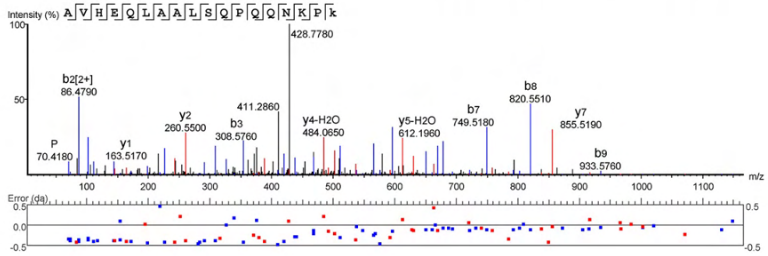


| # | Immonium | b | b-H2O | a | b (2+) | Seq | y | y-H2O | y (2+) | # |
|---|---|---|---|---|---|---|---|---|---|---|
| 1 | 44.0499 | 72.4430 | 54.0344 | 44.0499 | 36.5225 | A | | | | 18 |
| 2 | 72.4430 | 171.5200 | 153.4850 | 143.5170 | 86.4790 | V | 1932.0297 | 1914.0192 | 966.6290 | 17 |
| 3 | 110.4890 | 308.5760 | 290.5510 | 280.6430 | 154.4760 | H | 1832.9613 | 1814.9508 | 916.8350 | 16 |
| 4 | 102.0554 | 437.5110 | 419.6160 | 409.7040 | 218.6440 | E | 1695.9023 | 1677.8918 | 848.8810 | 15 |
| 5 | 101.4420 | 565.5360 | 547.4040 | 537.3280 | 283.5730 | Q | 1566.8597 | 1548.8492 | 784.2820 | 14 |
| 6 | 86.4790 | 678.4490 | 660.4770 | 650.4880 | 339.4990 | L | 1438.8011 | 1420.7906 | 719.8280 | 13 |
| 7 | 44.0499 | 749.5180 | 731.4690 | 721.5360 | 375.0740 | A | 1325.7172 | 1307.7067 | 662.9320 | 12 |
| 8 | 44.0499 | 820.5510 | 802.5020 | 792.5550 | 410.7159 | A | 1254.6801 | 1236.6696 | 627.9730 | 11 |
| 9 | 86.0969 | 933.5760 | 915.6070 | 905.6410 | 467.4760 | L | 1183.6429 | 1165.6324 | 592.6450 | 10 |
| 10 | 60.0449 | 1020.5710 | 1002.5950 | 992.5528 | 511.0830 | S | 1070.7980 | 1052.5483 | 536.1490 | 9 |
| 11 | 101.0714 | 1148.4969 | 1130.7200 | 1120.6113 | 575.2750 | Q | 983.5040 | 965.4450 | 492.4240 | 8 |
| 12 | 70.4180 | 1245.6592 | 1227.6487 | 1217.6642 | 623.3296 | P | 855.5190 | 837.5600 | 428.1330 | 7 |
| 13 | 101.0714 | 1373.7177 | 1355.7072 | 1345.7227 | 687.4470 | Q | 758.5540 | 740.4870 | 380.0200 | 6 |
| 14 | 101.0714 | 1501.7762 | 1483.7657 | 1473.7812 | 751.3881 | Q | 630.5060 | 612.1960 | 316.0060 | 5 |
| 15 | 87.0558 | 1615.8192 | 1597.8087 | 1587.8242 | 808.3820 | N | 502.5250 | 484.0650 | 251.4230 | 4 |
| 16 | 101.4420 | 1743.9142 | 1725.9037 | 1715.9192 | 872.5410 | K | 388.6740 | 370.4990 | 194.5900 | 3 |
| 17 | 70.0656 | 1840.9669 | 1822.9564 | 1812.9719 | 920.9835 | P | 260.5500 | 242.5790 | 130.5802 | 2 |
| 18 | 117.5040 | | | | | K(+15.99) | 163.5170 | 145.4960 | 82.4900 | 1 |

**(C) BRD4(520-537)AVHEQLAALSQPQQNK[O]PK[O]; -10lgP=57.70; Scan 4745 (MC15)**

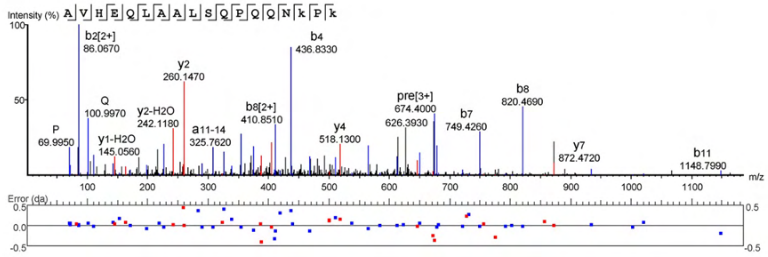


| # | Immonium | b | b-H2O | a | b (2+) | Seq | y | y-H2O | y (2+) | # |
|---|---|---|---|---|---|---|---|---|---|---|
| 1 | 44.0499 | 72.0090 | 54.0344 | 44.0499 | 36.5225 | A | | | | 18 |
| 2 | 72.0090 | 171.1240 | 152.9240 | 143.0250 | 86.0670 | V | 1948.0247 | 1930.0142 | 974.5123 | 17 |
| 3 | 110.0920 | 308.2180 | 290.2190 | 280.1772 | 154.5861 | H | 1848.9562 | 1830.9457 | 924.9781 | 16 |
| 4 | 102.0554 | 436.8330 | 418.8850 | 409.5470 | 219.0320 | E | 1711.8973 | 1693.8868 | 856.3380 | 15 |
| 5 | 100.9970 | 565.3550 | 547.2629 | 537.2180 | 282.7570 | Q | 1582.8547 | 1564.8442 | 791.9274 | 14 |
| 6 | 86.0670 | 678.4140 | 660.3469 | 650.3020 | 339.5070 | L | 1454.7961 | 1436.7856 | 727.6640 | 13 |
| 7 | 44.0499 | 749.4260 | 731.1060 | 721.4300 | 375.3130 | A | 1341.7120 | 1323.7015 | 671.6200 | 12 |
| 8 | 44.0499 | 820.4690 | 802.4330 | 792.4650 | 410.8510 | A | 1270.6749 | 1252.6644 | 635.8375 | 11 |
| 9 | 86.0969 | 933.4840 | 915.5052 | 905.5208 | 467.3970 | L | 1199.6378 | 1181.6273 | 600.3189 | 10 |
| 10 | 60.0449 | 1020.4660 | 1002.5860 | 992.5528 | 510.5700 | S | 1086.5538 | 1068.5433 | 543.7769 | 9 |
| 11 | 101.0714 | 1148.7990 | 1130.5958 | 1120.6113 | 574.8032 | Q | 999.5217 | 981.5112 | 500.1390 | 8 |
| 12 | 69.9950 | 1245.6592 | 1227.6487 | 1217.6642 | 623.3090 | P | 871.4680 | 853.4526 | 436.2316 | 7 |
| 13 | 101.0714 | 1373.7177 | 1355.7072 | 1345.7227 | 687.3588 | Q | 774.7100 | 756.3480 | 388.1160 | 6 |
| 14 | 101.0714 | 1501.7762 | 1483.7657 | 1473.7812 | 751.3881 | Q | 646.3900 | 628.3412 | 323.5900 | 5 |
| 15 | 87.0558 | 1615.8192 | 1597.8087 | 1587.8242 | 808.4096 | N | 518.1300 | 500.1390 | 259.1960 | 4 |
| 16 | 117.1027 | 1759.9091 | 1741.8986 | 1731.9141 | 880.4545 | K(+15.99) | 404.2900 | 386.1840 | 202.6252 | 3 |
| 17 | 70.0656 | 1856.9618 | 1838.9513 | 1828.9668 | 928.9809 | P | 260.1470 | 242.1180 | 130.5802 | 2 |
| 18 | 117.1027 | | | | | K(+15.99) | 163.0180 | 145.0560 | 82.0000 | 1 |

**Figure S4**

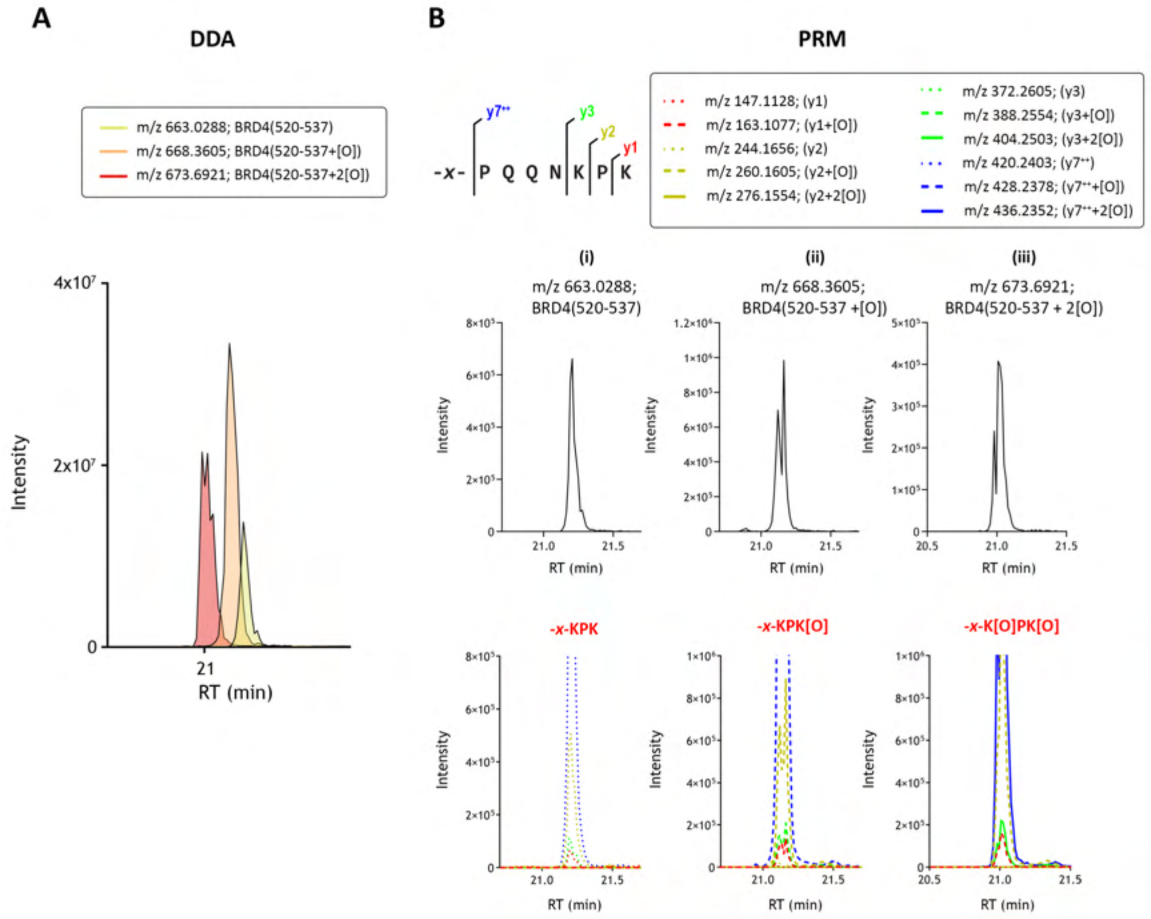
A
DDA
m/z 663.0288; BRD4(520-537)
m/z 668.3605; BRD4(520-537+[O])
m/z 673.6921; BRD4(520-537+2[O])
Intensity
RT (min)
B
PRM
-x- P Q Q N K P K
y7++
y3
y2
y1
m/z 147.1128; (y1)
m/z 163.1077; (y1+[O])
m/z 244.1656; (y2)
m/z 260.1605; (y2+[O])
m/z 276.1554; (y2+2[O])
m/z 372.2605; (y3)
m/z 388.2554; (y3+[O])
m/z 404.2503; (y3+2[O])
m/z 420.2403; (y7++)
m/z 428.2378; (y7++ +[O])
m/z 436.2352; (y7++ +2[O])
(i)
m/z 663.0288; BRD4(520-537)
(ii)
m/z 668.3605; BRD4(520-537 +[O])
(iii)
m/z 673.6921; BRD4(520-537 + 2[O])
-x-KPK
-x-KPK[O]
-x-K[O]PK[O]
Intensity
RT (min)

**Figure S5**

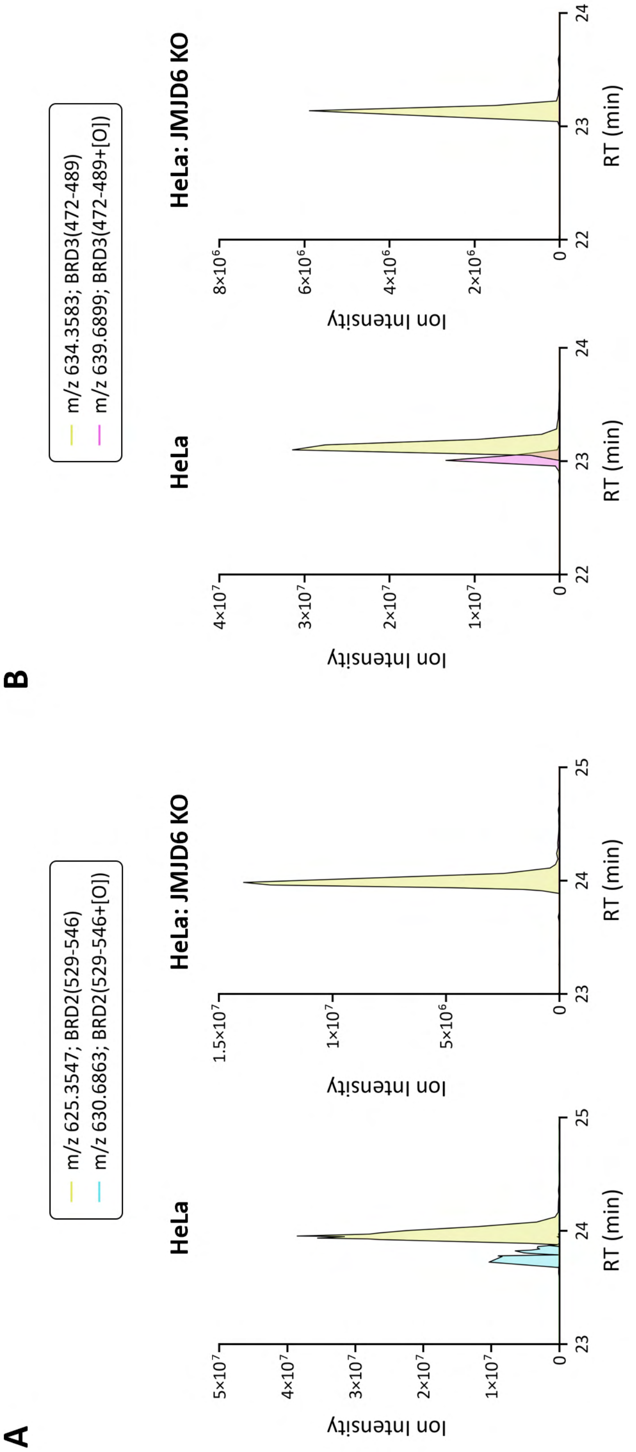
A
B
HeLa
HeLa: JMJD6 KO
m/z 625.3547; BRD2(529-546)
m/z 630.6863; BRD2(529-546+[O])
m/z 634.3583; BRD3(472-489)
m/z 639.6899; BRD3(472-489+[O])
Ion Intensity
RT (min)

**Figure S6(A-C)**

**(A) BRD4(523-541): EQLAALSQPQQNKPKKKEK;** -10lgP=22.68; Scan 4909 (MC46)

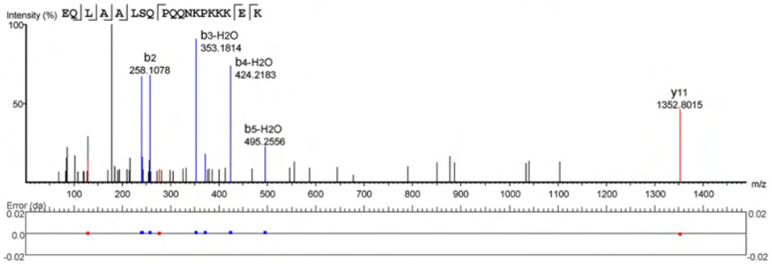


| # | b | b-H2O | b-NH3 | b (2+) | Seq | y | y-H2O | y-NH3 | y (2+) | # |
|---|---|---|---|---|---|---|---|---|---|---|
| 1 | 130.0504 | 112.0399 | 113.0234 | 65.5252 | E | | | | | 19 |
| 2 | 258.1078 | 240.0974 | 241.0813 | 129.5545 | Q | 2064.1924 | 2046.1818 | 2047.1653 | 1032.5962 | 18 |
| 3 | 371.1920 | 353.1814 | 354.1661 | 186.0965 | L | 1936.1338 | 1918.1233 | 1919.1068 | 968.5669 | 17 |
| 4 | 442.2302 | 424.2183 | 425.2032 | 221.6151 | A | 1823.0497 | 1805.0392 | 1806.0227 | 912.0248 | 16 |
| 5 | 513.2673 | 495.2556 | 496.2403 | 257.1336 | A | 1752.0126 | 1734.0021 | 1734.9856 | 876.5063 | 15 |
| 6 | 626.3513 | 608.3408 | 609.3243 | 313.6757 | L | 1680.9755 | 1662.9650 | 1663.9485 | 840.9877 | 14 |
| 7 | 713.3834 | 695.3728 | 696.3564 | 357.1917 | S | 1567.8914 | 1549.8809 | 1550.8644 | 784.4457 | 13 |
| 8 | 841.4419 | 823.4313 | 824.4149 | 421.2209 | Q | 1480.8594 | 1462.8489 | 1463.8324 | 740.9297 | 12 |
| 9 | 938.4947 | 920.4841 | 921.4677 | 469.7473 | P | 1352.8015 | 1334.7903 | 1335.7738 | 676.9004 | 11 |
| 10 | 1066.5532 | 1048.5427 | 1049.5262 | 533.7766 | Q | 1255.7480 | 1237.7375 | 1238.7211 | 628.3740 | 10 |
| 11 | 1194.6118 | 1176.6013 | 1177.5848 | 597.8059 | Q | 1127.6895 | 1109.6790 | 1110.6625 | 564.3447 | 9 |
| 12 | 1308.6548 | 1290.6443 | 1291.6278 | 654.8274 | N | 999.6309 | 981.6203 | 982.6039 | 500.3154 | 8 |
| 13 | 1436.7498 | 1418.7393 | 1419.7228 | 718.8749 | K | 885.5880 | 867.5774 | 868.5610 | 443.2940 | 7 |
| 14 | 1533.8025 | 1515.7920 | 1516.7755 | 767.4012 | P | 757.4930 | 739.4824 | 740.4660 | 379.2465 | 6 |
| 15 | 1661.8975 | 1643.8870 | 1644.8705 | 831.4487 | K | 660.4402 | 642.4297 | 643.4133 | 330.7201 | 5 |
| 16 | 1789.9924 | 1771.9819 | 1772.9655 | 895.4962 | K | 532.3453 | 514.3347 | 515.3183 | 266.6726 | 4 |
| 17 | 1918.0874 | 1900.0769 | 1901.0604 | 959.5437 | K | 404.2503 | 386.2397 | 387.2233 | 202.6252 | 3 |
| 18 | 2047.1299 | 2029.1194 | 2030.1029 | 1024.0649 | E | 276.1547 | 258.1448 | 259.1284 | 138.5777 | 2 |
| 19 | | | | | K | 147.1128 | 129.1019 | 130.0858 | 74.0564 | 1 |

**(B) BRD4(523-541): EQLAALSQPQQNKPKK[O]KEK;** -10lgP=43.39; Scan 4813 (MC46)

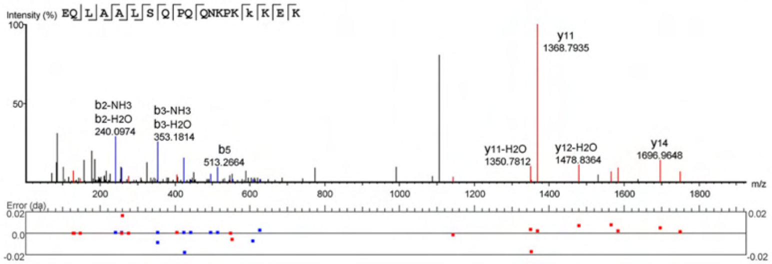


| # | b | b-H2O | b-NH3 | b (2+) | Seq | y | y-H2O | y-NH3 | y (2+) | # |
|---|---|---|---|---|---|---|---|---|---|---|
| 1 | 130.0504 | 112.0399 | 113.0234 | 65.5252 | E | | | | | 19 |
| 2 | 258.1078 | 240.0974 | 241.0813 | 129.5545 | Q | 2080.1873 | 2062.1768 | 2063.1602 | 1040.5936 | 18 |
| 3 | 371.1931 | 353.1814 | 354.1751 | 186.0965 | L | 1952.1287 | 1934.1182 | 1935.1017 | 976.5643 | 17 |
| 4 | 442.2290 | 424.2183 | 425.2214 | 221.6151 | A | 1839.0446 | 1821.0341 | 1822.0176 | 920.0223 | 16 |
| 5 | 513.2664 | 495.2556 | 496.2403 | 257.1336 | A | 1768.0074 | 1749.9950 | 1750.9805 | 884.5037 | 15 |
| 6 | 626.3484 | 608.3486 | 609.3243 | 313.6757 | L | 1696.9648 | 1678.9598 | 1679.9434 | 848.9852 | 14 |
| 7 | 713.3834 | 695.3728 | 696.3564 | 357.1917 | S | 1583.8835 | 1565.8677 | 1566.8594 | 792.4432 | 13 |
| 8 | 841.4419 | 823.4313 | 824.4149 | 421.2209 | Q | 1496.8542 | 1478.8364 | 1479.8273 | 748.9271 | 12 |
| 9 | 938.4947 | 920.4841 | 921.4677 | 469.7473 | P | 1368.7935 | 1350.7812 | 1351.7861 | 684.8978 | 11 |
| 10 | 1066.5532 | 1048.5427 | 1049.5262 | 533.7766 | Q | 1271.7429 | 1253.7324 | 1254.7159 | 636.3715 | 10 |
| 11 | 1194.6118 | 1176.6013 | 1177.5848 | 597.8059 | Q | 1143.6858 | 1125.6738 | 1126.6573 | 572.3422 | 9 |
| 12 | 1308.6548 | 1290.6443 | 1291.6278 | 654.8274 | N | 1015.6258 | 997.6152 | 998.5988 | 508.3129 | 8 |
| 13 | 1436.7498 | 1418.7393 | 1419.7228 | 718.8749 | K | 901.5829 | 883.5723 | 884.5559 | 451.2914 | 7 |
| 14 | 1533.8025 | 1515.7920 | 1516.7755 | 767.4012 | P | 773.4879 | 755.4774 | 756.4609 | 387.2440 | 6 |
| 15 | 1661.8975 | 1643.8870 | 1644.8705 | 831.4487 | K | 676.4352 | 658.4246 | 659.4082 | 338.7176 | 5 |
| 16 | 1805.9873 | 1787.9768 | 1788.9603 | 903.4937 | K(+15.99) | 548.3397 | 530.3297 | 531.3132 | 274.6701 | 4 |
| 17 | 1934.0823 | 1916.0718 | 1917.0553 | 967.5411 | K | 404.2493 | 386.2397 | 387.2233 | 202.6252 | 3 |
| 18 | 2063.1248 | 2045.1143 | 2046.0978 | 1032.0624 | E | 276.1547 | 258.1443 | 259.1119 | 138.5777 | 2 |
| 19 | | | | | K | 147.1127 | 129.1019 | 130.0862 | 74.0564 | 1 |

**(C) BRD4(523-541): EQLAALSQPQQNKPKK[O]KEK[O];** -10lgP=40.81; Scan 4732 (MC46)

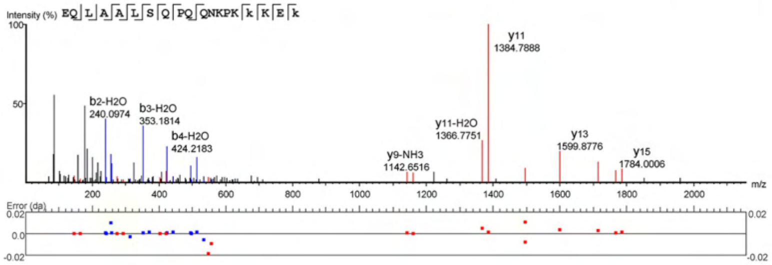


| # | b | b-H2O | b-NH3 | b (2+) | Seq | y | y-H2O | y-NH3 | y (2+) | # |
|---|---|---|---|---|---|---|---|---|---|---|
| 1 | 130.0504 | 112.0399 | 113.0234 | 65.5252 | E | | | | | 19 |
| 2 | 258.1078 | 240.0974 | 241.0815 | 129.5545 | Q | 2096.1821 | 2078.1716 | 2079.1550 | 1048.5911 | 18 |
| 3 | 371.1915 | 353.1814 | 354.1661 | 186.0965 | L | 1968.1235 | 1950.1130 | 1951.0966 | 984.5618 | 17 |
| 4 | 442.2284 | 424.2183 | 425.2032 | 221.6151 | A | 1855.0396 | 1837.0291 | 1838.0126 | 928.0198 | 16 |
| 5 | 513.2655 | 495.2556 | 496.2401 | 257.1236 | A | 1784.0006 | 1765.9907 | 1766.9755 | 892.5012 | 15 |
| 6 | 626.3513 | 608.3408 | 609.3243 | 313.6786 | L | 1712.9624 | 1694.9548 | 1695.9384 | 856.9827 | 14 |
| 7 | 713.3834 | 695.3728 | 696.3564 | 357.1917 | S | 1599.8776 | 1581.8707 | 1582.8542 | 800.4406 | 13 |
| 8 | 841.4419 | 823.4313 | 824.4149 | 421.2209 | Q | 1512.8492 | 1494.8278 | 1495.8307 | 756.9246 | 12 |
| 9 | 938.4947 | 920.4841 | 921.4677 | 469.7473 | P | 1384.7888 | 1366.7751 | 1367.7637 | 692.8953 | 11 |
| 10 | 1066.5532 | 1048.5427 | 1049.5262 | 533.7830 | Q | 1287.7379 | 1269.7274 | 1270.7109 | 644.3690 | 10 |
| 11 | 1194.6118 | 1176.6013 | 1177.5848 | 597.8059 | Q | 1159.6787 | 1141.6688 | 1142.6516 | 580.3397 | 9 |
| 12 | 1308.6548 | 1290.6443 | 1291.6278 | 654.8274 | N | 1031.6207 | 1013.6102 | 1014.5938 | 516.3104 | 8 |
| 13 | 1436.7498 | 1418.7393 | 1419.7228 | 718.8749 | K | 917.5778 | 899.5672 | 900.5508 | 459.2889 | 7 |
| 14 | 1533.8025 | 1515.7920 | 1516.7755 | 767.4012 | P | 789.4828 | 771.4723 | 772.4559 | 395.2414 | 6 |
| 15 | 1661.8975 | 1643.8870 | 1644.8705 | 831.4487 | K | 692.4301 | 674.4195 | 675.4031 | 346.7150 | 5 |
| 16 | 1805.9873 | 1787.9768 | 1788.9603 | 903.4937 | K(+15.99) | 564.3351 | 546.3245 | 547.3268 | 282.6675 | 4 |
| 17 | 1934.0823 | 1916.0718 | 1917.0553 | 967.5411 | K | 420.2448 | 402.2346 | 403.2183 | 210.6226 | 3 |
| 18 | 2063.1248 | 2045.1143 | 2046.0978 | 1032.0624 | E | 292.1501 | 274.1393 | 275.1233 | 146.5751 | 2 |
| 19 | | | | | K(+15.99) | 163.1076 | 145.0969 | 146.0807 | 82.0539 | 1 |

**Figure S6(D-F)**

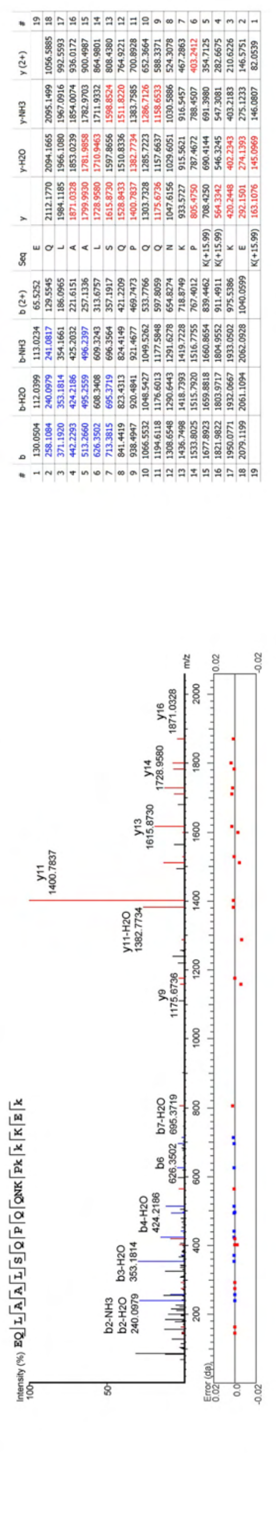

(D) BRD4(523-541): EQLAALSQPQQNKPK[O]K[O]KEK[O]; -10lgP=44.69; Scan 4694 (MC46)

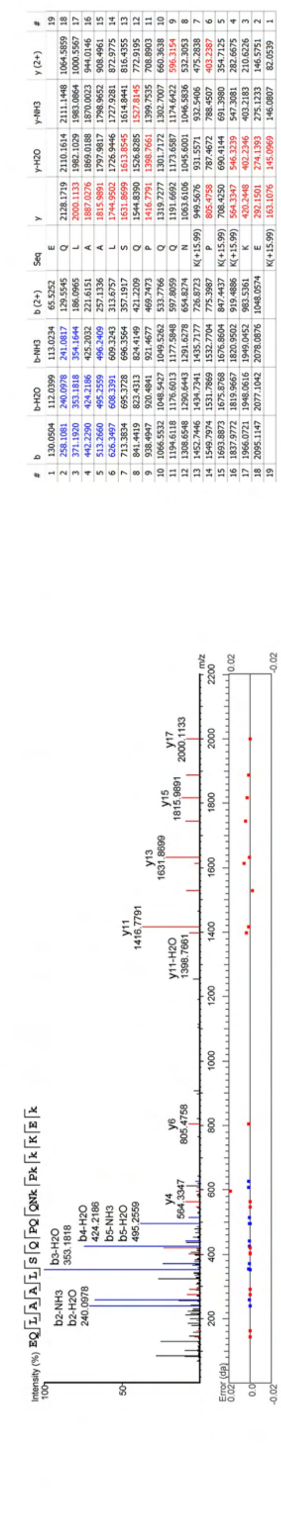

(E) BRD4(523-541): EQLAALSQPQQNK[O]PK[O]K[O]KEK[O]; -10lgP=36.26; Scan 4572 (MC46)

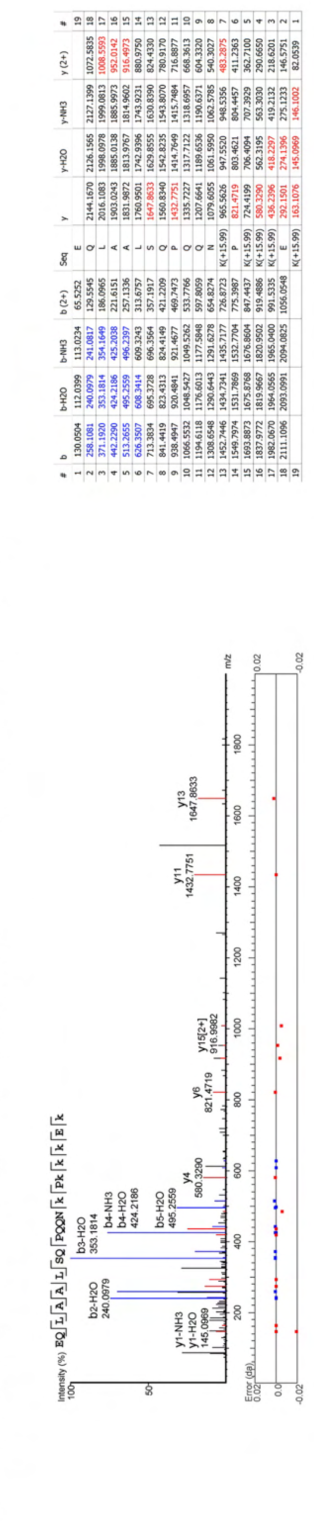

(F) BRD4(523-541): EQLAALSQPQQNK[O]PK[O]K[O]K[O]EK[O]; -10lgP=28.72; Scan 4500 (MC46)

**Figure S7**

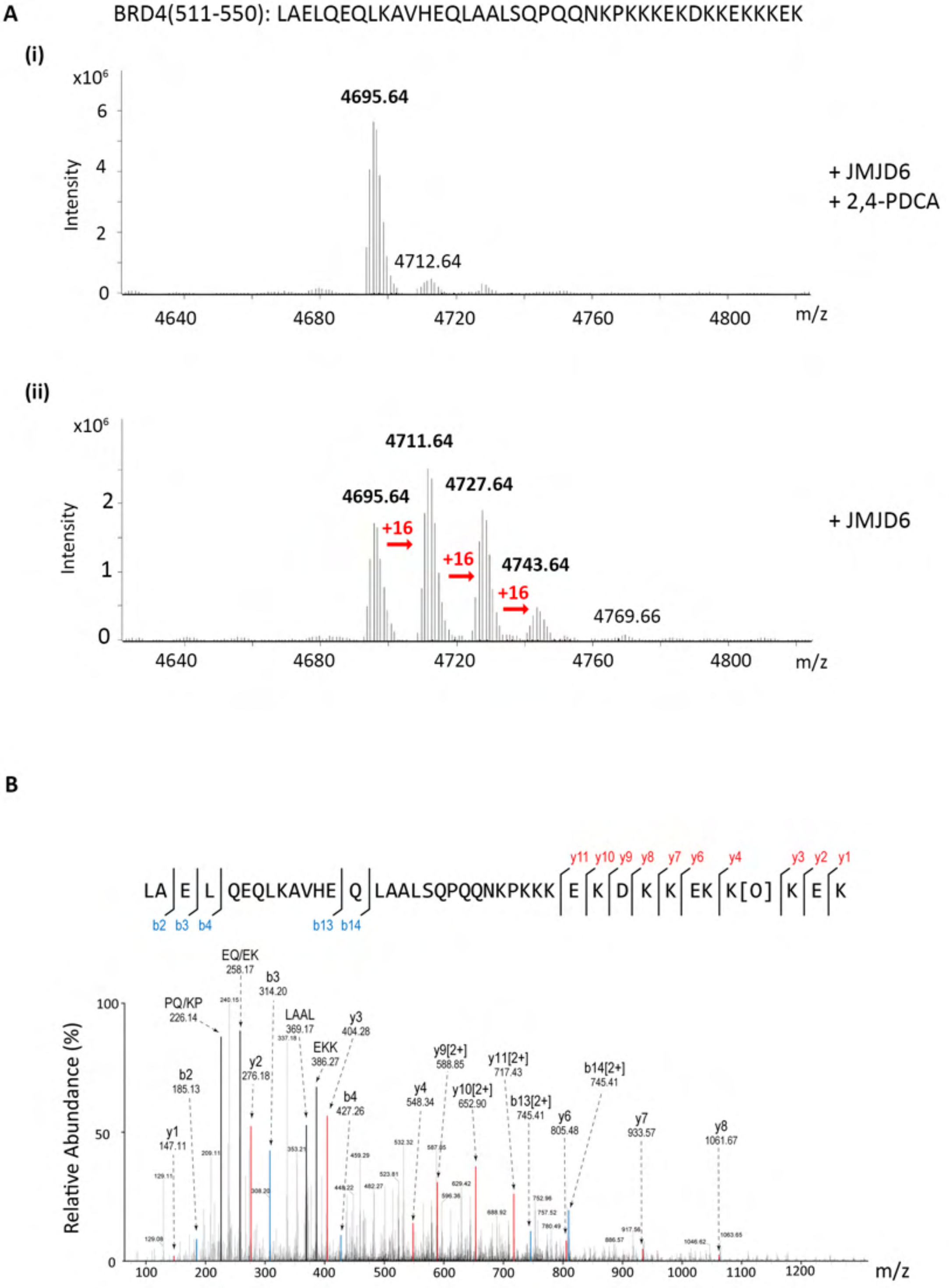
A
BRD4(511-550): LAELQEQLKAVHEQLAALSQPQQNKPKKKEKDKKEKKKEK
(i)
x10^6
Intensity
4695.64
4712.64
+ JMJD6
+ 2,4-PDCA
m/z
(ii)
4711.64
4695.64
4727.64
4743.64
4769.66
+16
+ JMJD6
B
LA E L QEQLKAVHE Q LAALSQPQQNKPKKK E K D K K EK K[O] K E K
y11 y10 y9 y8 y7 y6 y4 y3 y2 y1
b2 b3 b4 b13 b14
Relative Abundance (%)
EQ/EK 258.17
PQ/KP 226.14
b3 314.20
LAAL 369.17
y3 404.28
EKK 386.27
y2 276.18
b2 185.13
b4 427.26
y1 147.11
y9[2+] 588.85
y11[2+] 717.43
b14[2+] 745.41
y4 548.34
y10[2+] 652.90
b13[2+] 745.41
y6 805.48
y7 933.57
y8 1061.67
m/z

**Figure S8**

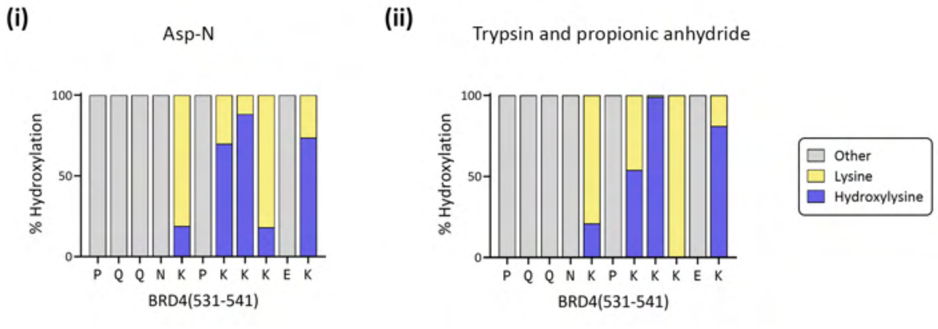

(i)
Asp-N
(ii)
Trypsin and propionic anhydride
100
50
0
% Hydroxylation
P Q Q N K P K K K E K
BRD4(531-541)
Other
Lysine
Hydroxylysine

**Figure S9**

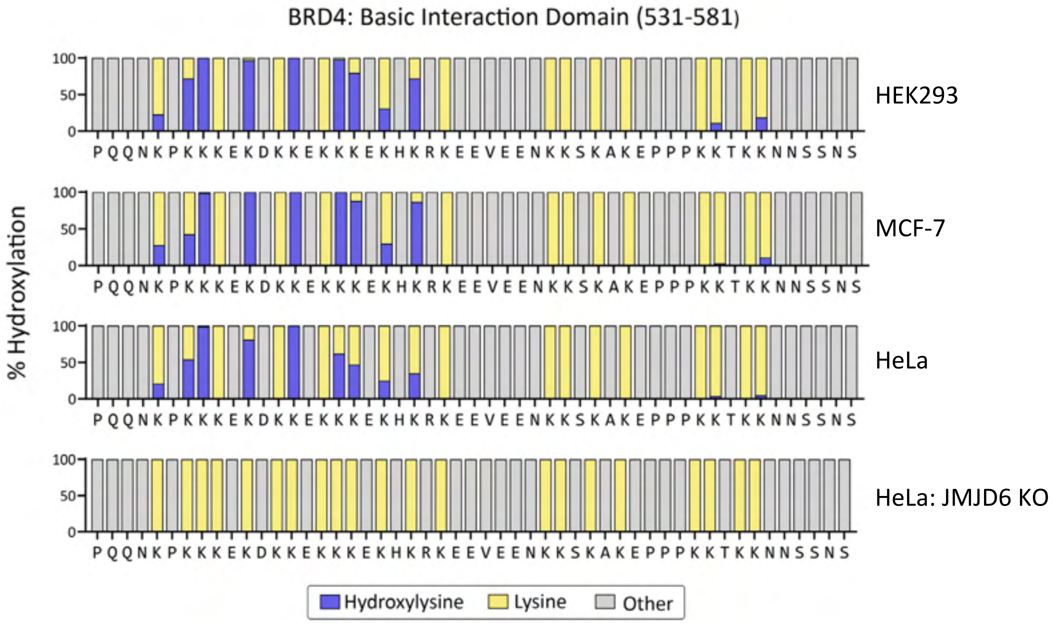
BRD4: Basic Interaction Domain (531-581)
% Hydroxylation
100
50
0
P Q Q N K P K K K E K D K K E K K K E K H K R K E E V E E N K K S K A K E P P P K K T K K N N S S N S
HEK293
MCF-7
HeLa
HeLa: JMJD6 KO
Hydroxylysine
Lysine
Other

**Figure S10**

**BRD4(547-553): K*[O]K*[O]EK*[O]HK*[O]R;** -10lgP=15.11; Scan 5469 (MC304)

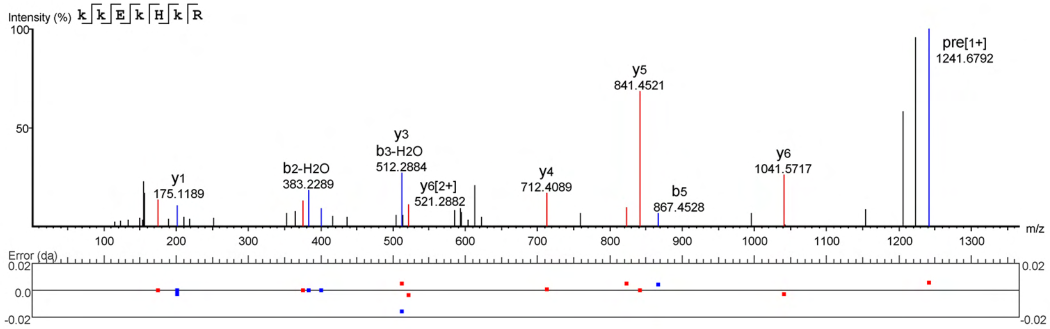


| # | b | b-H2O | b-NH3 | b (2+) | Seq | y | y-H2O | y-NH3 | y (2+) | # |
|---|---|---|---|---|---|---|---|---|---|---|
| 1 | 201.1232 | 183.1134 | 184.0969 | 101.0620 | K(+72.02) | | | | | 7 |
| 2 | 401.2399 | 383.2289 | 384.2130 | 201.1232 | K(+72.02) | 1041.5717 | 1023.5581 | 1024.5417 | 521.2882 | 6 |
| 3 | 530.2826 | 512.2884 | 513.2556 | 265.6413 | E | 841.4521 | 823.4368 | 824.4257 | 421.2263 | 5 |
| 4 | 730.3987 | 712.3881 | 713.3717 | 365.6993 | K(+72.02) | 712.4089 | 694.3995 | 695.3831 | 356.7050 | 4 |
| 5 | 867.4528 | 849.4470 | 850.4306 | 434.2288 | H | 512.2884 | 494.2834 | 495.2670 | 256.6470 | 3 |
| 6 | 1067.5737 | 1049.5632 | 1050.5468 | 534.2869 | K(+72.02) | 375.2346 | 357.2245 | 358.2080 | 188.1175 | 2 |
| 7 | | | | | R | 175.1189 | 157.1084 | 158.0919 | 88.0595 | 1 |

**Figure S11**

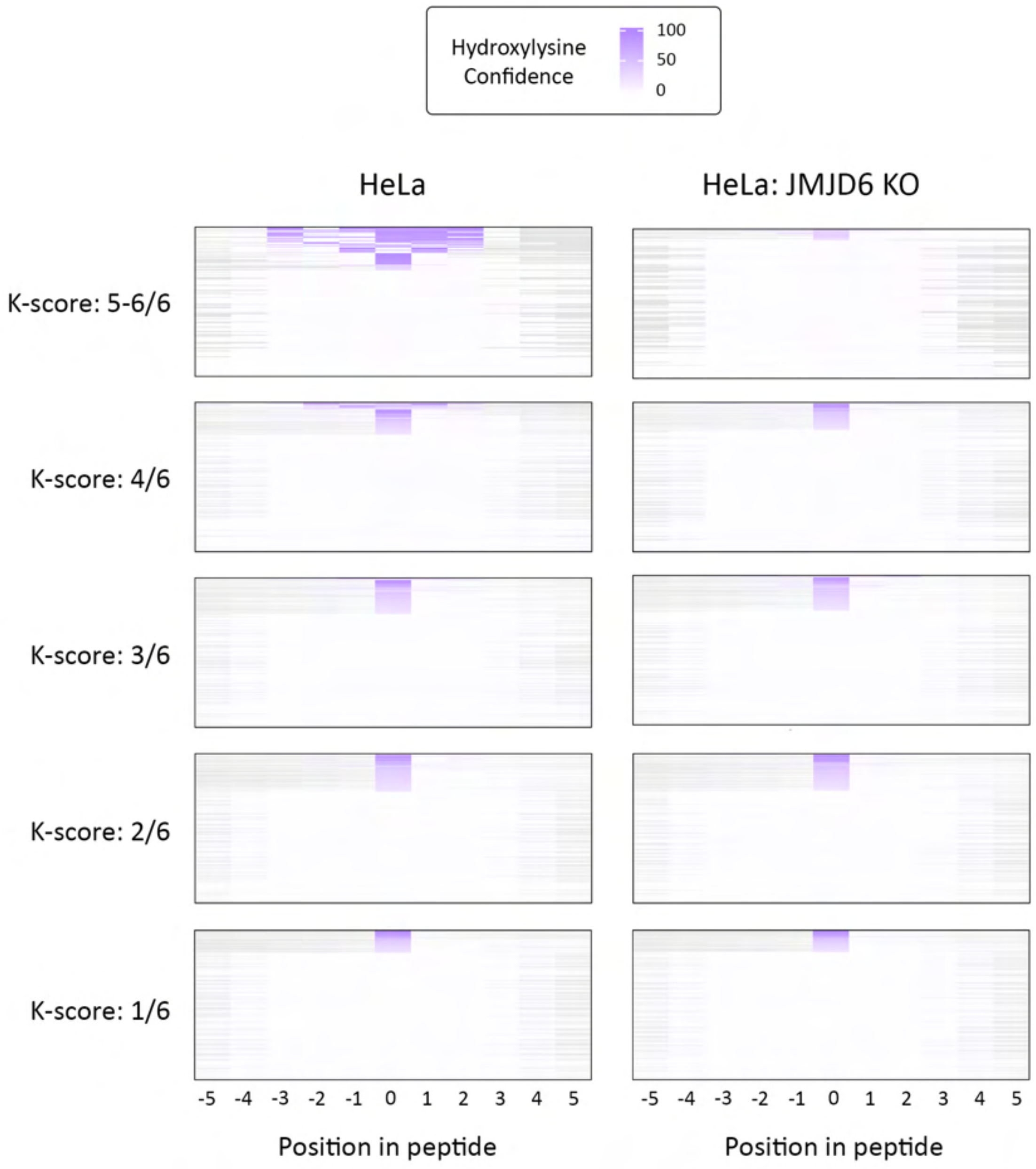
Hydroxylysine
Confidence
100
50
0
HeLa
HeLa: JMJD6 KO
K-score: 5-6/6
K-score: 4/6
K-score: 3/6
K-score: 2/6
K-score: 1/6
-5 -4 -3 -2 -1 0 1 2 3 4 5
Position in peptide
-5 -4 -3 -2 -1 0 1 2 3 4 5
Position in peptide

**Figure S12**

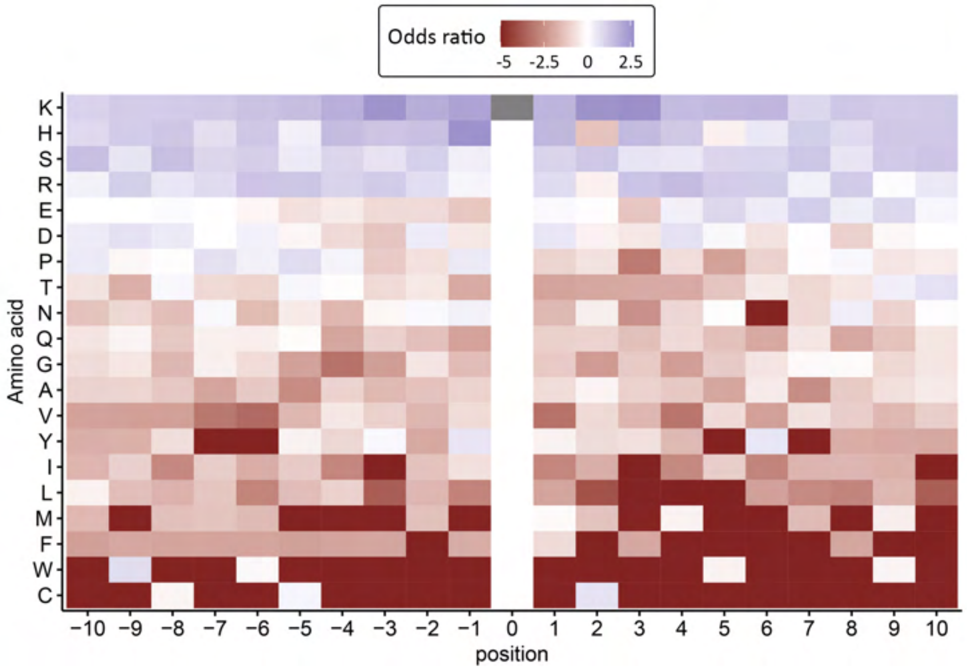

Odds ratio
-5
-2.5
0
2.5
K
H
S
R
E
D
P
T
N
Q
G
A
V
Y
I
L
M
F
W
C
Amino acid
−10
−9
−8
−7
−6
−5
−4
−3
−2
−1
0
1
2
3
4
5
6
7
8
9
10
position

**Figure S13**

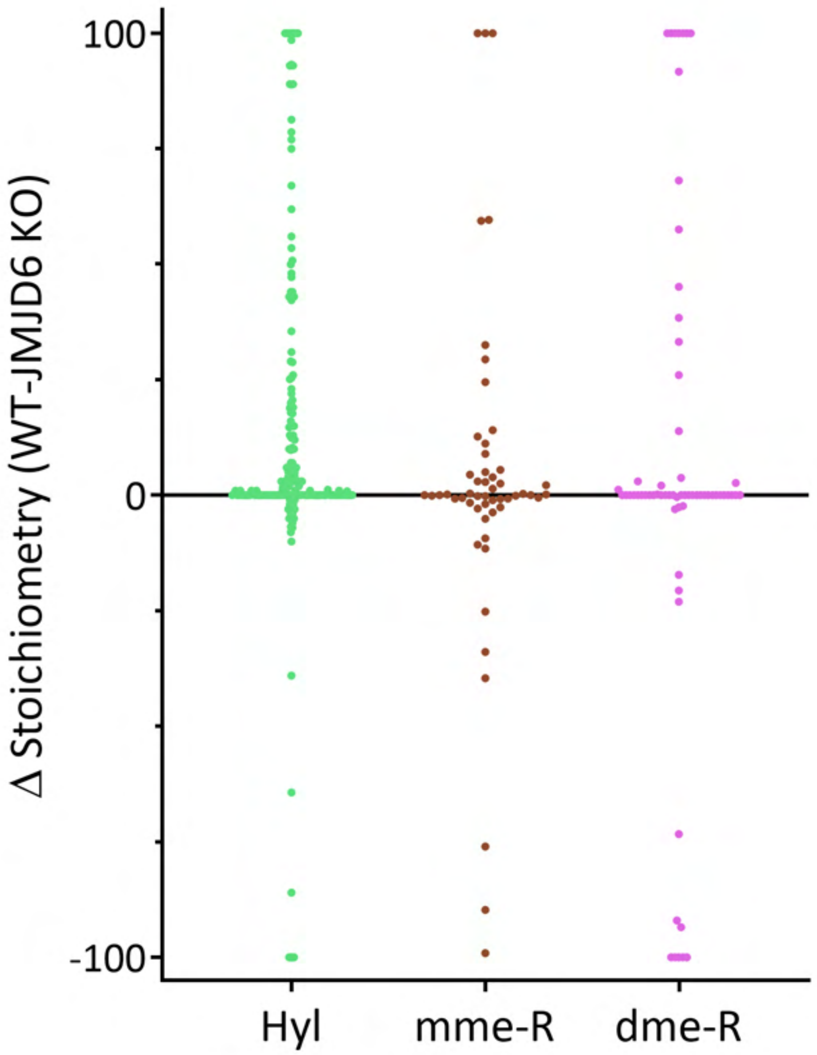

**Figure S14_1**

**MSMS assignment of K739ox in AP-3 complex subunit delta-1**

AP3D1(735-742): QK*LEK*[O]DK*R; -10lgP 26.50; Scan 10727 (MC303)

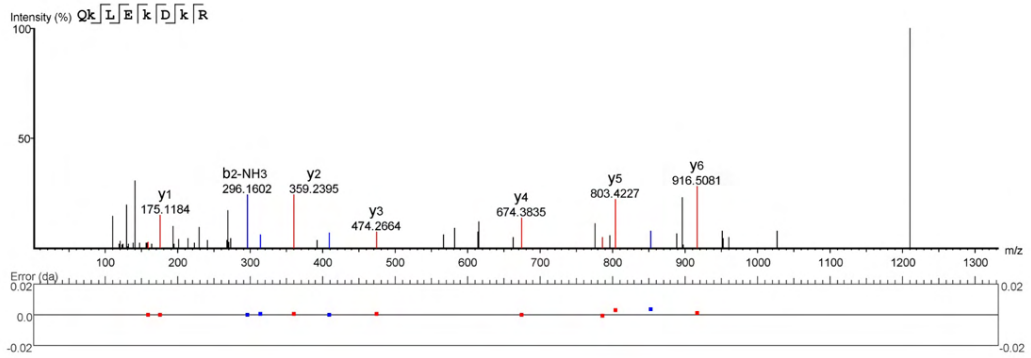


| # | b | b-H2O | b-NH3 | b (2+) | Seq | y | y-H2O | y-NH3 | y (2+) | # |
|---|---|---|---|---|---|---|---|---|---|---|
| 1 | 129.0664 | 111.0558 | 112.0394 | 65.0332 | Q | | | | | 8 |
| 2 | 313.1865 | 295.1770 | 296.1602 | 157.0938 | K(+56.03) | 1100.6310 | 1082.6205 | 1083.6040 | 550.8155 | 7 |
| 3 | 426.2716 | 408.2610 | 409.2445 | 213.6358 | L | 916.5081 | 898.4992 | 899.4828 | 458.7549 | 6 |
| 4 | 555.3142 | 537.3036 | 538.2872 | 278.1571 | E | 803.4227 | 785.4158 | 786.3987 | 402.2129 | 5 |
| 5 | 755.4303 | 737.4197 | 738.4033 | 378.2151 | K(+72.02) | 674.3835 | 656.3726 | 657.3561 | 337.6916 | 4 |
| 6 | 870.4572 | 852.4429 | 853.4302 | 435.7286 | D | 474.2664 | 456.2565 | 457.2401 | 237.6335 | 3 |
| 7 | 1054.5784 | 1036.5679 | 1037.5514 | 527.7892 | K(+56.03) | 359.2395 | 341.2296 | 342.2131 | 180.1201 | 2 |
| 8 | | | | | R | 175.1184 | 157.1084 | 158.0924 | 88.0595 | 1 |

Figure S14_2

**MSMS assignment of K749ox, K752ox in AP-3 complex subunit delta-1**

AP3D1(747-755): K*EK*[O]EK*K[O]GK*R; -10lgP 20.67; Scan 10022 (MC303)

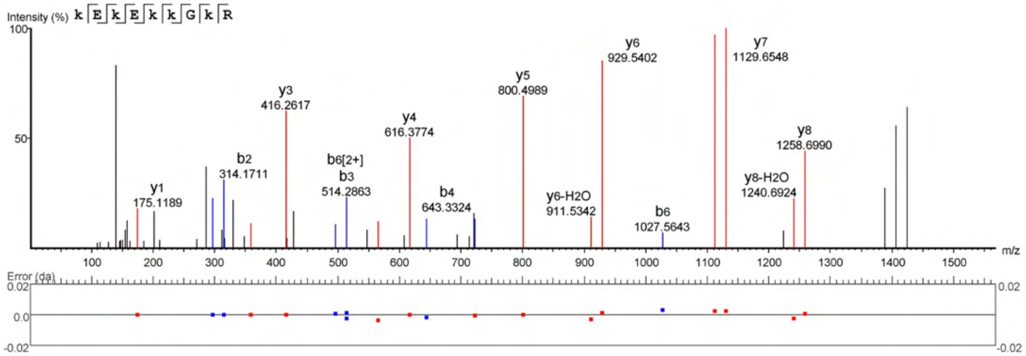


| # | b | b-H2O | b-NH3 | b (2+) | Seq | y | y-H2O | y-NH3 | y (2+) | # |
|---|---|---|---|---|---|---|---|---|---|---|
| 1 | 185.1290 | 167.1184 | 168.1020 | 93.0645 | K(+56.03) | | | | | 9 |
| 2 | 314.1711 | 296.1609 | 297.1446 | 157.5858 | E | 1258.6990 | 1240.6924 | 1241.6731 | 629.8500 | 8 |
| 3 | 514.2863 | 496.2761 | 497.2607 | 257.6438 | K(+72.02) | 1129.6548 | 1111.6444 | 1112.6305 | 565.3329 | 7 |
| 4 | 643.3324 | 625.3197 | 626.3033 | 322.1651 | E | 929.5402 | 911.5342 | 912.5144 | 465.2707 | 6 |
| 5 | 827.4514 | 809.4409 | 810.4244 | 414.2257 | K(+56.03) | 800.4989 | 782.4883 | 783.4719 | 400.7494 | 5 |
| 6 | 1027.5643 | 1009.5569 | 1010.5405 | 514.2863 | K(+72.02) | 616.3774 | 598.3671 | 599.3506 | 308.6888 | 4 |
| 7 | 1084.5890 | 1066.5785 | 1067.5620 | 542.7945 | G | 416.2617 | 398.2510 | 399.2346 | 208.6308 | 3 |
| 8 | 1268.7101 | 1250.6996 | 1251.6831 | 634.8550 | K(+56.03) | 359.2404 | 341.2296 | 342.2131 | 180.1201 | 2 |
| 9 | | | | | R | 175.1189 | 157.1084 | 158.0919 | 88.0595 | 1 |

Figure S14_3

**MSMS assignment of K13ox, K16ox, K19ox, K22ox, K23ox in Arginine and glutamate-rich protein 1**

ARGLU1(12-24): SK*[O]HTK*[O]SSK*[O]HNK*[O]K*[O]R; -10lgP 56.55; Scan 4848 (MC309)

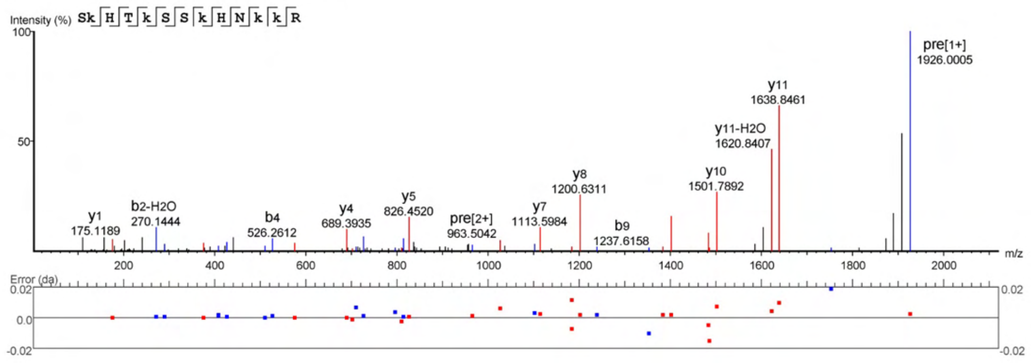


| # | b | b-H2O | b-NH3 | b (2+) | Seq | y | y-H2O | y-NH3 | y (2+) | # |
|---|---|---|---|---|---|---|---|---|---|---|
| 1 | 88.0399 | 70.0293 | 71.0129 | 44.5199 | S | | | | | 13 |
| 2 | 288.1550 | 270.1444 | 271.1290 | 144.5780 | K(+72.02) | 1838.9719 | 1820.9614 | 1821.9449 | 919.9860 | 12 |
| 3 | 425.2141 | 407.2029 | 408.1879 | 213.1074 | H | 1638.8461 | 1620.8407 | 1621.8289 | 819.9279 | 11 |
| 4 | 526.2612 | 508.2516 | 509.2355 | 263.6313 | T | 1501.7892 | 1483.7913 | 1484.7852 | 751.3984 | 10 |
| 5 | 726.3773 | 708.3613 | 709.3516 | 363.6893 | K(+72.02) | 1400.7468 | 1382.7368 | 1383.7222 | 700.8763 | 9 |
| 6 | 813.4095 | 795.3960 | 796.3837 | 407.2029 | S | 1200.6311 | 1182.6106 | 1183.6136 | 600.8165 | 8 |
| 7 | 900.4427 | 882.4321 | 883.4157 | 450.7213 | S | 1113.5984 | 1095.5906 | 1096.5741 | 557.3005 | 7 |
| 8 | 1100.5554 | 1082.5482 | 1083.5317 | 550.7794 | K(+72.02) | 1026.5625 | 1008.5585 | 1009.5421 | 513.7845 | 6 |
| 9 | 1237.6158 | 1219.6072 | 1220.5907 | 619.3088 | H | 826.4520 | 808.4424 | 809.4285 | 413.7265 | 5 |
| 10 | 1351.6713 | 1333.6501 | 1334.6337 | 676.3303 | N | 689.3935 | 671.3835 | 672.3671 | 345.1970 | 4 |
| 11 | 1551.7767 | 1533.7662 | 1534.7498 | 776.3884 | K(+72.02) | 575.3508 | 557.3405 | 558.3241 | 288.1755 | 3 |
| 12 | 1751.8737 | 1733.8823 | 1734.8658 | 876.4464 | K(+72.02) | 375.2349 | 357.2245 | 358.2080 | 188.1175 | 2 |
| 13 | | | | | R | 175.1189 | 157.1084 | 158.0919 | 88.0595 | 1 |

Figure S14_4

**MSMS assignment of K290ox and K292ox in ADP-ribosylation factor-like protein 6-interacting protein 4**

ARL6IP4(289-295): GK*[O]YK*[O]DK*R; 10lgP 27.45; Scan 6783 (MC319)

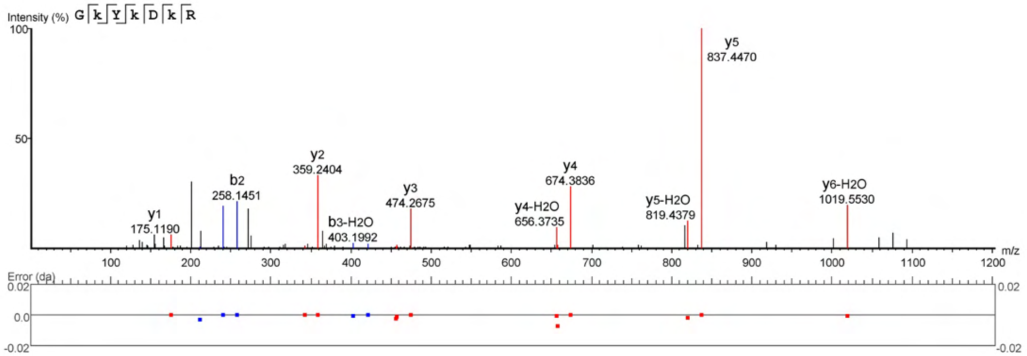


| # | b | b-H2O | b-NH3 | b (2+) | Seq | y | y-H2O | y-NH3 | y (2+) | # |
|---|---|---|---|---|---|---|---|---|---|---|
| 1 | 58.0293 | 40.0187 | 41.0023 | 29.5146 | G | | | | | 7 |
| 2 | 258.1451 | 240.1346 | 241.1184 | 129.5727 | K(+72.02) | 1037.5626 | 1019.5530 | 1020.5356 | 519.2813 | 6 |
| 3 | 421.2086 | 403.1992 | 404.1817 | 211.1075 | Y | 837.4470 | 819.4379 | 820.4195 | 419.2232 | 5 |
| 4 | 621.3248 | 603.3142 | 604.2978 | 311.1624 | K(+72.02) | 674.3836 | 656.3735 | 657.3640 | 337.6916 | 4 |
| 5 | 736.3517 | 718.3411 | 719.3247 | 368.6758 | D | 474.2675 | 456.2593 | 457.2413 | 237.6335 | 3 |
| 6 | 920.4729 | 902.4623 | 903.4459 | 460.7365 | K(+56.03) | 359.2404 | 341.2296 | 342.2131 | 180.1201 | 2 |
| 7 | | | | | R | 175.1190 | 157.1084 | 158.0919 | 88.0595 | 1 |

**Figure S14_5**

**MSMS assignment of K294ox in ADP-ribosylation factor-like protein 6-interacting protein 4**

ARL6IP4(289-295): GK*[O]YK*[O]DK*[O]R; -10lgP 17.62; Scan 6784 (MC312)

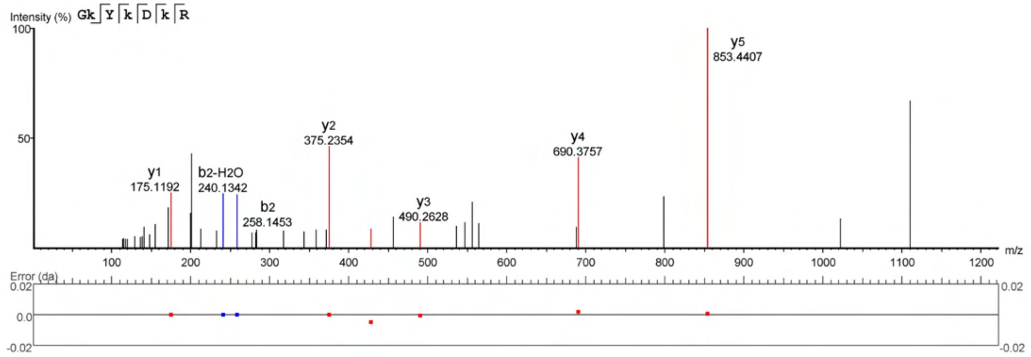


| # | b | b-H2O | b-NH3 | b (2+) | Seq | y | y-H2O | y-NH3 | y (2+) | # |
|---|---|---|---|---|---|---|---|---|---|---|
| 1 | 58.0293 | 40.0187 | 41.0023 | 29.5146 | G | | | | | 7 |
| 2 | 258.1453 | 240.1342 | 241.1184 | 129.5727 | K(+72.02) | 1053.5575 | 1035.5470 | 1036.5305 | 527.2787 | 6 |
| 3 | 421.2087 | 403.1981 | 404.1817 | 211.1043 | Y | 853.4407 | 835.4308 | 836.4144 | 427.2255 | 5 |
| 4 | 621.3248 | 603.3142 | 604.2978 | 311.1624 | K(+72.02) | 690.3757 | 672.3675 | 673.3511 | 345.6890 | 4 |
| 5 | 736.3517 | 718.3411 | 719.3247 | 368.6758 | D | 490.2628 | 472.2514 | 473.2350 | 245.6310 | 3 |
| 6 | 936.4678 | 918.4572 | 919.4408 | 468.7339 | K(+72.02) | 375.2354 | 357.2245 | 358.2080 | 188.1175 | 2 |
| 7 | | | | | R | 175.1192 | 157.1084 | 158.0919 | 88.0595 | 1 |

Figure S14_6

**MSMS assignment of K1422ox in Transcriptional regulator ATRX**

ATRX(1418-1425): SYK*QK*[O]K*K*R; -10lgP 20.23; Scan 17325 (MC356)

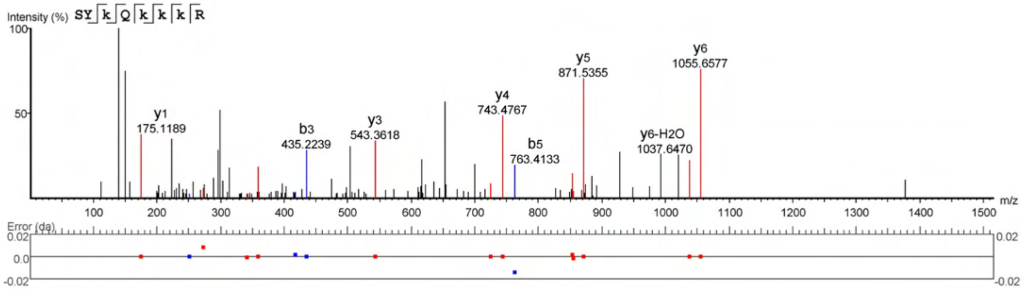


| # | b | b-H2O | b-NH3 | b (2+) | Seq | y | y-H2O | y-NH3 | y (2+) | # |
|---|---|---|---|---|---|---|---|---|---|---|
| 1 | 88.0399 | 70.0293 | 71.0129 | 44.5199 | S | | | | | 8 |
| 2 | 251.1031 | 233.0926 | 234.0762 | 126.0516 | Y | 1218.7205 | 1200.7100 | 1201.6935 | 609.8602 | 7 |
| 3 | 435.2239 | 417.2120 | 418.1974 | 218.1122 | K(+56.03) | 1055.6577 | 1037.6470 | 1038.6301 | 528.3286 | 6 |
| 4 | 563.2829 | 545.2723 | 546.2559 | 282.1414 | Q | 871.5355 | 853.5229 | 854.5114 | 436.2680 | 5 |
| 5 | 763.4133 | 745.3884 | 746.3720 | 382.1995 | K(+72.02) | 743.4767 | 725.4666 | 726.4504 | 372.2387 | 4 |
| 6 | 947.5201 | 929.5096 | 930.4932 | 474.2601 | K(+56.03) | 543.3618 | 525.3507 | 526.3343 | 272.1721 | 3 |
| 7 | 1131.6414 | 1113.6309 | 1114.6144 | 566.3207 | K(+56.03) | 359.2404 | 341.2296 | 342.2140 | 180.1201 | 2 |
| 8 | | | | | R | 175.1189 | 157.1084 | 158.0919 | 88.0595 | 1 |

**Figure S14_7**

**MSMS assignment of K1424ox in Transcriptional regulator ATRX**

ATRX(1418-1425): SYK*QK*K*K*[O]R; -10lgP 21.81; Scan 16964 (MC355)

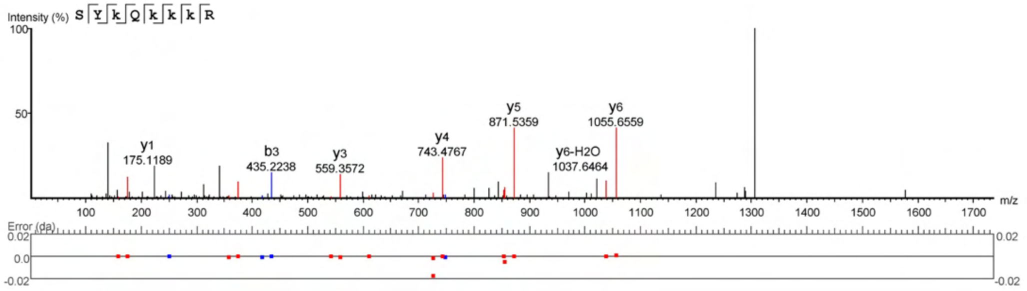


| # | b | b-H2O | b-NH3 | b (2+) | Seq | y | y-H2O | y-NH3 | y (2+) | # |
|---|---|---|---|---|---|---|---|---|---|---|
| 1 | 88.0399 | 70.0293 | 71.0129 | 44.5199 | S | | | | | 8 |
| 2 | 251.1025 | 233.0926 | 234.0762 | 126.0516 | Y | 1218.7205 | 1200.7100 | 1201.6935 | 609.8606 | 7 |
| 3 | 435.2238 | 417.2152 | 418.1974 | 218.1122 | K(+56.03) | 1055.6559 | 1037.6464 | 1038.6301 | 528.3286 | 6 |
| 4 | 563.2829 | 545.2723 | 546.2559 | 282.1414 | Q | 871.5359 | 853.5251 | 854.5140 | 436.2680 | 5 |
| 5 | 747.4056 | 729.3935 | 730.3771 | 374.2020 | K(+56.03) | 743.4767 | 725.4690 | 726.4680 | 372.2387 | 4 |
| 6 | 931.5253 | 913.5147 | 914.4983 | 466.2626 | K(+56.03) | 559.3572 | 541.3456 | 542.3292 | 280.1781 | 3 |
| 7 | 1131.6414 | 1113.6309 | 1114.6144 | 566.3207 | K(+72.02) | 375.2352 | 357.2257 | 358.2080 | 188.1175 | 2 |
| 8 | | | | | R | 175.1189 | 157.1084 | 158.0927 | 88.0595 | 1 |

**Figure S14_8**

## MSMS assignment of K546ox in Bromodomain-containing protein 2

BRD2(529-547): AVHEQLAALSQGPISK*PK*[O]R; -10lgP 84.85; Scan 24877 (MC275)

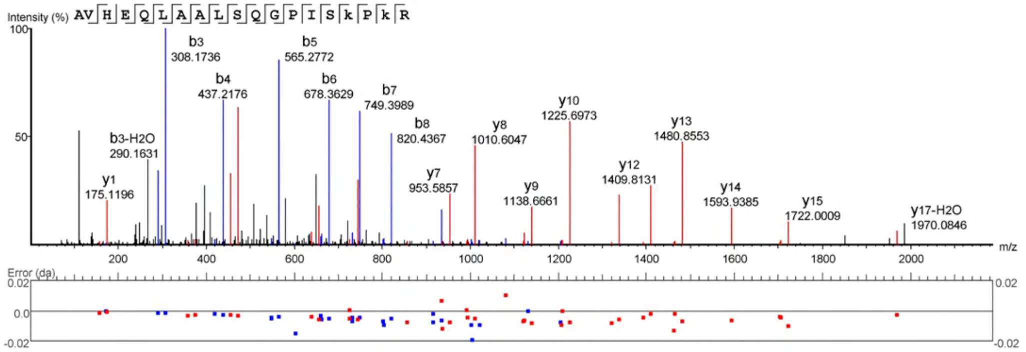


| # | b | b-H2O | b-NH3 | b (2+) | Seq | y | y-H2O | y-NH3 | y (2+) | # |
|---|---|---|---|---|---|---|---|---|---|---|
| 1 | 72.0449 | 54.0344 | 55.0179 | 36.5225 | A | | | | | 19 |
| 2 | 171.1134 | 153.1028 | 154.0863 | 86.0567 | V | 2087.1606 | 2069.1501 | 2070.1335 | 1044.0803 | 18 |
| 3 | 308.1736 | 290.1631 | 291.1453 | 154.5861 | H | 1988.0923 | 1970.0846 | 1971.0653 | 994.5461 | 17 |
| 4 | 437.2176 | 419.2066 | 420.1879 | 219.1074 | E | 1851.0333 | 1833.0228 | 1834.0063 | 926.0167 | 16 |
| 5 | 565.2772 | 547.2675 | 548.2519 | 283.1367 | Q | 1722.0009 | 1703.9841 | 1704.9686 | 861.4954 | 15 |
| 6 | 678.3629 | 660.3503 | 661.3364 | 339.6787 | L | 1593.9385 | 1575.9218 | 1576.9053 | 797.4661 | 14 |
| 7 | 749.3989 | 731.3885 | 732.3748 | 375.1973 | A | 1480.8553 | 1462.8506 | 1463.8235 | 740.9241 | 13 |
| 8 | 820.4367 | 802.4283 | 803.4141 | 410.7159 | A | 1409.8131 | 1391.8005 | 1392.7886 | 705.4055 | 12 |
| 9 | 933.5220 | 915.5074 | 916.4963 | 467.2579 | L | 1338.7798 | 1320.7634 | 1321.7551 | 669.8870 | 11 |
| 10 | 1020.5575 | 1002.5466 | 1003.5403 | 510.7739 | S | 1225.6973 | 1207.6890 | 1208.6630 | 613.3449 | 10 |
| 11 | 1148.6063 | 1130.5953 | 1131.5793 | 574.8032 | Q | 1138.6661 | 1120.6547 | 1121.6375 | 569.8289 | 9 |
| 12 | 1205.6356 | 1187.6173 | 1188.6008 | 603.3288 | G | 1010.6047 | 992.5878 | 993.5769 | 505.7996 | 8 |
| 13 | 1302.6805 | 1284.6700 | 1285.6536 | 651.8403 | P | 953.5857 | 935.5600 | 936.5630 | 477.2889 | 7 |
| 14 | 1415.7646 | 1397.7542 | 1398.7377 | 708.3823 | I | 856.5325 | 838.5145 | 839.4980 | 428.7625 | 6 |
| 15 | 1502.7966 | 1484.7861 | 1485.7697 | 751.8983 | S | 743.4465 | 725.4292 | 726.4191 | 372.2205 | 5 |
| 16 | 1686.9178 | 1668.9073 | 1669.8909 | 843.9589 | K(+56.03) | 656.4146 | 638.3984 | 639.3858 | 328.7045 | 4 |
| 17 | 1783.9706 | 1765.9601 | 1766.9436 | 892.4853 | P | 472.2911 | 454.2772 | 455.2638 | 236.6439 | 3 |
| 18 | 1984.0867 | 1966.0762 | 1967.0597 | 992.5433 | K(+72.02) | 375.2378 | 357.2245 | 358.2117 | 188.1175 | 2 |
| 19 | | | | | R | 175.1196 | 157.1084 | 158.0932 | 88.0595 | 1 |

**Figure S14_9**

**MSMS assignment of K586ox in Bromodomain-containing protein 2**

BRD2(578-588): APRPPQPK*K*[O]SK; -10lgP 53.56; Scan 3410 (MC277)

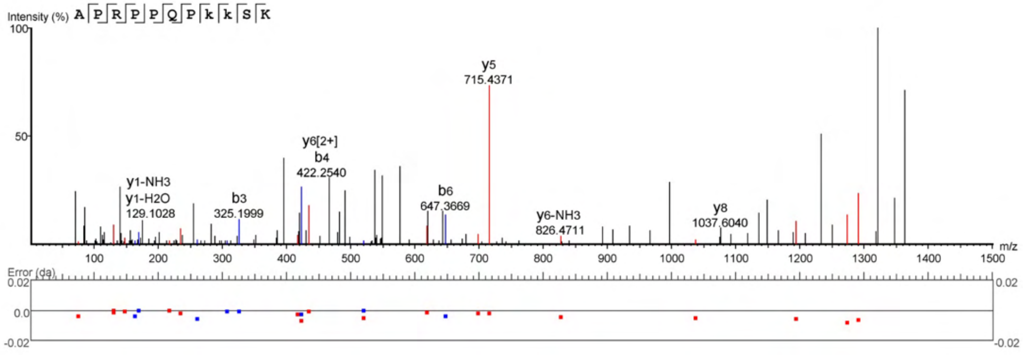


| # | b | b-H2O | b-NH3 | b (2+) | Seq | y | y-H2O | y-NH3 | y (2+) | # |
|---|---|---|---|---|---|---|---|---|---|---|
| 1 | 72.0449 | 54.0344 | 55.0179 | 36.5225 | A | | | | | 11 |
| 2 | 169.0978 | 151.0871 | 152.0707 | 85.0489 | P | 1290.7594 | 1272.7423 | 1273.7344 | 645.8764 | 10 |
| 3 | 325.1999 | 307.1892 | 308.1718 | 163.1037 | R | 1193.7058 | 1175.6896 | 1176.6731 | 597.3500 | 9 |
| 4 | 422.2540 | 404.2410 | 405.2245 | 211.6258 | P | 1037.6040 | 1019.5884 | 1020.5720 | 519.3046 | 8 |
| 5 | 519.3046 | 501.2938 | 502.2773 | 260.1579 | P | 940.5461 | 922.5356 | 923.5192 | 470.7731 | 7 |
| 6 | 647.3669 | 629.3523 | 630.3359 | 324.1814 | Q | 843.4934 | 825.4828 | 826.4711 | 422.2540 | 6 |
| 7 | 744.4156 | 726.4051 | 727.3887 | 372.7078 | P | 715.4371 | 697.4265 | 698.4078 | 358.2174 | 5 |
| 8 | 928.5368 | 910.5262 | 911.5098 | 464.7684 | K(+56.03) | 618.3834 | 600.3715 | 601.3551 | 309.6910 | 4 |
| 9 | 1128.6528 | 1110.6423 | 1111.6259 | 564.8264 | K(+72.02) | 434.2621 | 416.2531 | 417.2339 | 217.6304 | 3 |
| 10 | 1215.6849 | 1197.6744 | 1198.6580 | 608.3425 | S | 234.1470 | 216.1347 | 217.1178 | 117.5724 | 2 |
| 11 | | | | | K | 147.1137 | 129.1028 | 130.0871 | 74.0602 | 1 |

Figure S14_10

## MSMS assignment of K589ox in Bromodomain-containing protein 2

BRD2(589-614): K*[O]ASGSGGGSAALGPSGFGPSGGSGTK; -10lgP 57.22; Scan 14997 (MC271)

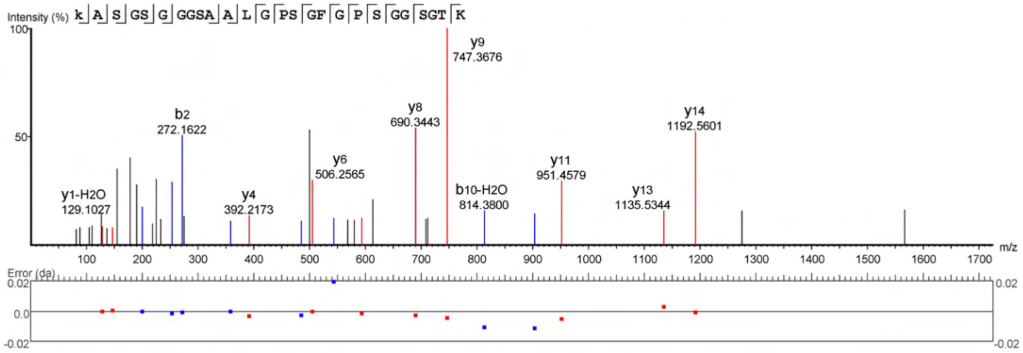


| # | b | b-H2O | b-NH3 | b (2+) | Seq | y | y-H2O | y-NH3 | y (2+) | # |
|---|---|---|---|---|---|---|---|---|---|---|
| 1 | 201.1243 | 183.1134 | 184.0969 | 101.0620 | K(+72.02) | | | | | 26 |
| 2 | 272.1622 | 254.1519 | 255.1340 | 136.5805 | A | 2007.9365 | 1989.9260 | 1990.9095 | 1004.4683 | 25 |
| 3 | 359.1927 | 341.1825 | 342.1661 | 180.0965 | S | 1936.8994 | 1918.8889 | 1919.8724 | 968.9497 | 24 |
| 4 | 416.2145 | 398.2039 | 399.1875 | 208.6073 | G | 1849.8674 | 1831.8569 | 1832.8405 | 925.4337 | 23 |
| 5 | 503.2465 | 485.2386 | 486.2195 | 252.1233 | S | 1792.8459 | 1774.8354 | 1775.8190 | 896.9230 | 22 |
| 6 | 560.2680 | 542.2574 | 543.2211 | 280.6340 | G | 1705.8140 | 1687.8035 | 1688.7870 | 853.4070 | 21 |
| 7 | 617.2894 | 599.2789 | 600.2625 | 309.1447 | G | 1648.7925 | 1630.7820 | 1631.7655 | 824.8962 | 20 |
| 8 | 674.3109 | 656.3004 | 657.2839 | 337.6555 | G | 1591.7710 | 1573.7605 | 1574.7440 | 796.3855 | 19 |
| 9 | 761.3430 | 743.3324 | 744.3160 | 381.1715 | S | 1534.7495 | 1516.7390 | 1517.7225 | 767.8748 | 18 |
| 10 | 832.3801 | 814.3800 | 815.3531 | 416.6900 | A | 1447.7175 | 1429.7070 | 1430.6906 | 724.3588 | 17 |
| 11 | 903.4285 | 885.4066 | 886.3902 | 452.2086 | A | 1376.6804 | 1358.6699 | 1359.6534 | 688.8402 | 16 |
| 12 | 1016.5012 | 998.4907 | 999.4742 | 508.7506 | L | 1305.6433 | 1287.6328 | 1288.6163 | 653.3217 | 15 |
| 13 | 1073.5227 | 1055.5122 | 1056.4957 | 537.2614 | G | 1192.5601 | 1174.5487 | 1175.5322 | 596.7796 | 14 |
| 14 | 1170.5754 | 1152.5649 | 1153.5485 | 585.7877 | P | 1135.5344 | 1117.5272 | 1118.5107 | 568.2689 | 13 |
| 15 | 1257.6074 | 1239.5969 | 1240.5804 | 629.3037 | S | 1038.4850 | 1020.4744 | 1021.4580 | 519.7425 | 12 |
| 16 | 1314.6289 | 1296.6184 | 1297.6019 | 657.8145 | G | 951.4579 | 933.4424 | 934.4260 | 476.2265 | 11 |
| 17 | 1461.6974 | 1443.6869 | 1444.6704 | 731.3487 | F | 894.4315 | 876.4210 | 877.4045 | 447.7158 | 10 |
| 18 | 1518.7188 | 1500.7083 | 1501.6918 | 759.8594 | G | 747.3676 | 729.3525 | 730.3361 | 374.1815 | 9 |
| 19 | 1615.7716 | 1597.7611 | 1598.7446 | 808.3858 | P | 690.3443 | 672.3311 | 673.3147 | 345.6708 | 8 |
| 20 | 1702.8036 | 1684.7931 | 1685.7766 | 851.9018 | S | 593.2904 | 575.2783 | 576.2619 | 297.1444 | 7 |
| 21 | 1759.8251 | 1741.8146 | 1742.7981 | 880.4125 | G | 506.2565 | 488.2463 | 489.2299 | 253.6284 | 6 |
| 22 | 1816.8466 | 1798.8361 | 1799.8196 | 908.9233 | G | 449.2354 | 431.2249 | 432.2084 | 225.1177 | 5 |
| 23 | 1903.8785 | 1885.8680 | 1886.8516 | 952.4393 | S | 392.2173 | 374.2034 | 375.1870 | 196.6070 | 4 |
| 24 | 1960.9000 | 1942.8895 | 1943.8730 | 980.9500 | G | 305.1819 | 287.1714 | 288.1549 | 153.0910 | 3 |
| 25 | 2061.9478 | 2043.9373 | 2044.9208 | 1031.4739 | T | 248.1605 | 230.1499 | 231.1335 | 124.5802 | 2 |
| 26 | | | | | K | 147.1121 | 129.1027 | 130.0858 | 74.0564 | 1 |

**Figure S14_11**

**MSMS assignment of K755ox in Bromodomain-containing protein 2**

BRD2(740-756): LQDVSGQLNSTK*KPPK*[O]K; -10lgP 52.01; Scan 12987 (MC271)

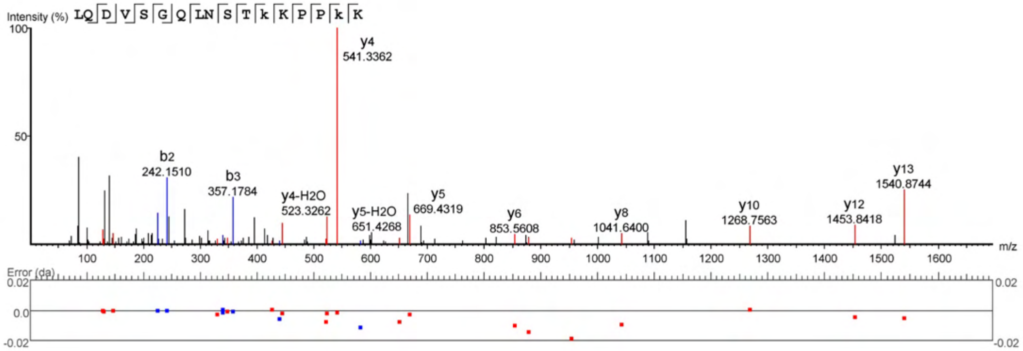


| # | b | b-H2O | b-NH3 | b (2+) | Seq | y | y-H2O | y-NH3 | y (2+) | # |
|---|---|---|---|---|---|---|---|---|---|---|
| 1 | 114.0919 | 96.0813 | 97.0649 | 57.5459 | L | | | | | 17 |
| 2 | 242.1510 | 224.1399 | 225.1240 | 121.5752 | Q | 1883.0232 | 1865.0127 | 1865.9962 | 942.0116 | 16 |
| 3 | 357.1784 | 339.1660 | 340.1518 | 179.0887 | D | 1754.9646 | 1736.9541 | 1737.9376 | 877.9968 | 15 |
| 4 | 456.2458 | 438.2352 | 439.2244 | 228.6229 | V | 1639.9377 | 1621.9272 | 1622.9108 | 820.4689 | 14 |
| 5 | 543.2778 | 525.2673 | 526.2509 | 272.1389 | S | 1540.8744 | 1522.8588 | 1523.8423 | 770.9346 | 13 |
| 6 | 600.2993 | 582.3002 | 583.2723 | 300.6497 | G | 1453.8418 | 1435.8268 | 1436.8103 | 727.4186 | 12 |
| 7 | 728.3578 | 710.3473 | 711.3309 | 364.6789 | Q | 1396.8158 | 1378.8053 | 1379.7888 | 698.9079 | 11 |
| 8 | 841.4420 | 823.4314 | 824.4150 | 421.2210 | L | 1268.7563 | 1250.7467 | 1251.7302 | 634.8786 | 10 |
| 9 | 955.4849 | 937.4743 | 938.4579 | 478.2424 | N | 1155.6731 | 1137.6626 | 1138.6461 | 578.3365 | 9 |
| 10 | 1042.5168 | 1024.5063 | 1025.4899 | 521.7584 | S | 1041.6400 | 1023.6196 | 1024.6033 | 521.3225 | 8 |
| 11 | 1143.5646 | 1125.5541 | 1126.5376 | 572.2823 | T | 954.6168 | 936.5876 | 937.5712 | 477.7991 | 7 |
| 12 | 1327.6858 | 1309.6753 | 1310.6588 | 664.3429 | K(+56.03) | 853.5608 | 835.5400 | 836.5236 | 427.2753 | 6 |
| 13 | 1455.7808 | 1437.7703 | 1438.7538 | 728.3904 | K | 669.4319 | 651.4268 | 652.4023 | 335.2147 | 5 |
| 14 | 1552.8335 | 1534.8230 | 1535.8065 | 776.9167 | P | 541.3362 | 523.3262 | 524.3074 | 271.1672 | 4 |
| 15 | 1649.8862 | 1631.8757 | 1632.8593 | 825.4431 | P | 444.2838 | 426.2702 | 427.2546 | 222.6408 | 3 |
| 16 | 1850.0023 | 1831.9918 | 1832.9753 | 925.5012 | K(+72.02) | 347.2300 | 329.2211 | 330.2019 | 174.1144 | 2 |
| 17 | | | | | K | 147.1131 | 129.1023 | 130.0866 | 74.0564 | 1 |

**Figure S14_12**

**MSMS assignment of K364ox in Bromodomain-containing protein 3**

BRD3(364-381): K*[O]MDGREYPDAQGFAADVR; -10lgP 67.92; Scan 18664 (MC277)

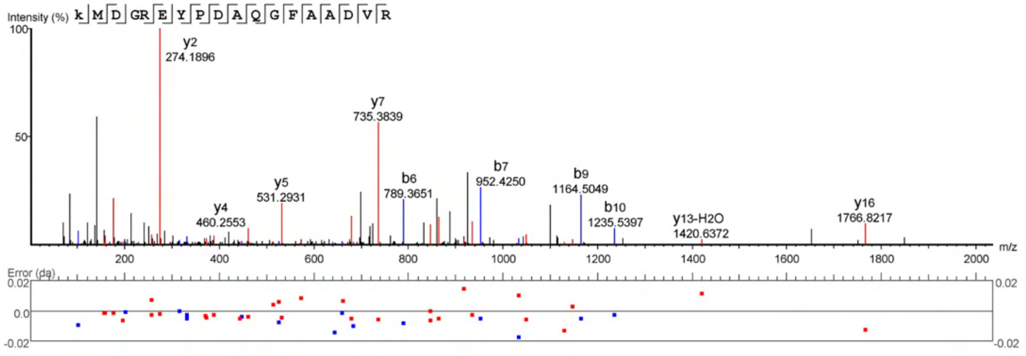


| # | b | b-H2O | b-NH3 | b (2+) | Seq | y | y-H2O | y-NH3 | y (2+) | # |
|---|---|---|---|---|---|---|---|---|---|---|
| 1 | 201.1251 | 183.1134 | 184.0969 | 101.0713 | K(+72.02) | | | | | 18 |
| 2 | 332.1671 | 314.1538 | 315.1379 | 166.5822 | M | 1897.8496 | 1879.8391 | 1880.8226 | 949.4248 | 17 |
| 3 | 447.1956 | 429.1808 | 430.1644 | 224.0957 | D | 1766.8217 | 1748.7987 | 1749.7822 | 883.9046 | 16 |
| 4 | 504.2128 | 486.2022 | 487.1858 | 252.6064 | G | 1651.7822 | 1633.7717 | 1634.7552 | 826.3911 | 15 |
| 5 | 660.3157 | 642.3033 | 643.3013 | 330.6621 | R | 1594.7607 | 1576.7502 | 1577.7338 | 797.8804 | 14 |
| 6 | 789.3651 | 771.3459 | 772.3295 | 395.1783 | E | 1438.6597 | 1420.6372 | 1421.6327 | 719.8298 | 13 |
| 7 | 952.4250 | 934.4092 | 935.3928 | 476.7099 | Y | 1309.6171 | 1291.6066 | 1292.5901 | 655.3085 | 12 |
| 8 | 1049.4725 | 1031.4620 | 1032.4630 | 525.2439 | P | 1146.5500 | 1128.5432 | 1129.5397 | 573.7678 | 11 |
| 9 | 1164.5049 | 1146.4890 | 1147.4725 | 582.7498 | D | 1049.5067 | 1031.4905 | 1032.4630 | 525.2439 | 10 |
| 10 | 1235.5397 | 1217.5261 | 1218.5096 | 618.2683 | A | 934.4766 | 916.4635 | 917.4320 | 467.7370 | 9 |
| 11 | 1363.5952 | 1345.5847 | 1346.5682 | 682.3079 | Q | 863.4421 | 845.4261 | 846.4167 | 432.2185 | 8 |
| 12 | 1420.6167 | 1402.6062 | 1403.5897 | 710.8083 | G | 735.3839 | 717.3678 | 718.3514 | 368.1892 | 7 |
| 13 | 1567.6851 | 1549.6746 | 1550.6581 | 784.3425 | F | 678.3624 | 660.3464 | 661.3229 | 339.6785 | 6 |
| 14 | 1638.7222 | 1620.7117 | 1621.6952 | 819.8611 | A | 531.2931 | 513.2780 | 514.2572 | 266.1443 | 5 |
| 15 | 1709.7593 | 1691.7488 | 1692.7323 | 855.3796 | A | 460.2553 | 442.2408 | 443.2299 | 230.6257 | 4 |
| 16 | 1824.7863 | 1806.7758 | 1807.7593 | 912.8931 | D | 389.2171 | 371.2073 | 372.1920 | 195.1134 | 3 |
| 17 | 1923.8546 | 1905.8441 | 1906.8276 | 962.4273 | V | 274.1896 | 256.1693 | 257.1630 | 137.5937 | 2 |
| 18 | | | | | R | 175.1203 | 157.1099 | 158.0934 | 88.0595 | 1 |

Figure S14_13

**MSMS assignment of K487ox in Bromodomain-containing protein 3**

BRD3(472-489): AVHEQLAALSQAPVNK[O]PK; -10lgP 47.68; Scan 12743 (MC271)

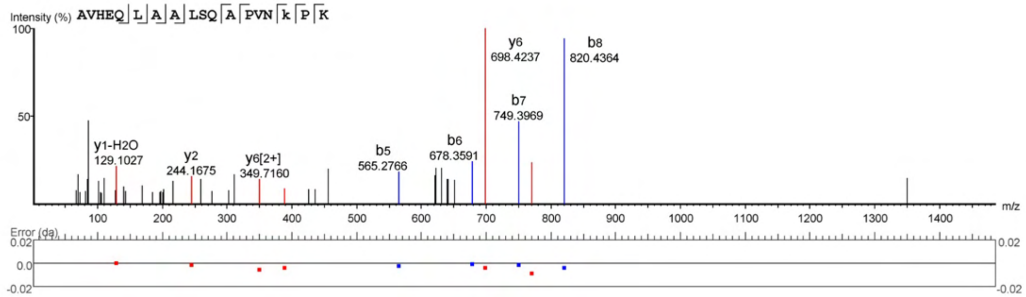


| # | b | b-H2O | b-NH3 | b (2+) | Seq | y | y-H2O | y-NH3 | y (2+) | # |
|---|---|---|---|---|---|---|---|---|---|---|
| 1 | 72.0449 | 54.0344 | 55.0179 | 36.5225 | A | | | | | 18 |
| 2 | 171.1134 | 153.1028 | 154.0863 | 86.0567 | V | 1846.0181 | 1828.0076 | 1828.9911 | 923.5090 | 17 |
| 3 | 308.1722 | 290.1617 | 291.1453 | 154.5861 | H | 1746.9496 | 1728.9391 | 1729.9226 | 873.9748 | 16 |
| 4 | 437.2148 | 419.2043 | 420.1879 | 219.1074 | E | 1609.8907 | 1591.8802 | 1592.8638 | 805.4454 | 15 |
| 5 | 565.2766 | 547.2629 | 548.2465 | 283.1367 | Q | 1480.8481 | 1462.8376 | 1463.8212 | 740.9241 | 14 |
| 6 | 678.3591 | 660.3469 | 661.3305 | 339.6787 | L | 1352.7896 | 1334.7791 | 1335.7626 | 676.8948 | 13 |
| 7 | 749.3969 | 731.3840 | 732.3676 | 375.1973 | A | 1239.7054 | 1221.6949 | 1222.6785 | 620.3527 | 12 |
| 8 | 820.4364 | 802.4211 | 803.4047 | 410.7159 | A | 1168.6683 | 1150.6578 | 1151.6414 | 584.8342 | 11 |
| 9 | 933.5157 | 915.5052 | 916.4888 | 467.2579 | L | 1097.6312 | 1079.6207 | 1080.6042 | 549.3156 | 10 |
| 10 | 1020.5478 | 1002.5372 | 1003.5208 | 510.7739 | S | 984.5472 | 966.5367 | 967.5203 | 492.7736 | 9 |
| 11 | 1148.6063 | 1130.5958 | 1131.5793 | 574.8032 | Q | 897.5152 | 879.5046 | 880.4882 | 449.2576 | 8 |
| 12 | 1219.6434 | 1201.6329 | 1202.6165 | 610.3217 | A | 769.4656 | 751.4460 | 752.4296 | 385.2283 | 7 |
| 13 | 1316.6963 | 1298.6858 | 1299.6693 | 658.8481 | P | 698.4237 | 680.4089 | 681.3925 | 349.7160 | 6 |
| 14 | 1415.7646 | 1397.7542 | 1398.7377 | 708.3823 | V | 601.3668 | 583.3562 | 584.3398 | 301.1834 | 5 |
| 15 | 1529.8076 | 1511.7971 | 1512.7806 | 765.4038 | N | 502.2983 | 484.2878 | 485.2714 | 251.6492 | 4 |
| 16 | 1673.8975 | 1655.8870 | 1656.8705 | 837.4487 | K(+15.99) | 388.2600 | 370.2448 | 371.2284 | 194.6277 | 3 |
| 17 | 1770.9502 | 1752.9397 | 1753.9232 | 885.9751 | P | 244.1675 | 226.1550 | 227.1385 | 122.5828 | 2 |
| 18 | | | | | K | 147.1128 | 129.1027 | 130.0858 | 74.0564 | 1 |

Figure S14_14

**MSMS assignment of K489ox in Bromodomain-containing protein 3**

BRD3(472-490): AVHEQLAALSQAPVNK*PK*[O]K; -10lgP 53.80; Scan 19269 (MC271)

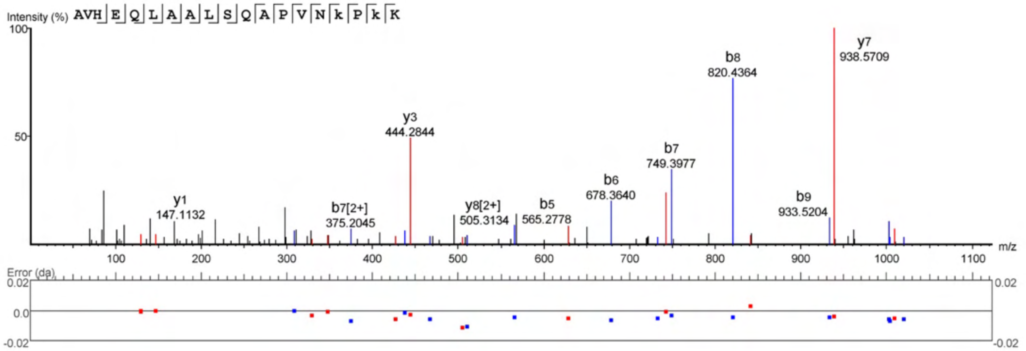


| # | b | b-H2O | b-NH3 | b (2+) | Seq | y | y-H2O | y-NH3 | y (2+) | # |
|---|---|---|---|---|---|---|---|---|---|---|
| 1 | 72.0449 | 54.0344 | 55.0179 | 36.5225 | A | | | | | 19 |
| 2 | 171.1134 | 153.1028 | 154.0863 | 86.0567 | V | 2086.1655 | 2068.1550 | 2069.1384 | 1043.5828 | 18 |
| 3 | 308.1729 | 290.1617 | 291.1453 | 154.5861 | H | 1987.0970 | 1969.0865 | 1970.0701 | 994.0485 | 17 |
| 4 | 437.2166 | 419.2043 | 420.1879 | 219.1074 | E | 1850.0381 | 1832.0276 | 1833.0111 | 925.5190 | 16 |
| 5 | 565.2778 | 547.2629 | 548.2465 | 283.1367 | Q | 1720.9955 | 1702.9850 | 1703.9685 | 860.9977 | 15 |
| 6 | 678.3640 | 660.3469 | 661.3305 | 339.6787 | L | 1592.9370 | 1574.9265 | 1575.9100 | 796.9685 | 14 |
| 7 | 749.3977 | 731.3840 | 732.3727 | 375.2045 | A | 1479.8529 | 1461.8424 | 1462.8259 | 740.4265 | 13 |
| 8 | 820.4364 | 802.4211 | 803.4047 | 410.7159 | A | 1408.8158 | 1390.8053 | 1391.7888 | 704.9079 | 12 |
| 9 | 933.5204 | 915.5052 | 916.4888 | 467.2638 | L | 1337.7787 | 1319.7682 | 1320.7517 | 669.3893 | 11 |
| 10 | 1020.5537 | 1002.5432 | 1003.5280 | 510.7845 | S | 1224.6946 | 1206.6841 | 1207.6676 | 612.8473 | 10 |
| 11 | 1148.6063 | 1130.5958 | 1131.5793 | 574.8032 | Q | 1137.6626 | 1119.6521 | 1120.6356 | 569.3313 | 9 |
| 12 | 1219.6434 | 1201.6329 | 1202.6165 | 610.3217 | A | 1009.6091 | 991.5934 | 992.5770 | 505.3134 | 8 |
| 13 | 1316.6963 | 1298.6858 | 1299.6693 | 658.8481 | P | 938.5709 | 920.5563 | 921.5399 | 469.7834 | 7 |
| 14 | 1415.7646 | 1397.7542 | 1398.7377 | 708.3823 | V | 841.5107 | 823.5036 | 824.4872 | 421.2571 | 6 |
| 15 | 1529.8076 | 1511.7971 | 1512.7806 | 765.4038 | N | 742.4467 | 724.4352 | 725.4188 | 371.7229 | 5 |
| 16 | 1713.9287 | 1695.9182 | 1696.9017 | 857.4644 | K(+56.03) | 628.4083 | 610.3922 | 611.3758 | 314.7014 | 4 |
| 17 | 1810.9814 | 1792.9709 | 1793.9545 | 905.9907 | P | 444.2844 | 426.2767 | 427.2546 | 222.6408 | 3 |
| 18 | 2011.0975 | 1993.0870 | 1994.0706 | 1006.0488 | K(+72.02) | 347.2301 | 329.2216 | 330.2019 | 174.1144 | 2 |
| 19 | | | | | K | 147.1132 | 129.1027 | 130.0869 | 74.0564 | 1 |

Figure S14_15

**MSMS assignment of K651ox in Bromodomain-containing protein 3**

BRD3(643-655): K*PFSASGK*K*[O]QAAK; -10lgP 55.45; Scan 12620 (MC303)

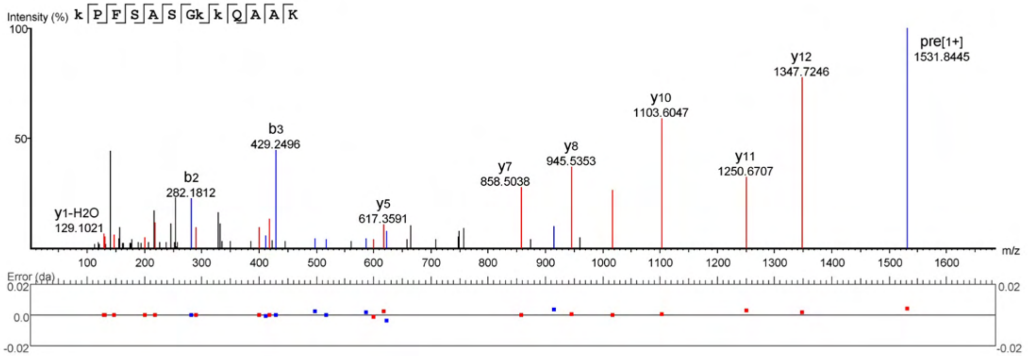


| # | b | b-H2O | b-NH3 | b (2+) | Seq | y | y-H2O | y-NH3 | y (2+) | # |
|---|---|---|---|---|---|---|---|---|---|---|
| 1 | 185.1290 | 167.1184 | 168.1020 | 93.0645 | K(+56.03) | | | | | 13 |
| 2 | 282.1812 | 264.1712 | 265.1548 | 141.5909 | P | 1347.7246 | 1329.7161 | 1330.6996 | 674.3633 | 12 |
| 3 | 429.2496 | 411.2403 | 412.2232 | 215.1251 | F | 1250.6707 | 1232.6633 | 1233.6469 | 625.8369 | 11 |
| 4 | 516.2827 | 498.2689 | 499.2552 | 258.6411 | S | 1103.6047 | 1085.5950 | 1086.5785 | 552.3027 | 10 |
| 5 | 587.3170 | 569.3087 | 570.2923 | 294.1596 | A | 1016.5729 | 998.5629 | 999.5464 | 508.7867 | 9 |
| 6 | 674.3513 | 656.3408 | 657.3243 | 337.6757 | S | 945.5353 | 927.5258 | 928.5093 | 473.2682 | 8 |
| 7 | 731.3728 | 713.3622 | 714.3458 | 366.1864 | G | 858.5038 | 840.4937 | 841.4773 | 429.7521 | 7 |
| 8 | 915.4902 | 897.4834 | 898.4670 | 458.2470 | K(+56.03) | 801.4828 | 783.4723 | 784.4559 | 401.2414 | 6 |
| 9 | 1115.6100 | 1097.5995 | 1098.5830 | 558.3050 | K(+72.02) | 617.3591 | 599.3511 | 600.3362 | 309.1808 | 5 |
| 10 | 1243.6686 | 1225.6581 | 1226.6416 | 622.3385 | Q | 417.2452 | 399.2350 | 400.2191 | 209.1228 | 4 |
| 11 | 1314.7057 | 1296.6952 | 1297.6787 | 657.8528 | A | 289.1874 | 271.1765 | 272.1600 | 145.0935 | 3 |
| 12 | 1385.7428 | 1367.7323 | 1368.7158 | 693.3714 | A | 218.1498 | 200.1393 | 201.1233 | 109.5750 | 2 |
| 13 | | | | | K | 147.1128 | 129.1021 | 130.0863 | 74.0564 | 1 |

**Figure S14_16**

## MSMS assignment of K683ox in Bromodomain-containing protein 3

BRD3(672-687): LQDVSGQLSSSK*[O]KPAR; -10lgP 44.60; Scan 10327 (MC271)

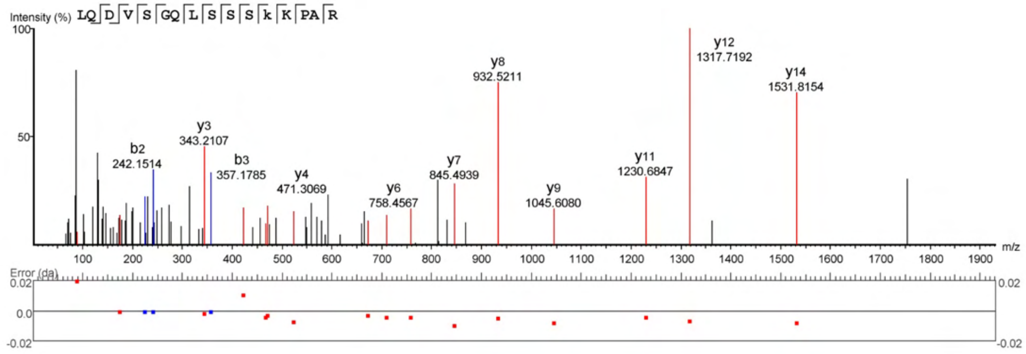


| # | b | b-H2O | b-NH3 | b (2+) | Seq | y | y-H2O | y-NH3 | y (2+) | # |
|---|---|---|---|---|---|---|---|---|---|---|
| 1 | 114.0919 | 96.0813 | 97.0649 | 57.5459 | L | | | | | 16 |
| 2 | 242.1514 | 224.1399 | 225.1243 | 121.5752 | Q | 1659.8660 | 1641.8555 | 1642.8390 | 830.4330 | 15 |
| 3 | 357.1785 | 339.1668 | 340.1504 | 179.0887 | D | 1531.8154 | 1513.7969 | 1514.7804 | 766.4037 | 14 |
| 4 | 456.2458 | 438.2352 | 439.2188 | 228.6229 | V | 1416.7805 | 1398.7700 | 1399.7535 | 708.8950 | 13 |
| 5 | 543.2778 | 525.2673 | 526.2509 | 272.1389 | S | 1317.7192 | 1299.7015 | 1300.6851 | 659.3560 | 12 |
| 6 | 600.2993 | 582.2888 | 583.2723 | 300.6497 | G | 1230.6847 | 1212.6696 | 1213.6531 | 615.8400 | 11 |
| 7 | 728.3578 | 710.3473 | 711.3309 | 364.6789 | Q | 1173.6586 | 1155.6481 | 1156.6316 | 587.3293 | 10 |
| 8 | 841.4420 | 823.4314 | 824.4150 | 421.2210 | L | 1045.6080 | 1027.5895 | 1028.5730 | 523.3078 | 9 |
| 9 | 928.4739 | 910.4634 | 911.4470 | 464.7370 | S | 932.5211 | 914.5054 | 915.4890 | 466.7626 | 8 |
| 10 | 1015.5060 | 997.4954 | 998.4790 | 508.2530 | S | 845.4939 | 827.4733 | 828.4569 | 423.2310 | 7 |
| 11 | 1102.5380 | 1084.5275 | 1085.5110 | 551.7690 | S | 758.4567 | 740.4413 | 741.4249 | 379.7259 | 6 |
| 12 | 1302.6541 | 1284.6436 | 1285.6271 | 651.8270 | K(+72.02) | 671.4230 | 653.4093 | 654.3929 | 336.2099 | 5 |
| 13 | 1430.7490 | 1412.7385 | 1413.7220 | 715.8745 | K | 471.3069 | 453.2932 | 454.2768 | 236.1519 | 4 |
| 14 | 1527.8018 | 1509.7913 | 1510.7748 | 764.4009 | P | 343.2107 | 325.1982 | 326.1818 | 172.1044 | 3 |
| 15 | 1598.8389 | 1580.8284 | 1581.8119 | 799.9194 | A | 246.1561 | 228.1455 | 229.1290 | 123.5780 | 2 |
| 16 | | | | | R | 175.1200 | 157.1084 | 158.0919 | 88.0397 | 1 |

Figure S14_17

**MSMS assignment of K684ox in Bromodomain-containing protein 3**

BRD3(672-687): LQDVSGQLSSSK*K*[O]PAR; -10lgP 63.47; Scan 14671 (MC277)

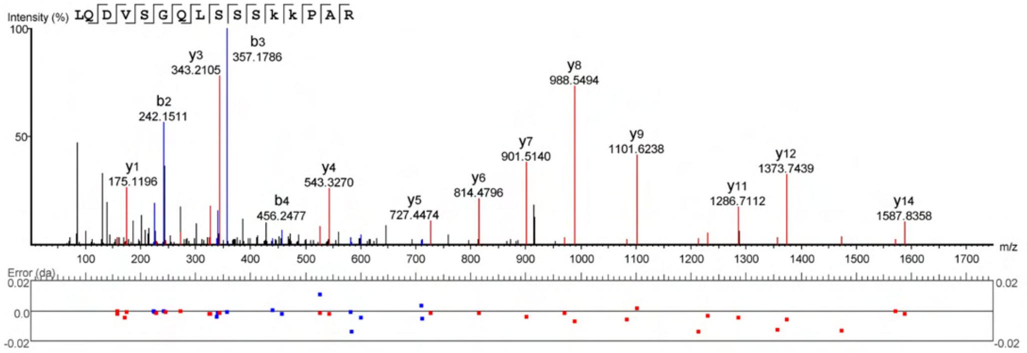


| # | b | b-H2O | b-NH3 | b (2+) | Seq | y | y-H2O | y-NH3 | y (2+) | # |
|---|---|---|---|---|---|---|---|---|---|---|
| 1 | 114.0919 | 96.0813 | 97.0649 | 57.5459 | L | | | | | 16 |
| 2 | 242.1511 | 224.1402 | 225.1245 | 121.5752 | Q | 1715.8922 | 1697.8817 | 1698.8652 | 858.4461 | 15 |
| 3 | 357.1786 | 339.1705 | 340.1520 | 179.0887 | D | 1587.8358 | 1569.8231 | 1570.8066 | 794.4168 | 14 |
| 4 | 456.2477 | 438.2352 | 439.2176 | 228.6229 | V | 1472.8197 | 1454.7961 | 1455.7797 | 736.9033 | 13 |
| 5 | 543.2778 | 525.2557 | 526.2509 | 272.1389 | S | 1373.7439 | 1355.7278 | 1356.7241 | 687.3691 | 12 |
| 6 | 600.3038 | 582.2896 | 583.2861 | 300.6497 | G | 1286.7112 | 1268.6958 | 1269.6793 | 643.8531 | 11 |
| 7 | 728.3578 | 710.3431 | 711.3362 | 364.6789 | Q | 1229.6882 | 1211.6743 | 1212.6714 | 615.3424 | 10 |
| 8 | 841.4420 | 823.4314 | 824.4150 | 421.2210 | L | 1101.6238 | 1083.6218 | 1084.5992 | 551.3131 | 9 |
| 9 | 928.4739 | 910.4634 | 911.4470 | 464.7370 | S | 988.5494 | 970.5330 | 971.5151 | 494.7711 | 8 |
| 10 | 1015.5060 | 997.4954 | 998.4790 | 508.2530 | S | 901.5140 | 883.4996 | 884.4832 | 451.2551 | 7 |
| 11 | 1102.5380 | 1084.5275 | 1085.5110 | 551.7690 | S | 814.4796 | 796.4675 | 797.4511 | 407.7390 | 6 |
| 12 | 1286.6592 | 1268.6487 | 1269.6322 | 643.8296 | K(+56.03) | 727.4474 | 709.4355 | 710.4191 | 364.2230 | 5 |
| 13 | 1486.7753 | 1468.7648 | 1469.7483 | 743.8876 | K(+72.02) | 543.3270 | 525.3143 | 526.2993 | 272.1624 | 4 |
| 14 | 1583.8280 | 1565.8175 | 1566.8010 | 792.4140 | P | 343.2105 | 325.2004 | 326.1840 | 172.1088 | 3 |
| 15 | 1654.8651 | 1636.8546 | 1637.8381 | 827.9326 | A | 246.1571 | 228.1455 | 229.1308 | 123.5780 | 2 |
| 16 | | | | | R | 175.1196 | 157.1102 | 158.0924 | 88.0595 | 1 |

**Figure S14_18**

**MSMS assignment of K286ox, K289ox in Bromodomain-containing protein 4**

BRD4(284-290): TK*K*[O]GVK*[O]R; -10lgP 27.87; Scan 4997 (MC264)

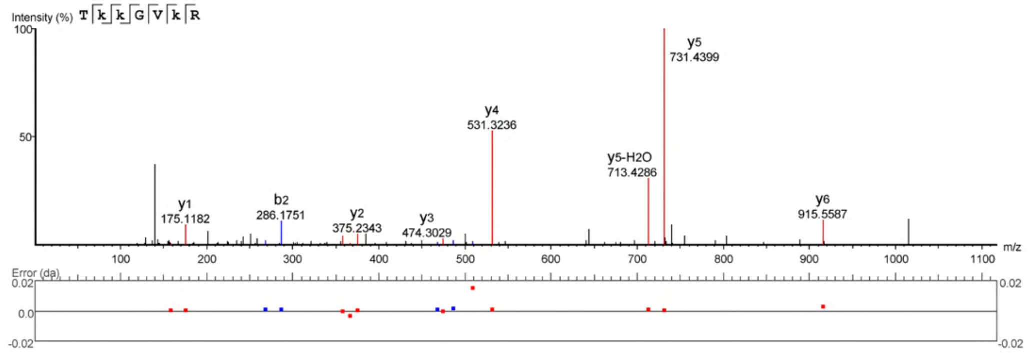


| # | b | b-H2O | b-NH3 | b (2+) | Seq | y | y-H2O | y-NH3 | y (2+) | # |
|---|---|---|---|---|---|---|---|---|---|---|
| 1 | 102.0555 | 84.0450 | 85.0285 | 51.5278 | T | | | | | 7 |
| 2 | 286.1751 | 268.1649 | 269.1497 | 143.5883 | K(+56.03) | 915.5587 | 897.5516 | 898.5352 | 458.2811 | 6 |
| 3 | 486.2903 | 468.2805 | 469.2658 | 243.6464 | K(+72.02) | 731.4399 | 713.4286 | 714.4140 | 366.2236 | 5 |
| 4 | 543.3142 | 525.3036 | 526.2872 | 272.1571 | G | 531.3236 | 513.3143 | 514.2979 | 266.1624 | 4 |
| 5 | 642.3826 | 624.3721 | 625.3557 | 321.6913 | V | 474.3029 | 456.2929 | 457.2765 | 237.6517 | 3 |
| 6 | 842.4987 | 824.4882 | 825.4717 | 421.7494 | K(+72.02) | 375.2343 | 357.2245 | 358.2078 | 188.1175 | 2 |
| 7 | | | | | R | 175.1182 | 157.1084 | 158.0913 | 88.0595 | 1 |

Figure S14_19

**MSMS assignment of K291ox in Bromodomain-containing protein 4**

BRD4(291-314): K*[O]ADTTTPTTIDPIHEPPSLPPEPK; -10lgP 40.91; Scan 12172 (MC264)

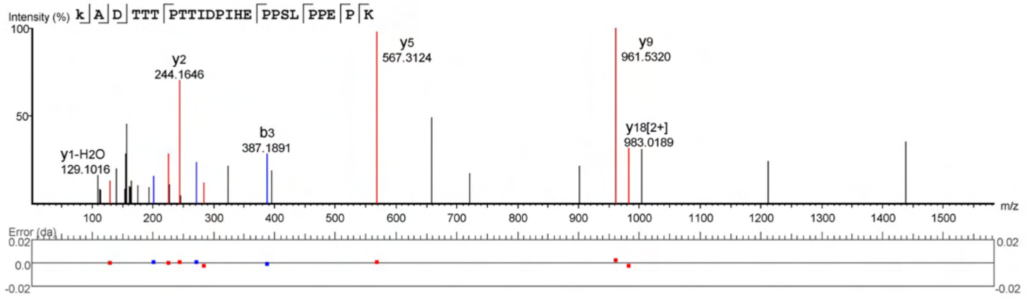


| # | b | b-H2O | b-NH3 | b (2+) | Seq | y | y-H2O | y-NH3 | y (2+) | # |
|---|---|---|---|---|---|---|---|---|---|---|
| 1 | 201.1227 | 183.1134 | 184.0969 | 101.0620 | K(+72.02) | | | | | 24 |
| 2 | 272.1597 | 254.1505 | 255.1340 | 136.5805 | A | 2454.2397 | 2436.2292 | 2437.2126 | 1227.6199 | 23 |
| 3 | 387.1891 | 369.1774 | 370.1610 | 194.0940 | D | 2383.2026 | 2365.1921 | 2366.1755 | 1192.1013 | 22 |
| 4 | 488.2356 | 470.2251 | 471.2086 | 244.6178 | T | 2268.1758 | 2250.1653 | 2251.1487 | 1134.5879 | 21 |
| 5 | 589.2833 | 571.2728 | 572.2563 | 295.1417 | T | 2167.1282 | 2149.1177 | 2150.1011 | 1084.0641 | 20 |
| 6 | 690.3310 | 672.3204 | 673.3040 | 345.6655 | T | 2066.0803 | 2048.0698 | 2049.0532 | 1033.5402 | 19 |
| 7 | 787.3838 | 769.3732 | 770.3568 | 394.1919 | P | 1965.0327 | 1947.0222 | 1948.0057 | 983.0189 | 18 |
| 8 | 888.4315 | 870.4209 | 871.4045 | 444.7157 | T | 1867.9800 | 1849.9695 | 1850.9530 | 934.4900 | 17 |
| 9 | 989.4791 | 971.4686 | 972.4521 | 495.2396 | T | 1766.9323 | 1748.9218 | 1749.9053 | 883.9661 | 16 |
| 10 | 1102.5632 | 1084.5527 | 1085.5363 | 551.7816 | I | 1665.8845 | 1647.8740 | 1648.8575 | 833.4423 | 15 |
| 11 | 1217.5901 | 1199.5796 | 1200.5631 | 609.2950 | D | 1552.8005 | 1534.7900 | 1535.7736 | 776.9003 | 14 |
| 12 | 1314.6428 | 1296.6323 | 1297.6158 | 657.8214 | P | 1437.7736 | 1419.7631 | 1420.7466 | 719.3868 | 13 |
| 13 | 1427.7269 | 1409.7164 | 1410.7000 | 714.3635 | I | 1340.7208 | 1322.7103 | 1323.6938 | 670.8604 | 12 |
| 14 | 1564.7859 | 1546.7754 | 1547.7589 | 782.8929 | H | 1227.6367 | 1209.6262 | 1210.6097 | 614.3184 | 11 |
| 15 | 1693.8285 | 1675.8180 | 1676.8015 | 847.4142 | E | 1090.5779 | 1072.5674 | 1073.5509 | 545.7889 | 10 |
| 16 | 1790.8812 | 1772.8707 | 1773.8542 | 895.9406 | P | 961.5320 | 943.5247 | 944.5082 | 481.2676 | 9 |
| 17 | 1887.9340 | 1869.9235 | 1870.9070 | 944.4670 | P | 864.4825 | 846.4719 | 847.4555 | 432.7412 | 8 |
| 18 | 1974.9659 | 1956.9554 | 1957.9390 | 987.9830 | S | 767.4297 | 749.4192 | 750.4028 | 384.2149 | 7 |
| 19 | 2088.0500 | 2070.0396 | 2071.0229 | 1044.5250 | L | 680.3977 | 662.3871 | 663.3707 | 340.6989 | 6 |
| 20 | 2185.1028 | 2167.0923 | 2168.0757 | 1093.0514 | P | 567.3124 | 549.3031 | 550.2867 | 284.1596 | 5 |
| 21 | 2282.1555 | 2264.1450 | 2265.1284 | 1141.5778 | P | 470.2609 | 452.2503 | 453.2339 | 235.6304 | 4 |
| 22 | 2411.1982 | 2393.1877 | 2394.1711 | 1206.0991 | E | 373.2081 | 355.1976 | 356.1812 | 187.1041 | 3 |
| 23 | 2508.2510 | 2490.2405 | 2491.2239 | 1254.6255 | P | 244.1646 | 226.1545 | 227.1385 | 122.5828 | 2 |
| 24 | | | | | K | 147.1128 | 129.1016 | 130.0858 | 74.0564 | 1 |

**Figure S14_20**

**MSMS assignment of K332ox in Bromodomain-containing protein 4**

BRD4(323-333): ESSRPVK*PPK*[O]K; -10lgP 42.3; Scan 4602 (MC264)

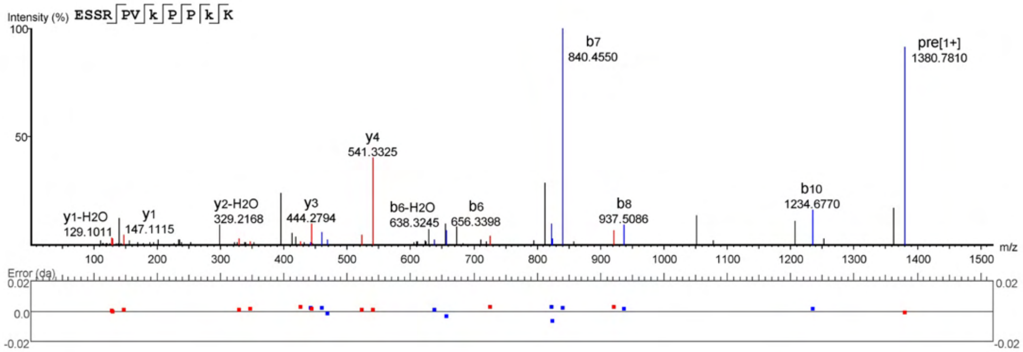


| # | b | b-H2O | b-NH3 | b (2+) | Seq | y | y-H2O | y-NH3 | y (2+) | # |
|---|---|---|---|---|---|---|---|---|---|---|
| 1 | 130.0504 | 112.0399 | 113.0234 | 65.5252 | E | | | | | 11 |
| 2 | 217.0824 | 199.0719 | 200.0554 | 109.0412 | S | 1251.7419 | 1233.7314 | 1234.7150 | 626.3710 | 10 |
| 3 | 304.1144 | 286.1039 | 287.0875 | 152.5572 | S | 1164.7098 | 1146.6993 | 1147.6829 | 582.8549 | 9 |
| 4 | 460.2129 | 442.2024 | 443.1886 | 230.6078 | R | 1077.6779 | 1059.6674 | 1060.6509 | 539.3389 | 8 |
| 5 | 557.2683 | 539.2578 | 540.2413 | 279.1342 | P | 921.5731 | 903.5662 | 904.5497 | 461.2884 | 7 |
| 6 | 656.3398 | 638.3245 | 639.3098 | 328.6684 | V | 824.5240 | 806.5134 | 807.4970 | 412.7620 | 6 |
| 7 | 840.4550 | 822.4440 | 823.4376 | 420.7289 | K(+56.03) | 725.4521 | 707.4450 | 708.4286 | 363.2278 | 5 |
| 8 | 937.5086 | 919.5001 | 920.4837 | 469.2566 | P | 541.3325 | 523.3224 | 524.3074 | 271.1672 | 4 |
| 9 | 1034.5635 | 1016.5529 | 1017.5365 | 517.7817 | P | 444.2794 | 426.2679 | 427.2546 | 222.6408 | 3 |
| 10 | 1234.6770 | 1216.6689 | 1217.6525 | 617.8397 | K(+72.02) | 347.2268 | 329.2168 | 330.2019 | 174.1144 | 2 |
| 11 | | | | | K | 147.1115 | 129.1011 | 130.0854 | 74.0564 | 1 |

**Figure S14_21**

**MSMS assignment of K535ox, K537ox, K538ox in Bromodomain-containing protein 4**

BRD4(520-539): AVHEQLAALSQPQQNK*[O]PK*[O]K*[O]K; -10lgP 68.97; Scan 16259 (MC273)

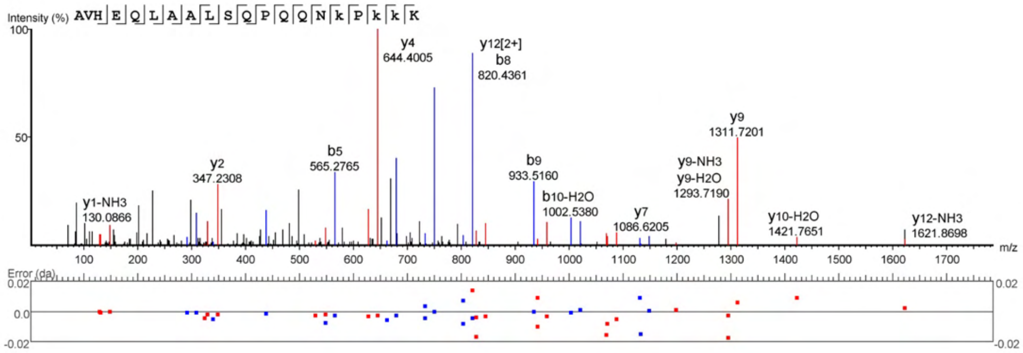


| # | b | b-H2O | b-NH3 | b (2+) | Seq | y | y-H2O | y-NH3 | y (2+) | # |
|---|---|---|---|---|---|---|---|---|---|---|
| 1 | 72.0449 | 54.0344 | 55.0179 | 36.5225 | A | | | | | 20 |
| 2 | 171.1134 | 153.1028 | 154.0863 | 86.0567 | V | 2388.2881 | 2370.2776 | 2371.2610 | 1194.6440 | 19 |
| 3 | 308.1733 | 290.1629 | 291.1453 | 154.5861 | H | 2289.2197 | 2271.2092 | 2272.1926 | 1145.1099 | 18 |
| 4 | 437.2165 | 419.2043 | 420.1879 | 219.1074 | E | 2152.1606 | 2134.1501 | 2135.1335 | 1076.5803 | 17 |
| 5 | 565.2765 | 547.2703 | 548.2465 | 283.1367 | Q | 2023.1182 | 2005.1077 | 2006.0912 | 1012.0591 | 16 |
| 6 | 678.3603 | 660.3469 | 661.3363 | 339.6843 | L | 1895.0596 | 1877.0491 | 1878.0326 | 948.0298 | 15 |
| 7 | 749.3951 | 731.3802 | 732.3719 | 375.1973 | A | 1781.9756 | 1763.9651 | 1764.9486 | 891.4878 | 14 |
| 8 | 820.4361 | 802.4137 | 803.4129 | 410.7159 | A | 1710.9385 | 1692.9280 | 1693.9115 | 855.9692 | 13 |
| 9 | 933.5160 | 915.5052 | 916.4888 | 467.2579 | L | 1639.9014 | 1621.8909 | 1622.8717 | 820.4361 | 12 |
| 10 | 1020.5461 | 1002.5380 | 1003.5208 | 510.7739 | S | 1526.8173 | 1508.8068 | 1509.7903 | 763.9086 | 11 |
| 11 | 1148.6055 | 1130.5862 | 1131.5945 | 574.8032 | Q | 1439.7853 | 1421.7651 | 1422.7583 | 720.3926 | 10 |
| 12 | 1245.6592 | 1227.6487 | 1228.6322 | 623.3296 | P | 1311.7201 | 1293.7190 | 1294.7170 | 656.3633 | 9 |
| 13 | 1373.7177 | 1355.7072 | 1356.6907 | 687.3588 | Q | 1214.6738 | 1196.6633 | 1197.6453 | 607.8369 | 8 |
| 14 | 1501.7762 | 1483.7657 | 1484.7493 | 751.3881 | Q | 1086.6205 | 1068.6204 | 1069.5966 | 543.8077 | 7 |
| 15 | 1615.8192 | 1597.8087 | 1598.7922 | 808.4096 | N | 958.5603 | 940.5367 | 941.5397 | 479.7784 | 6 |
| 16 | 1815.9353 | 1797.9248 | 1798.9083 | 908.4677 | K(+72.02) | 844.5172 | 826.5071 | 827.5037 | 422.7569 | 5 |
| 17 | 1912.9880 | 1894.9775 | 1895.9611 | 956.9940 | P | 644.4005 | 626.3903 | 627.3707 | 322.7035 | 4 |
| 18 | 2113.1042 | 2095.0938 | 2096.0771 | 1057.0521 | K(+72.02) | 547.3474 | 529.3374 | 530.3180 | 274.1725 | 3 |
| 19 | 2313.2202 | 2295.2097 | 2296.1931 | 1157.1101 | K(+72.02) | 347.2308 | 329.2203 | 330.2019 | 174.1144 | 2 |
| 20 | | | | | K | 147.1132 | 129.1027 | 130.0866 | 74.0564 | 1 |

**Figure S14_22**

## MSMS assignment of K539ox in Bromodomain-containing protein 4

BRD4(520-539): AVHEQLAALSQPQQNKPK*[O]K[O]K[O]; -10lgP 50.12; Scan 7539 (MC264)

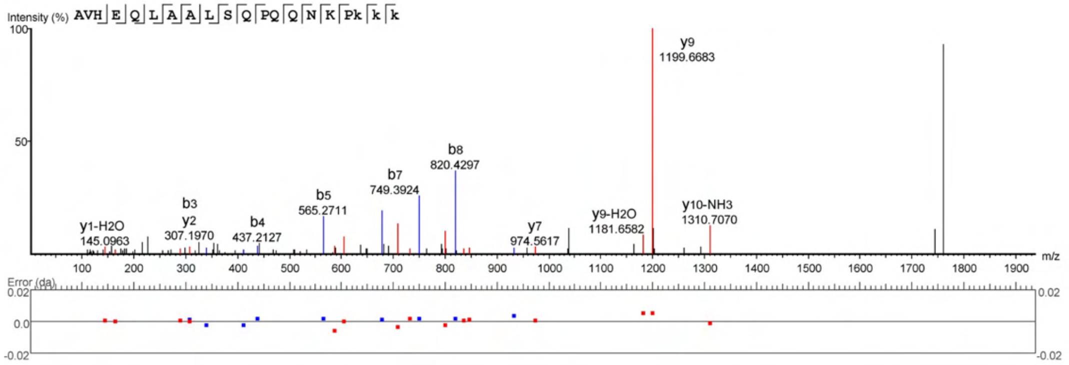


| # | b | b-H2O | b-NH3 | b (2+) | Seq | y | y-H2O | y-NH3 | y (2+) | # |
|---|---|---|---|---|---|---|---|---|---|---|
| 1 | 72.0449 | 54.0344 | 55.0179 | 36.5225 | A | | | | | 20 |
| 2 | 171.1134 | 153.1028 | 154.0863 | 86.0567 | V | 2276.2356 | 2258.2251 | 2259.2085 | 1138.6178 | 19 |
| 3 | 308.1708 | 290.1617 | 291.1453 | 154.5861 | H | 2177.1672 | 2159.1567 | 2160.1401 | 1089.0836 | 18 |
| 4 | 437.2127 | 419.2043 | 420.1879 | 219.1074 | E | 2040.1083 | 2022.0978 | 2023.0813 | 1020.5541 | 17 |
| 5 | 565.2711 | 547.2629 | 548.2465 | 283.1367 | Q | 1911.0658 | 1893.0553 | 1894.0388 | 956.0329 | 16 |
| 6 | 678.3560 | 660.3469 | 661.3305 | 339.6815 | L | 1783.0072 | 1764.9967 | 1765.9802 | 892.0036 | 15 |
| 7 | 749.3924 | 731.3840 | 732.3676 | 375.1973 | A | 1669.9231 | 1651.9126 | 1652.8961 | 835.4606 | 14 |
| 8 | 820.4297 | 802.4211 | 803.4047 | 410.7183 | A | 1598.8860 | 1580.8755 | 1581.8590 | 799.9457 | 13 |
| 9 | 933.5121 | 915.5052 | 916.4888 | 467.2579 | L | 1527.8489 | 1509.8384 | 1510.8219 | 764.4244 | 12 |
| 10 | 1020.5478 | 1002.5372 | 1003.5208 | 510.7739 | S | 1414.7648 | 1396.7543 | 1397.7378 | 707.8861 | 11 |
| 11 | 1148.6063 | 1130.5958 | 1131.5793 | 574.8032 | Q | 1327.7328 | 1309.7223 | 1310.7070 | 664.3664 | 10 |
| 12 | 1245.6592 | 1227.6487 | 1228.6322 | 623.3296 | P | 1199.6683 | 1181.6582 | 1182.6472 | 600.3371 | 9 |
| 13 | 1373.7177 | 1355.7072 | 1356.6907 | 687.3588 | Q | 1102.6215 | 1084.6110 | 1085.5945 | 551.8107 | 8 |
| 14 | 1501.7762 | 1483.7657 | 1484.7493 | 751.3881 | Q | 974.5617 | 956.5523 | 957.5359 | 487.7814 | 7 |
| 15 | 1615.8192 | 1597.8087 | 1598.7922 | 808.4096 | N | 846.5031 | 828.4937 | 829.4773 | 423.7521 | 6 |
| 16 | 1743.9142 | 1725.9037 | 1726.8872 | 872.4571 | K | 732.4590 | 714.4508 | 715.4344 | 366.7307 | 5 |
| 17 | 1840.9669 | 1822.9564 | 1823.9399 | 920.9835 | P | 604.3669 | 586.3558 | 587.3459 | 302.6832 | 4 |
| 18 | 2041.0830 | 2023.0725 | 2024.0560 | 1021.0415 | K(+72.02) | 507.3137 | 489.3031 | 490.2867 | 254.1568 | 3 |
| 19 | 2185.1729 | 2167.1624 | 2168.1458 | 1093.0864 | K(+15.99) | 307.1970 | 289.1860 | 290.1706 | 154.0988 | 2 |
| 20 | | | | | K(+15.99) | 163.1072 | 145.0963 | 146.0807 | 82.0539 | 1 |

Figure S14_23

**MSMS assignment of K541ox, K544ox in Bromodomain-containing protein 4**

BRD4(538-546): K*[O]K*EK*[O]DK*K*[O]EK; -10lgP 16.75; Scan 9090 (MC273)

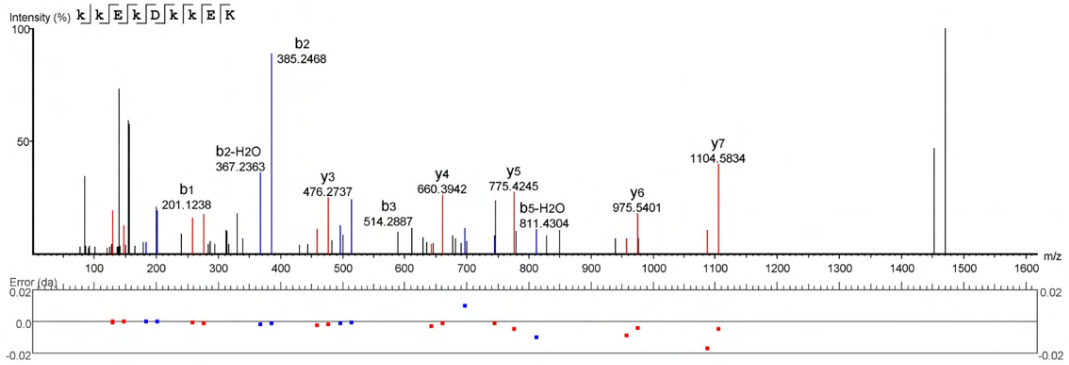


| # | b | b-H2O | b-NH3 | b (2+) | Seq | y | y-H2O | y-NH3 | y (2+) | # |
|---|---|---|---|---|---|---|---|---|---|---|
| 1 | 201.1238 | 183.1132 | 184.0969 | 101.0620 | K(+72.02) | | | | | 9 |
| 2 | 385.2468 | 367.2363 | 368.2181 | 193.1225 | K(+56.03) | 1288.6995 | 1270.6890 | 1271.6725 | 644.8497 | 8 |
| 3 | 514.2887 | 496.2787 | 497.2607 | 257.6438 | E | 1104.5834 | 1086.5850 | 1087.5513 | 552.7891 | 7 |
| 4 | 714.4037 | 696.3829 | 697.3768 | 357.7019 | K(+72.02) | 975.5401 | 957.5343 | 958.5087 | 488.2678 | 6 |
| 5 | 829.4307 | 811.4304 | 812.4037 | 415.2153 | D | 775.4245 | 757.4090 | 758.3926 | 388.2098 | 5 |
| 6 | 1013.5519 | 995.5413 | 996.5249 | 507.2759 | K(+56.03) | 660.3942 | 642.3855 | 643.3657 | 330.6963 | 4 |
| 7 | 1213.6680 | 1195.6575 | 1196.6410 | 607.3340 | K(+72.02) | 476.2737 | 458.2635 | 459.2445 | 238.6357 | 3 |
| 8 | 1342.7106 | 1324.7001 | 1325.6836 | 671.8553 | E | 276.1567 | 258.1460 | 259.1284 | 138.5777 | 2 |
| 9 | | | | | K | 147.1132 | 129.1024 | 130.0866 | 74.0564 | 1 |

Figure S14_24

**MSMS assignment of K547ox, K548ox, K550ox, K552ox in Bromodomain-containing protein 4**

BRD4(547-553): K*[O]K*[O]EK*[O]HK*[O]R; -10lgP 19.78; Scan 3905 (MC264)

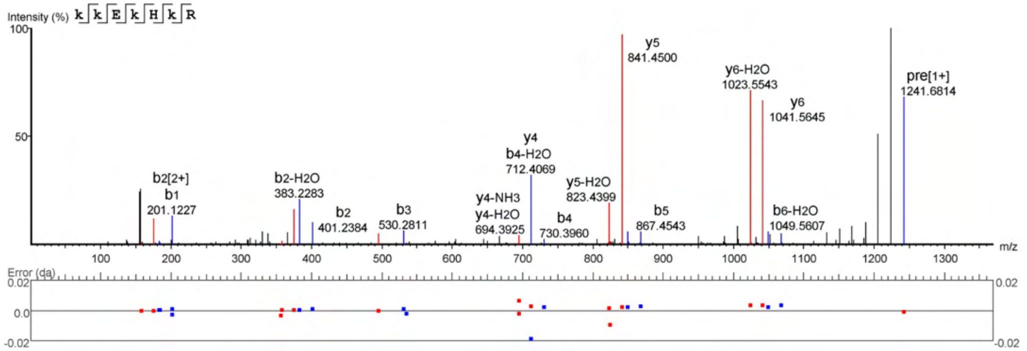


| # | b | b-H2O | b-NH3 | b (2+) | Seq | y | y-H2O | y-NH3 | y (2+) | # |
|---|---|---|---|---|---|---|---|---|---|---|
| 1 | 201.1227 | 183.1123 | 184.0961 | 101.0620 | K(+72.02) | | | | | 7 |
| 2 | 401.2384 | 383.2283 | 384.2130 | 201.1227 | K(+72.02) | 1041.5645 | 1023.5543 | 1024.5417 | 521.2844 | 6 |
| 3 | 530.2811 | 512.2720 | 513.2556 | 265.6413 | E | 841.4500 | 823.4399 | 824.4351 | 421.2263 | 5 |
| 4 | 730.3960 | 712.4069 | 713.3717 | 365.6993 | K(+72.02) | 712.4069 | 694.3925 | 695.3849 | 356.7081 | 4 |
| 5 | 867.4543 | 849.4443 | 850.4306 | 434.2288 | H | 512.2939 | 494.2834 | 495.2664 | 256.6470 | 3 |
| 6 | 1067.5698 | 1049.5607 | 1050.5468 | 534.2888 | K(+72.02) | 375.2344 | 357.2235 | 358.2072 | 188.1175 | 2 |
| 7 | | | | | R | 175.1185 | 157.1084 | 158.0918 | 88.0595 | 1 |

**Figure S14_25**

**MSMS assignment of K561ox in Bromodomain-containing protein 4**

BRD4(554-564): KEEVEENK*[O]K*SK; -10lgP 34.63; Scan 4151 (MC264)

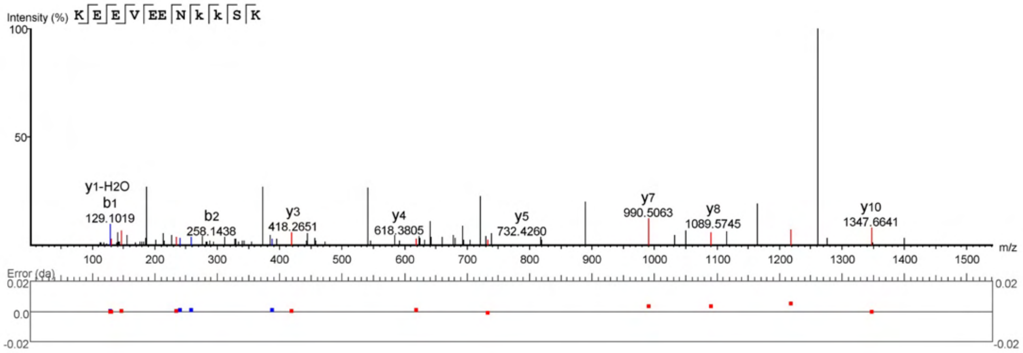


| # | b | b-H2O | b-NH3 | b (2+) | Seq | y | y-H2O | y-NH3 | y (2+) | # |
|---|---|---|---|---|---|---|---|---|---|---|
| 1 | 129.1019 | 111.0922 | 112.0758 | 65.0514 | K | | | | | 11 |
| 2 | 258.1438 | 240.1333 | 241.1184 | 129.5727 | E | 1347.6641 | 1329.6533 | 1330.6368 | 674.3319 | 10 |
| 3 | 387.1861 | 369.1774 | 370.1609 | 194.0940 | E | 1218.6151 | 1200.6107 | 1201.5942 | 609.8106 | 9 |
| 4 | 486.2563 | 468.2458 | 469.2294 | 243.6282 | V | 1089.5745 | 1071.5681 | 1072.5516 | 545.2893 | 8 |
| 5 | 615.2990 | 597.2884 | 598.2720 | 308.1495 | E | 990.5063 | 972.4996 | 973.4832 | 495.7551 | 7 |
| 6 | 744.3416 | 726.3310 | 727.3146 | 372.6708 | E | 861.4676 | 843.4570 | 844.4406 | 431.2338 | 6 |
| 7 | 858.3845 | 840.3739 | 841.3575 | 429.6922 | N | 732.4260 | 714.4144 | 715.3980 | 366.7125 | 5 |
| 8 | 1058.5005 | 1040.4900 | 1041.4735 | 529.7502 | K(+72.02) | 618.3805 | 600.3715 | 601.3551 | 309.6910 | 4 |
| 9 | 1242.6217 | 1224.6112 | 1225.5947 | 621.8109 | K(+56.03) | 418.2651 | 400.2554 | 401.2390 | 209.6330 | 3 |
| 10 | 1329.6538 | 1311.6433 | 1312.6268 | 665.3269 | S | 234.1438 | 216.1343 | 217.1178 | 117.5724 | 2 |
| 11 | | | | | K | 147.1122 | 129.1019 | 130.0854 | 74.0564 | 1 |

Figure S14_26

**MSMS assignment of K562ox in Bromodomain-containing protein 4**

BRD4(554-564): KEEVEENK*K*[O]SK; -10lgP 44.51; Scan 4019 (MC264)

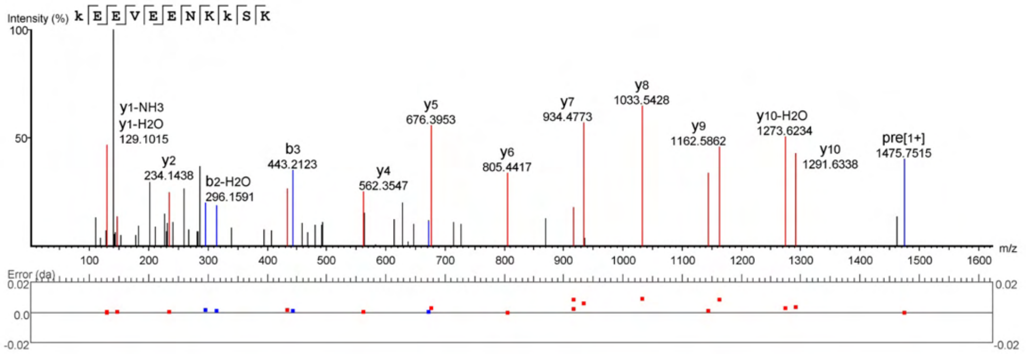


| # | b | b-H2O | b-NH3 | b (2+) | Seq | y | y-H2O | y-NH3 | y (2+) | # |
|---|---|---|---|---|---|---|---|---|---|---|
| 1 | 185.1290 | 167.1184 | 168.1020 | 93.0645 | K(+56.03) | | | | | 11 |
| 2 | 314.1702 | 296.1591 | 297.1446 | 157.5858 | E | 1291.6338 | 1273.6234 | 1274.6106 | 646.3188 | 10 |
| 3 | 443.2123 | 425.2036 | 426.1872 | 222.1071 | E | 1162.5862 | 1144.5828 | 1145.5680 | 581.7975 | 9 |
| 4 | 542.2826 | 524.2720 | 525.2556 | 271.6413 | V | 1033.5428 | 1015.5418 | 1016.5254 | 517.2762 | 8 |
| 5 | 671.3242 | 653.3146 | 654.2982 | 336.1626 | E | 934.4773 | 916.4644 | 917.4540 | 467.7420 | 7 |
| 6 | 800.3677 | 782.3572 | 783.3408 | 400.6839 | E | 805.4417 | 787.4308 | 788.4144 | 403.2207 | 6 |
| 7 | 914.4106 | 896.4001 | 897.3837 | 457.7053 | N | 676.3953 | 658.3882 | 659.3718 | 338.6994 | 5 |
| 8 | 1042.5056 | 1024.4951 | 1025.4786 | 521.7528 | K | 562.3547 | 544.3453 | 545.3289 | 281.6779 | 4 |
| 9 | 1242.6217 | 1224.6112 | 1225.5947 | 621.8109 | K(+72.02) | 434.2587 | 416.2503 | 417.2339 | 217.6304 | 3 |
| 10 | 1329.6538 | 1311.6433 | 1312.6268 | 665.3269 | S | 234.1438 | 216.1343 | 217.1178 | 117.5724 | 2 |
| 11 | | | | | K | 147.1120 | 129.1015 | 130.0858 | 74.0564 | 1 |

**Figure S14_27**

**MSMS assignment of K572ox in Bromodomain-containing protein 4**

BRD4(565-575): AK*EPPPK*K*[O]TK*K; -10lgP 39.21; Scan 7630 (MC264)

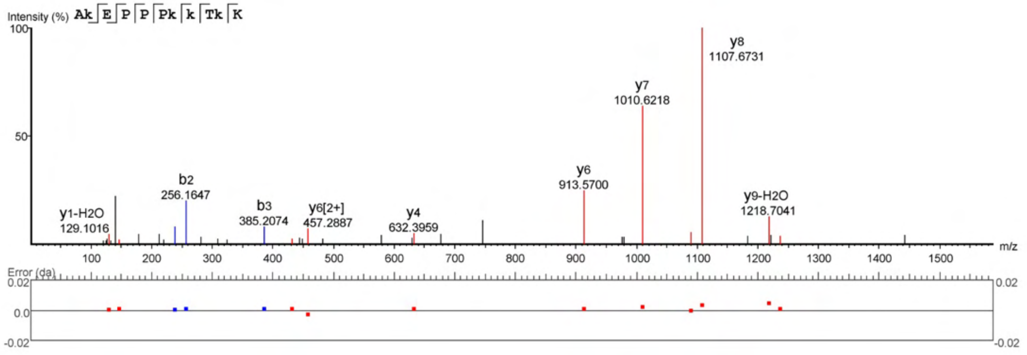


| # | b | b-H2O | b-NH3 | b (2+) | Seq | y | y-H2O | y-NH3 | y (2+) | # |
|---|---|---|---|---|---|---|---|---|---|---|
| 1 | 72.0449 | 54.0344 | 55.0179 | 36.5225 | A | | | | | 11 |
| 2 | 256.1647 | 238.1555 | 239.1382 | 128.5831 | K(+56.03) | 1420.8409 | 1402.8304 | 1403.8140 | 710.9205 | 10 |
| 3 | 385.2074 | 367.1981 | 368.1817 | 193.1043 | E | 1236.7179 | 1218.7041 | 1219.6927 | 618.8599 | 9 |
| 4 | 482.2614 | 464.2509 | 465.2345 | 241.6307 | P | 1107.6731 | 1089.6672 | 1090.6501 | 554.3386 | 8 |
| 5 | 579.3142 | 561.3036 | 562.2872 | 290.1571 | P | 1010.6218 | 992.6138 | 993.5974 | 505.8122 | 7 |
| 6 | 676.3669 | 658.3564 | 659.3400 | 338.6835 | P | 913.5700 | 895.5611 | 896.5447 | 457.2887 | 6 |
| 7 | 860.4881 | 842.4775 | 843.4611 | 430.7440 | K(+56.03) | 816.5189 | 798.5084 | 799.4919 | 408.7595 | 5 |
| 8 | 1060.6042 | 1042.5938 | 1043.5773 | 530.8021 | K(+72.02) | 632.3959 | 614.3871 | 615.3707 | 316.6989 | 4 |
| 9 | 1161.6519 | 1143.6414 | 1144.6249 | 581.3259 | T | 432.2802 | 414.2711 | 415.2546 | 216.6408 | 3 |
| 10 | 1345.7731 | 1327.7626 | 1328.7461 | 673.3865 | K(+56.03) | 331.2339 | 313.2234 | 314.2070 | 166.1170 | 2 |
| 11 | | | | | K | 147.1115 | 129.1016 | 130.0858 | 74.0564 | 1 |

Figure S14_28

**MSMS assignment of K575ox in Bromodomain-containing protein 4**

BRD4(573-585): TK*K*[O]NNSSNSNVSK; -10lgP 51.76; Scan 3741 (MC264)

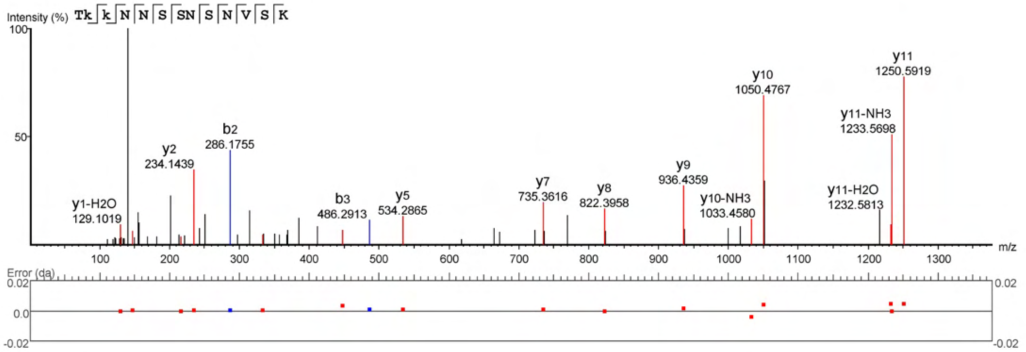


| # | b | b-H2O | b-NH3 | b (2+) | Seq | y | y-H2O | y-NH3 | y (2+) | # |
|---|---|---|---|---|---|---|---|---|---|---|
| 1 | 102.0555 | 84.0450 | 85.0285 | 51.5278 | T | | | | | 13 |
| 2 | 286.1755 | 268.1661 | 269.1497 | 143.5883 | K(+56.03) | 1434.7183 | 1416.7078 | 1417.6913 | 717.8591 | 12 |
| 3 | 486.2913 | 468.2822 | 469.2658 | 243.6464 | K(+72.02) | 1250.5919 | 1232.5813 | 1233.5698 | 625.7986 | 11 |
| 4 | 600.3357 | 582.3251 | 583.3087 | 300.6678 | N | 1050.4767 | 1032.4706 | 1033.4580 | 525.7405 | 10 |
| 5 | 714.3786 | 696.3680 | 697.3516 | 357.6893 | N | 936.4359 | 918.4276 | 919.4111 | 468.7191 | 9 |
| 6 | 801.4106 | 783.4001 | 784.3837 | 401.2053 | S | 822.3958 | 804.3846 | 805.3682 | 411.6976 | 8 |
| 7 | 888.4427 | 870.4321 | 871.4157 | 444.7213 | S | 735.3616 | 717.3526 | 718.3362 | 368.1816 | 7 |
| 8 | 1002.4856 | 984.4750 | 985.4586 | 501.7428 | N | 648.3311 | 630.3206 | 631.3041 | 324.6656 | 6 |
| 9 | 1089.5176 | 1071.5071 | 1072.4906 | 545.2588 | S | 534.2865 | 516.2776 | 517.2612 | 267.6441 | 5 |
| 10 | 1203.5605 | 1185.5500 | 1186.5336 | 602.2803 | N | 447.2523 | 429.2456 | 430.2292 | 224.1281 | 4 |
| 11 | 1302.6290 | 1284.6185 | 1285.6021 | 651.8145 | V | 333.2122 | 315.2027 | 316.1862 | 167.1066 | 3 |
| 12 | 1389.6610 | 1371.6505 | 1372.6340 | 695.3305 | S | 234.1439 | 216.1340 | 217.1178 | 117.5724 | 2 |
| 13 | | | | | K | 147.1121 | 129.1019 | 130.0858 | 74.0564 | 1 |

**Figure S14_29**

**MSMS assignment of K727ox in Bromodomain-containing protein 4**

BRD4(725-733): SK*K*[O]K*GHPGR; -10lgP 39.08; Scan 4485 (MC264)

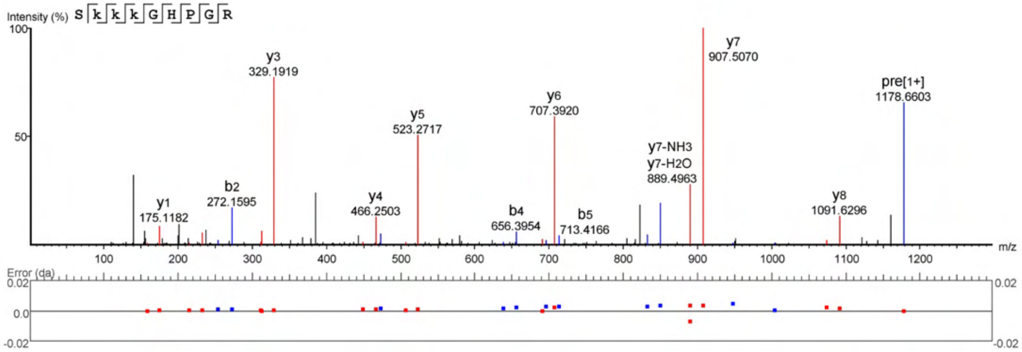


| # | b | b-H2O | b-NH3 | b (2+) | Seq | y | y-H2O | y-NH3 | y (2+) | # |
|---|---|---|---|---|---|---|---|---|---|---|
| 1 | 88.0399 | 70.0293 | 71.0129 | 44.5199 | S | | | | | 9 |
| 2 | 272.1595 | 254.1490 | 255.1340 | 136.5805 | K(+56.03) | 1091.6296 | 1073.6184 | 1074.6050 | 546.3160 | 8 |
| 3 | 472.2748 | 454.2665 | 455.2501 | 236.6385 | K(+72.02) | 907.5070 | 889.4963 | 890.4911 | 454.2554 | 7 |
| 4 | 656.3954 | 638.3853 | 639.3713 | 328.6991 | K(+56.03) | 707.3920 | 689.3842 | 690.3677 | 354.1974 | 6 |
| 5 | 713.4166 | 695.4056 | 696.3928 | 357.2099 | G | 523.2717 | 505.2630 | 506.2457 | 262.1368 | 5 |
| 6 | 850.4747 | 832.4647 | 833.4517 | 425.7393 | H | 466.2503 | 448.2415 | 449.2238 | 233.6260 | 4 |
| 7 | 947.5264 | 929.5208 | 930.5044 | 474.2657 | P | 329.1919 | 311.1814 | 312.1659 | 165.0966 | 3 |
| 8 | 1004.5521 | 986.5423 | 987.5259 | 502.7764 | G | 232.1394 | 214.1298 | 215.1127 | 116.5702 | 2 |
| 9 | | | | | R | 175.1182 | 157.1084 | 158.0921 | 88.0595 | 1 |

Figure S14_30

**MSMS assignment of K17ox in Ribosome biogenesis protein BRX1 homolog**

BRIX1(10-21): GGFAVQAK*[O]K*PK*R; -10lgP 32.83; Scan 25446 (MC336)

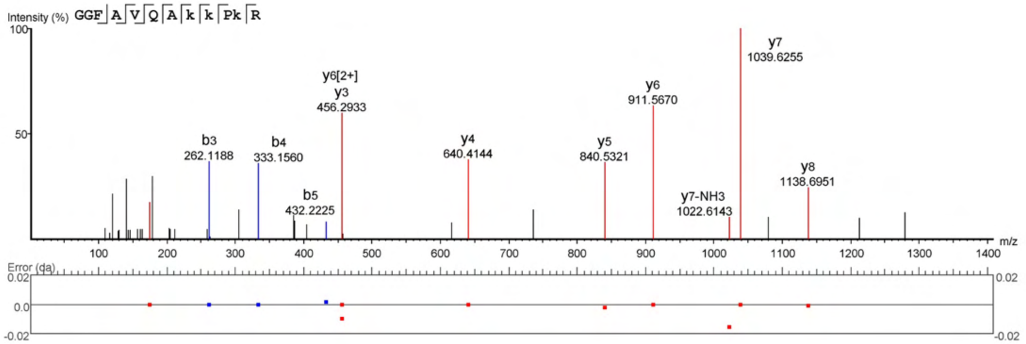


| # | b | b-H2O | b-NH3 | b (2+) | Seq | y | y-H2O | y-NH3 | y (2+) | # |
|---|---|---|---|---|---|---|---|---|---|---|
| 1 | 58.0293 | 40.0187 | 41.0023 | 29.5146 | G | | | | | 12 |
| 2 | 115.0508 | 97.0402 | 98.0238 | 58.0254 | G | 1413.8212 | 1395.8107 | 1396.7942 | 707.4106 | 11 |
| 3 | 262.1188 | 244.1086 | 245.0921 | 131.5596 | F | 1356.7997 | 1338.7892 | 1339.7727 | 678.8998 | 10 |
| 4 | 333.1560 | 315.1457 | 316.1293 | 167.0781 | A | 1209.7313 | 1191.7208 | 1192.7043 | 605.3657 | 9 |
| 5 | 432.2225 | 414.2141 | 415.1977 | 216.6123 | V | 1138.6951 | 1120.6837 | 1121.6672 | 569.8471 | 8 |
| 6 | 560.2833 | 542.2727 | 543.2563 | 280.6416 | Q | 1039.6255 | 1021.6152 | 1022.6143 | 520.3129 | 7 |
| 7 | 631.3204 | 613.3098 | 614.2934 | 316.1602 | A | 911.5670 | 893.5566 | 894.5402 | 456.2933 | 6 |
| 8 | 831.4365 | 813.4259 | 814.4095 | 416.2182 | K(+72.02) | 840.5321 | 822.5195 | 823.5031 | 420.7650 | 5 |
| 9 | 1015.5576 | 997.5471 | 998.5306 | 508.2788 | K(+56.03) | 640.4144 | 622.4034 | 623.3870 | 320.7070 | 4 |
| 10 | 1112.6104 | 1094.5999 | 1095.5834 | 556.8052 | P | 456.2933 | 438.2823 | 439.2659 | 228.6464 | 3 |
| 11 | 1296.7316 | 1278.7211 | 1279.7046 | 648.8658 | K(+56.03) | 359.2401 | 341.2296 | 342.2131 | 180.1201 | 2 |
| 12 | | | | | R | 175.1191 | 157.1084 | 158.0919 | 88.0595 | 1 |

Figure S14_31

**MSMS assignment of K55ox in Chromodomain-helicase-DNA-binding protein 3**

CHD3(53-66): GPK*[O]K*QK*ENK*PGK*PR; -10lgP 44.75; Scan 27652 (MC356)

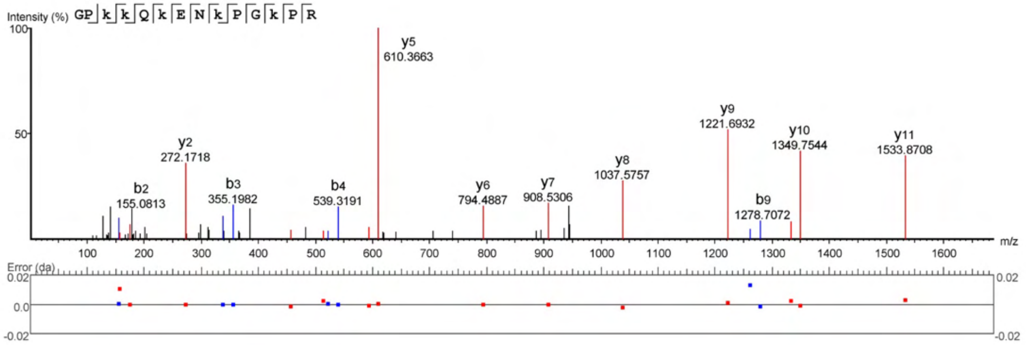


| # | b | b-H2O | b-NH3 | b (2+) | Seq | y | y-H2O | y-NH3 | y (2+) | # |
|---|---|---|---|---|---|---|---|---|---|---|
| 1 | 58.0293 | 40.0187 | 41.0023 | 29.5146 | G | | | | | 14 |
| 2 | 155.0813 | 137.0715 | 138.0550 | 78.0410 | P | 1831.0436 | 1813.0331 | 1814.0166 | 916.0218 | 13 |
| 3 | 355.1982 | 337.1872 | 338.1711 | 178.0991 | K(+72.02) | 1733.9907 | 1715.9802 | 1716.9637 | 867.4954 | 12 |
| 4 | 539.3191 | 521.3075 | 522.2923 | 270.1596 | K(+56.03) | 1533.8708 | 1515.8641 | 1516.8477 | 767.4373 | 11 |
| 5 | 667.3779 | 649.3673 | 650.3509 | 334.1889 | Q | 1349.7544 | 1331.7430 | 1332.7234 | 675.3768 | 10 |
| 6 | 851.4990 | 833.4885 | 834.4720 | 426.2495 | K(+56.03) | 1221.6932 | 1203.6844 | 1204.6680 | 611.3475 | 9 |
| 7 | 980.5416 | 962.5311 | 963.5146 | 490.7708 | E | 1037.5757 | 1019.5632 | 1020.5468 | 519.2869 | 8 |
| 8 | 1094.5846 | 1076.5741 | 1077.5576 | 547.7923 | N | 908.5306 | 890.5206 | 891.5042 | 454.7656 | 7 |
| 9 | 1278.7072 | 1260.6813 | 1261.6787 | 639.8528 | K(+56.03) | 794.4887 | 776.4777 | 777.4612 | 397.7441 | 6 |
| 10 | 1375.7585 | 1357.7480 | 1358.7316 | 688.3793 | P | 610.3663 | 592.3565 | 593.3411 | 305.6835 | 5 |
| 11 | 1432.7799 | 1414.7694 | 1415.7529 | 716.8900 | G | 513.3114 | 495.3038 | 496.2874 | 257.1572 | 4 |
| 12 | 1616.9011 | 1598.8906 | 1599.8741 | 808.9506 | K(+56.03) | 456.2947 | 438.2823 | 439.2659 | 228.6464 | 3 |
| 13 | 1713.9539 | 1695.9434 | 1696.9269 | 857.4769 | P | 272.1718 | 254.1611 | 255.1447 | 136.5858 | 2 |
| 14 | | | | | R | 175.1188 | 157.0974 | 158.0919 | 88.0595 | 1 |

Figure S14_32

**MSMS assignment of K67ox in Chromodomain-helicase-DNA-binding protein 4**

CHD4(62-70): DPK*IPK*[O]SK*R; -10lgP 32.94; Scan 23506 (MC355)

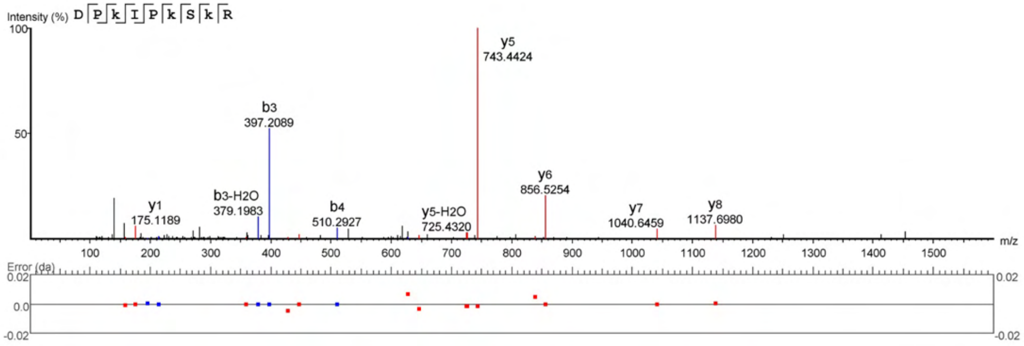


| # | b | b-H2O | b-NH3 | b (2+) | Seq | y | y-H2O | y-NH3 | y (2+) | # |
|---|---|---|---|---|---|---|---|---|---|---|
| 1 | 116.0348 | 98.0242 | 99.0078 | 58.5174 | D | | | | | 9 |
| 2 | 213.0872 | 195.0763 | 196.0605 | 107.0438 | P | 1137.6980 | 1119.6885 | 1120.6720 | 569.3495 | 8 |
| 3 | 397.2089 | 379.1983 | 380.1817 | 199.1043 | K(+56.03) | 1040.6459 | 1022.6356 | 1023.6191 | 520.8231 | 7 |
| 4 | 510.2927 | 492.2822 | 493.2657 | 255.6464 | I | 856.5254 | 838.5092 | 839.4980 | 428.7670 | 6 |
| 5 | 607.3455 | 589.3350 | 590.3185 | 304.1728 | P | 743.4424 | 725.4320 | 726.4156 | 372.2205 | 5 |
| 6 | 807.4616 | 789.4510 | 790.4346 | 404.2308 | K(+72.02) | 646.3915 | 628.3776 | 629.3612 | 323.6941 | 4 |
| 7 | 894.4936 | 876.4830 | 877.4666 | 447.7468 | S | 446.2727 | 428.2616 | 429.2451 | 223.6361 | 3 |
| 8 | 1078.6147 | 1060.6042 | 1061.5878 | 539.8074 | K(+56.03) | 359.2398 | 341.2296 | 342.2131 | 180.1201 | 2 |
| 9 | | | | | R | 175.1189 | 157.1084 | 158.0927 | 88.0595 | 1 |

**Figure S14_33**

**MSMS assignment of K993ox in RNA polymerase-associated protein CTR9 homolog**

CTR9(985-999): RPPK*AEK*K*K*[O]APK*PER; -10lgP 42.79; Scan 23004 (MC355)

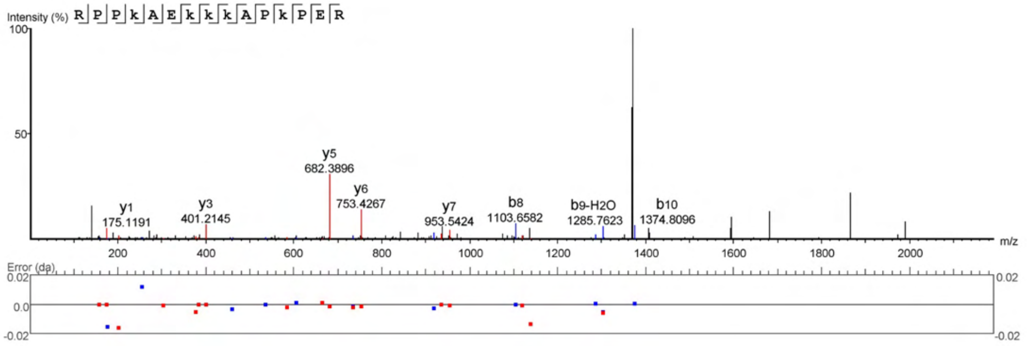


| # | b | b-H2O | b-NH3 | b (2+) | Seq | y | y-H2O | y-NH3 | y (2+) | # |
|---|---|---|---|---|---|---|---|---|---|---|
| 1 | 157.1085 | 139.0984 | 140.0819 | 79.0545 | R | | | | | 15 |
| 2 | 254.1493 | 236.1511 | 237.1347 | 127.5808 | P | 1900.0901 | 1882.0796 | 1883.0631 | 950.5450 | 14 |
| 3 | 351.2144 | 333.2039 | 334.1874 | 176.1226 | P | 1803.0374 | 1785.0269 | 1786.0104 | 902.0187 | 13 |
| 4 | 535.3361 | 517.3250 | 518.3086 | 268.1678 | K(+56.03) | 1705.9846 | 1687.9741 | 1688.9576 | 853.4923 | 12 |
| 5 | 606.3709 | 588.3621 | 589.3457 | 303.6863 | A | 1521.8634 | 1503.8529 | 1504.8364 | 761.4317 | 11 |
| 6 | 735.4172 | 717.4047 | 718.3883 | 368.2076 | E | 1450.8263 | 1432.8158 | 1433.7993 | 725.9131 | 10 |
| 7 | 919.5397 | 901.5259 | 902.5095 | 460.2715 | K(+56.03) | 1321.7837 | 1303.7793 | 1304.7567 | 661.3918 | 9 |
| 8 | 1103.6582 | 1085.6471 | 1086.6306 | 552.3288 | K(+56.03) | 1137.6763 | 1119.6531 | 1120.6356 | 569.3313 | 8 |
| 9 | 1303.7793 | 1285.7623 | 1286.7467 | 652.3868 | K(+72.02) | 953.5424 | 935.5312 | 936.5144 | 477.2707 | 7 |
| 10 | 1374.8096 | 1356.8003 | 1357.7838 | 687.9054 | A | 753.4267 | 735.4172 | 736.3983 | 377.2178 | 6 |
| 11 | 1471.8636 | 1453.8531 | 1454.8367 | 736.4318 | P | 682.3896 | 664.3776 | 665.3595 | 341.6941 | 5 |
| 12 | 1655.9847 | 1637.9742 | 1638.9578 | 828.4924 | K(+56.03) | 585.3375 | 567.3249 | 568.3085 | 293.1677 | 4 |
| 13 | 1753.0375 | 1735.0270 | 1736.0105 | 877.0187 | P | 401.2145 | 383.2037 | 384.1877 | 201.1234 | 3 |
| 14 | 1882.0801 | 1864.0696 | 1865.0531 | 941.5400 | E | 304.1621 | 286.1509 | 287.1345 | 152.5807 | 2 |
| 15 | | | | | R | 175.1191 | 157.1085 | 158.0919 | 88.0595 | 1 |

**Figure S14_34**

**MSMS assignment of K18ox in Pre-mRNA-splicing factor ATP-dependent RNA helicase DHX15**

DHX15(7-19): LDLGEDYPSGK*K*[O]R; -10lgP 34.92; Scan 33966 (MC362)

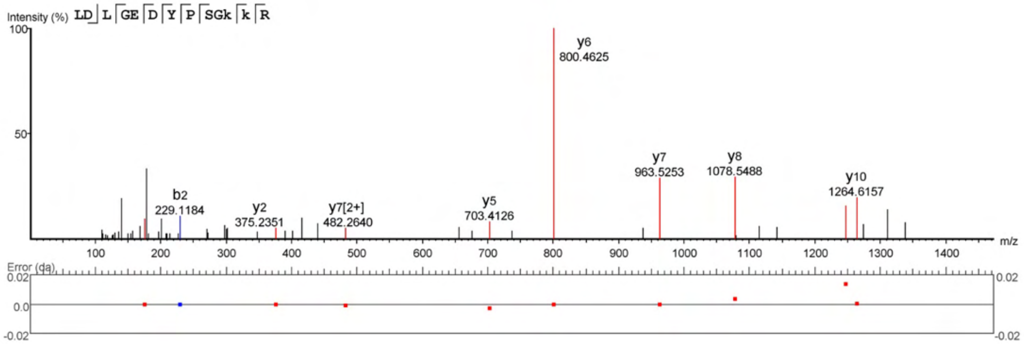


| # | b | b-H2O | b-NH3 | b (2+) | Seq | y | y-H2O | y-NH3 | y (2+) | # |
|---|---|---|---|---|---|---|---|---|---|---|
| 1 | 114.0919 | 96.0813 | 97.0649 | 57.5459 | L | | | | | 13 |
| 2 | 229.1184 | 211.1083 | 212.0918 | 115.0594 | D | 1492.7278 | 1474.7173 | 1475.7008 | 746.8639 | 12 |
| 3 | 342.2029 | 324.1923 | 325.1759 | 171.6014 | L | 1377.7008 | 1359.6903 | 1360.6738 | 689.3504 | 11 |
| 4 | 399.2243 | 381.2138 | 382.1974 | 200.1122 | G | 1264.6157 | 1246.5920 | 1247.5897 | 632.8083 | 10 |
| 5 | 528.2669 | 510.2563 | 511.2399 | 264.6335 | E | 1207.5953 | 1189.5848 | 1190.5684 | 604.2977 | 9 |
| 6 | 643.2939 | 625.2833 | 626.2669 | 322.1469 | D | 1078.5488 | 1060.5422 | 1061.5258 | 539.7764 | 8 |
| 7 | 806.3572 | 788.3466 | 789.3302 | 403.6786 | Y | 963.5253 | 945.5152 | 946.4988 | 482.2640 | 7 |
| 8 | 903.4099 | 885.3994 | 886.3829 | 452.2050 | P | 800.4625 | 782.4518 | 783.4354 | 400.7312 | 6 |
| 9 | 990.4420 | 972.4314 | 973.4150 | 495.7210 | S | 703.4126 | 685.3991 | 686.3827 | 352.2048 | 5 |
| 10 | 1047.4634 | 1029.4529 | 1030.4364 | 524.2317 | G | 616.3776 | 598.3671 | 599.3506 | 308.6888 | 4 |
| 11 | 1231.5846 | 1213.5741 | 1214.5576 | 616.2923 | K(+56.03) | 559.3562 | 541.3456 | 542.3292 | 280.1781 | 3 |
| 12 | 1431.7007 | 1413.6902 | 1414.6737 | 716.3503 | K(+72.02) | 375.2351 | 357.2245 | 358.2080 | 188.1175 | 2 |
| 13 | | | | | R | 175.1190 | 157.1084 | 158.0919 | 88.0595 | 1 |

**Figure S14_35**

**MSMS assignment of K475ox in H/ACA ribonucleoprotein complex subunit DKC1**

DKC(473-480): SK*K*[O]DK*K*AK; -10lgP 27.46; Scan 8697 (MC309)

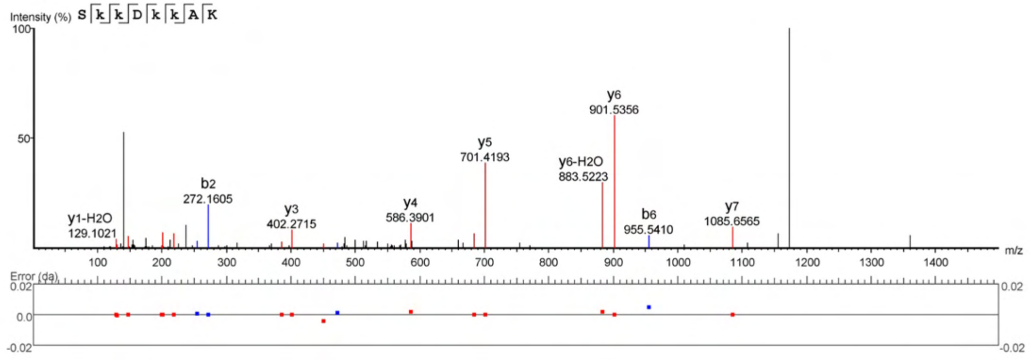


| # | b | b-H2O | b-NH3 | b (2+) | Seq | y | y-H2O | y-NH3 | y (2+) | # |
|---|---|---|---|---|---|---|---|---|---|---|
| 1 | 88.0399 | 70.0293 | 71.0129 | 44.5199 | S | | | | | 8 |
| 2 | 272.1605 | 254.1498 | 255.1340 | 136.5805 | K(+56.03) | 1085.6565 | 1067.6459 | 1068.6294 | 543.3282 | 7 |
| 3 | 472.2758 | 454.2665 | 455.2501 | 236.6385 | K(+72.02) | 901.5356 | 883.5223 | 884.5083 | 451.2719 | 6 |
| 4 | 587.3040 | 569.2935 | 570.2770 | 294.1520 | D | 701.4193 | 683.4086 | 684.3928 | 351.2096 | 5 |
| 5 | 771.4252 | 753.4147 | 754.3983 | 386.2126 | K(+56.03) | 586.3901 | 568.3817 | 569.3652 | 293.6961 | 4 |
| 6 | 955.5410 | 937.5358 | 938.5194 | 478.2732 | K(+56.03) | 402.2715 | 384.2605 | 385.2439 | 201.6355 | 3 |
| 7 | 1026.5835 | 1008.5729 | 1009.5565 | 513.7917 | A | 218.1502 | 200.1391 | 201.1233 | 109.5750 | 2 |
| 8 | | | | | K | 147.1124 | 129.1021 | 130.0864 | 74.0564 | 1 |

**Figure S14_36**

**MSMS assignment of K90ox in Protein FAM133B**

FAM133B(78-91): EK*LLSGSESSSK*K*[O]R; -10lgP 51.19; Scan 13718 (MC315)

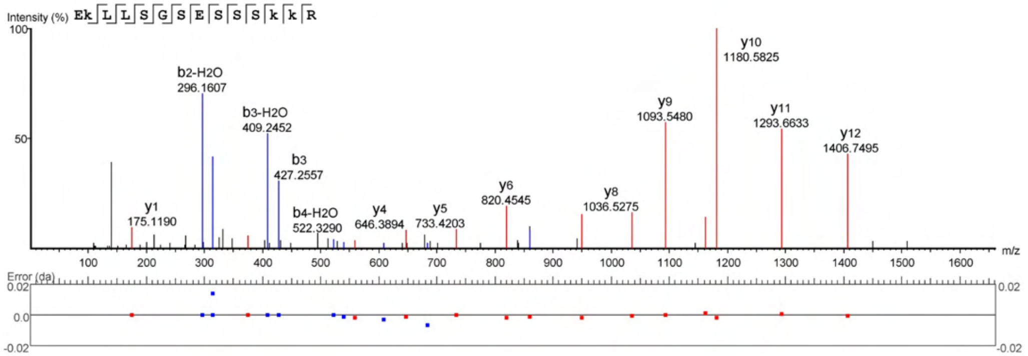


| # | b | b-H2O | b-NH3 | b (2+) | Seq | y | y-H2O | y-NH3 | y (2+) | # |
|---|---|---|---|---|---|---|---|---|---|---|
| 1 | 130.0504 | 112.0399 | 113.0234 | 65.5252 | E | | | | | 14 |
| 2 | 314.1714 | 296.1607 | 297.1446 | 157.5858 | K(+56.03) | 1590.8696 | 1572.8591 | 1573.8427 | 795.9348 | 13 |
| 3 | 427.2557 | 409.2452 | 410.2286 | 214.1278 | L | 1406.7495 | 1388.7380 | 1389.7216 | 703.8743 | 12 |
| 4 | 540.3412 | 522.3290 | 523.3127 | 270.6699 | L | 1293.6633 | 1275.6539 | 1276.6375 | 647.3322 | 11 |
| 5 | 627.3717 | 609.3643 | 610.3447 | 314.1714 | S | 1180.5825 | 1162.5684 | 1163.5533 | 590.7902 | 10 |
| 6 | 684.4003 | 666.3826 | 667.3662 | 342.6966 | G | 1093.5480 | 1075.5378 | 1076.5214 | 547.2742 | 9 |
| 7 | 771.4252 | 753.4147 | 754.3983 | 386.2126 | S | 1036.5275 | 1018.5163 | 1019.4999 | 518.7634 | 8 |
| 8 | 900.4678 | 882.4572 | 883.4408 | 450.7339 | E | 949.4969 | 931.4843 | 932.4679 | 475.2474 | 7 |
| 9 | 987.4998 | 969.4893 | 970.4728 | 494.2499 | S | 820.4545 | 802.4417 | 803.4253 | 410.7261 | 6 |
| 10 | 1074.5319 | 1056.5214 | 1057.5049 | 537.7659 | S | 733.4203 | 715.4097 | 716.3932 | 367.2101 | 5 |
| 11 | 1161.5638 | 1143.5533 | 1144.5369 | 581.2819 | S | 646.3894 | 628.3776 | 629.3612 | 323.6941 | 4 |
| 12 | 1345.6851 | 1327.6746 | 1328.6581 | 673.3425 | K(+56.03) | 559.3580 | 541.3456 | 542.3292 | 280.1781 | 3 |
| 13 | 1545.8011 | 1527.7906 | 1528.7742 | 773.4006 | K(+72.02) | 375.2356 | 357.2245 | 358.2080 | 188.1175 | 2 |
| 14 | | | | | R | 175.1190 | 157.1084 | 158.0919 | 88.0595 | 1 |

**Figure S14_37**

**MSMS assignment of K208ox, K209ox, K210ox in Protein FAM133B**

FAM133B(206-215): **AK*K*[O]K*[O]K*[O]SSEER**; -10lgP 34.34; Scan 6424 (MC309)

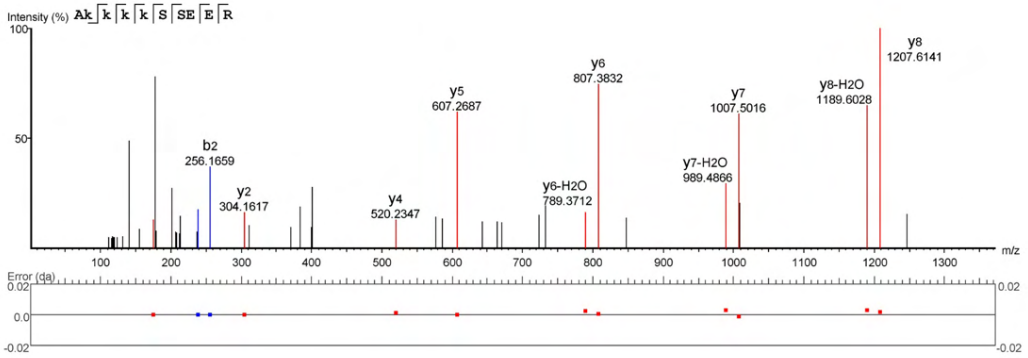


| # | b | b-H2O | b-NH3 | b (2+) | Seq | y | y-H2O | y-NH3 | y (2+) | # |
|---|---|---|---|---|---|---|---|---|---|---|
| 1 | 72.0449 | 54.0344 | 55.0179 | 36.5225 | A | | | | | 10 |
| 2 | 256.1659 | 238.1555 | 239.1389 | 128.5831 | K(+56.03) | 1391.7375 | 1373.7271 | 1374.7106 | 696.3688 | 9 |
| 3 | 456.2822 | 438.2716 | 439.2552 | 228.6411 | K(+72.02) | 1207.6141 | 1189.6028 | 1190.5895 | 604.3082 | 8 |
| 4 | 656.3983 | 638.3877 | 639.3713 | 328.6991 | K(+72.02) | 1007.5016 | 989.4866 | 990.4734 | 504.2502 | 7 |
| 5 | 856.5143 | 838.5038 | 839.4874 | 428.7572 | K(+72.02) | 807.3832 | 789.3712 | 790.3573 | 404.1921 | 6 |
| 6 | 943.5464 | 925.5358 | 926.5194 | 472.2732 | S | 607.2687 | 589.2576 | 590.2412 | 304.1341 | 5 |
| 7 | 1030.5784 | 1012.5678 | 1013.5514 | 515.7892 | S | 520.2347 | 502.2256 | 503.2092 | 260.6181 | 4 |
| 8 | 1159.6210 | 1141.6105 | 1142.5940 | 580.3105 | E | 433.2041 | 415.1935 | 416.1771 | 217.1021 | 3 |
| 9 | 1288.6636 | 1270.6531 | 1271.6366 | 644.8318 | E | 304.1617 | 286.1509 | 287.1345 | 152.5807 | 2 |
| 10 | | | | | R | 175.1192 | 157.1084 | 158.0919 | 88.0595 | 1 |

Figure S14_38

**MSMS assignment of K564ox, K566ox, K567ox, K569ox in Pre-mRNA 3'-end-processing factor FIP1**

FIP1L1(563-570): HK*[O]HK*[O]K*[O]SK*[O]R; -10lgP 37.45; Scan 4737 (MC308)

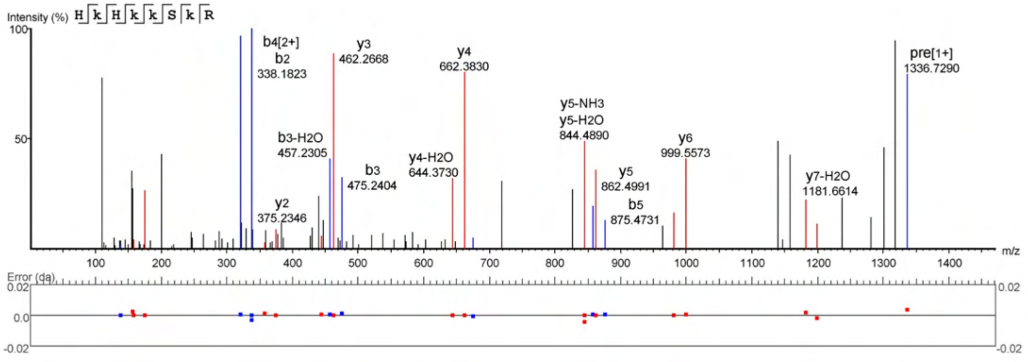


| # | b | b-H2O | b-NH3 | b (2+) | Seq | y | y-H2O | y-NH3 | y (2+) | # |
|---|---|---|---|---|---|---|---|---|---|---|
| 1 | 138.0664 | 120.0562 | 121.0397 | 69.5334 | H | | | | | 8 |
| 2 | 338.1823 | 320.1715 | 321.1558 | 169.5914 | K(+72.02) | 1199.6763 | 1181.6614 | 1182.6472 | 600.3371 | 7 |
| 3 | 475.2404 | 457.2305 | 458.2148 | 238.1209 | H | 999.5573 | 981.5475 | 982.5311 | 500.2791 | 6 |
| 4 | 675.3588 | 657.3472 | 658.3308 | 338.1823 | K(+72.02) | 862.4991 | 844.4890 | 845.4769 | 431.7496 | 5 |
| 5 | 875.4731 | 857.4622 | 858.4469 | 438.2369 | K(+72.02) | 662.3830 | 644.3730 | 645.3561 | 331.6916 | 4 |
| 6 | 962.5059 | 944.4954 | 945.4789 | 481.7530 | S | 462.2668 | 444.2559 | 445.2401 | 231.6335 | 3 |
| 7 | 1162.6221 | 1144.6116 | 1145.5951 | 581.8110 | K(+72.02) | 375.2346 | 357.2245 | 358.2063 | 188.1175 | 2 |
| 8 | | | | | R | 175.1188 | 157.1055 | 158.0919 | 88.0595 | 1 |

**Figure S14_39**

**Non-unique MSMS assignment:** K*SK*[O]DK*[O]K*[O]R; -10lgP 15.85; Scan 6660 (MC305)
**1. K27ox, K29ox, K30ox in Protein FRG1; FRG1(25-31)**
**2. K241ox, K242ox, K244ox in Serine/arginine repetitive matrix protein 2; SRRM2(239-245)**

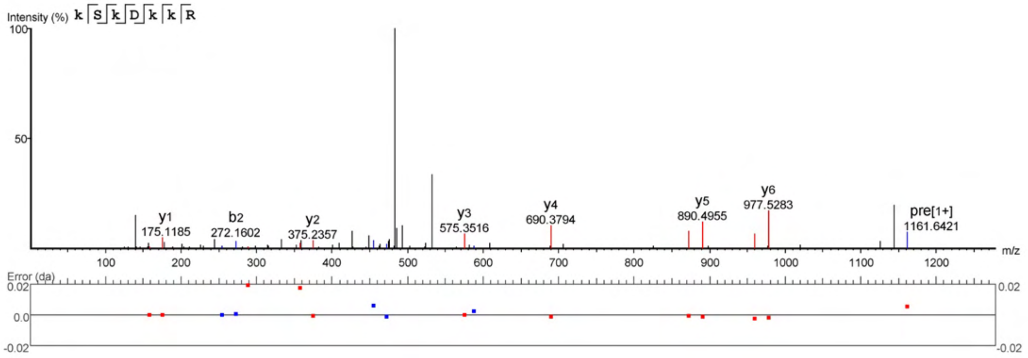


| # | b | b-H2O | b-NH3 | b (2+) | Seq | y | y-H2O | y-NH3 | y (2+) | # |
|---|---|---|---|---|---|---|---|---|---|---|
| 1 | 185.1290 | 167.1184 | 168.1020 | 93.0645 | K(+56.03) | | | | | 7 |
| 2 | 272.1602 | 254.1501 | 255.1340 | 136.5805 | S | 977.5283 | 959.5181 | 960.4992 | 489.2631 | 6 |
| 3 | 472.2786 | 454.2600 | 455.2501 | 236.6385 | K(+72.02) | 890.4955 | 872.4845 | 873.4672 | 445.7471 | 5 |
| 4 | 587.3014 | 569.2935 | 570.2770 | 294.1520 | D | 690.3794 | 672.3675 | 673.3511 | 345.6890 | 4 |
| 5 | 787.4201 | 769.4095 | 770.3931 | 394.2101 | K(+72.02) | 575.3516 | 557.3405 | 558.3241 | 288.1558 | 3 |
| 6 | 987.5362 | 969.5256 | 970.5092 | 494.2681 | K(+72.02) | 375.2357 | 357.2064 | 358.2080 | 188.1175 | 2 |
| 7 | | | | | R | 175.1185 | 157.1084 | 158.0919 | 88.0595 | 1 |

Figure S14_40

**MSMS assignment of K20ox, K23ox in Guanine nucleotide-binding protein-like 3**
GNL3(19-26): YK*[O]IQK*[O]K*VR; -10lgP 26.57; Scan 12215 (MC309)

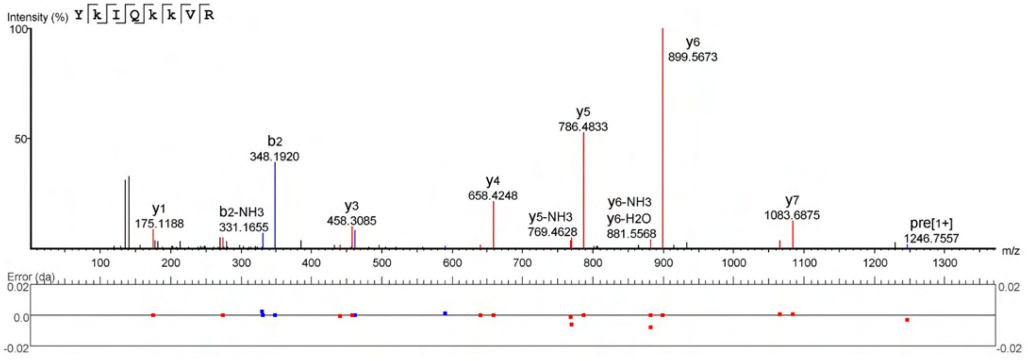


| # | b | b-H2O | b-NH3 | b (2+) | Seq | y | y-H2O | y-NH3 | y (2+) | # |
|---|---|---|---|---|---|---|---|---|---|---|
| 1 | 164.0712 | 146.0606 | 147.0441 | 82.5356 | Y | | | | | 8 |
| 2 | 348.1920 | 330.1790 | 331.1655 | 174.5962 | K(+56.03) | 1083.6875 | 1065.6770 | 1066.6614 | 542.3442 | 7 |
| 3 | 461.2763 | 443.2658 | 444.2494 | 231.1382 | I | 899.5673 | 881.5568 | 882.5487 | 450.2836 | 6 |
| 4 | 589.3336 | 571.3244 | 572.3080 | 295.1675 | Q | 786.4833 | 768.4741 | 769.4628 | 393.7416 | 5 |
| 5 | 789.4510 | 771.4405 | 772.4241 | 395.2255 | K(+72.02) | 658.4248 | 640.4139 | 641.3976 | 329.7123 | 4 |
| 6 | 973.5722 | 955.5616 | 956.5452 | 487.2861 | K(+56.03) | 458.3085 | 440.2979 | 441.2827 | 229.6543 | 3 |
| 7 | 1072.6406 | 1054.6301 | 1055.6136 | 536.8203 | V | 274.1875 | 256.1768 | 257.1604 | 137.5937 | 2 |
| 8 | | | | | R | 175.1188 | 157.1084 | 158.0919 | 88.0595 | 1 |

**Figure S14_41**

**MSMS assignment of K221ox in Guanine nucleotide-binding protein-like 3**

GNL3(220-230): VK*[O]AK*K*NAAPFR; -10lgP 25.05 Scan 27049 (MC361)

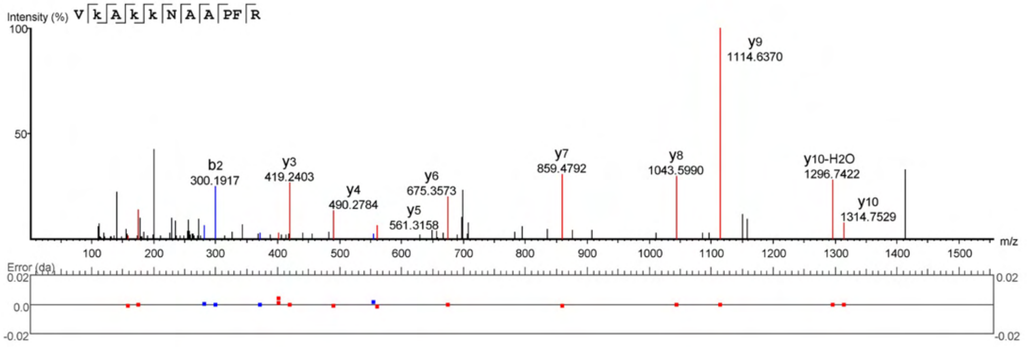


| # | b | b-H2O | b-NH3 | b (2+) | Seq | y | y-H2O | y-NH3 | y (2+) | # |
|---|---|---|---|---|---|---|---|---|---|---|
| 1 | 100.0762 | 82.0657 | 83.0492 | 50.5381 | V | | | | | 11 |
| 2 | 300.1917 | 282.1811 | 283.1653 | 150.5962 | K(+72.02) | 1314.7529 | 1296.7422 | 1297.7258 | 657.8764 | 10 |
| 3 | 371.2299 | 353.2189 | 354.2025 | 186.1147 | A | 1114.6370 | 1096.6262 | 1097.6097 | 557.8184 | 9 |
| 4 | 555.3486 | 537.3400 | 538.3236 | 278.1753 | K(+56.03) | 1043.5990 | 1025.5891 | 1026.5726 | 522.2998 | 8 |
| 5 | 739.4717 | 721.4612 | 722.4448 | 370.2359 | K(+56.03) | 859.4792 | 841.4678 | 842.4514 | 430.2392 | 7 |
| 6 | 853.5147 | 835.5042 | 836.4877 | 427.2574 | N | 675.3573 | 657.3467 | 658.3303 | 338.1786 | 6 |
| 7 | 924.5518 | 906.5413 | 907.5248 | 462.7759 | A | 561.3158 | 543.3038 | 544.2874 | 281.1572 | 5 |
| 8 | 995.5889 | 977.5784 | 978.5620 | 498.2945 | A | 490.2784 | 472.2666 | 473.2502 | 245.6386 | 4 |
| 9 | 1092.6417 | 1074.6312 | 1075.6147 | 546.8209 | P | 419.2403 | 401.2247 | 402.2115 | 210.1200 | 3 |
| 10 | 1239.7101 | 1221.6996 | 1222.6831 | 620.3550 | F | 322.1873 | 304.1768 | 305.1603 | 161.5937 | 2 |
| 11 | | | | | R | 175.1188 | 157.1084 | 158.0931 | 88.0595 | 1 |

**Figure S14_42**

**MSMS assignment of K248ox in Heat shock cognate 71 kDa protein**

HSPA8(248-258): K*[O]HK*K*DISENK*R; -10lgP 36.09; Scan 9710 (MC309)

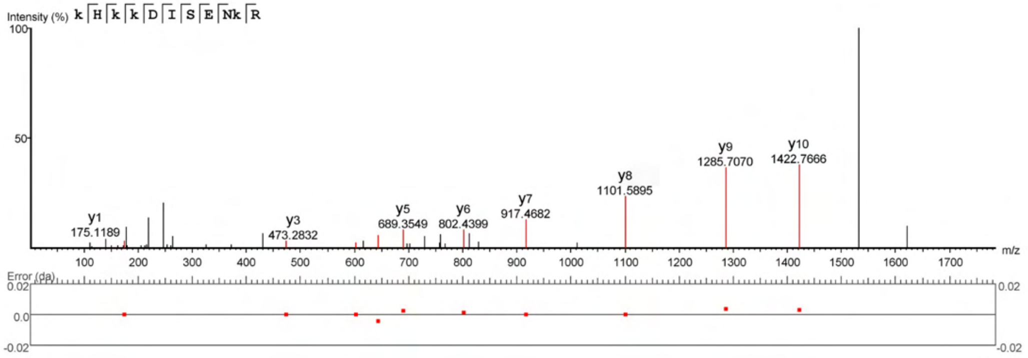


| # | b | b-H2O | b-NH3 | b (2+) | Seq | y | y-H2O | y-NH3 | y (2+) | # |
|---|---|---|---|---|---|---|---|---|---|---|
| 1 | 201.1239 | 183.1134 | 184.0969 | 101.0620 | K(+72.02) | | | | | 11 |
| 2 | 338.1828 | 320.1722 | 321.1558 | 169.5914 | H | 1422.7666 | 1404.7594 | 1405.7429 | 711.8849 | 10 |
| 3 | 522.3040 | 504.2934 | 505.2770 | 261.6520 | K(+56.03) | 1285.7070 | 1267.7004 | 1268.6840 | 643.3599 | 9 |
| 4 | 706.4252 | 688.4146 | 689.3982 | 353.7126 | K(+56.03) | 1101.5895 | 1083.5793 | 1084.5629 | 551.2949 | 8 |
| 5 | 821.4521 | 803.4415 | 804.4251 | 411.2260 | D | 917.4682 | 899.4581 | 900.4417 | 459.2343 | 7 |
| 6 | 934.5361 | 916.5256 | 917.5092 | 467.7681 | I | 802.4399 | 784.4312 | 785.4147 | 401.7209 | 6 |
| 7 | 1021.5682 | 1003.5576 | 1004.5412 | 511.2841 | S | 689.3549 | 671.3471 | 672.3307 | 345.1788 | 5 |
| 8 | 1150.6107 | 1132.6002 | 1133.5837 | 575.8054 | E | 602.3257 | 584.3151 | 585.2986 | 301.6628 | 4 |
| 9 | 1264.6537 | 1246.6432 | 1247.6267 | 632.8268 | N | 473.2832 | 455.2725 | 456.2560 | 237.1415 | 3 |
| 10 | 1448.7749 | 1430.7644 | 1431.7479 | 724.8875 | K(+56.03) | 359.2401 | 341.2296 | 342.2131 | 180.1201 | 2 |
| 11 | | | | | R | 175.1189 | 157.1084 | 158.0919 | 88.0595 | 1 |

Figure S14_43

**MSMS assignment for K219ox in Bifunctional arginine demethylase and lysyl-hydroxylase JMJD6**
JMJD6(216-222): ELIK*[O]VTR; -10lgP 29.89; Scan 11429 (MC317)

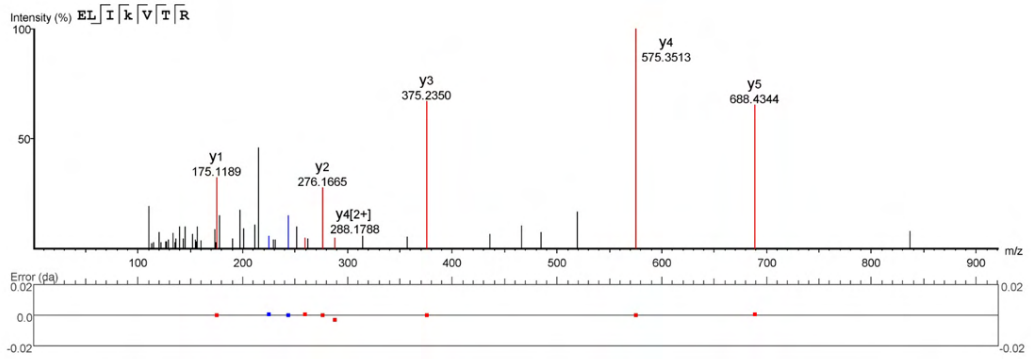


| # | b | b-H2O | b-NH3 | b (2+) | Seq | y | y-H2O | y-NH3 | y (2+) | # |
|---|---|---|---|---|---|---|---|---|---|---|
| 1 | 130.0504 | 112.0399 | 113.0234 | 65.5252 | E | | | | | 7 |
| 2 | 243.1339 | 225.1232 | 226.1075 | 122.0672 | L | 801.5192 | 783.5087 | 784.4922 | 401.2596 | 6 |
| 3 | 356.2185 | 338.2079 | 339.1915 | 178.6093 | I | 688.4344 | 670.4246 | 671.4082 | 344.7176 | 5 |
| 4 | 556.3346 | 538.3240 | 539.3076 | 278.6673 | K(+72.02) | 575.3513 | 557.3406 | 558.3242 | 288.1788 | 4 |
| 5 | 655.4030 | 637.3925 | 638.3760 | 328.2015 | V | 375.2350 | 357.2245 | 358.2080 | 188.1175 | 3 |
| 6 | 756.4507 | 738.4401 | 739.4237 | 378.7253 | T | 276.1665 | 258.1561 | 259.1387 | 138.5833 | 2 |
| 7 | | | | | R | 175.1189 | 157.1084 | 158.0919 | 88.0595 | 1 |

Figure S14_44

## MSMS assignment of K307ox in Bifunctional arginine demethylase and lysyl-hydroxylase JMJD6

JMJD6(304-310): GRPK*[O]LSR; -10lgP 34.77; Scan 3885 (MC320)

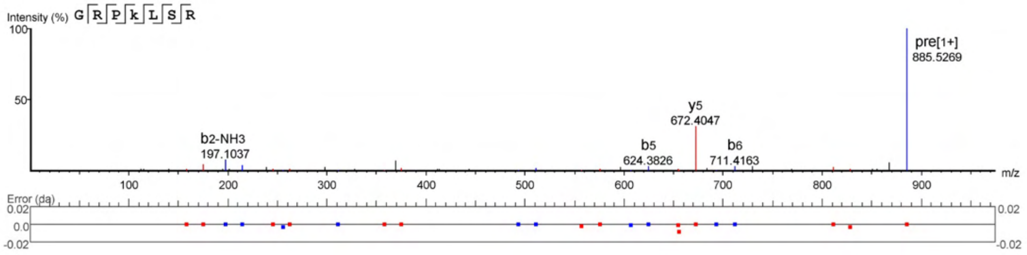


| # | b | b-H2O | b-NH3 | b (2+) | Seq | y | y-H2O | y-NH3 | y (2+) | # |
|---|---|---|---|---|---|---|---|---|---|---|
| 1 | 58.0293 | 40.0187 | 41.0023 | 29.5146 | G | | | | | 7 |
| 2 | 214.1300 | 196.1198 | 197.1037 | 107.5652 | R | 828.5083 | 810.4944 | 811.4780 | 414.7525 | 6 |
| 3 | 311.1833 | 293.1726 | 294.1562 | 156.0916 | P | 672.4047 | 654.3952 | 655.3856 | 336.7019 | 5 |
| 4 | 511.2985 | 493.2892 | 494.2722 | 256.1534 | K(+72.02) | 575.3521 | 557.3434 | 558.3241 | 288.1755 | 4 |
| 5 | 624.3826 | 606.3746 | 607.3563 | 312.6917 | L | 375.2355 | 357.2245 | 358.2088 | 188.1175 | 3 |
| 6 | 711.4163 | 693.4056 | 694.3883 | 356.2076 | S | 262.1515 | 244.1404 | 245.1248 | 131.5755 | 2 |
| 7 | | | | | R | 175.1192 | 157.1084 | 158.0928 | 88.0595 | 1 |

**Figure S14_45**

**MSMS assignment of K360ox in La-related protein 7**

LARP7(358-366): K*HK*[O]K*K*HK*ER; -10lgP 18.52; Scan 3999 (MC275)

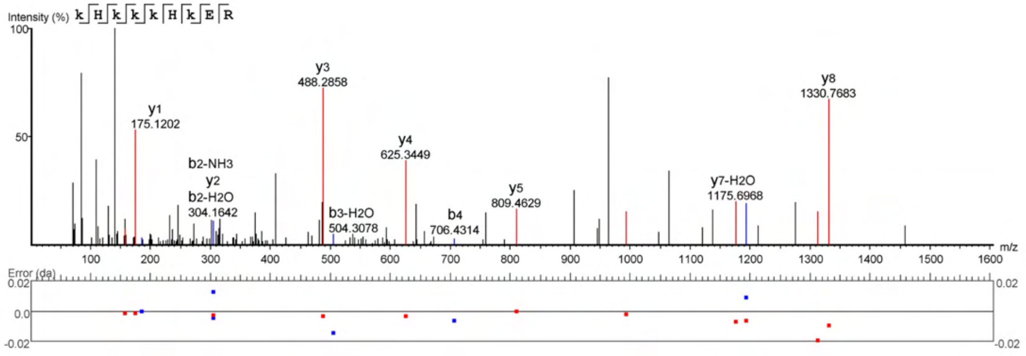


| # | b | b-H2O | b-NH3 | b (2+) | Seq | y | y-H2O | y-NH3 | y (2+) | # |
|---|---|---|---|---|---|---|---|---|---|---|
| 1 | 185.1295 | 167.1184 | 168.1020 | 93.0645 | K(+56.03) | | | | | 9 |
| 2 | 322.1879 | 304.1642 | 305.1656 | 161.5939 | H | 1330.7683 | 1312.7676 | 1313.7319 | 665.8795 | 8 |
| 3 | 522.3040 | 504.3078 | 505.2770 | 261.6520 | K(+72.02) | 1193.7063 | 1175.6968 | 1176.6731 | 597.3500 | 7 |
| 4 | 706.4314 | 688.4146 | 689.3982 | 353.7126 | K(+56.03) | 993.5858 | 975.5734 | 976.5569 | 497.2920 | 6 |
| 5 | 890.5463 | 872.5358 | 873.5193 | 445.7732 | K(+56.03) | 809.4629 | 791.4522 | 792.4358 | 405.2314 | 5 |
| 6 | 1027.6052 | 1009.5947 | 1010.5782 | 514.3026 | H | 625.3449 | 607.3311 | 608.3146 | 313.1708 | 4 |
| 7 | 1211.7264 | 1193.7063 | 1194.6995 | 606.3632 | K(+56.03) | 488.2858 | 470.2721 | 471.2557 | 244.6413 | 3 |
| 8 | 1340.7690 | 1322.7585 | 1323.7421 | 670.8845 | E | 304.1642 | 286.1509 | 287.1345 | 152.5807 | 2 |
| 9 | | | | | R | 175.1202 | 157.1084 | 158.0935 | 88.0595 | 1 |

Figure S14_46

**MSMS assignment of K341ox in Prelamin-A/C**

LMNA(337-343): LLAEK*[O]ER; -10lgP 22.86; Scan 9430 (MC306)

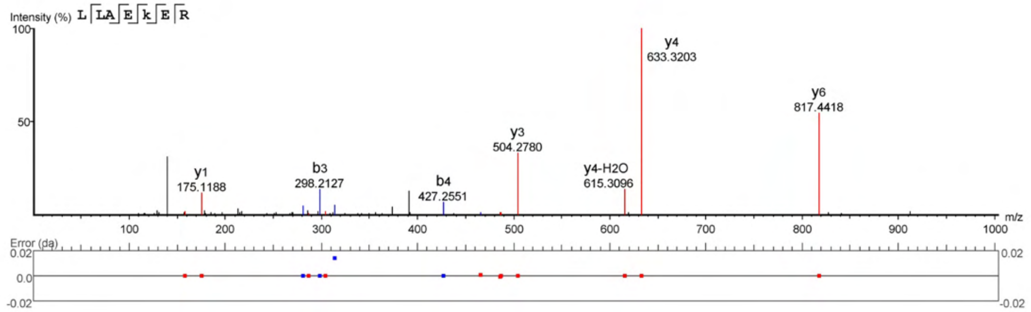


| # | b | b-H2O | b-NH3 | b (2+) | Seq | y | y-H2O | y-NH3 | y (2+) | # |
|---|---|---|---|---|---|---|---|---|---|---|
| 1 | 114.0919 | 96.0813 | 97.0649 | 57.5459 | L | | | | | 7 |
| 2 | 227.1759 | 209.1654 | 210.1489 | 114.0880 | L | 817.4418 | 799.4308 | 800.4144 | 409.2207 | 6 |
| 3 | 298.2127 | 280.2025 | 281.1859 | 149.6065 | A | 704.3573 | 686.3467 | 687.3303 | 352.6786 | 5 |
| 4 | 427.2551 | 409.2451 | 410.2286 | 214.1278 | E | 633.3203 | 615.3096 | 616.2932 | 317.1601 | 4 |
| 5 | 627.3717 | 609.3611 | 610.3447 | 314.1713 | K(+72.02) | 504.2780 | 486.2679 | 487.2504 | 252.6388 | 3 |
| 6 | 756.4143 | 738.4037 | 739.3873 | 378.7072 | E | 304.1617 | 286.1509 | 287.1351 | 152.5807 | 2 |
| 7 | | | | | R | 175.1188 | 157.1084 | 158.0924 | 88.0595 | 1 |

Figure S14_47

**MSMS assignment of K323ox, K325ox in Putative RNA-binding protein Luc7-like 1**

LUC7L(321-328): SAK*[O]YK*[O]FSR; -10lgP 36.68; Scan 9323 (MC317)

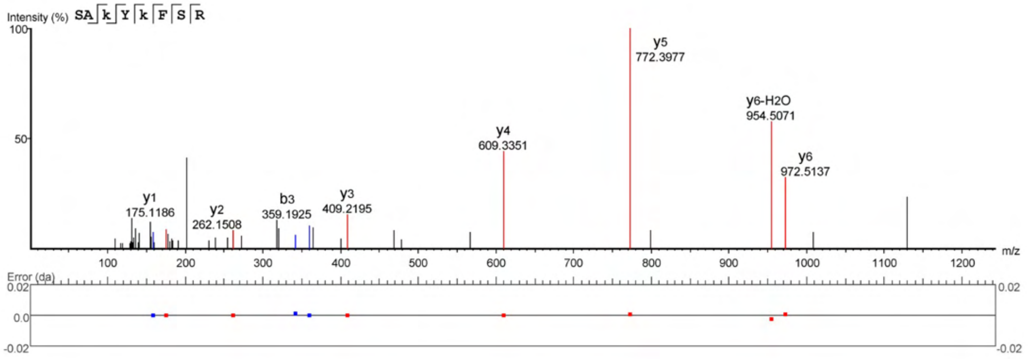


| # | b | b-H2O | b-NH3 | b (2+) | Seq | y | y-H2O | y-NH3 | y (2+) | # |
|---|---|---|---|---|---|---|---|---|---|---|
| 1 | 88.0399 | 70.0293 | 71.0129 | 44.5199 | S | | | | | 8 |
| 2 | 159.0766 | 141.0664 | 142.0500 | 80.0385 | A | 1043.5520 | 1025.5415 | 1026.5250 | 522.2760 | 7 |
| 3 | 359.1925 | 341.1811 | 342.1661 | 180.0965 | K(+72.02) | 972.5137 | 954.5071 | 955.4879 | 486.7574 | 6 |
| 4 | 522.2563 | 504.2458 | 505.2294 | 261.6282 | Y | 772.3977 | 754.3882 | 755.3718 | 386.6994 | 5 |
| 5 | 722.3724 | 704.3619 | 705.3455 | 361.6862 | K(+72.02) | 609.3351 | 591.3249 | 592.3085 | 305.1677 | 4 |
| 6 | 869.4409 | 851.4303 | 852.4139 | 435.2204 | F | 409.2195 | 391.2088 | 392.1924 | 205.1097 | 3 |
| 7 | 956.4729 | 938.4623 | 939.4459 | 478.7365 | S | 262.1508 | 244.1404 | 245.1240 | 131.5755 | 2 |
| 8 | | | | | R | 175.1186 | 157.1084 | 158.0919 | 88.0595 | 1 |

**Figure S14_48**

**MSMS assignment of K266ox, K269ox in Putative RNA-binding protein Luc7-like 2**

LUC7L2(263-270): SHSK*[O]NPK*[O]R; -10lgP 64.15; Scan 3234 (MC318)

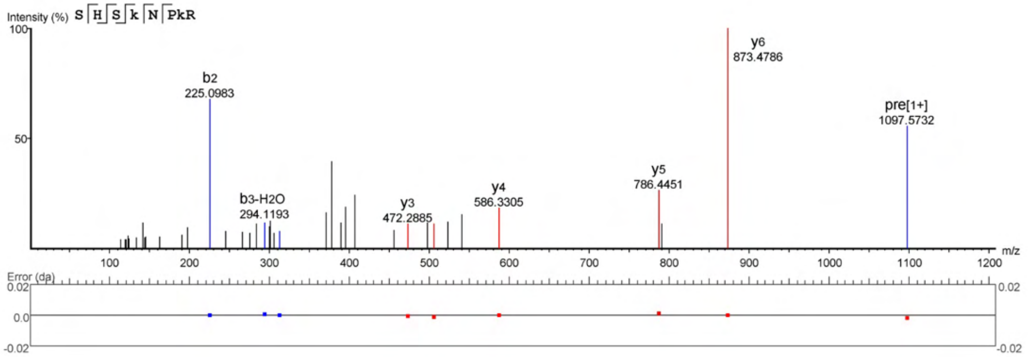


| # | b | b-H2O | b-NH3 | b (2+) | Seq | y | y-H2O | y-NH3 | y (2+) | # |
|---|---|---|---|---|---|---|---|---|---|---|
| 1 | 88.0399 | 70.0293 | 71.0129 | 44.5199 | S | | | | | 8 |
| 2 | 225.0983 | 207.0882 | 208.0718 | 113.0494 | H | 1010.5377 | 992.5272 | 993.5107 | 505.7702 | 7 |
| 3 | 312.1310 | 294.1193 | 295.1038 | 156.5654 | S | 873.4786 | 855.4683 | 856.4518 | 437.2394 | 6 |
| 4 | 512.2469 | 494.2363 | 495.2199 | 256.6234 | K(+72.02) | 786.4451 | 768.4362 | 769.4198 | 393.7234 | 5 |
| 5 | 626.2898 | 608.2792 | 609.2628 | 313.6449 | N | 586.3305 | 568.3201 | 569.3037 | 293.6653 | 4 |
| 6 | 723.3425 | 705.3320 | 706.3156 | 362.1713 | P | 472.2885 | 454.2772 | 455.2608 | 236.6439 | 3 |
| 7 | 923.4586 | 905.4481 | 906.4316 | 462.2293 | K(+72.02) | 375.2350 | 357.2245 | 358.2080 | 188.1175 | 2 |
| 8 | | | | | R | 175.1189 | 157.1084 | 158.0919 | 88.0595 | 1 |

**Figure S14_49**

**MSMS assignment of K365ox, K368ox in Putative RNA-binding protein Luc7-like 2**

LUC7L2(363-369): DRK*[O]DK*K*[O]R; -10lgP 26.30; Scan 5119 (MC312)

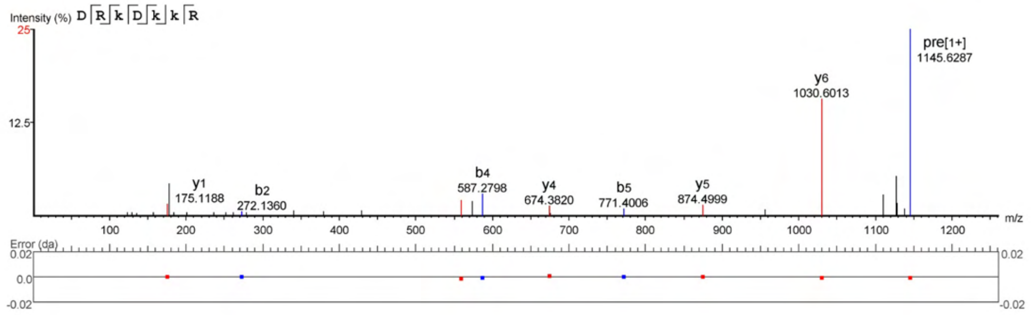


| # | b | b-H2O | b-NH3 | b (2+) | Seq | y | y-H2O | y-NH3 | y (2+) | # |
|---|---|---|---|---|---|---|---|---|---|---|
| 1 | 88.0399 | 70.0293 | 71.0129 | 44.5199 | S | | | | | 8 |
| 2 | 225.0983 | 207.0882 | 208.0718 | 113.0494 | H | 1010.5377 | 992.5272 | 993.5107 | 505.7702 | 7 |
| 3 | 312.1310 | 294.1193 | 295.1038 | 156.5654 | S | 873.4786 | 855.4683 | 856.4518 | 437.2394 | 6 |
| 4 | 512.2469 | 494.2363 | 495.2199 | 256.6234 | K(+72.02) | 786.4451 | 768.4362 | 769.4198 | 393.7234 | 5 |
| 5 | 626.2898 | 608.2792 | 609.2628 | 313.6449 | N | 586.3305 | 568.3201 | 569.3037 | 293.6653 | 4 |
| 6 | 723.3425 | 705.3320 | 706.3156 | 362.1713 | P | 472.2885 | 454.2772 | 455.2608 | 236.6439 | 3 |
| 7 | 923.4586 | 905.4481 | 906.4316 | 462.2293 | K(+72.02) | 375.2350 | 357.2245 | 358.2080 | 188.1175 | 2 |
| 8 | | | | | R | 175.1189 | 157.1084 | 158.0919 | 88.0595 | 1 |

**Figure S14_50**

**MSMS assignment of K248ox and K249ox in Luc7-like protein 3**

LUC7L3(247-257): LK*[O]K*[O]EK*QEREER; -10lgP 30.58; Scan 6968 (MC303)

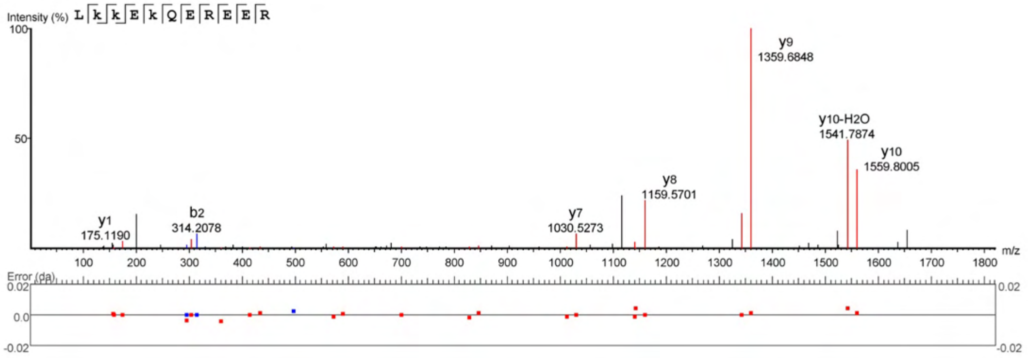


| # | b | b-H2O | b-NH3 | b (2+) | Seq | y | y-H2O | y-NH3 | y (2+) | # |
|---|---|---|---|---|---|---|---|---|---|---|
| 1 | 114.0919 | 96.0813 | 97.0649 | 57.5459 | L | | | | | 11 |
| 2 | 314.2078 | 296.1971 | 297.1810 | 157.6040 | K(+72.02) | 1559.8005 | 1541.7874 | 1542.7753 | 780.4011 | 10 |
| 3 | 514.3240 | 496.3110 | 497.2971 | 257.6620 | K(+72.02) | 1359.6848 | 1341.6753 | 1342.6593 | 680.3431 | 9 |
| 4 | 643.3666 | 625.3561 | 626.3397 | 322.1833 | E | 1159.5701 | 1141.5610 | 1142.5386 | 580.2850 | 8 |
| 5 | 827.4878 | 809.4772 | 810.4608 | 414.2439 | K(+56.03) | 1030.5273 | 1012.5170 | 1013.5021 | 515.7638 | 7 |
| 6 | 955.5464 | 937.5358 | 938.5194 | 478.2732 | Q | 846.4048 | 828.3958 | 829.3815 | 423.7032 | 6 |
| 7 | 1084.5890 | 1066.5785 | 1067.5620 | 542.7945 | E | 718.3478 | 700.3372 | 701.3204 | 359.6782 | 5 |
| 8 | 1240.6901 | 1222.6796 | 1223.6631 | 620.8450 | R | 589.3044 | 571.2946 | 572.2798 | 295.1563 | 4 |
| 9 | 1369.7327 | 1351.7222 | 1352.7057 | 685.3663 | E | 433.2025 | 415.1941 | 416.1771 | 217.1021 | 3 |
| 10 | 1498.7753 | 1480.7648 | 1481.7483 | 749.8876 | E | 304.1616 | 286.1509 | 287.1345 | 152.5807 | 2 |
| 11 | | | | | R | 175.1190 | 157.1077 | 158.0925 | 88.0595 | 1 |

Figure S14_51

**MSMS assignment for K251ox in Luc7-like protein 3**

LUC7L3(247-254): LK*[O]K*EK*[O]QER; -10lgP 24.43; Scan 6492 (MC317)

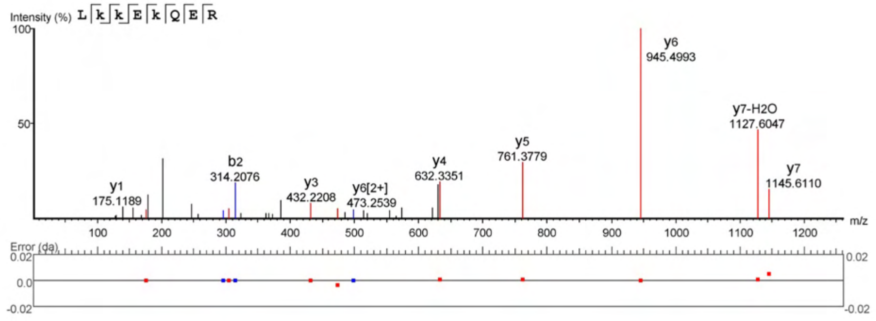


| # | b | b-H2O | b-NH3 | b (2+) | Seq | y | y-H2O | y-NH3 | y (2+) | # |
|---|---|---|---|---|---|---|---|---|---|---|
| 1 | 114.0919 | 96.0813 | 97.0649 | 57.5459 | L | | | | | 8 |
| 2 | 314.2076 | 296.1969 | 297.1810 | 157.6040 | K(+72.02) | 1145.6110 | 1127.6047 | 1128.5890 | 573.3080 | 7 |
| 3 | 498.3293 | 480.3186 | 481.3022 | 249.6646 | K(+56.03) | 945.4993 | 927.4894 | 928.4730 | 473.2539 | 6 |
| 4 | 627.3717 | 609.3611 | 610.3447 | 314.1859 | E | 761.3779 | 743.3682 | 744.3518 | 381.1894 | 5 |
| 5 | 827.4878 | 809.4772 | 810.4608 | 414.2439 | K(+72.02) | 632.3351 | 614.3256 | 615.3092 | 316.6681 | 4 |
| 6 | 955.5464 | 937.5358 | 938.5194 | 478.2732 | Q | 432.2208 | 414.2095 | 415.1931 | 216.6100 | 3 |
| 7 | 1084.5890 | 1066.5785 | 1067.5620 | 542.7945 | E | 304.1613 | 286.1509 | 287.1345 | 152.5807 | 2 |
| 8 | | | | | R | 175.1189 | 157.1084 | 158.0919 | 88.0595 | 1 |

**Figure S14_52**

**MSMS assignment for K359ox, K362ox in Luc7-like protein 3**

LUC7L3(357-364): DRK*[O]SYK*[O]HR; -10lgP 41.89; Scan 5575 (MC315)

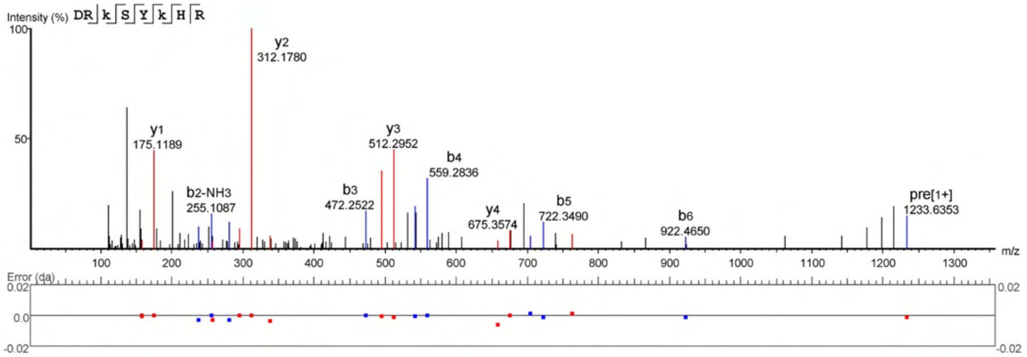


| # | b | b-H2O | b-NH3 | b (2+) | Seq | y | y-H2O | y-NH3 | y (2+) | # |
|---|---|---|---|---|---|---|---|---|---|---|
| 1 | 116.0348 | 98.0242 | 99.0078 | 58.5174 | D | | | | | 8 |
| 2 | 272.1359 | 254.1253 | 255.1087 | 136.5679 | R | 1118.6064 | 1100.5959 | 1101.5795 | 559.8032 | 7 |
| 3 | 472.2522 | 454.2414 | 455.2250 | 236.6295 | K(+72.02) | 962.5054 | 944.4948 | 945.4784 | 481.7527 | 6 |
| 4 | 559.2836 | 541.2740 | 542.2570 | 280.1452 | S | 762.3878 | 744.3787 | 745.3623 | 381.6946 | 5 |
| 5 | 722.3490 | 704.3351 | 705.3203 | 361.6736 | Y | 675.3574 | 657.3467 | 658.3365 | 338.1828 | 4 |
| 6 | 922.4650 | 904.4528 | 905.4364 | 461.7317 | K(+72.02) | 512.2952 | 494.2842 | 495.2670 | 256.6505 | 3 |
| 7 | 1059.5223 | 1041.5118 | 1042.4954 | 530.2612 | H | 312.1780 | 294.1670 | 295.1514 | 156.5889 | 2 |
| 8 | | | | | R | 175.1189 | 157.1084 | 158.0926 | 88.0595 | 1 |

Figure S14_53

**MSMS assignment of K375ox in Luc7-like protein 3**

LUC7L3(375-382): K*[O]SK*EK*EK*R; -10lgP 29.52; Scan 9691 (MC303)

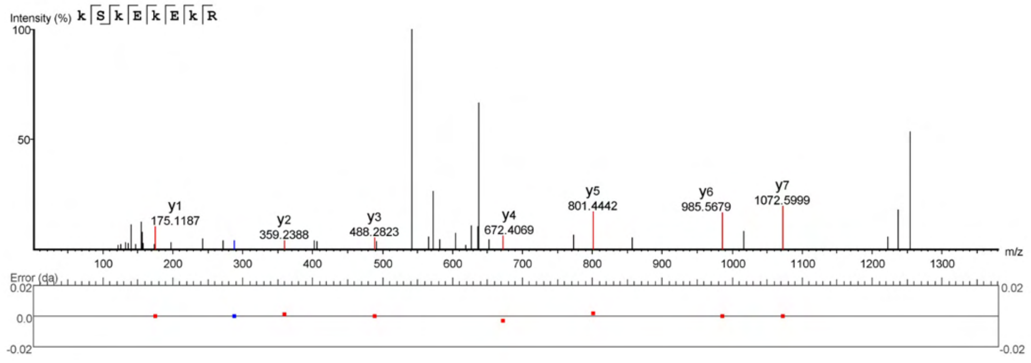


| # | b | b-H2O | b-NH3 | b (2+) | Seq | y | y-H2O | y-NH3 | y (2+) | # |
|---|---|---|---|---|---|---|---|---|---|---|
| 1 | 201.1239 | 183.1134 | 184.0969 | 101.0620 | K(+72.02) | | | | | 8 |
| 2 | 288.1557 | 270.1454 | 271.1290 | 144.5780 | S | 1072.5999 | 1054.5891 | 1055.5726 | 536.7998 | 7 |
| 3 | 472.2771 | 454.2665 | 455.2501 | 236.6385 | K(+56.03) | 985.5679 | 967.5571 | 968.5406 | 493.2838 | 6 |
| 4 | 601.3197 | 583.3091 | 584.2927 | 301.1599 | E | 801.4442 | 783.4359 | 784.4194 | 401.2232 | 5 |
| 5 | 785.4409 | 767.4303 | 768.4139 | 393.2204 | K(+56.03) | 672.4069 | 654.3933 | 655.3769 | 336.7019 | 4 |
| 6 | 914.4835 | 896.4729 | 897.4565 | 457.7417 | E | 488.2823 | 470.2721 | 471.2557 | 244.6413 | 3 |
| 7 | 1098.6046 | 1080.5941 | 1081.5776 | 549.8023 | K(+56.03) | 359.2388 | 341.2296 | 342.2131 | 180.1201 | 2 |
| 8 | | | | | R | 175.1187 | 157.1084 | 158.0919 | 88.0595 | 1 |

**Figure S14_54**

**MSMS assignment for K388ox in Luc7-like protein 3**

LUC7L3(383-396): GSDDK*K*[O]SSVK*SGSR; -10lgP 60.68; Scan 9889 (MC311)

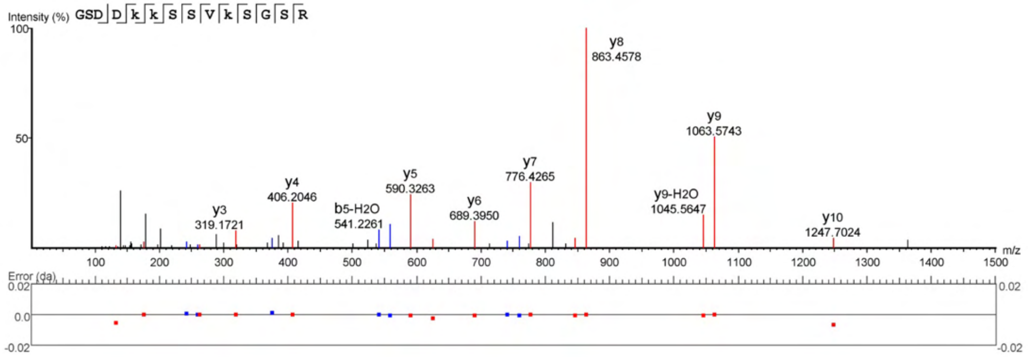


| # | b | b-H2O | b-NH3 | b (2+) | Seq | y | y-H2O | y-NH3 | y (2+) | # |
|---|---|---|---|---|---|---|---|---|---|---|
| 1 | 58.0293 | 40.0187 | 41.0023 | 29.5146 | G | | | | | 14 |
| 2 | 145.0613 | 127.0508 | 128.0343 | 73.0307 | S | 1564.7812 | 1546.7708 | 1547.7543 | 782.8906 | 13 |
| 3 | 260.0880 | 242.0770 | 243.0613 | 130.5441 | D | 1477.7493 | 1459.7388 | 1460.7223 | 739.3746 | 12 |
| 4 | 375.1136 | 357.1046 | 358.0882 | 188.0576 | D | 1362.7223 | 1344.7118 | 1345.6953 | 681.8611 | 11 |
| 5 | 559.2371 | 541.2261 | 542.2094 | 280.1182 | K(+56.03) | 1247.7024 | 1229.6848 | 1230.6683 | 624.3505 | 10 |
| 6 | 759.3535 | 741.3418 | 742.3254 | 380.1762 | K(+72.02) | 1063.5743 | 1045.5647 | 1046.5472 | 532.2871 | 9 |
| 7 | 846.3845 | 828.3739 | 829.3575 | 423.6922 | S | 863.4578 | 845.4485 | 846.4311 | 432.2290 | 8 |
| 8 | 933.4165 | 915.4059 | 916.3895 | 467.2083 | S | 776.4265 | 758.4155 | 759.3990 | 388.7130 | 7 |
| 9 | 1032.4849 | 1014.4743 | 1015.4579 | 516.7424 | V | 689.3950 | 671.3835 | 672.3671 | 345.1970 | 6 |
| 10 | 1216.6061 | 1198.5956 | 1199.5791 | 608.8030 | K(+56.03) | 590.3263 | 572.3151 | 573.2986 | 295.6628 | 5 |
| 11 | 1303.6381 | 1285.6276 | 1286.6111 | 652.3190 | S | 406.2046 | 388.1939 | 389.1775 | 203.6022 | 4 |
| 12 | 1360.6595 | 1342.6490 | 1343.6326 | 680.8298 | G | 319.1721 | 301.1619 | 302.1454 | 160.0862 | 3 |
| 13 | 1447.6917 | 1429.6812 | 1430.6647 | 724.3458 | S | 262.1514 | 244.1404 | 245.1240 | 131.5812 | 2 |
| 14 | | | | | R | 175.1189 | 157.1084 | 158.0919 | 88.0595 | 1 |

**Figure S14_55**

**MSMS assignment of K392ox in Luc7-like protein 3**

LUC7L3(383-396): GSDDK*K*SSVK*[O]SGSR; -10lgP 37.15; Scan 10322 (MC305)

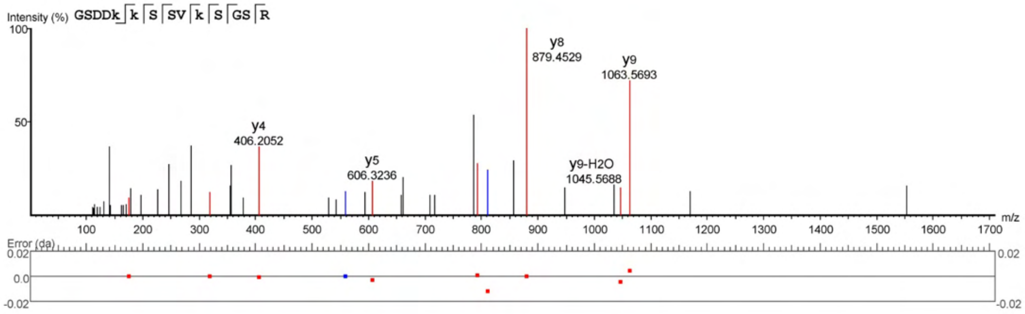


| # | b | b-H2O | b-NH3 | b (2+) | Seq | y | y-H2O | y-NH3 | y (2+) | # |
|---|---|---|---|---|---|---|---|---|---|---|
| 1 | 58.0293 | 40.0187 | 41.0023 | 29.5146 | G | | | | | 14 |
| 2 | 145.0613 | 127.0508 | 128.0343 | 73.0307 | S | 1564.7812 | 1546.7708 | 1547.7543 | 782.8906 | 13 |
| 3 | 260.0883 | 242.0777 | 243.0613 | 130.5441 | D | 1477.7493 | 1459.7388 | 1460.7223 | 739.3746 | 12 |
| 4 | 375.1152 | 357.1046 | 358.0882 | 188.0576 | D | 1362.7223 | 1344.7118 | 1345.6953 | 681.8611 | 11 |
| 5 | 559.2363 | 541.2258 | 542.2094 | 280.1182 | K(+56.03) | 1247.6953 | 1229.6848 | 1230.6683 | 624.3477 | 10 |
| 6 | 743.3575 | 725.3470 | 726.3306 | 372.1788 | K(+56.03) | 1063.5693 | 1045.5688 | 1046.5472 | 532.2871 | 9 |
| 7 | 830.3895 | 812.3790 | 813.3625 | 415.6948 | S | 879.4529 | 861.4424 | 862.4260 | 440.2265 | 8 |
| 8 | 917.4216 | 899.4110 | 900.3946 | 459.2108 | S | 792.4198 | 774.4104 | 775.3940 | 396.7105 | 7 |
| 9 | 1016.4900 | 998.4794 | 999.4630 | 508.7450 | V | 705.3889 | 687.3784 | 688.3619 | 353.1945 | 6 |
| 10 | 1216.6061 | 1198.5956 | 1199.5791 | 608.8030 | K(+72.02) | 606.3236 | 588.3100 | 589.2936 | 303.6603 | 5 |
| 11 | 1303.6381 | 1285.6276 | 1286.6111 | 652.3190 | S | 406.2052 | 388.1939 | 389.1775 | 203.6022 | 4 |
| 12 | 1360.6595 | 1342.6490 | 1343.6326 | 680.8298 | G | 319.1725 | 301.1619 | 302.1454 | 160.0862 | 3 |
| 13 | 1447.6917 | 1429.6812 | 1430.6647 | 724.3458 | S | 262.1510 | 244.1404 | 245.1240 | 131.5755 | 2 |
| 14 | | | | | R | 175.1190 | 157.1084 | 158.0919 | 88.0595 | 1 |

Figure S14_56

**MSMS assignment of K322ox in 39S ribosomal protein L39, mitochondrial**

MRPL39(322-330): K*[O]MVTEDQSK; -10lgP 42.04; Scan 6031 (MC314)

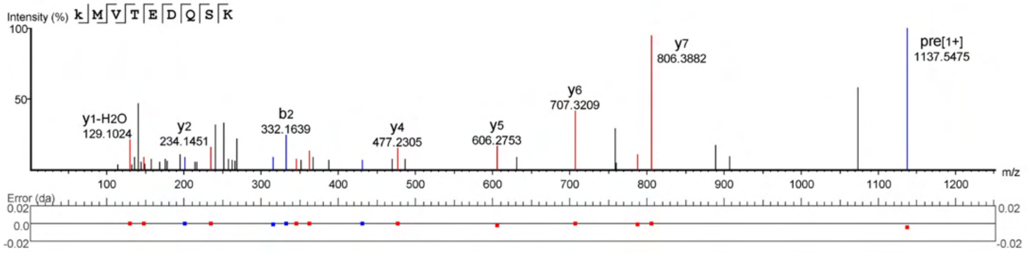


| # | b | b-H2O | b-NH3 | b (2+) | Seq | y | y-H2O | y-NH3 | y (2+) | # |
|---|---|---|---|---|---|---|---|---|---|---|
| 1 | 201.1239 | 183.1134 | 184.0969 | 101.0620 | K(+72.02) | | | | | 9 |
| 2 | 332.1639 | 314.1538 | 315.1392 | 166.5822 | M | 937.4295 | 919.4189 | 920.4025 | 469.2148 | 8 |
| 3 | 431.2319 | 413.2223 | 414.2058 | 216.1164 | V | 806.3882 | 788.3802 | 789.3621 | 403.6945 | 7 |
| 4 | 532.2805 | 514.2700 | 515.2535 | 266.6403 | T | 707.3209 | 689.3101 | 690.2936 | 354.1603 | 6 |
| 5 | 661.3231 | 643.3125 | 644.2961 | 331.1615 | E | 606.2753 | 588.2623 | 589.2459 | 303.6364 | 5 |
| 6 | 776.3500 | 758.3395 | 759.3231 | 388.6750 | D | 477.2305 | 459.2198 | 460.2033 | 239.1152 | 4 |
| 7 | 904.4086 | 886.3980 | 887.3816 | 452.7043 | Q | 362.2026 | 344.1928 | 345.1765 | 181.6017 | 3 |
| 8 | 991.4406 | 973.4301 | 974.4136 | 496.2203 | S | 234.1451 | 216.1343 | 217.1178 | 117.5724 | 2 |
| 9 | | | | | K | 147.1133 | 129.1024 | 130.0858 | 74.0564 | 1 |

Figure S14_57

**MSMS assignment of K198ox, K19ox, K200ox, K203ox in NF-kappa-B-activating protein**

NKAP(197-204): RK*[O]K*[O]K*[O]SSK*[O]R; -10lgP 21.51; Scan 4675 (MC309)

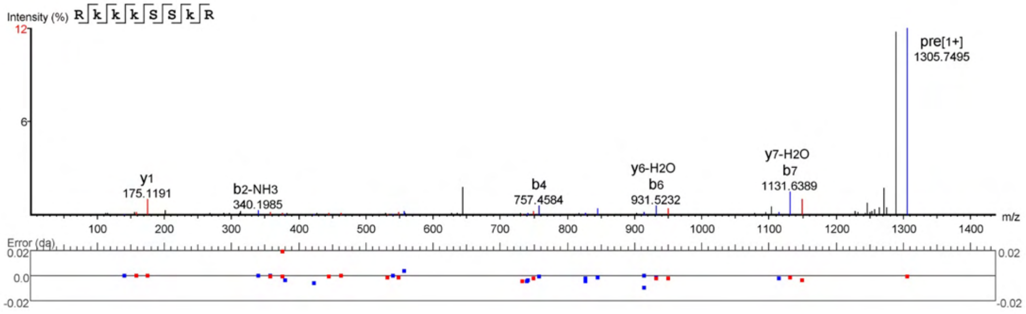


| # | b | b-H2O | b-NH3 | b (2+) | Seq | y | y-H2O | y-NH3 | y (2+) | # |
|---|---|---|---|---|---|---|---|---|---|---|
| 1 | 157.1089 | 139.0984 | 140.0815 | 79.0545 | R | | | | | 8 |
| 2 | 357.2254 | 339.2144 | 340.1985 | 179.1125 | K(+72.02) | 1149.6514 | 1131.6389 | 1132.6204 | 575.3237 | 7 |
| 3 | 557.3372 | 539.3305 | 540.3146 | 279.1705 | K(+72.02) | 949.5339 | 931.5232 | 932.5043 | 475.2656 | 6 |
| 4 | 757.4584 | 739.4514 | 740.4343 | 379.2327 | K(+72.02) | 749.4174 | 731.4046 | 732.3929 | 375.1883 | 5 |
| 5 | 844.4913 | 826.4812 | 827.4669 | 422.7505 | S | 549.3009 | 531.2885 | 532.2742 | 275.1495 | 4 |
| 6 | 931.5232 | 913.5112 | 914.5046 | 466.2606 | S | 462.2676 | 444.2565 | 445.2412 | 231.6335 | 3 |
| 7 | 1131.6389 | 1113.6268 | 1114.6130 | 566.3187 | K(+72.02) | 375.2363 | 357.2254 | 358.2095 | 188.1175 | 2 |
| 8 | | | | | R | 175.1191 | 157.1084 | 158.0924 | 88.0595 | 1 |

**Figure S14_58**

**MSMS assignment of K199ox in Histone-lysine N-methyltransferase NSD3**

NSD3(198-207): K*K*[O]SNK*HDSSR; -10lgP 33.37; Scan 5486 (MC361)

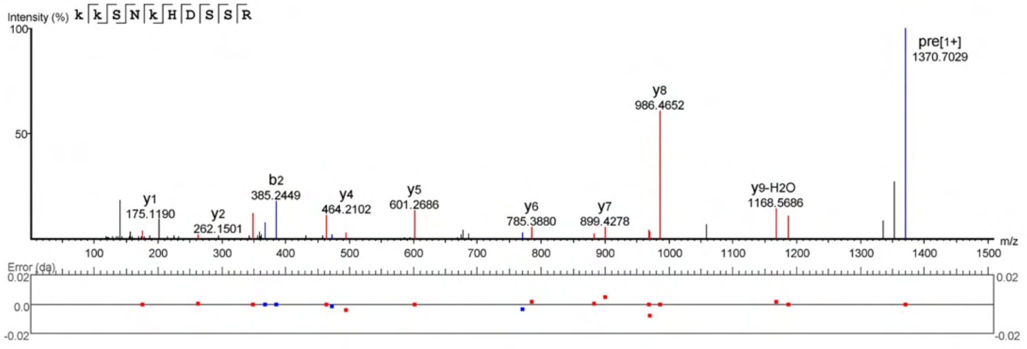


| # | b | b-H2O | b-NH3 | b (2+) | Seq | y | y-H2O | y-NH3 | y (2+) | # |
|---|---|---|---|---|---|---|---|---|---|---|
| 1 | 185.1290 | 167.1184 | 168.1020 | 93.0645 | K(+56.03) | | | | | 10 |
| 2 | 385.2449 | 367.2341 | 368.2181 | 193.1225 | K(+72.02) | 1186.5806 | 1168.5686 | 1169.5541 | 593.7905 | 9 |
| 3 | 472.2788 | 454.2665 | 455.2501 | 236.6385 | S | 986.4652 | 968.4546 | 969.4462 | 493.7368 | 8 |
| 4 | 586.3200 | 568.3094 | 569.2930 | 293.6600 | N | 899.4278 | 881.4224 | 882.4052 | 450.2165 | 7 |
| 5 | 770.4446 | 752.4307 | 753.4142 | 385.7206 | K(+56.03) | 785.3880 | 767.3795 | 768.3630 | 393.1950 | 6 |
| 6 | 907.5001 | 889.4896 | 890.4731 | 454.2501 | H | 601.2686 | 583.2583 | 584.2419 | 301.1344 | 5 |
| 7 | 1022.5270 | 1004.5165 | 1005.5001 | 511.7635 | D | 464.2102 | 446.1994 | 447.1830 | 232.6050 | 4 |
| 8 | 1109.5591 | 1091.5486 | 1092.5321 | 555.2795 | S | 349.1830 | 331.1724 | 332.1560 | 175.0915 | 3 |
| 9 | 1196.5911 | 1178.5806 | 1179.5641 | 598.7955 | S | 262.1501 | 244.1404 | 245.1240 | 131.5755 | 2 |
| 10 | | | | | R | 175.1190 | 157.1084 | 158.0919 | 88.0595 | 1 |

**Figure S14_59**

**MSMS assignment of K173 in PHD finger protein 6**

PHF6(169-180): K*TNFK*[O]GLSEDTR; -10lgP 41.15; Scan 12215 (MC319)

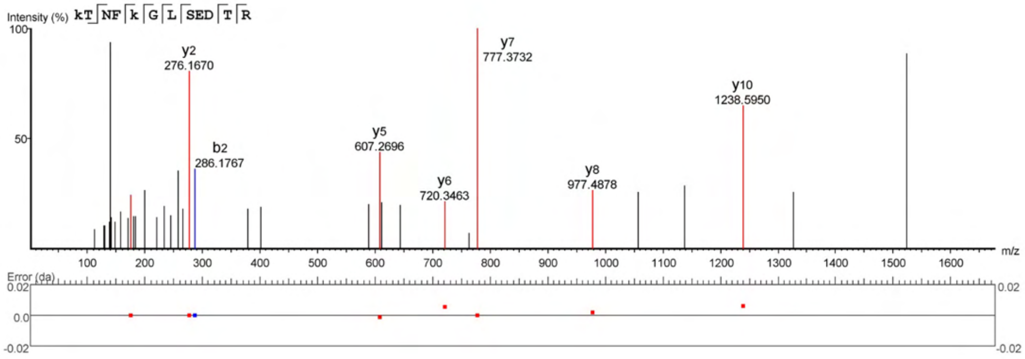


| # | b | b-H2O | b-NH3 | b (2+) | Seq | y | y-H2O | y-NH3 | y (2+) | # |
|---|---|---|---|---|---|---|---|---|---|---|
| 1 | 185.1290 | 167.1184 | 168.1020 | 93.0645 | K(+56.03) | | | | | 12 |
| 2 | 286.1767 | 268.1661 | 269.1497 | 143.5883 | T | 1339.6488 | 1321.6383 | 1322.6218 | 670.3244 | 11 |
| 3 | 400.2196 | 382.2090 | 383.1926 | 200.6098 | N | 1238.5950 | 1220.5906 | 1221.5741 | 619.8005 | 10 |
| 4 | 547.2880 | 529.2775 | 530.2610 | 274.1440 | F | 1124.5582 | 1106.5477 | 1107.5312 | 562.7791 | 9 |
| 5 | 747.4041 | 729.3936 | 730.3771 | 374.2021 | K(+72.02) | 977.4878 | 959.4792 | 960.4628 | 489.2449 | 8 |
| 6 | 804.4255 | 786.4150 | 787.3986 | 402.7128 | G | 777.3732 | 759.3631 | 760.3467 | 389.1868 | 7 |
| 7 | 917.5096 | 899.4991 | 900.4827 | 459.2548 | L | 720.3463 | 702.3417 | 703.3253 | 360.6761 | 6 |
| 8 | 1004.5416 | 986.5311 | 987.5146 | 502.7708 | S | 607.2696 | 589.2576 | 590.2412 | 304.1341 | 5 |
| 9 | 1133.5842 | 1115.5737 | 1116.5573 | 567.2921 | E | 520.2361 | 502.2256 | 503.2092 | 260.6181 | 4 |
| 10 | 1248.6112 | 1230.6007 | 1231.5842 | 624.8056 | D | 391.1935 | 373.1830 | 374.1666 | 196.0968 | 3 |
| 11 | 1349.6588 | 1331.6483 | 1332.6318 | 675.3294 | T | 276.1670 | 258.1561 | 259.1396 | 138.5833 | 2 |
| 12 | | | | | R | 175.1189 | 157.1084 | 158.0919 | 88.0595 | 1 |

**Figure S14_60**

**MSMS assignment of K1068ox, K1069ox, K1071ox, K1073ox in PHD and RING finger domain-containing protein 1**

PHRF1(1068-1075): K*[O]K*[O]AK*[O]DK*[O]SR; -10lgP 18.49; Scan 54671 (MC301)

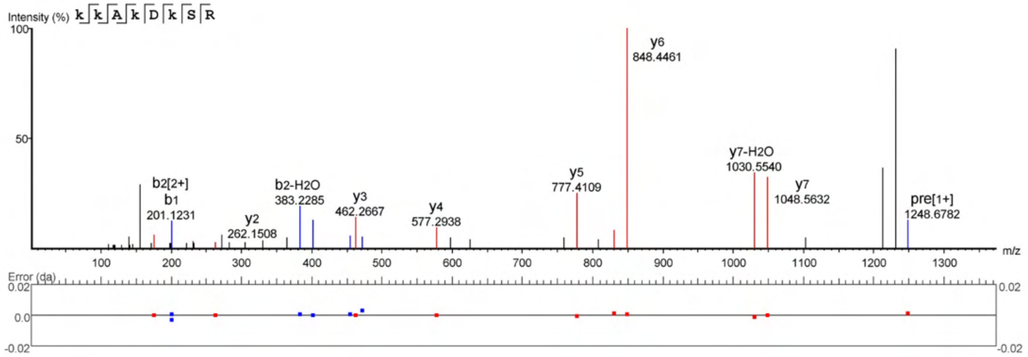


| # | b | b-H2O | b-NH3 | b (2+) | Seq | y | y-H2O | y-NH3 | y (2+) | # |
|---|---|---|---|---|---|---|---|---|---|---|
| 1 | 201.1231 | 183.1134 | 184.0969 | 101.0620 | K(+72.02) | | | | | 8 |
| 2 | 401.2394 | 383.2285 | 384.2130 | 201.1231 | K(+72.02) | 1048.5632 | 1030.5540 | 1031.5363 | 524.7816 | 7 |
| 3 | 472.2738 | 454.2659 | 455.2501 | 236.6385 | A | 848.4461 | 830.4349 | 831.4202 | 424.7236 | 6 |
| 4 | 672.3932 | 654.3826 | 655.3662 | 336.6966 | K(+72.02) | 777.4109 | 759.3995 | 760.3831 | 389.2050 | 5 |
| 5 | 787.4201 | 769.4095 | 770.3931 | 394.2101 | D | 577.2938 | 559.2834 | 560.2670 | 289.1470 | 4 |
| 6 | 987.5362 | 969.5256 | 970.5092 | 494.2681 | K(+72.02) | 462.2667 | 444.2565 | 445.2401 | 231.6335 | 3 |
| 7 | 1074.5682 | 1056.5577 | 1057.5413 | 537.7841 | S | 262.1508 | 244.1404 | 245.1240 | 131.5755 | 2 |
| 8 | | | | | R | 175.1187 | 157.1084 | 158.0919 | 88.0595 | 1 |

**Figure S14_61**

**MSMS assignment for K458ox in Peptidyl-prolyl cis-trans isomerase-like 4**

PPIL4(450-468): DGHYSNSHK*[O]SK*YQTDLYER; -10lgP 74.89; Scan 10653 (MC302)

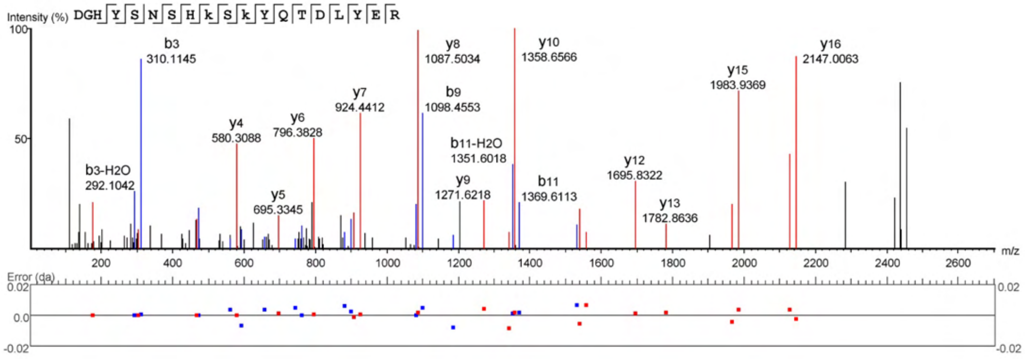


| # | b | b-H2O | b-NH3 | b (2+) | Seq | y | y-H2O | y-NH3 | y (2+) | # |
|---|---|---|---|---|---|---|---|---|---|---|
| 1 | 116.0348 | 98.0242 | 99.0078 | 58.5174 | D | | | | | 19 |
| 2 | 173.0562 | 155.0457 | 156.0292 | 87.0281 | G | 2341.0842 | 2323.0737 | 2324.0571 | 1171.0421 | 18 |
| 3 | 310.1145 | 292.1042 | 293.0881 | 155.5576 | H | 2284.0627 | 2266.0522 | 2267.0356 | 1142.5314 | 17 |
| 4 | 473.1781 | 455.1679 | 456.1515 | 237.0892 | Y | 2147.0063 | 2128.9897 | 2129.9768 | 1074.0020 | 16 |
| 5 | 560.2067 | 542.1999 | 543.1835 | 280.6052 | S | 1983.9369 | 1965.9302 | 1966.9185 | 992.4703 | 15 |
| 6 | 674.2534 | 656.2391 | 657.2264 | 337.6267 | N | 1896.9086 | 1878.8981 | 1879.8816 | 948.9543 | 14 |
| 7 | 761.2855 | 743.2695 | 744.2585 | 381.1427 | S | 1782.8636 | 1764.8551 | 1765.8386 | 891.9328 | 13 |
| 8 | 898.3419 | 880.3271 | 881.3174 | 449.6722 | H | 1695.8322 | 1677.8231 | 1678.8066 | 848.4168 | 12 |
| 9 | 1098.4553 | 1080.4500 | 1081.4335 | 549.7302 | K(+72.02) | 1558.7675 | 1540.7700 | 1541.7477 | 779.8873 | 11 |
| 10 | 1185.5009 | 1167.4819 | 1168.4655 | 593.2534 | S | 1358.6566 | 1340.6481 | 1341.6405 | 679.8293 | 10 |
| 11 | 1369.6113 | 1351.6018 | 1352.5867 | 685.3068 | K(+56.03) | 1271.6218 | 1253.6161 | 1254.5996 | 636.3133 | 9 |
| 12 | 1532.6698 | 1514.6665 | 1515.6500 | 766.8385 | Y | 1087.5034 | 1069.4949 | 1070.4784 | 544.2527 | 8 |
| 13 | 1660.7356 | 1642.7251 | 1643.7086 | 830.8678 | Q | 924.4412 | 906.4315 | 907.4165 | 462.7210 | 7 |
| 14 | 1761.7832 | 1743.7727 | 1744.7562 | 881.3916 | T | 796.3828 | 778.3730 | 779.3566 | 398.6918 | 6 |
| 15 | 1876.8102 | 1858.7997 | 1859.7832 | 938.9051 | D | 695.3345 | 677.3253 | 678.3088 | 348.1679 | 5 |
| 16 | 1989.8943 | 1971.8838 | 1972.8673 | 995.4471 | L | 580.3088 | 562.2983 | 563.2819 | 290.6544 | 4 |
| 17 | 2152.9575 | 2134.9470 | 2135.9304 | 1076.9788 | Y | 467.2250 | 449.2143 | 450.1979 | 234.1124 | 3 |
| 18 | 2282.0002 | 2263.9897 | 2264.9731 | 1141.5001 | E | 304.1611 | 286.1509 | 287.1345 | 152.5807 | 2 |
| 19 | | | | | R | 175.1189 | 157.1084 | 158.0919 | 88.0595 | 1 |

**Figure S14_62**

**MSMS assignment of K103ox in RNA-binding protein 39**

RBM39(97-109): SPYSGPK*[O]FNSAIR; -10lgP 54.47; Scan 14607 (MC315)

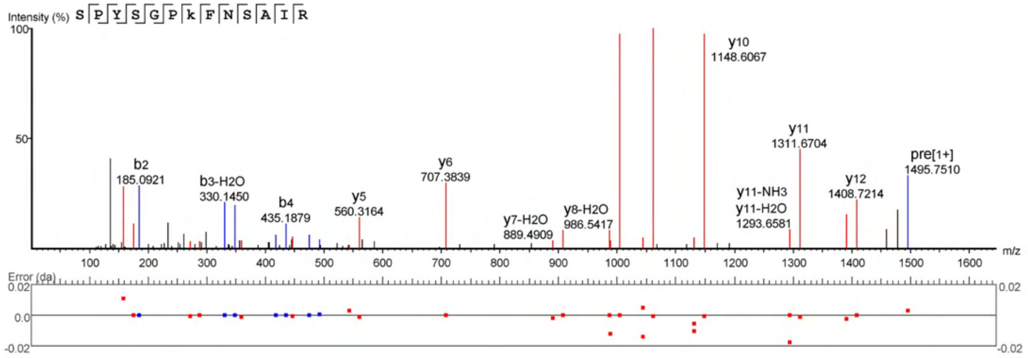


| # | b | b-H2O | b-NH3 | b (2+) | Seq | y | y-H2O | y-NH3 | y (2+) | # |
|---|---|---|---|---|---|---|---|---|---|---|
| 1 | 88.0399 | 70.0293 | 71.0129 | 44.5199 | S | | | | | 13 |
| 2 | 185.0921 | 167.0820 | 168.0656 | 93.0463 | P | 1408.7214 | 1390.7139 | 1391.6949 | 704.8610 | 12 |
| 3 | 348.1558 | 330.1450 | 331.1289 | 174.5780 | Y | 1311.6704 | 1293.6581 | 1294.6599 | 656.3345 | 11 |
| 4 | 435.1879 | 417.1774 | 418.1610 | 218.0940 | S | 1148.6067 | 1130.6011 | 1131.5895 | 574.8029 | 10 |
| 5 | 492.2085 | 474.1989 | 475.1824 | 246.6047 | G | 1061.5746 | 1043.5580 | 1044.5610 | 531.2869 | 9 |
| 6 | 589.2621 | 571.2516 | 572.2352 | 295.1311 | P | 1004.5529 | 986.5417 | 987.5375 | 502.7762 | 8 |
| 7 | 789.3782 | 771.3677 | 772.3513 | 395.1891 | K(+72.02) | 907.4990 | 889.4909 | 890.4726 | 454.2498 | 7 |
| 8 | 936.4467 | 918.4361 | 919.4197 | 468.7233 | F | 707.3839 | 689.3729 | 690.3565 | 354.1917 | 6 |
| 9 | 1050.4896 | 1032.4791 | 1033.4626 | 525.7448 | N | 560.3164 | 542.3045 | 543.2850 | 280.6575 | 5 |
| 10 | 1137.5216 | 1119.5111 | 1120.4946 | 569.2608 | S | 446.2728 | 428.2616 | 429.2451 | 223.6361 | 4 |
| 11 | 1208.5587 | 1190.5482 | 1191.5317 | 604.7794 | A | 359.2416 | 341.2296 | 342.2131 | 180.1201 | 3 |
| 12 | 1321.6428 | 1303.6323 | 1304.6158 | 661.3214 | I | 288.2031 | 270.1924 | 271.1767 | 144.6015 | 2 |
| 13 | | | | | R | 175.1189 | 157.0971 | 158.0919 | 88.0595 | 1 |

**Figure S14_63**

**MSMS assignment of K119ox in RNA-binding protein 39**

RBM39(110-122): GK*IGLPHSIK*[O]LSR; -10lgP 54.51; Scan 16858 (MC315)

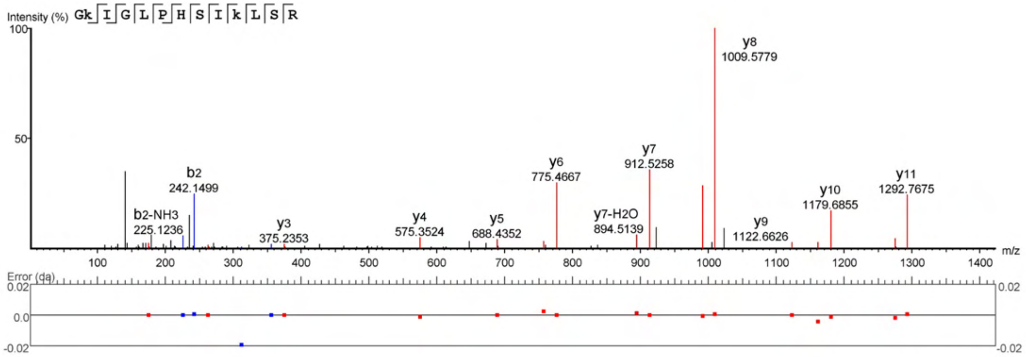


| # | b | b-H2O | b-NH3 | b (2+) | Seq | y | y-H2O | y-NH3 | y (2+) | # |
|---|---|---|---|---|---|---|---|---|---|---|
| 1 | 58.0293 | 40.0187 | 41.0023 | 29.5146 | G | | | | | 13 |
| 2 | 242.1499 | 224.1399 | 225.1236 | 121.5752 | K(+56.03) | 1476.8896 | 1458.8792 | 1459.8627 | 738.9448 | 12 |
| 3 | 355.2340 | 337.2239 | 338.2075 | 178.1172 | I | 1292.7675 | 1274.7598 | 1275.7415 | 646.8842 | 11 |
| 4 | 412.2560 | 394.2454 | 395.2290 | 206.6280 | G | 1179.6855 | 1161.6782 | 1162.6573 | 590.3422 | 10 |
| 5 | 525.3400 | 507.3295 | 508.3130 | 263.1700 | L | 1122.6626 | 1104.6525 | 1105.6360 | 561.8315 | 9 |
| 6 | 622.3928 | 604.3822 | 605.3658 | 311.7162 | P | 1009.5779 | 991.5690 | 992.5519 | 505.2894 | 8 |
| 7 | 759.4517 | 741.4412 | 742.4247 | 380.2259 | H | 912.5258 | 894.5139 | 895.4991 | 456.7631 | 7 |
| 8 | 846.4837 | 828.4731 | 829.4567 | 423.7419 | S | 775.4667 | 757.4537 | 758.4402 | 388.2336 | 6 |
| 9 | 959.5678 | 941.5573 | 942.5408 | 480.2839 | I | 688.4352 | 670.4246 | 671.4082 | 344.7176 | 5 |
| 10 | 1159.6838 | 1141.6733 | 1142.6569 | 580.3419 | K(+72.02) | 575.3524 | 557.3405 | 558.3241 | 288.1755 | 4 |
| 11 | 1272.7679 | 1254.7574 | 1255.7410 | 636.8840 | L | 375.2353 | 357.2245 | 358.2080 | 188.1175 | 3 |
| 12 | 1359.7999 | 1341.7894 | 1342.7729 | 680.4000 | S | 262.1513 | 244.1404 | 245.1240 | 131.5755 | 2 |
| 13 | | | | | R | 175.1190 | 157.1084 | 158.0919 | 88.0595 | 1 |

**Figure S14_64**

## MSMS assignment of K38ox in Replication factor C subunit 1

RFC1(36-49): AK*K*[O]GIK*EIK*VNSSR; -10lgP 40.41; Scan 42314 (MC358)

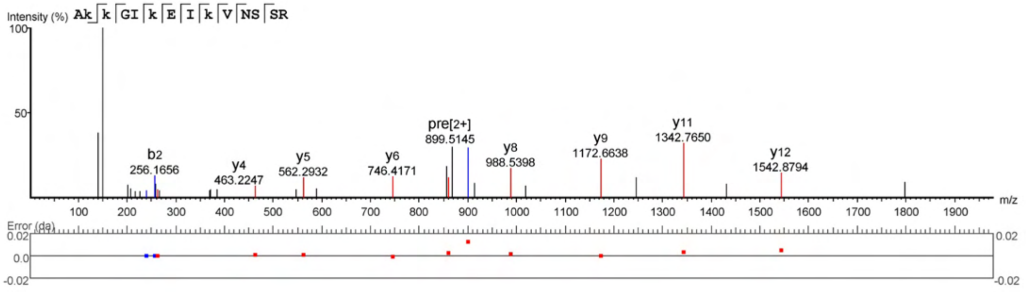


| # | b | b-H2O | b-NH3 | b (2+) | Seq | y | y-H2O | y-NH3 | y (2+) | # |
|---|---|---|---|---|---|---|---|---|---|---|
| 1 | 72.0449 | 54.0344 | 55.0179 | 36.5225 | A | | | | | 14 |
| 2 | 256.1656 | 238.1555 | 239.1399 | 128.5831 | K(+56.03) | 1727.0061 | 1708.9956 | 1709.9791 | 864.0031 | 13 |
| 3 | 456.2822 | 438.2716 | 439.2552 | 228.6411 | K(+72.02) | 1542.8794 | 1524.8744 | 1525.8579 | 771.9424 | 12 |
| 4 | 513.3036 | 495.2931 | 496.2767 | 257.1518 | G | 1342.7650 | 1324.7583 | 1325.7418 | 671.8844 | 11 |
| 5 | 626.3877 | 608.3771 | 609.3607 | 313.6938 | I | 1285.7473 | 1267.7368 | 1268.7203 | 643.3737 | 10 |
| 6 | 810.5089 | 792.4983 | 793.4819 | 405.7544 | K(+56.03) | 1172.6638 | 1154.6528 | 1155.6364 | 586.8317 | 9 |
| 7 | 939.5515 | 921.5409 | 922.5245 | 470.2757 | E | 988.5398 | 970.5316 | 971.5151 | 494.7711 | 8 |
| 8 | 1052.6355 | 1034.6250 | 1035.6085 | 526.8177 | I | 859.4969 | 841.4890 | 842.4726 | 430.2498 | 7 |
| 9 | 1236.7567 | 1218.7462 | 1219.7297 | 618.8784 | K(+56.03) | 746.4171 | 728.4050 | 729.3885 | 373.7078 | 6 |
| 10 | 1335.8251 | 1317.8146 | 1318.7981 | 668.4125 | V | 562.2932 | 544.2838 | 545.2673 | 281.6472 | 5 |
| 11 | 1449.8680 | 1431.8575 | 1432.8411 | 725.4340 | N | 463.2247 | 445.2154 | 446.1989 | 232.1130 | 4 |
| 12 | 1536.9000 | 1518.8895 | 1519.8730 | 768.9500 | S | 349.1830 | 331.1724 | 332.1560 | 175.0915 | 3 |
| 13 | 1623.9321 | 1605.9216 | 1606.9052 | 812.4661 | S | 262.1507 | 244.1404 | 245.1240 | 131.5755 | 2 |
| 14 | | | | | R | 175.1189 | 157.1084 | 158.0919 | 88.0595 | 1 |

**Figure S14_65**

**MSMS assignment of K535ox, K539ox in Serine/threonine-protein kinase RIO1**

RIOK1(535-543): TK*[O]K*MVK*[O]EAQR; -10lgP 28.22; Scan 9331 (MC316)

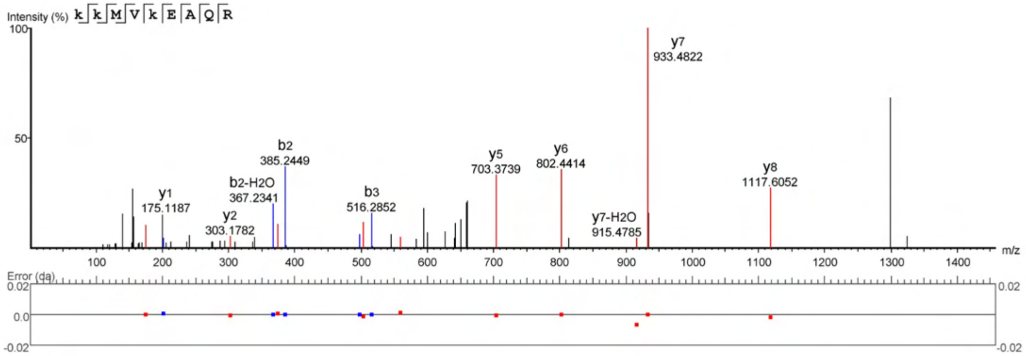


| # | b | b-H2O | b-NH3 | b (2+) | Seq | y | y-H2O | y-NH3 | y (2+) | # |
|---|---|---|---|---|---|---|---|---|---|---|
| 1 | 201.1231 | 183.1134 | 184.0969 | 101.0620 | K(+72.02) | | | | | 9 |
| 2 | 385.2449 | 367.2341 | 368.2181 | 193.1225 | K(+56.03) | 1117.6052 | 1099.5929 | 1100.5764 | 559.3003 | 8 |
| 3 | 516.2852 | 498.2745 | 499.2586 | 258.6428 | M | 933.4822 | 915.4785 | 916.4552 | 467.2411 | 7 |
| 4 | 615.3540 | 597.3434 | 598.3270 | 308.1770 | V | 802.4414 | 784.4312 | 785.4147 | 401.7209 | 6 |
| 5 | 815.4701 | 797.4595 | 798.4431 | 408.2350 | K(+72.02) | 703.3739 | 685.3627 | 686.3463 | 352.1866 | 5 |
| 6 | 944.5126 | 926.5021 | 927.4857 | 472.7563 | E | 503.2585 | 485.2466 | 486.2302 | 252.1286 | 4 |
| 7 | 1015.5497 | 997.5392 | 998.5228 | 508.2749 | A | 374.2140 | 356.2040 | 357.1876 | 187.6073 | 3 |
| 8 | 1143.6084 | 1125.5979 | 1126.5814 | 572.3042 | Q | 303.1782 | 285.1669 | 286.1505 | 152.0887 | 2 |
| 9 | | | | | R | 175.1187 | 157.1084 | 158.0919 | 88.0595 | 1 |

Figure S14_66

**MSMS assignment of K552ox, K555ox in Serine/threonine-protein kinase RIO1**

RIOK1(547-557): K*NK*IPK*[O]HVK*[O]K*R; -10lgP 40.00; Scan 13202 (MC315)

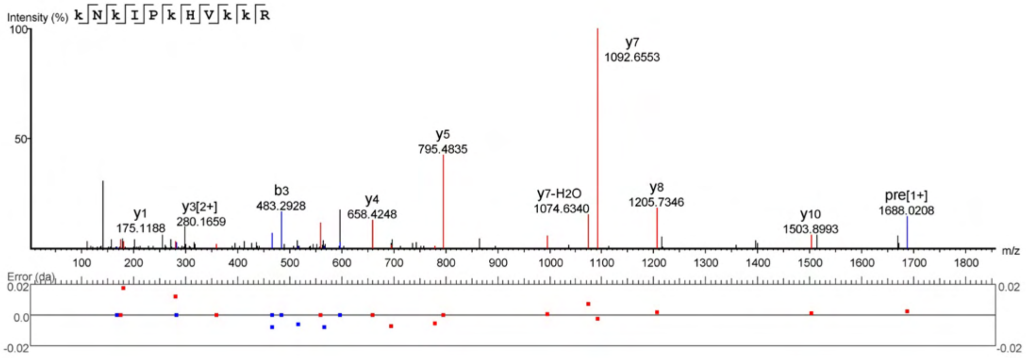


| # | b | b-H2O | b-NH3 | b (2+) | Seq | y | y-H2O | y-NH3 | y (2+) | # |
|---|---|---|---|---|---|---|---|---|---|---|
| 1 | 185.1290 | 167.1184 | 168.1022 | 93.0645 | K(+56.03) | | | | | 11 |
| 2 | 299.1719 | 281.1613 | 282.1454 | 150.0860 | N | 1503.8993 | 1485.8900 | 1486.8735 | 752.4503 | 10 |
| 3 | 483.2928 | 465.2820 | 466.2745 | 242.1465 | K(+56.03) | 1389.8575 | 1371.8470 | 1372.8306 | 695.4366 | 9 |
| 4 | 596.3766 | 578.3666 | 579.3502 | 298.6886 | I | 1205.7346 | 1187.7260 | 1188.7095 | 603.3682 | 8 |
| 5 | 693.4299 | 675.4194 | 676.4030 | 347.2150 | P | 1092.6553 | 1074.6340 | 1075.6254 | 546.8262 | 7 |
| 6 | 893.5460 | 875.5355 | 876.5190 | 447.2730 | K(+72.02) | 995.5986 | 977.5891 | 978.5726 | 498.2998 | 6 |
| 7 | 1030.6049 | 1012.5943 | 1013.5779 | 515.8090 | H | 795.4835 | 777.4730 | 778.4622 | 398.2418 | 5 |
| 8 | 1129.6733 | 1111.6628 | 1112.6464 | 565.3450 | V | 658.4248 | 640.4141 | 641.3976 | 329.7123 | 4 |
| 9 | 1329.7894 | 1311.7789 | 1312.7625 | 665.3947 | K(+72.02) | 559.3567 | 541.3456 | 542.3292 | 280.1659 | 3 |
| 10 | 1513.9105 | 1495.9000 | 1496.8835 | 757.4553 | K(+56.03) | 359.2401 | 341.2296 | 342.2131 | 180.1019 | 2 |
| 11 | | | | | R | 175.1188 | 157.1084 | 158.0919 | 88.0595 | 1 |

**Figure S14_67**

**MSMS assignment of K45ox in 60S ribosomal protein L35a**

RPL35A(37-46): DETEFYLGK*[O]R; -10lgP 30.99; Scan 14742 (MC326)

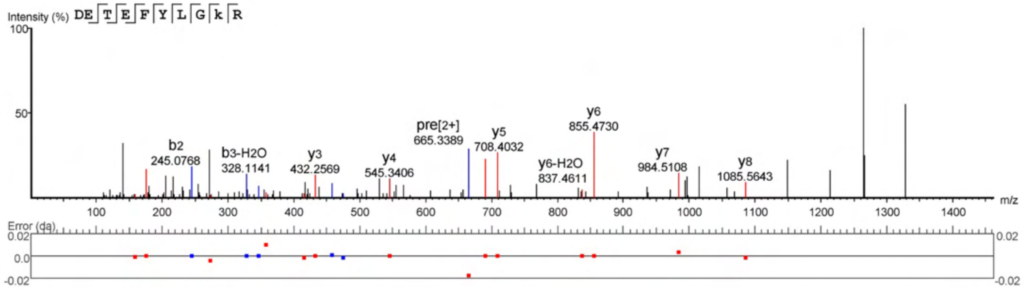


| # | b | b-H2O | b-NH3 | b (2+) | Seq | y | y-H2O | y-NH3 | y (2+) | # |
|---|---|---|---|---|---|---|---|---|---|---|
| 1 | 116.0348 | 98.0242 | 99.0078 | 58.5174 | D | | | | | 10 |
| 2 | 245.0768 | 227.0668 | 228.0503 | 123.0387 | E | 1214.6051 | 1196.5946 | 1197.5781 | 607.8026 | 9 |
| 3 | 346.1246 | 328.1141 | 329.0981 | 173.5625 | T | 1085.5643 | 1067.5520 | 1068.5355 | 543.2812 | 8 |
| 4 | 475.1693 | 457.1561 | 458.1406 | 238.0838 | E | 984.5108 | 966.5043 | 967.4879 | 492.7574 | 7 |
| 5 | 622.2360 | 604.2255 | 605.2090 | 311.6180 | F | 855.4730 | 837.4611 | 838.4453 | 428.2361 | 6 |
| 6 | 785.2993 | 767.2888 | 768.2723 | 393.1497 | Y | 708.4032 | 690.3928 | 691.3769 | 354.7019 | 5 |
| 7 | 898.3834 | 880.3729 | 881.3564 | 449.6917 | L | 545.3406 | 527.3300 | 528.3135 | 273.1746 | 4 |
| 8 | 955.4048 | 937.3943 | 938.3779 | 478.2024 | G | 432.2569 | 414.2459 | 415.2319 | 216.6282 | 3 |
| 9 | 1155.5210 | 1137.5105 | 1138.4940 | 578.2605 | K(+72.02) | 375.2350 | 357.2141 | 358.2080 | 188.1175 | 2 |
| 10 | | | | | R | 175.1188 | 157.1084 | 158.0928 | 88.0595 | 1 |

**Figure S14_68**

**MSMS assignment of K113ox in 40S ribosomal protein S7**

RPS7(112-118): NK*[O]QK*RPR; -10lgP 23.54; Scan3701 (MC330)

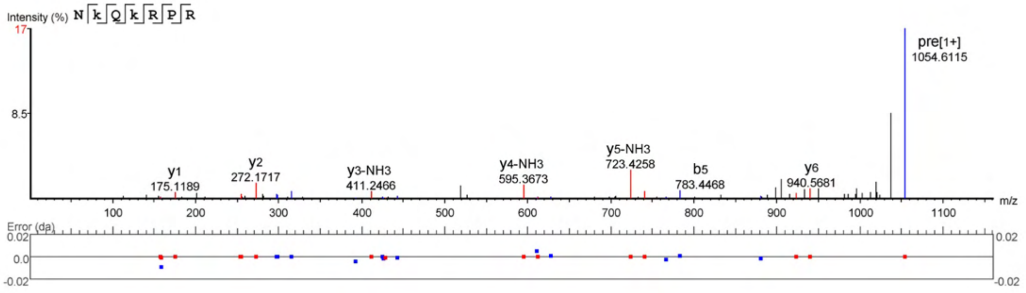


| # | b | b-H2O | b-NH3 | b (2+) | Seq | y | y-H2O | y-NH3 | y (2+) | # |
|---|---|---|---|---|---|---|---|---|---|---|
| 1 | 115.0508 | 97.0402 | 98.0238 | 58.0254 | N | | | | | 7 |
| 2 | 315.1664 | 297.1559 | 298.1396 | 158.0928 | K(+72.02) | 940.5681 | 922.5580 | 923.5416 | 470.7843 | 6 |
| 3 | 443.2263 | 425.2144 | 426.2002 | 222.1127 | Q | 740.4531 | 722.4420 | 723.4258 | 370.7263 | 5 |
| 4 | 627.3455 | 609.3360 | 610.3145 | 314.1733 | K(+56.03) | 612.3944 | 594.3834 | 595.3673 | 306.6970 | 4 |
| 5 | 783.4468 | 765.4371 | 766.4236 | 392.2286 | R | 428.2741 | 410.2622 | 411.2466 | 214.6364 | 3 |
| 6 | 880.5022 | 862.4899 | 863.4734 | 440.7502 | P | 272.1717 | 254.1614 | 255.1450 | 136.5858 | 2 |
| 7 | | | | | R | 175.1189 | 157.1081 | 158.0928 | 88.0595 | 1 |

Figure S14_69

**MSMS assignment of K14ox in 40S ribosomal protein S25**
RPS25(9-17): K*K*DAGK*[O]SAK; -10lgP 29.45; Scan 4813 (MC328)

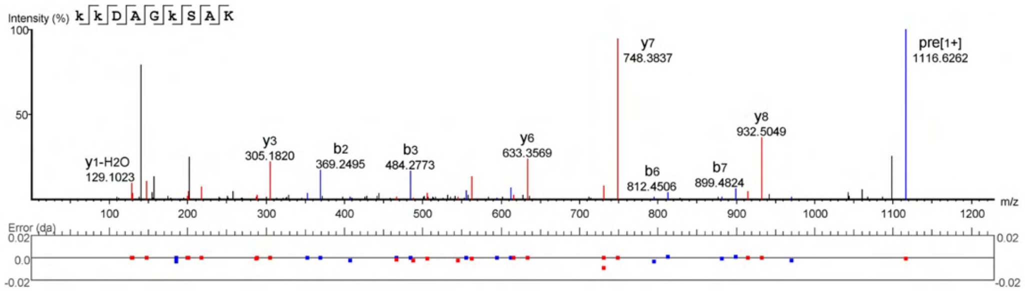


| # | b | b-H2O | b-NH3 | b (2+) | Seq | y | y-H2O | y-NH3 | y (2+) | # |
|---|---|---|---|---|---|---|---|---|---|---|
| 1 | 185.1289 | 167.1184 | 168.1020 | 93.0645 | K(+56.03) | | | | | 9 |
| 2 | 369.2495 | 351.2396 | 352.2238 | 185.1289 | K(+56.03) | 932.5049 | 914.4938 | 915.4777 | 466.7547 | 8 |
| 3 | 484.2773 | 466.2670 | 467.2501 | 242.6385 | D | 748.3837 | 730.3732 | 731.3663 | 374.6918 | 7 |
| 4 | 555.3141 | 537.3036 | 538.2872 | 278.1571 | A | 633.3569 | 615.3456 | 616.3296 | 317.1783 | 6 |
| 5 | 612.3352 | 594.3247 | 595.3087 | 306.6678 | G | 562.3205 | 544.3114 | 545.2925 | 281.6597 | 5 |
| 6 | 812.4506 | 794.4447 | 795.4248 | 406.7292 | K(+72.02) | 505.2992 | 487.2903 | 488.2710 | 253.1490 | 4 |
| 7 | 899.4824 | 881.4745 | 882.4568 | 450.2419 | S | 305.1820 | 287.1724 | 288.1551 | 153.0910 | 3 |
| 8 | 970.5239 | 952.5103 | 953.4939 | 485.7604 | A | 218.1499 | 200.1396 | 201.1234 | 109.5750 | 2 |
| 9 | | | | | K | 147.1127 | 129.1023 | 130.0861 | 74.0564 | 1 |

Figure S14_70

**MSMS assignment of K320ox in Splicing factor 3B subunit 2**

SF3B2(302-325): SSLGQSASETEEDTVSVSK*[O]K*EK*NR; -10lgP 52.86; Scan 16763 (MC304)

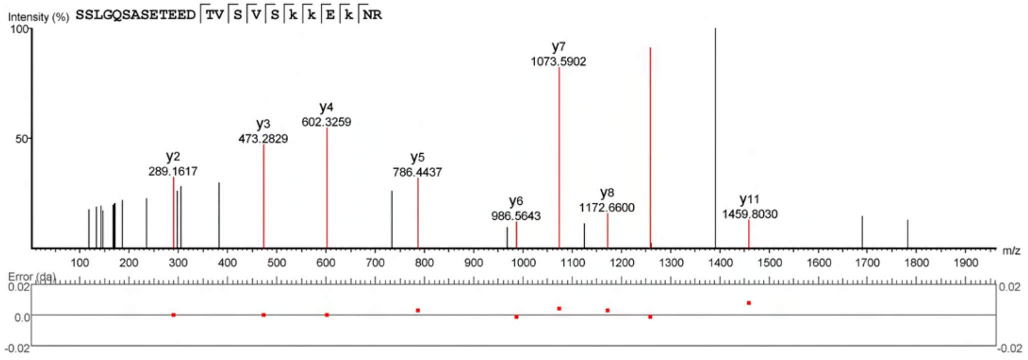


| # | b | b-H2O | b-NH3 | b (2+) | Seq | y | y-H2O | y-NH3 | y (2+) | # |
|---|---|---|---|---|---|---|---|---|---|---|
| 1 | 88.0399 | 70.0293 | 71.0129 | 44.5199 | S | | | | | 24 |
| 2 | 175.0719 | 157.0613 | 158.0449 | 88.0359 | S | 2693.3110 | 2675.3005 | 2676.2839 | 1347.1555 | 23 |
| 3 | 288.1559 | 270.1454 | 271.1290 | 144.5780 | L | 2606.2791 | 2588.2686 | 2589.2520 | 1303.6395 | 22 |
| 4 | 345.1774 | 327.1668 | 328.1504 | 173.0887 | G | 2493.1951 | 2475.1846 | 2476.1680 | 1247.0975 | 21 |
| 5 | 473.2360 | 455.2254 | 456.2090 | 237.1180 | Q | 2436.1736 | 2418.1631 | 2419.1465 | 1218.5868 | 20 |
| 6 | 560.2680 | 542.2574 | 543.2410 | 280.6340 | S | 2308.1150 | 2290.1045 | 2291.0879 | 1154.5575 | 19 |
| 7 | 631.3051 | 613.2946 | 614.2781 | 316.1526 | A | 2221.0830 | 2203.0725 | 2204.0559 | 1111.0415 | 18 |
| 8 | 718.3372 | 700.3266 | 701.3102 | 359.6686 | S | 2150.0459 | 2132.0354 | 2133.0188 | 1075.5229 | 17 |
| 9 | 847.3797 | 829.3691 | 830.3527 | 424.1898 | E | 2063.0139 | 2045.0033 | 2045.9868 | 1032.0070 | 16 |
| 10 | 948.4274 | 930.4169 | 931.4005 | 474.7137 | T | 1933.9712 | 1915.9607 | 1916.9442 | 967.4856 | 15 |
| 11 | 1077.4700 | 1059.4595 | 1060.4430 | 539.2350 | E | 1832.9236 | 1814.9131 | 1815.8966 | 916.9618 | 14 |
| 12 | 1206.5126 | 1188.5021 | 1189.4856 | 603.7563 | E | 1703.8810 | 1685.8705 | 1686.8540 | 852.4405 | 13 |
| 13 | 1321.5396 | 1303.5291 | 1304.5126 | 661.2698 | D | 1574.8384 | 1556.8279 | 1557.8114 | 787.9192 | 12 |
| 14 | 1422.5872 | 1404.5767 | 1405.5602 | 711.7936 | T | 1459.8030 | 1441.8009 | 1442.7844 | 730.4057 | 11 |
| 15 | 1521.6556 | 1503.6451 | 1504.6287 | 761.3278 | V | 1358.7638 | 1340.7533 | 1341.7368 | 679.8819 | 10 |
| 16 | 1608.6876 | 1590.6771 | 1591.6606 | 804.8438 | S | 1259.6969 | 1241.6848 | 1242.6683 | 630.3477 | 9 |
| 17 | 1707.7561 | 1689.7456 | 1690.7291 | 854.3781 | V | 1172.6600 | 1154.6528 | 1155.6364 | 586.8317 | 8 |
| 18 | 1794.7881 | 1776.7776 | 1777.7611 | 897.8940 | S | 1073.5902 | 1055.5844 | 1056.5679 | 537.2974 | 7 |
| 19 | 1994.9042 | 1976.8937 | 1977.8772 | 997.9521 | K(+72.02) | 986.5643 | 968.5523 | 969.5359 | 493.7814 | 6 |
| 20 | 2179.0254 | 2161.0149 | 2161.9983 | 1090.0127 | K(+56.03) | 786.4437 | 768.4362 | 769.4198 | 393.7234 | 5 |
| 21 | 2308.0679 | 2290.0574 | 2291.0408 | 1154.5339 | E | 602.3259 | 584.3151 | 585.2986 | 301.6628 | 4 |
| 22 | 2492.1892 | 2474.1787 | 2475.1621 | 1246.5946 | K(+56.03) | 473.2829 | 455.2725 | 456.2560 | 237.1415 | 3 |
| 23 | 2606.2319 | 2588.2214 | 2589.2048 | 1303.6160 | N | 289.1617 | 271.1513 | 272.1349 | 145.0809 | 2 |
| 24 | | | | | R | 175.1189 | 157.1084 | 158.0919 | 88.0595 | 1 |

**Figure S14_71**

**MSMS assignment of K331ox, K332ox, K334ox, K335ox in Splicing factor 3B subunit 2**

SF3B2(331-338): K*[O]K*[O]K*K*[O]K*PQR; -10lgP 26.70; Scan 8477 (MC304)

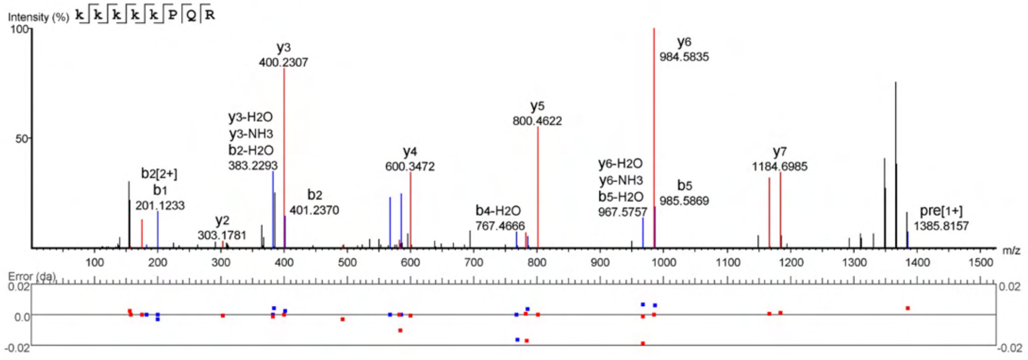


| # | b | b-H2O | b-NH3 | b (2+) | Seq | y | y-H2O | y-NH3 | y (2+) | # |
|---|---|---|---|---|---|---|---|---|---|---|
| 1 | 201.1233 | 183.1129 | 184.0969 | 101.0620 | K(+72.02) | | | | | 8 |
| 2 | 401.2370 | 383.2293 | 384.2085 | 201.1233 | K(+72.02) | 1184.6985 | 1166.6882 | 1167.6727 | 592.8499 | 7 |
| 3 | 585.3613 | 567.3503 | 568.3342 | 293.1806 | K(+56.03) | 984.5835 | 966.5745 | 967.5757 | 492.7953 | 6 |
| 4 | 785.4735 | 767.4666 | 768.4672 | 393.2386 | K(+72.02) | 800.4622 | 782.4512 | 783.4526 | 400.7312 | 5 |
| 5 | 985.5869 | 967.5757 | 968.5663 | 493.2967 | K(+72.02) | 600.3472 | 582.3356 | 583.3298 | 300.6732 | 4 |
| 6 | 1082.6461 | 1064.6356 | 1065.6191 | 541.8231 | P | 400.2307 | 382.2214 | 383.2046 | 200.6151 | 3 |
| 7 | 1210.7047 | 1192.6942 | 1193.6777 | 605.8524 | Q | 303.1781 | 285.1669 | 286.1505 | 152.0887 | 2 |
| 8 | | | | | R | 175.1192 | 157.1056 | 158.0922 | 88.0595 | 1 |

Figure S14_72

**MSMS assignment for K269ox in Splicing regulatory glutamine/lysine-rich protein 1**

SREK1(266-273): EK*IK*[O]EK*ER; -10lgP 24.73; Scan 10897 (MC315)

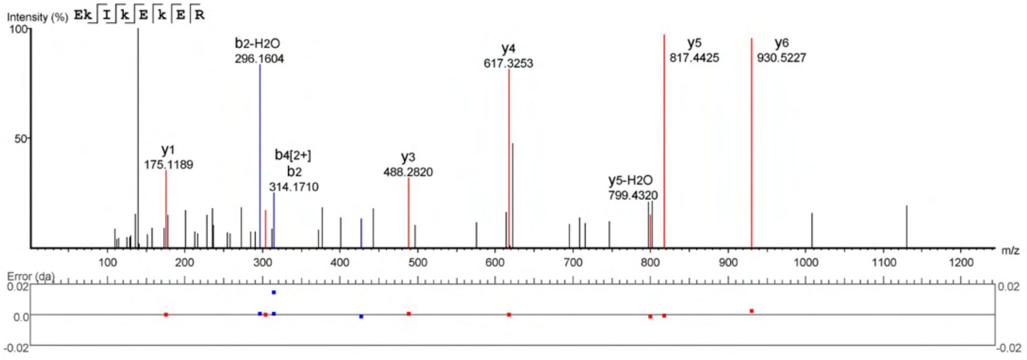


| # | b | b-H2O | b-NH3 | b (2+) | Seq | y | y-H2O | y-NH3 | y (2+) | # |
|---|---|---|---|---|---|---|---|---|---|---|
| 1 | 130.0504 | 112.0399 | 113.0234 | 65.5252 | E | | | | | 8 |
| 2 | 314.1710 | 296.1604 | 297.1446 | 157.5858 | K(+56.03) | 1114.6466 | 1096.6361 | 1097.6196 | 557.8233 | 7 |
| 3 | 427.2570 | 409.2451 | 410.2286 | 214.1278 | I | 930.5227 | 912.5148 | 913.4984 | 465.7627 | 6 |
| 4 | 627.3717 | 609.3611 | 610.3447 | 314.1710 | K(+72.02) | 817.4425 | 799.4320 | 800.4144 | 409.2207 | 5 |
| 5 | 756.4143 | 738.4037 | 739.3873 | 378.7072 | E | 617.3253 | 599.3147 | 600.2983 | 309.1626 | 4 |
| 6 | 940.5355 | 922.5249 | 923.5085 | 470.7677 | K(+56.03) | 488.2820 | 470.2721 | 471.2557 | 244.6413 | 3 |
| 7 | 1069.5781 | 1051.5676 | 1052.5511 | 535.2891 | E | 304.1610 | 286.1509 | 287.1345 | 152.5807 | 2 |
| 8 | | | | | R | 175.1189 | 157.1084 | 158.0919 | 88.0595 | 1 |

**Figure S14_73**

**MSMS assignment of K397ox, K400ox in Splicing regulatory glutamine/lysine-rich protein 1**

SREK1(395-401): TSK*[O]TIK*[O]R; -10lgP 34.43; Scan 6860  (MC315)

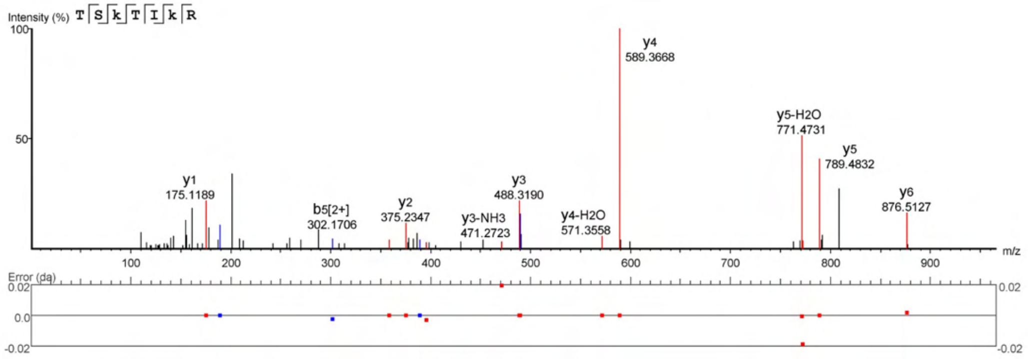


| # | b | b-H2O | b-NH3 | b (2+) | Seq | y | y-H2O | y-NH3 | y (2+) | # |
|---|---|---|---|---|---|---|---|---|---|---|
| 1 | 102.0555 | 84.0450 | 85.0285 | 51.5278 | T | | | | | 7 |
| 2 | 189.0871 | 171.0770 | 172.0605 | 95.0438 | S | 876.5127 | 858.5043 | 859.4879 | 438.7574 | 6 |
| 3 | 389.2031 | 371.1931 | 372.1766 | 195.1018 | K(+72.02) | 789.4832 | 771.4731 | 772.4751 | 395.2449 | 5 |
| 4 | 490.2513 | 472.2407 | 473.2243 | 245.6256 | T | 589.3668 | 571.3558 | 572.3398 | 295.1834 | 4 |
| 5 | 603.3353 | 585.3248 | 586.3083 | 302.1706 | I | 488.3190 | 470.3085 | 471.2723 | 244.6595 | 3 |
| 6 | 803.4514 | 785.4409 | 786.4244 | 402.2257 | K(+72.02) | 375.2347 | 357.2245 | 358.2077 | 188.1175 | 2 |
| 7 | | | | | R | 175.1189 | 157.1084 | 158.0919 | 88.0595 | 1 |

**Figure S14_74**

**Figure S14_76**

## MSMS assignment of K16ox, K18ox, K19ox in SRSF protein kinase 1

SRPK1(15-24): TK*[O]AK*[O]K*[O]DK*AQR; -10lgP 32.05; Scan 7719 (MC306)

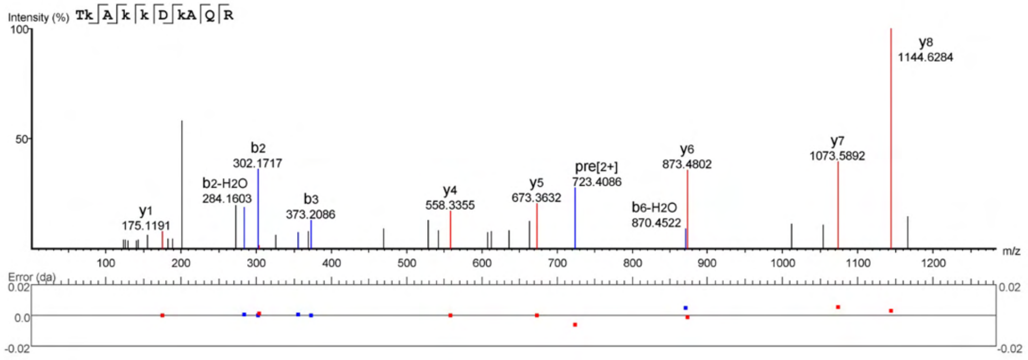


| # | b | b-H2O | b-NH3 | b (2+) | Seq | y | y-H2O | y-NH3 | y (2+) | # |
|---|---|---|---|---|---|---|---|---|---|---|
| 1 | 102.0555 | 84.0450 | 85.0285 | 51.5278 | T | | | | | 10 |
| 2 | 302.1717 | 284.1603 | 285.1446 | 151.5858 | K(+72.02) | 1344.7480 | 1326.7375 | 1327.7211 | 672.8740 | 9 |
| 3 | 373.2086 | 355.1972 | 356.1817 | 187.1043 | A | 1144.6284 | 1126.6215 | 1127.6050 | 572.8160 | 8 |
| 4 | 573.3248 | 555.3142 | 556.2978 | 287.1624 | K(+72.02) | 1073.5892 | 1055.5844 | 1056.5679 | 537.2974 | 7 |
| 5 | 773.4409 | 755.4303 | 756.4139 | 387.2204 | K(+72.02) | 873.4802 | 855.4683 | 856.4518 | 437.2394 | 6 |
| 6 | 888.4678 | 870.4522 | 871.4409 | 444.7339 | D | 673.3632 | 655.3522 | 656.3358 | 337.1814 | 5 |
| 7 | 1072.5890 | 1054.5785 | 1055.5620 | 536.7945 | K(+56.03) | 558.3355 | 540.3253 | 541.3088 | 279.6679 | 4 |
| 8 | 1143.6261 | 1125.6156 | 1126.5991 | 572.3130 | A | 374.2146 | 356.2040 | 357.1876 | 187.6073 | 3 |
| 9 | 1271.6847 | 1253.6742 | 1254.6577 | 636.3423 | Q | 303.1757 | 285.1669 | 286.1505 | 152.0887 | 2 |
| 10 | | | | | R | 175.1191 | 157.1084 | 158.0919 | 88.0595 | 1 |

Figure S14_77

**MSMS assignment of K242ox in Serine/arginine-rich splicing factor 11**

SRSF11(228-246): EAQSLISAAIEPDK*K*[O]EEK*R; -10lgP 65.23; Scan 21298 (MC303)

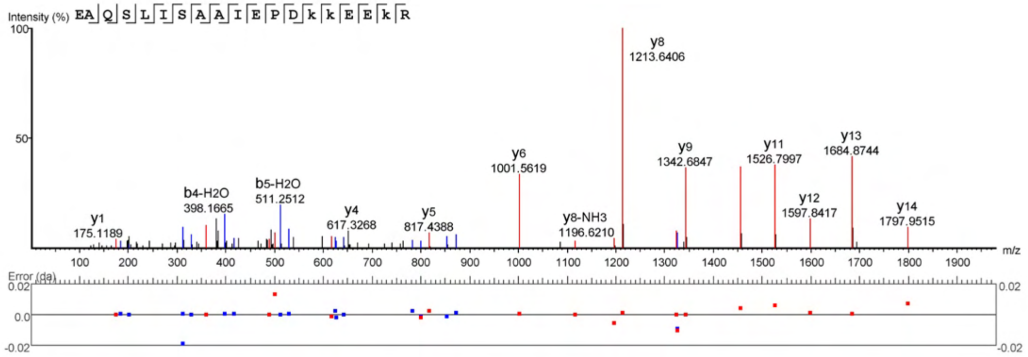


| # | b | b-H2O | b-NH3 | b (2+) | Seq | y | y-H2O | y-NH3 | y (2+) | # |
|---|---|---|---|---|---|---|---|---|---|---|
| 1 | 130.0504 | 112.0399 | 113.0234 | 65.5252 | E | | | | | 19 |
| 2 | 201.0872 | 183.0761 | 184.0605 | 101.0438 | A | 2197.1709 | 2179.1604 | 2180.1438 | 1099.0854 | 18 |
| 3 | 329.1457 | 311.1346 | 312.1381 | 165.0730 | Q | 2126.1338 | 2108.1233 | 2109.1067 | 1063.5669 | 17 |
| 4 | 416.1769 | 398.1665 | 399.1511 | 208.5891 | S | 1998.0753 | 1980.0648 | 1981.0483 | 999.5377 | 16 |
| 5 | 529.2615 | 511.2512 | 512.2352 | 265.1311 | L | 1911.0432 | 1893.0327 | 1894.0162 | 956.0216 | 15 |
| 6 | 642.3464 | 624.3329 | 625.3216 | 321.6731 | I | 1797.9515 | 1779.9487 | 1780.9323 | 899.4796 | 14 |
| 7 | 729.3783 | 711.3677 | 712.3513 | 365.1891 | S | 1684.8744 | 1666.8646 | 1667.8481 | 842.9376 | 13 |
| 8 | 800.4166 | 782.4020 | 783.3884 | 400.7077 | A | 1597.8417 | 1579.8326 | 1580.8162 | 799.4216 | 12 |
| 9 | 871.4507 | 853.4434 | 854.4255 | 436.2263 | A | 1526.7997 | 1508.7955 | 1509.7791 | 763.9030 | 11 |
| 10 | 984.5366 | 966.5260 | 967.5096 | 492.7683 | I | 1455.7645 | 1437.7584 | 1438.7419 | 728.3845 | 10 |
| 11 | 1113.5791 | 1095.5686 | 1096.5521 | 557.2896 | E | 1342.6847 | 1324.6748 | 1325.6685 | 671.8424 | 9 |
| 12 | 1210.6318 | 1192.6213 | 1193.6049 | 605.8159 | P | 1213.6406 | 1195.6317 | 1196.6210 | 607.3211 | 8 |
| 13 | 1325.6685 | 1307.6483 | 1308.6318 | 663.3294 | D | 1116.5896 | 1098.5790 | 1099.5625 | 558.7947 | 7 |
| 14 | 1509.7800 | 1491.7695 | 1492.7531 | 755.3900 | K(+56.03) | 1001.5619 | 983.5519 | 984.5355 | 501.2673 | 6 |
| 15 | 1709.8961 | 1691.8856 | 1692.8691 | 855.4481 | K(+72.02) | 817.4388 | 799.4308 | 800.4166 | 409.2207 | 5 |
| 16 | 1838.9387 | 1820.9282 | 1821.9117 | 919.9694 | E | 617.3268 | 599.3147 | 600.2983 | 309.1626 | 4 |
| 17 | 1967.9812 | 1949.9707 | 1950.9542 | 984.4906 | E | 488.2826 | 470.2721 | 471.2557 | 244.6413 | 3 |
| 18 | 2152.1025 | 2134.0920 | 2135.0754 | 1076.5513 | K(+56.03) | 359.2399 | 341.2296 | 342.2131 | 180.1201 | 2 |
| 19 | | | | | R | 175.1189 | 157.1084 | 158.0919 | 88.0595 | 1 |

Figure S14_78

**MSMS assignment of K314ox in Serine/arginine-rich splicing factor 11**

SRSF11(312-320): DK*K*[O]K*EDK*EK; -10lgP 22.49; Scan 10961 (MC304)

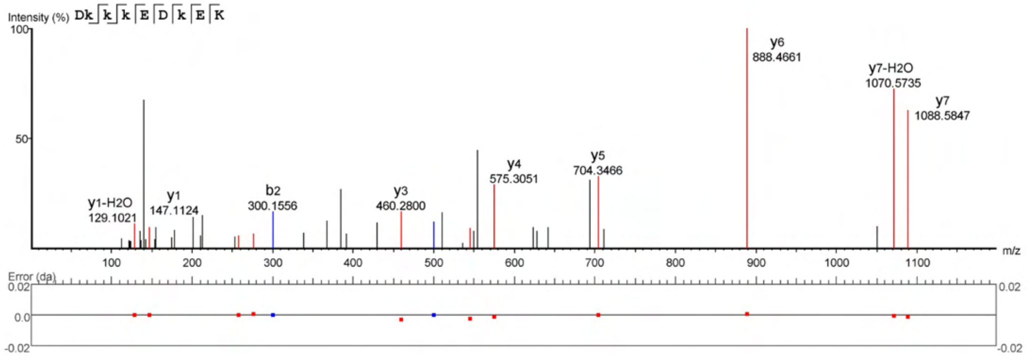


| # | b | b-H2O | b-NH3 | b (2+) | Seq | y | y-H2O | y-NH3 | y (2+) | # |
|---|---|---|---|---|---|---|---|---|---|---|
| 1 | 116.0348 | 98.0242 | 99.0078 | 58.5174 | D | | | | | 9 |
| 2 | 300.1556 | 282.1454 | 283.1289 | 150.5780 | K(+56.03) | 1272.7045 | 1254.6940 | 1255.6775 | 636.8522 | 8 |
| 3 | 500.2722 | 482.2614 | 483.2450 | 250.6360 | K(+72.02) | 1088.5847 | 1070.5735 | 1071.5564 | 544.7943 | 7 |
| 4 | 684.3932 | 666.3826 | 667.3662 | 342.6966 | K(+56.03) | 888.4661 | 870.4567 | 871.4402 | 444.7336 | 6 |
| 5 | 813.4358 | 795.4252 | 796.4088 | 407.2179 | E | 704.3466 | 686.3355 | 687.3191 | 352.6730 | 5 |
| 6 | 928.4627 | 910.4521 | 911.4357 | 464.7314 | D | 575.3051 | 557.2929 | 558.2765 | 288.1517 | 4 |
| 7 | 1112.5839 | 1094.5734 | 1095.5569 | 556.7919 | K(+56.03) | 460.2800 | 442.2660 | 443.2496 | 230.6383 | 3 |
| 8 | 1241.6265 | 1223.6160 | 1224.5995 | 621.3132 | E | 276.1547 | 258.1449 | 259.1284 | 138.5777 | 2 |
| 9 | | | | | K | 147.1124 | 129.1021 | 130.0858 | 74.0564 | 1 |

**Figure S14_79**

**MSMS assignment of K411ox in Serine/arginine-rich splicing factor 11**

SRSF11(411-423): K*[O]SESDK*DVK*QVTR; -10lgP 44.25; Scan 12062 (MC301)

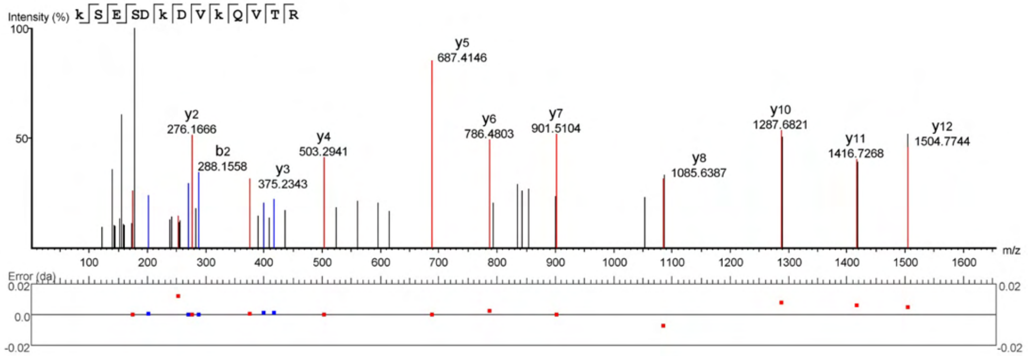


| # | b | b-H2O | b-NH3 | b (2+) | Seq | y | y-H2O | y-NH3 | y (2+) | # |
|---|---|---|---|---|---|---|---|---|---|---|
| 1 | 201.1230 | 183.1134 | 184.0969 | 101.0620 | K(+72.02) | | | | | 13 |
| 2 | 288.1558 | 270.1453 | 271.1290 | 144.5780 | S | 1503.7600 | 1485.7544 | 1486.7379 | 752.3824 | 12 |
| 3 | 417.1973 | 399.1866 | 400.1715 | 209.0993 | E | 1416.7268 | 1398.7224 | 1399.7059 | 708.8665 | 11 |
| 4 | 504.2305 | 486.2200 | 487.2036 | 252.6153 | S | 1287.6821 | 1269.6798 | 1270.6633 | 644.3452 | 10 |
| 5 | 619.2575 | 601.2469 | 602.2305 | 310.1288 | D | 1200.6582 | 1182.6477 | 1183.6312 | 600.8291 | 9 |
| 6 | 803.3787 | 785.3681 | 786.3517 | 402.1893 | K(+56.03) | 1085.6387 | 1067.6208 | 1068.6044 | 543.3157 | 8 |
| 7 | 918.4056 | 900.3950 | 901.3786 | 459.7028 | D | 901.5104 | 883.4996 | 884.4832 | 451.2551 | 7 |
| 8 | 1017.4740 | 999.4634 | 1000.4470 | 509.2370 | V | 786.4803 | 768.4726 | 769.4562 | 393.7416 | 6 |
| 9 | 1201.5952 | 1183.5847 | 1184.5682 | 601.2976 | K(+56.03) | 687.4146 | 669.4042 | 670.3878 | 344.2074 | 5 |
| 10 | 1329.6538 | 1311.6433 | 1312.6268 | 665.3269 | Q | 503.2941 | 485.2831 | 486.2666 | 252.1347 | 4 |
| 11 | 1428.7222 | 1410.7117 | 1411.6952 | 714.8611 | V | 375.2343 | 357.2245 | 358.2080 | 188.1175 | 3 |
| 12 | 1529.7699 | 1511.7594 | 1512.7429 | 765.3849 | T | 276.1666 | 258.1561 | 259.1396 | 138.5833 | 2 |
| 13 | | | | | R | 175.1188 | 157.1084 | 158.0919 | 88.0595 | 1 |

**Figure S14_80**

**MSMS assignment of K521ox in FACT complex subunit SSRP1**

SSRP1(518-529): K*QLK*[O]K*AK*MAK*DR; -10lgP 47.56; Scan 25968 (MC275)

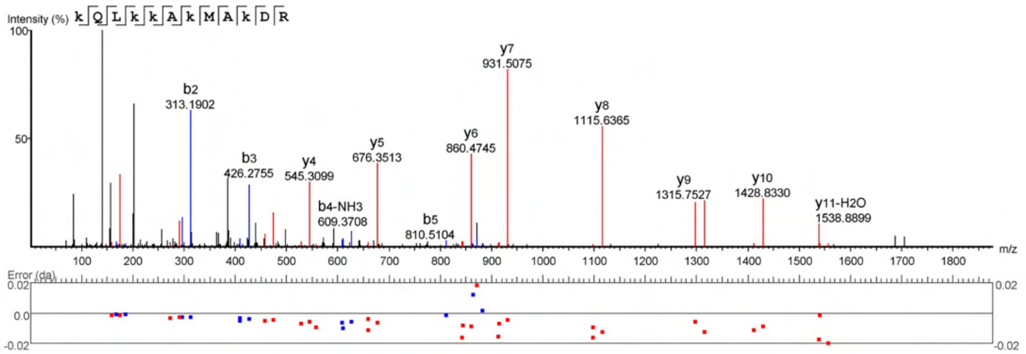


| # | b | b-H2O | b-NH3 | b (2+) | Seq | y | y-H2O | y-NH3 | y (2+) | # |
|---|---|---|---|---|---|---|---|---|---|---|
| 1 | 185.1299 | 167.1184 | 168.1027 | 93.0645 | K(+56.03) | | | | | 12 |
| 2 | 313.1902 | 295.1770 | 296.1635 | 157.0938 | Q | 1556.9026 | 1538.8899 | 1539.8572 | 778.9414 | 11 |
| 3 | 426.2755 | 408.2645 | 409.2497 | 213.6358 | L | 1428.8330 | 1410.8252 | 1411.7972 | 714.9121 | 10 |
| 4 | 626.3938 | 608.3837 | 609.3708 | 313.6938 | K(+72.02) | 1315.7527 | 1297.7354 | 1298.7133 | 658.3812 | 9 |
| 5 | 810.5104 | 792.4984 | 793.4819 | 405.7545 | K(+56.03) | 1115.6365 | 1097.6302 | 1098.6068 | 558.3219 | 8 |
| 6 | 881.5438 | 863.5230 | 864.5190 | 441.2730 | A | 931.5075 | 913.5080 | 914.4832 | 466.2515 | 7 |
| 7 | 1065.6671 | 1047.6566 | 1048.6401 | 533.3336 | K(+56.03) | 860.4745 | 842.4717 | 843.4471 | 430.7329 | 6 |
| 8 | 1196.7076 | 1178.6971 | 1179.6807 | 598.8538 | M | 676.3513 | 658.3341 | 659.3216 | 338.6723 | 5 |
| 9 | 1267.7448 | 1249.7343 | 1250.7178 | 634.3724 | A | 545.3099 | 527.2936 | 528.2840 | 273.1521 | 4 |
| 10 | 1451.8660 | 1433.8555 | 1434.8390 | 726.4330 | K(+56.03) | 474.2717 | 456.2565 | 457.2451 | 237.6335 | 3 |
| 11 | 1566.8928 | 1548.8823 | 1549.8658 | 783.9464 | D | 290.1489 | 272.1353 | 273.1220 | 145.5729 | 2 |
| 12 | | | | | R | 175.1204 | 157.1084 | 158.0938 | 88.0595 | 1 |

**Figure S14_81**

**MSMS assignment of K524ox in FACT complex subunit SSRP1**

SSRP1(518-529): K*QLK*K*AK*[O]MAK*DR; -10lgP 32.58; Scan 39541 (MC362)

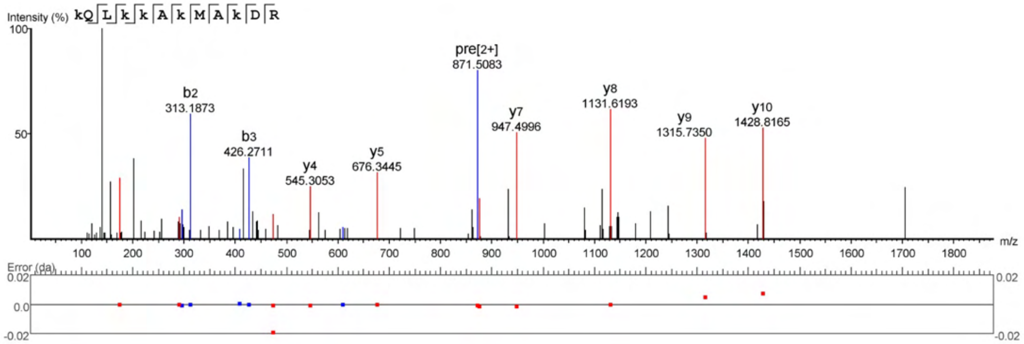


| # | b | b-H2O | b-NH3 | b (2+) | Seq | y | y-H2O | y-NH3 | y (2+) | # |
|---|---|---|---|---|---|---|---|---|---|---|
| 1 | 185.1290 | 167.1184 | 168.1020 | 93.0645 | K(+56.03) | | | | | 12 |
| 2 | 313.1873 | 295.1770 | 296.1612 | 157.0938 | Q | 1556.8828 | 1538.8723 | 1539.8558 | 778.9414 | 11 |
| 3 | 426.2711 | 408.2610 | 409.2434 | 213.6358 | L | 1428.8165 | 1410.8137 | 1411.7972 | 714.9121 | 10 |
| 4 | 610.3934 | 592.3823 | 593.3658 | 305.6964 | K(+56.03) | 1315.7350 | 1297.7297 | 1298.7133 | 658.3701 | 9 |
| 5 | 794.5140 | 776.5034 | 777.4870 | 397.7570 | K(+56.03) | 1131.6193 | 1113.6085 | 1114.5920 | 566.3095 | 8 |
| 6 | 865.5511 | 847.5405 | 848.5241 | 433.2755 | A | 947.4996 | 929.4873 | 930.4709 | 474.2682 | 7 |
| 7 | 1065.6671 | 1047.6566 | 1048.6401 | 533.3336 | K(+72.02) | 876.4622 | 858.4502 | 859.4338 | 438.7304 | 6 |
| 8 | 1196.7076 | 1178.6971 | 1179.6807 | 598.8538 | M | 676.3445 | 658.3341 | 659.3177 | 338.6723 | 5 |
| 9 | 1267.7448 | 1249.7343 | 1250.7178 | 634.3724 | A | 545.3053 | 527.2936 | 528.2772 | 273.1521 | 4 |
| 10 | 1451.8660 | 1433.8555 | 1434.8390 | 726.4330 | K(+56.03) | 474.2682 | 456.2565 | 457.2401 | 237.6335 | 3 |
| 11 | 1566.8928 | 1548.8823 | 1549.8658 | 783.9464 | D | 290.1463 | 272.1353 | 273.1189 | 145.5729 | 2 |
| 12 | | | | | R | 175.1190 | 157.1084 | 158.0919 | 88.0595 | 1 |

Figure S14_82

**MSMS assignment of K1348ox in Treacle protein**

TCOF1(1348-1357): K*[O]LSGDQPAAR; -10lgP 37.48; Scan 5589 (MC301)

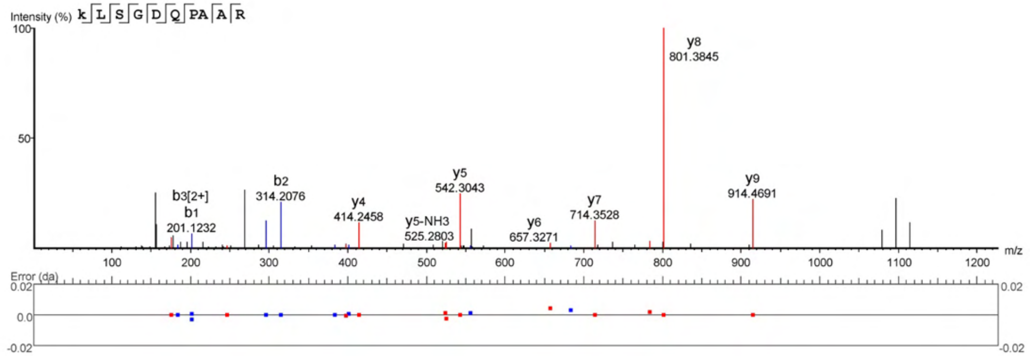


| # | b | b-H2O | b-NH3 | b (2+) | Seq | y | y-H2O | y-NH3 | y (2+) | # |
|---|---|---|---|---|---|---|---|---|---|---|
| 1 | 201.1232 | 183.1128 | 184.0969 | 101.0620 | K(+72.02) | | | | | 10 |
| 2 | 314.2076 | 296.1970 | 297.1810 | 157.6040 | L | 914.4691 | 896.4584 | 897.4420 | 457.7345 | 9 |
| 3 | 401.2390 | 383.2291 | 384.2130 | 201.1232 | S | 801.3845 | 783.3724 | 784.3579 | 401.1924 | 8 |
| 4 | 458.2614 | 440.2509 | 441.2345 | 229.6307 | G | 714.3528 | 696.3423 | 697.3259 | 357.6765 | 7 |
| 5 | 573.2884 | 555.2766 | 556.2614 | 287.1442 | D | 657.3271 | 639.3209 | 640.3044 | 329.1657 | 6 |
| 6 | 701.3470 | 683.3334 | 684.3200 | 351.1735 | Q | 542.3043 | 524.2927 | 525.2803 | 271.6523 | 5 |
| 7 | 798.3997 | 780.3892 | 781.3727 | 399.6999 | P | 414.2458 | 396.2354 | 397.2200 | 207.6230 | 4 |
| 8 | 869.4368 | 851.4263 | 852.4099 | 435.2184 | A | 317.1931 | 299.1826 | 300.1662 | 159.0966 | 3 |
| 9 | 940.4739 | 922.4634 | 923.4470 | 470.7370 | A | 246.1564 | 228.1455 | 229.1290 | 123.5780 | 2 |
| 10 | | | | | R | 175.1189 | 157.1084 | 158.0919 | 88.0595 | 1 |

Figure S14_83

**MSMS assignment of K1389ox in Treacle protein**

TCOF1(1382-1392): TSTTSK*GK*[O]AK*R; -10lgP 31.46; Scan 6621 (MC301)

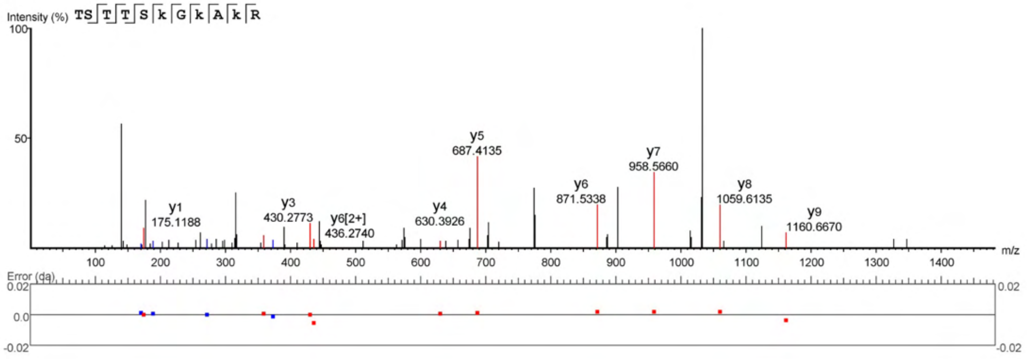


| # | b | b-H2O | b-NH3 | b (2+) | Seq | y | y-H2O | y-NH3 | y (2+) | # |
|---|---|---|---|---|---|---|---|---|---|---|
| 1 | 102.0555 | 84.0450 | 85.0285 | 51.5278 | T |  |  |  |  | 11 |
| 2 | 189.0868 | 171.0756 | 172.0605 | 95.0438 | S | 1247.6953 | 1229.6848 | 1230.6683 | 624.3477 | 10 |
| 3 | 290.1352 | 272.1248 | 273.1082 | 145.5676 | T | 1160.6670 | 1142.6528 | 1143.6364 | 580.8317 | 9 |
| 4 | 391.1829 | 373.1740 | 374.1559 | 196.0914 | T | 1059.6135 | 1041.6051 | 1042.5886 | 530.3078 | 8 |
| 5 | 478.2149 | 460.2043 | 461.1879 | 239.6075 | S | 958.5660 | 940.5574 | 941.5410 | 479.7840 | 7 |
| 6 | 662.3361 | 644.3255 | 645.3091 | 331.6680 | K(+56.03) | 871.5338 | 853.5254 | 854.5090 | 436.2740 | 6 |
| 7 | 719.3575 | 701.3470 | 702.3306 | 360.1788 | G | 687.4135 | 669.4042 | 670.3878 | 344.2074 | 5 |
| 8 | 919.4736 | 901.4631 | 902.4467 | 460.2368 | K(+72.02) | 630.3926 | 612.3828 | 613.3663 | 315.6967 | 4 |
| 9 | 990.5107 | 972.5002 | 973.4838 | 495.7554 | A | 430.2773 | 412.2667 | 413.2502 | 215.6386 | 3 |
| 10 | 1174.6320 | 1156.6215 | 1157.6050 | 587.8160 | K(+56.03) | 359.2393 | 341.2296 | 342.2131 | 180.1201 | 2 |
| 11 |  |  |  |  | R | 175.1188 | 157.1084 | 158.0919 | 88.0595 | 1 |

**Figure S14_84**

**MSMS assignment of K1440ox, K1442ox, K1443ox, K1444ox, K1447ox in Treacle protein**

TCOF1(1440-1448): K*[O]EK*[O]K*[O]K*[O]SDK*[O]R; -10lgP 22.72; Scan 6898 (MC302)

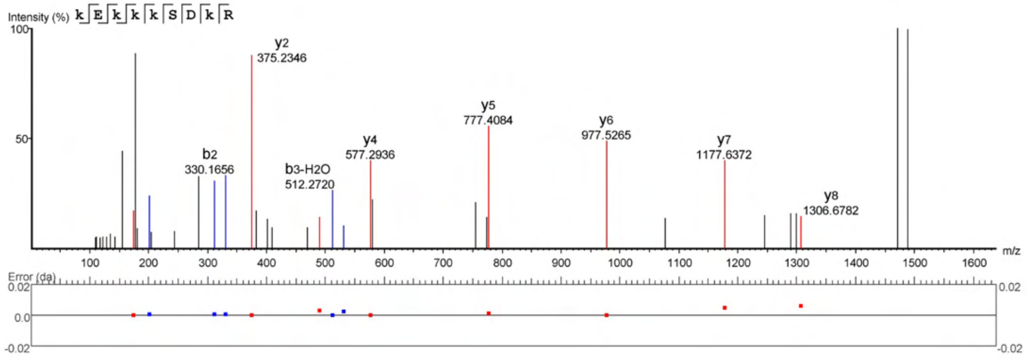


| # | b | b-H2O | b-NH3 | b (2+) | Seq | y | y-H2O | y-NH3 | y (2+) | # |
|---|---|---|---|---|---|---|---|---|---|---|
| 1 | 201.1233 | 183.1134 | 184.0969 | 101.0620 | K(+72.02) | | | | | 9 |
| 2 | 330.1656 | 312.1548 | 313.1395 | 165.5833 | E | 1306.6782 | 1288.6743 | 1289.6578 | 653.8424 | 8 |
| 3 | 530.2797 | 512.2720 | 513.2556 | 265.6413 | K(+72.02) | 1177.6372 | 1159.6317 | 1160.6152 | 589.3211 | 7 |
| 4 | 730.3987 | 712.3881 | 713.3717 | 365.6993 | K(+72.02) | 977.5265 | 959.5156 | 960.4992 | 489.2631 | 6 |
| 5 | 930.5148 | 912.5042 | 913.4878 | 465.7574 | K(+72.02) | 777.4084 | 759.3995 | 760.3831 | 389.2050 | 5 |
| 6 | 1017.5468 | 999.5362 | 1000.5198 | 509.2734 | S | 577.2936 | 559.2834 | 560.2670 | 289.1470 | 4 |
| 7 | 1132.5737 | 1114.5632 | 1115.5468 | 566.7869 | D | 490.2589 | 472.2514 | 473.2350 | 245.6310 | 3 |
| 8 | 1332.6898 | 1314.6793 | 1315.6628 | 666.8449 | K(+72.02) | 375.2346 | 357.2245 | 358.2080 | 188.1175 | 2 |
| 9 | | | | | R | 175.1187 | 157.1084 | 158.0919 | 88.0595 | 1 |

Figure S14_85

**MSMS assignment of K25ox in DNA topoisomerase 1**

TOP1(18-31): LNDSHK*HK*[O]DK*HK*DR; -10lgP 52.38; Scan 10714 (MC315)

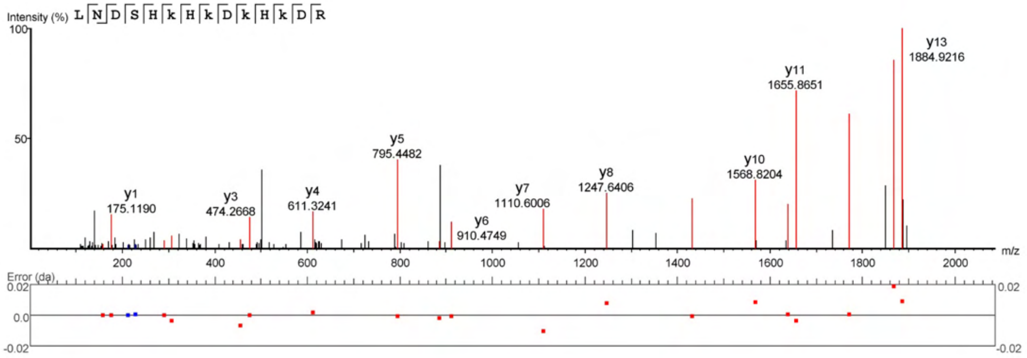


| # | b | b-H2O | b-NH3 | b (2+) | Seq | y | y-H2O | y-NH3 | y (2+) | # |
|---|---|---|---|---|---|---|---|---|---|---|
| 1 | 114.0919 | 96.0813 | 97.0649 | 57.5459 | L | | | | | 14 |
| 2 | 228.1338 | 210.1243 | 211.1075 | 114.5674 | N | 1884.9216 | 1866.9016 | 1867.9041 | 942.9655 | 13 |
| 3 | 343.1617 | 325.1512 | 326.1348 | 172.0809 | D | 1770.8873 | 1752.8776 | 1753.8611 | 885.9462 | 12 |
| 4 | 430.1938 | 412.1832 | 413.1668 | 215.5969 | S | 1655.8651 | 1637.8499 | 1638.8342 | 828.4306 | 11 |
| 5 | 567.2527 | 549.2421 | 550.2257 | 284.1263 | H | 1568.8204 | 1550.8186 | 1551.8021 | 784.9146 | 10 |
| 6 | 751.3738 | 733.3633 | 734.3469 | 376.1869 | K(+56.03) | 1431.7714 | 1413.7598 | 1414.7433 | 716.3851 | 9 |
| 7 | 888.4327 | 870.4222 | 871.4058 | 444.7164 | H | 1247.6406 | 1229.6385 | 1230.6221 | 624.3245 | 8 |
| 8 | 1088.5488 | 1070.5383 | 1071.5219 | 544.7744 | K(+72.02) | 1110.6006 | 1092.5796 | 1093.5631 | 555.7950 | 7 |
| 9 | 1203.5758 | 1185.5653 | 1186.5488 | 602.2879 | D | 910.4749 | 892.4635 | 893.4471 | 455.7437 | 6 |
| 10 | 1387.6970 | 1369.6865 | 1370.6700 | 694.3485 | K(+56.03) | 795.4482 | 777.4366 | 778.4202 | 398.2236 | 5 |
| 11 | 1524.7559 | 1506.7454 | 1507.7289 | 762.8779 | H | 611.3241 | 593.3154 | 594.2990 | 306.1672 | 4 |
| 12 | 1708.8771 | 1690.8666 | 1691.8501 | 854.9385 | K(+56.03) | 474.2668 | 456.2565 | 457.2401 | 237.6335 | 3 |
| 13 | 1823.9041 | 1805.8936 | 1806.8771 | 912.4520 | D | 290.1461 | 272.1353 | 273.1189 | 145.5729 | 2 |
| 14 | | | | | R | 175.1190 | 157.1084 | 158.0923 | 88.0595 | 1 |

**Figure S14_86**

**MSMS assignment of K36ox, K39ox in DNA topoisomerase 1**

TOP1(35-44): HK*[O]EHK*[O]K*EK*DR; -10lgP 47.29; Scan 5788 (MC306)

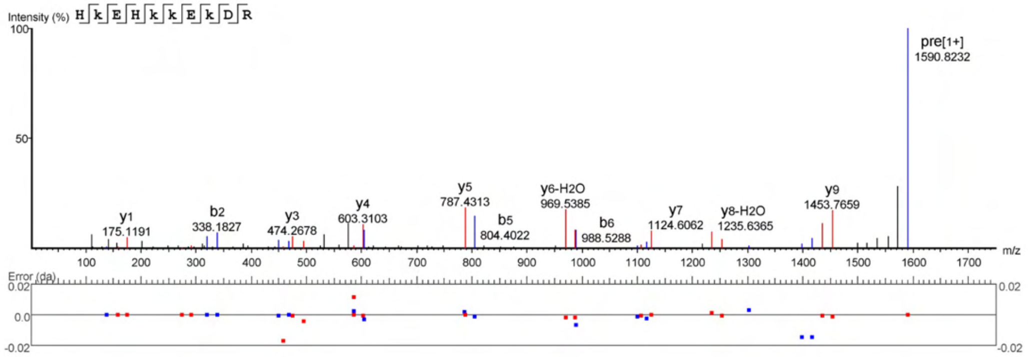


| # | b | b-H2O | b-NH3 | b (2+) | Seq | y | y-H2O | y-NH3 | y (2+) | # |
|---|---|---|---|---|---|---|---|---|---|---|
| 1 | 138.0664 | 120.0562 | 121.0397 | 69.5334 | H | | | | | 10 |
| 2 | 338.1827 | 320.1719 | 321.1558 | 169.5914 | K(+72.02) | 1453.7659 | 1435.7546 | 1436.7375 | 727.3823 | 9 |
| 3 | 467.2256 | 449.2156 | 450.1984 | 234.1127 | E | 1253.6490 | 1235.6365 | 1236.6215 | 627.3242 | 8 |
| 4 | 604.2874 | 586.2711 | 587.2573 | 302.6422 | H | 1124.6062 | 1106.5962 | 1107.5789 | 562.8029 | 7 |
| 5 | 804.4022 | 786.3880 | 787.3734 | 402.7002 | K(+72.02) | 987.5487 | 969.5385 | 970.5199 | 494.2782 | 6 |
| 6 | 988.5288 | 970.5110 | 971.4946 | 494.7608 | K(+56.03) | 787.4313 | 769.4202 | 770.4038 | 394.2154 | 5 |
| 7 | 1117.5667 | 1099.5554 | 1100.5372 | 559.2821 | E | 603.3103 | 585.2988 | 586.2711 | 302.1548 | 4 |
| 8 | 1301.6821 | 1283.6748 | 1284.6583 | 651.3427 | K(+56.03) | 474.2678 | 456.2565 | 457.2572 | 237.6335 | 3 |
| 9 | 1416.7274 | 1398.7164 | 1399.6853 | 708.8561 | D | 290.1460 | 272.1353 | 273.1186 | 145.5729 | 2 |
| 10 | | | | | R | 175.1191 | 157.1084 | 158.0925 | 88.0595 | 1 |

**Figure S14_87**

## MSMS assignment of K40ox in DNA topoisomerase 1

TOP1(35-44): HK*[O]EHK*[O]K*[O]EK*DR; -10lgP 33.51; Scan 5452 (MC306)

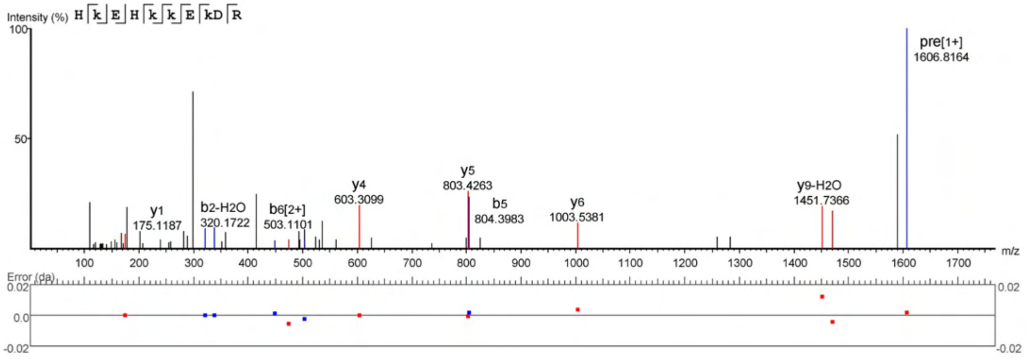


| # | b | b-H2O | b-NH3 | b (2+) | Seq | y | y-H2O | y-NH3 | y (2+) | # |
|---|---|---|---|---|---|---|---|---|---|---|
| 1 | 138.0667 | 120.0562 | 121.0397 | 69.5334 | H | | | | | 10 |
| 2 | 338.1823 | 320.1722 | 321.1558 | 169.5914 | K(+72.02) | 1469.7638 | 1451.7366 | 1452.7324 | 735.3797 | 9 |
| 3 | 467.2254 | 449.2135 | 450.1984 | 234.1127 | E | 1269.6433 | 1251.6328 | 1252.6163 | 635.3217 | 8 |
| 4 | 604.2843 | 586.2737 | 587.2573 | 302.6422 | H | 1140.6007 | 1122.5902 | 1123.5737 | 570.8004 | 7 |
| 5 | 804.3983 | 786.3898 | 787.3734 | 402.7002 | K(+72.02) | 1003.5381 | 985.5312 | 986.5148 | 502.2709 | 6 |
| 6 | 1004.5165 | 986.5059 | 987.4895 | 502.7608 | K(+72.02) | 803.4263 | 785.4152 | 786.3987 | 402.2129 | 5 |
| 7 | 1133.5591 | 1115.5486 | 1116.5321 | 567.2795 | E | 603.3099 | 585.2991 | 586.2827 | 302.1548 | 4 |
| 8 | 1317.6803 | 1299.6698 | 1300.6533 | 659.3401 | K(+56.03) | 474.2725 | 456.2565 | 457.2401 | 237.6335 | 3 |
| 9 | 1432.7072 | 1414.6967 | 1415.6802 | 716.8536 | D | 290.1459 | 272.1353 | 273.1189 | 145.5729 | 2 |
| 10 | | | | | R | 175.1187 | 157.1084 | 158.0919 | 88.0595 | 1 |

Figure S14_88

**MSMS assignment of K159ox in DNA topoisomerase 1**

TOP1(154-163): TEDTK*K*[O]EK*K*R; -10lgP 32.64; Scan 10327 (MC306)

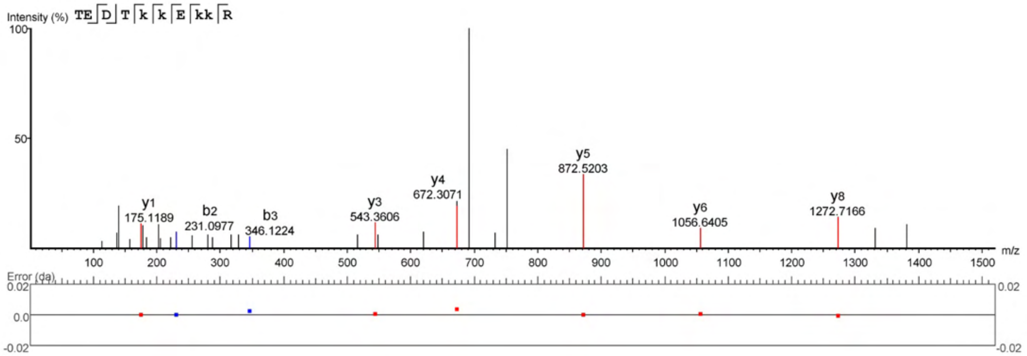


| # | b | b-H2O | b-NH3 | b (2+) | Seq | y | y-H2O | y-NH3 | y (2+) | # |
|---|---|---|---|---|---|---|---|---|---|---|
| 1 | 102.0555 | 84.0450 | 85.0285 | 51.5278 | T | | | | | 10 |
| 2 | 231.0977 | 213.0875 | 214.0711 | 116.0490 | E | 1401.7583 | 1383.7478 | 1384.7313 | 701.3792 | 9 |
| 3 | 346.1224 | 328.1145 | 329.0981 | 173.5625 | D | 1272.7166 | 1254.7052 | 1255.6887 | 636.8578 | 8 |
| 4 | 447.1727 | 429.1621 | 430.1457 | 224.0863 | T | 1157.6888 | 1139.6783 | 1140.6619 | 579.3444 | 7 |
| 5 | 631.2939 | 613.2833 | 614.2669 | 316.1469 | K(+56.03) | 1056.6405 | 1038.6306 | 1039.6141 | 528.8206 | 6 |
| 6 | 831.4100 | 813.3994 | 814.3830 | 416.2050 | K(+72.02) | 872.5203 | 854.5094 | 855.4930 | 436.7600 | 5 |
| 7 | 960.4526 | 942.4420 | 943.4256 | 480.7263 | E | 672.4001 | 654.3933 | 655.3769 | 336.7019 | 4 |
| 8 | 1144.5737 | 1126.5632 | 1127.5468 | 572.7869 | K(+56.03) | 543.3606 | 525.3507 | 526.3343 | 272.1806 | 3 |
| 9 | 1328.6949 | 1310.6844 | 1311.6680 | 664.8475 | K(+56.03) | 359.2401 | 341.2296 | 342.2131 | 180.1201 | 2 |
| 10 | | | | | R | 175.1189 | 157.1084 | 158.0919 | 88.0595 | 1 |

**Figure S14_89**

**MSMS assignment of K42ox in Pre-rRNA-processing protein TSR1 homolog**

TSR1(35-45): LALK*TLSK*[O]K*VR; -10lgP 24.97; Scan 50609 (MC357)

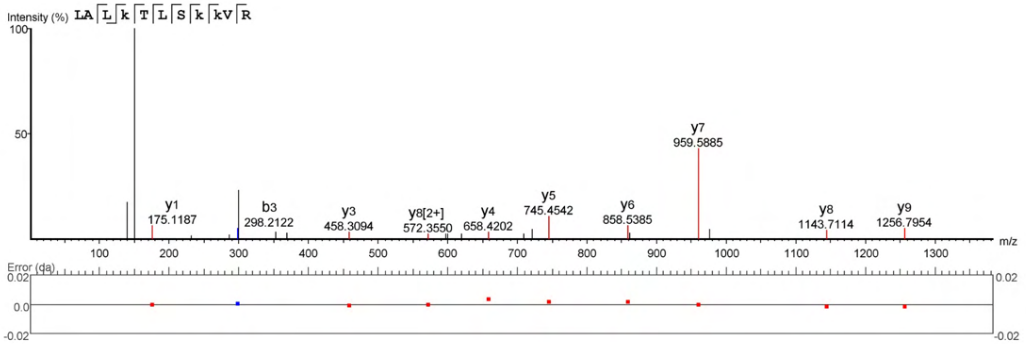


| # | b | b-H2O | b-NH3 | b (2+) | Seq | y | y-H2O | y-NH3 | y (2+) | # |
|---|---|---|---|---|---|---|---|---|---|---|
| 1 | 114.0919 | 96.0813 | 97.0649 | 57.5459 | L | | | | | 11 |
| 2 | 185.1290 | 167.1184 | 168.1020 | 93.0645 | A | 1327.8307 | 1309.8202 | 1310.8037 | 664.4153 | 10 |
| 3 | 298.2122 | 280.2025 | 281.1861 | 149.6065 | L | 1256.7954 | 1238.7831 | 1239.7666 | 628.8968 | 9 |
| 4 | 482.3342 | 464.3237 | 465.3073 | 241.6671 | K(+56.03) | 1143.7114 | 1125.6991 | 1126.6826 | 572.3550 | 8 |
| 5 | 583.3819 | 565.3713 | 566.3549 | 292.1909 | T | 959.5885 | 941.5778 | 942.5614 | 480.2942 | 7 |
| 6 | 696.4659 | 678.4554 | 679.4390 | 348.7330 | L | 858.5385 | 840.5302 | 841.5137 | 429.7704 | 6 |
| 7 | 783.4980 | 765.4874 | 766.4710 | 392.2490 | S | 745.4542 | 727.4460 | 728.4296 | 373.2283 | 5 |
| 8 | 983.6141 | 965.6035 | 966.5871 | 492.3070 | K(+72.02) | 658.4202 | 640.4141 | 641.3976 | 329.7123 | 4 |
| 9 | 1167.7352 | 1149.7247 | 1150.7083 | 584.3676 | K(+56.03) | 458.3094 | 440.2979 | 441.2815 | 229.6543 | 3 |
| 10 | 1266.8037 | 1248.7932 | 1249.7767 | 633.9019 | V | 274.1873 | 256.1768 | 257.1604 | 137.5937 | 2 |
| 11 | | | | | R | 175.1187 | 157.1084 | 158.0919 | 88.0595 | 1 |

Figure S14_90

**MSMS assignment of K15ox in Splicing factor U2AF 65 kDa subunit**

U2AF2(10-18): QLNENK*[O]QER; -10lgP 45.85; Scan 5669 (MC311)

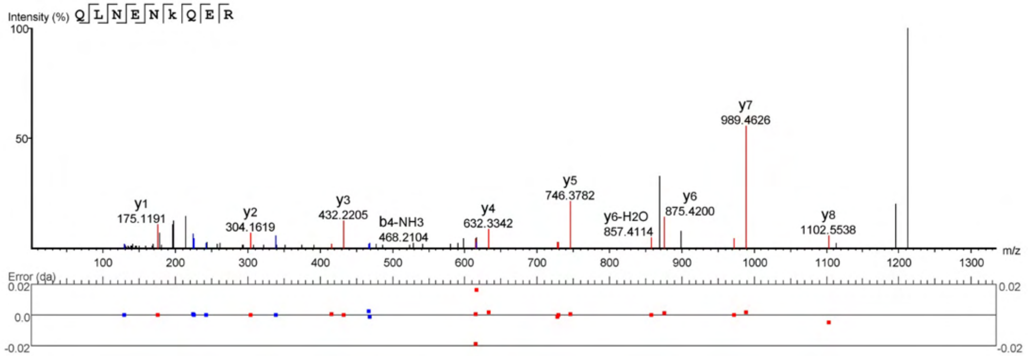


| # | b | b-H2O | b-NH3 | b (2+) | Seq | y | y-H2O | y-NH3 | y (2+) | # |
|---|---|---|---|---|---|---|---|---|---|---|
| 1 | 129.0661 | 111.0558 | 112.0394 | 65.0332 | Q | | | | | 9 |
| 2 | 242.1504 | 224.1390 | 225.1237 | 121.5752 | L | 1102.5538 | 1084.5382 | 1085.5217 | 551.7744 | 8 |
| 3 | 356.1934 | 338.1826 | 339.1665 | 178.5967 | N | 989.4626 | 971.4540 | 972.4381 | 495.2323 | 7 |
| 4 | 485.2360 | 467.2226 | 468.2104 | 243.1180 | E | 875.4200 | 857.4114 | 858.3947 | 438.2108 | 6 |
| 5 | 599.2789 | 581.2684 | 582.2520 | 300.1395 | N | 746.3782 | 728.3702 | 729.3519 | 373.6895 | 5 |
| 6 | 799.3950 | 781.3845 | 782.3680 | 400.1975 | K(+72.02) | 632.3342 | 614.3245 | 615.3284 | 316.6681 | 4 |
| 7 | 927.4536 | 909.4430 | 910.4266 | 464.2268 | Q | 432.2205 | 414.2095 | 415.1921 | 216.6100 | 3 |
| 8 | 1056.4961 | 1038.4856 | 1039.4691 | 528.7480 | E | 304.1619 | 286.1509 | 287.1345 | 152.5807 | 2 |
| 9 | | | | | R | 175.1191 | 157.1084 | 158.0919 | 88.0595 | 1 |

**Figure S14_91**

**MSMS assignment of K70ox in Splicing factor U2AF 65 kDa subunit**

U2AF2(68-78): GAK*[O]EEHGGLIR; -10lgP 48.53; Scan 6473 (MC317)

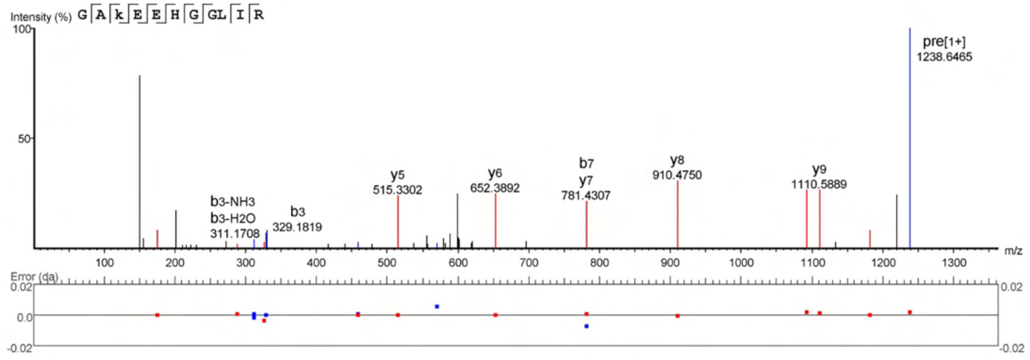


| # | b | b-H2O | b-NH3 | b (2+) | Seq | y | y-H2O | y-NH3 | y (2+) | # |
|---|---|---|---|---|---|---|---|---|---|---|
| 1 | 58.0293 | 40.0187 | 41.0023 | 29.5146 | G | | | | | 11 |
| 2 | 129.0664 | 111.0558 | 112.0394 | 65.0332 | A | 1181.6277 | 1163.6167 | 1164.6002 | 591.3136 | 10 |
| 3 | 329.1819 | 311.1708 | 312.1575 | 165.0912 | K(+72.02) | 1110.5889 | 1092.5776 | 1093.5631 | 555.7950 | 9 |
| 4 | 458.2239 | 440.2145 | 441.1981 | 229.6125 | E | 910.4750 | 892.4635 | 893.4471 | 455.7370 | 8 |
| 5 | 587.2676 | 569.2571 | 570.2350 | 294.1338 | E | 781.4307 | 763.4209 | 764.4045 | 391.2157 | 7 |
| 6 | 724.3265 | 706.3160 | 707.2996 | 362.6633 | H | 652.3892 | 634.3784 | 635.3619 | 326.6984 | 6 |
| 7 | 781.3553 | 763.3375 | 764.3210 | 391.1740 | G | 515.3302 | 497.3194 | 498.3030 | 258.1650 | 5 |
| 8 | 838.3695 | 820.3589 | 821.3425 | 419.6848 | G | 458.3085 | 440.2979 | 441.2815 | 229.6543 | 4 |
| 9 | 951.4536 | 933.4430 | 934.4266 | 476.2268 | L | 401.2870 | 383.2765 | 384.2601 | 201.1435 | 3 |
| 10 | 1064.5376 | 1046.5271 | 1047.5106 | 532.7688 | I | 288.2021 | 270.1924 | 271.1760 | 144.6015 | 2 |
| 11 | | | | | R | 175.1188 | 157.1084 | 158.0919 | 88.0595 | 1 |

**Figure S14_92**

**MSMS assignment of K962ox in U2 snRNP-associated SURP motif-containing protein**

SR140(960-966): SHK*[O]ESSR; -10lgP 35.97; Scan 3529 (MC310)

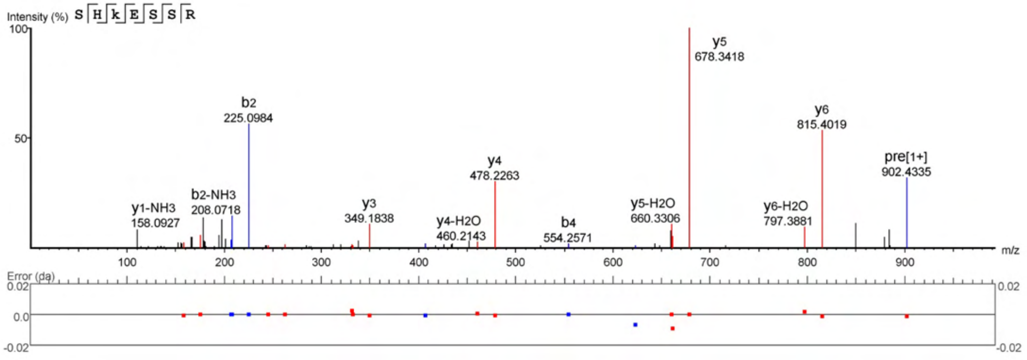


| # | b | b-H2O | b-NH3 | b (2+) | Seq | y | y-H2O | y-NH3 | y (2+) | # |
|---|---|---|---|---|---|---|---|---|---|---|
| 1 | 88.0399 | 70.0293 | 71.0129 | 44.5199 | S | | | | | 7 |
| 2 | 225.0984 | 207.0878 | 208.0718 | 113.0494 | H | 815.4019 | 797.3881 | 798.3736 | 408.2003 | 6 |
| 3 | 425.2148 | 407.2049 | 408.1879 | 213.1074 | K(+72.02) | 678.3418 | 660.3306 | 661.3242 | 339.6708 | 5 |
| 4 | 554.2571 | 536.2469 | 537.2305 | 277.6287 | E | 478.2263 | 460.2143 | 461.1986 | 239.6128 | 4 |
| 5 | 641.2894 | 623.2859 | 624.2625 | 321.1447 | S | 349.1838 | 331.1698 | 332.1561 | 175.0915 | 3 |
| 6 | 728.3215 | 710.3109 | 711.2945 | 364.6607 | S | 262.1516 | 244.1404 | 245.1245 | 131.5755 | 2 |
| 7 | | | | | R | 175.1191 | 157.1084 | 158.0927 | 88.0595 | 1 |

Figure S14_93

**MSMS assignment of K972ox in U2 snRNP-associated SURP motif-containing protein**

SR140(969-976): SSHK*[O]DSPR; -10lgP 34.55; Scan 4219 (MC310)

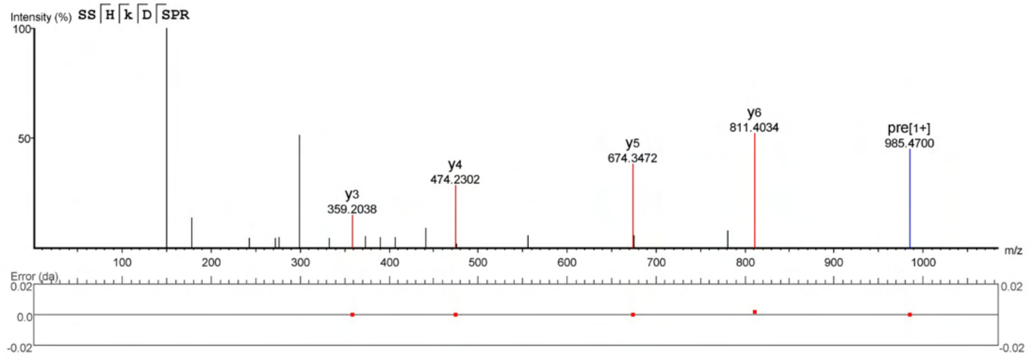


| # | b | b-H2O | b-NH3 | b (2+) | Seq | y | y-H2O | y-NH3 | y (2+) | # |
|---|---|---|---|---|---|---|---|---|---|---|
| 1 | 88.0399 | 70.0293 | 71.0129 | 44.5199 | S | | | | | 8 |
| 2 | 175.0719 | 157.0613 | 158.0449 | 88.0359 | S | 898.4377 | 880.4271 | 881.4107 | 449.7188 | 7 |
| 3 | 312.1308 | 294.1202 | 295.1038 | 156.5654 | H | 811.4034 | 793.3951 | 794.3787 | 406.2028 | 6 |
| 4 | 512.2469 | 494.2363 | 495.2199 | 256.6234 | K(+72.02) | 674.3472 | 656.3362 | 657.3198 | 337.6734 | 5 |
| 5 | 627.2738 | 609.2632 | 610.2468 | 314.1369 | D | 474.2302 | 456.2201 | 457.2037 | 237.6153 | 4 |
| 6 | 714.3058 | 696.2953 | 697.2789 | 357.6529 | S | 359.2038 | 341.1931 | 342.1767 | 180.1019 | 3 |
| 7 | 811.3586 | 793.3480 | 794.3316 | 406.1793 | P | 272.1717 | 254.1611 | 255.1447 | 136.5858 | 2 |
| 8 | | | | | R | 175.1189 | 157.1084 | 158.0919 | 88.0595 | 1 |

Figure S14_94

**MSMS assignment of K980ox, K981ox in U2 snRNP-associated SURP motif-containing protein**

SR140(977-984): DVSK*[O]K*[O]AK*R; -10lgP 36.37; Scan 7728 (MC313)

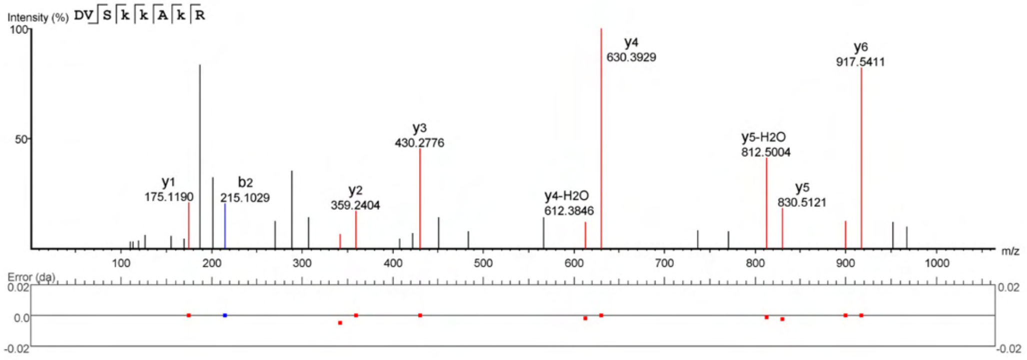


| # | b | b-H2O | b-NH3 | b (2+) | Seq | y | y-H2O | y-NH3 | y (2+) | # |
|---|---|---|---|---|---|---|---|---|---|---|
| 1 | 116.0348 | 98.0242 | 99.0078 | 58.5174 | D | | | | | 8 |
| 2 | 215.1029 | 197.0926 | 198.0762 | 108.0516 | V | 1016.6098 | 998.5992 | 999.5828 | 508.8049 | 7 |
| 3 | 302.1352 | 284.1246 | 285.1082 | 151.5676 | S | 917.5411 | 899.5303 | 900.5144 | 459.2707 | 6 |
| 4 | 502.2513 | 484.2407 | 485.2243 | 251.6256 | K(+72.02) | 830.5121 | 812.5004 | 813.4824 | 415.7547 | 5 |
| 5 | 702.3674 | 684.3568 | 685.3404 | 351.6837 | K(+72.02) | 630.3929 | 612.3846 | 613.3663 | 315.6967 | 4 |
| 6 | 773.4045 | 755.3939 | 756.3775 | 387.2022 | A | 430.2776 | 412.2667 | 413.2502 | 215.6386 | 3 |
| 7 | 957.5256 | 939.5151 | 940.4987 | 479.2628 | K(+56.03) | 359.2404 | 341.2296 | 342.2182 | 180.1201 | 2 |
| 8 | | | | | R | 175.1190 | 157.1084 | 158.0919 | 88.0595 | 1 |
